# Biallelic mutations in *MOS* cause female infertility characterized by human early embryonic arrest and fragmentation

**DOI:** 10.15252/emmm.202114887

**Published:** 2021-11-15

**Authors:** Yin‐Li Zhang, Wei Zheng, Peipei Ren, Huiling Hu, Xiaomei Tong, Shuo‐Ping Zhang, Xiang Li, Haichao Wang, Jun‐Chao Jiang, Jiamin Jin, Weijie Yang, Lanrui Cao, Yuanlin He, Yerong Ma, Yingyi Zhang, Yifan Gu, Liang Hu, Keli Luo, Fei Gong, Guang‐Xiu Lu, Ge Lin, Heng‐Yu Fan, Songying Zhang

**Affiliations:** ^1^ Assisted Reproduction Unit Department of Obstetrics and Gynecology Sir Run Run Shaw Hospital School of Medicine Zhejiang University Hangzhou China; ^2^ Key Laboratory of Reproductive Dysfunction Management of Zhejiang Province Hangzhou China; ^3^ Clinical Research Center for Reproduction and Genetics in Hunan Province, Reproductive and Genetic Hospital of CITIC‐Xiangya Changsha China; ^4^ Laboratory of Reproductive and Stem Cell Engineering, NHC Key Laboratory of Human Stem Cell and Reproductive Engineering Central South University Changsha China; ^5^ Life Sciences Institute Zhejiang University Hangzhou China; ^6^ Department of Epidemiology Center for Global Health School of Public Health Nanjing Medical University Nanjing China

**Keywords:** female infertility, human oocyte, maternal mRNA decay, mitochondria, *MOS*, Development, Urogenital System

## Abstract

Early embryonic arrest and fragmentation (EEAF) is a common phenomenon leading to female infertility, but the genetic determinants remain largely unknown. The Moloney sarcoma oncogene (*MOS*) encodes a serine/threonine kinase that activates the ERK signaling cascade during oocyte maturation in vertebrates. Here, we identified four rare variants of *MOS* in three infertile female individuals with EEAF that followed a recessive inheritance pattern. These *MOS* variants encoded proteins that resulted in decreased phosphorylated ERK1/2 level in cells and oocytes, and displayed attenuated rescuing effects on cortical F‐actin assembly. Using oocyte‐specific *Erk1/2* knockout mice, we verified that MOS‐ERK signal pathway inactivation in oocytes caused EEAF as human. The RNA sequencing data revealed that maternal mRNA clearance was disrupted in human mature oocytes either with *MOS* homozygous variant or with U0126 treatment, especially genes relative to mitochondrial function. Mitochondrial dysfunction was observed in oocytes with ERK1/2 deficiency or inactivation. In conclusion, this study not only uncovers biallelic *MOS* variants causes EEAF but also demonstrates that MOS‐ERK signaling pathway drives human oocyte cytoplasmic maturation to prevent EEAF.

## Graphical abstract

Biallelic variants in MOS gene cause recurrent early embryonic arrest and fragmentation (EEAF) and female infertility. MOS variants impair activation of MOS‐ERK signal cascade, prevent substantial maternal mRNAs decay and hamper embryonic development both in human and mice.

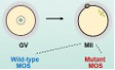

**The paper explained**

**Problem**

Early embryonic arrest and fragmentation (EEAF) is a compound phenotype of cleavage arrest and embryo fragmentation, leading to recurrent failure of assisted reproductive technology treatment and female infertility. In recent years, several pathogenic variants in maternal genes could account for 10–15% of the etiology of embryonic arrest. However, the underlying genetic determinants and mechanism of EEAF are largely unknown.

**Results**

We identified three patients bearing biallelic mutations in Moloney sarcoma oncogene (*MOS*) gene displaying EEAF. They carry homozygous missense variant c.285C>A (p. Asn95Lys), homozygous nonsense variant c.960C>A (p. Cys320Ter), and compound heterozygous missense variants c.416T>C (p. Met139Thr) and c.737G>A (p. Arg246His), respectively. Compared with wild‐type *MOS*, these *MOS* variants impaired ERK1/2 activation, resulting in oocyte meiotic resumption and cytoskeleton assembly defects. RNA‐sequencing results revealed that substantial maternal mRNAs’ clearance during oocyte maturation was retarded either in mature oocytes carrying MOS^Asn95Lys^ variant or in U0126‐treated oocytes, in which the most affected transcripts were enriched in mitochondrial biological process. Furthermore, inactivation of MOS‐ERK signal cascade caused mitochondrial dysfunction of mature oocytes. These results indicate MOS‐ERK1/2 signal pathway is vital for embryo development through driving oocyte cytoplasmic maturation in human and mouse.

**Clinical impact**

This study firstly provides direct evidence that biallelic *MOS* mutation is associated with EEAF phenotype and female infertility, which may serve a target gene for early diagnosis and therapeutic strategy development.

## Introduction

Early embryo fragmentation is defined as anucleate cell fragments derived from the blastomere, and it is a common feature of *in vitro* fertilization (IVF) or intracytoplasmic sperm injection (ICSI) cycles. Embryos with more than 25% fragmentation are closely associated with deleterious outcomes, including decreased blastocyst formation and implantation rate, and increased malformation rate after pregnancy (Ebner *et al*, 2001). Several hypotheses for pathogenesis of embryo fragmentation concerns on apoptosis, telomere length, cytoskeleton abnormality, increasing maternal age, and DNA fragment in sperm, with no definitive conclusion having been drawn (Fujimoto *et al*, 2011). Recurrent early embryonic arrest with fragmentation (EEAF) is a severe type of embryo developmental failure that results in no embryo transfer, which performed as a compound phenotype of cleavage arrest and embryo fragmentation. Despite increasing studies support that EEAF may be of maternal origin defects, however, the causative gene and the underlying mechanism remain largely unknown.

In recent years, variants in single maternal‐effect gene, including *TUBB8* (MIM: 616768), *PADI6* (MIM: 610363), *TLE6* (MIM: 612399), *KHDC3L* (MIM: 611687), *NLRP2* (MIM: 609364), and *NLRP5* (MIM: 609658) (Alazami *et al*, 2015; Feng *et al*, 2016; Xu *et al*, 2016; Mu *et al*, 2019; Zhang *et al*, 2019b; Zheng *et al*, 2020a), have been found to be responsible for human recurrent embryonic arrest in IVF/ICSI attempts. Additionally, we previously indicated variants in human *BTG4* (MIM: 605673), causing large‐scale maternal‐effect gene decay defect, resulted in zygotic cleavage failure (Zheng *et al*, 2020b). However, none of these genes can explain the EEAF mechanistically.

The Moloney sarcoma oncogene (*Mos*) gene encodes a serine/threonine protein kinase, which was first identified as a cytostatic factor to maintain oocyte metaphase II (MII) arrest via activation of the ERK pathway by phosphorylating MEK (Sagata *et al*, 1989; Verlhac *et al*, 2000). MOS‐ERK signal pathway‐mediated MII arrest relied on EMI2‐mediated APC^CDC20^ inhibition to prevent cyclin B1 from degradation (Shoji *et al*, 2006; Suzuki *et al*, 2010; Sako *et al*, 2014). The *MOS* mRNA is highly expressed in oocyte, and MOS protein is actively translated during oocyte maturation and rapidly degraded after fertilization, in several vertebrates, including human (Sagata *et al*, 1988; Watanabe *et al*, 1991; Sha *et al*, 2020b). Despite that MOS protein is nearly untranslated in oocyte at GV stage, precocious activation MOS‐ERK signal cascade by microinjection of *Mos* mRNAs in mouse immature oocytes could promote cyclin B1 translation and maturation promoting factor (MPF) activation, leading to oocyte meiotic maturation resumption (Choi *et al*, 1996b; Cao *et al*, 2020). Deficiency of *Mos* or *Erk1/2* in mice results in oocyte MII arrest failure, spindle abnormality, large polar body, and early embryo developmental arrest (Colledge *et al*, 1994; Hashimoto *et al*, 1994; Araki *et al*, 1996; Choi *et al*, 1996a; Zhang *et al*, 2015). Until now, *MOS* (OMIM ID: 190060; NM_005372.1) has not been found to be associated with any human disease definitely.

In this study, we identified four pathogenic variants in *MOS* that are responsible for human recurrent EEAF. Both affected individuals had biallelic variants that followed a Mendelian recessive inheritance pattern. We verified the pathogenic effects of *MOS* variants on protein expression and ERK1/2 activation. Inactivation of MOS‐ERK signal pathway in oocyte caused abnormal F‐actin assembly disorder, maternal‐effect genes decay defect, and mitochondrial dysfunction, resulting in EEAF. Our findings established the causal relationship between *MOS* and the phenotype of EEAF in human, which may provide targets for therapeutic strategies in future.

## Results

### Clinical characteristics of the affected individuals

**Figure 1:**
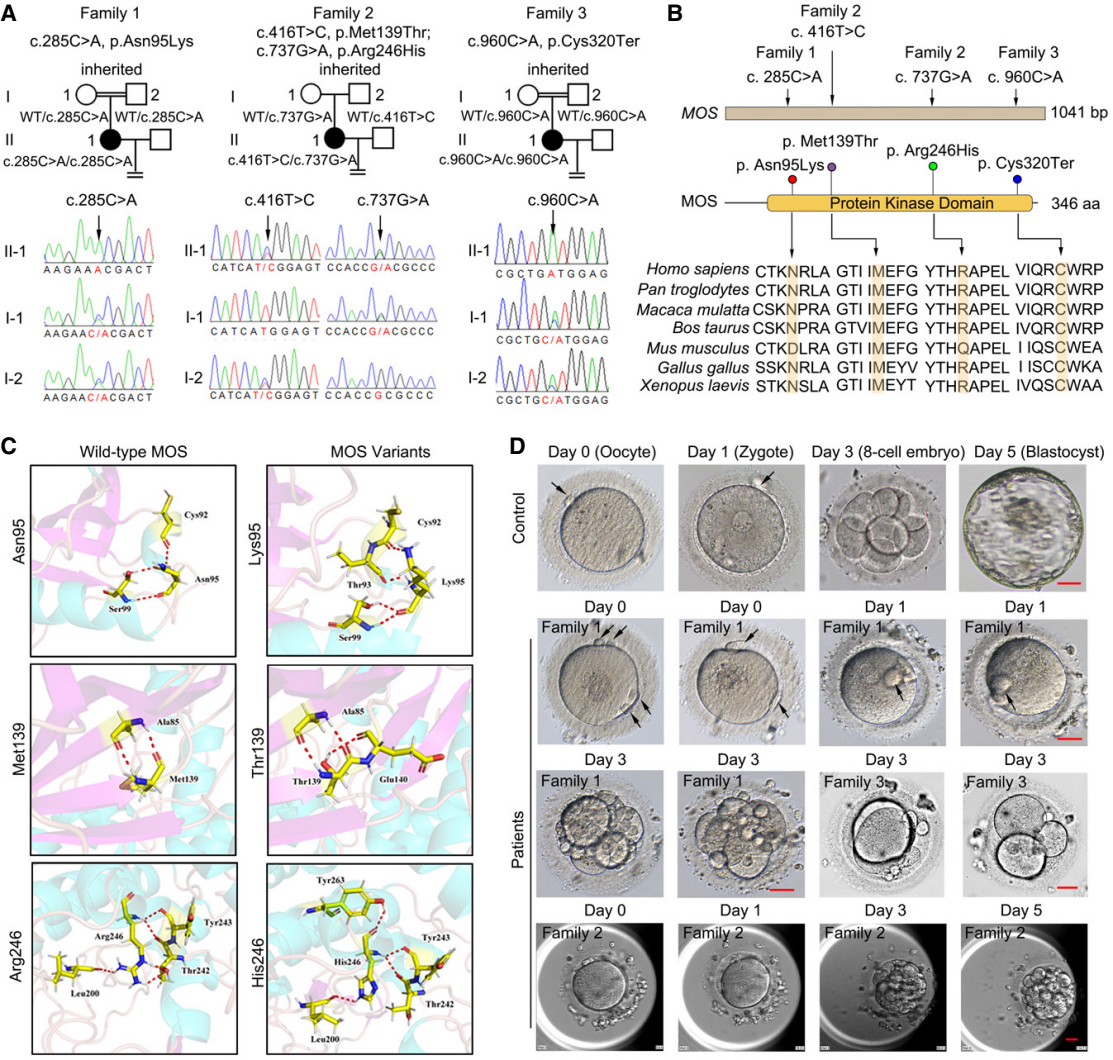
Identification of *MOS* variants in female patients with early embryonic arrest and fragmentation Three pedigrees and four *MOS* variants were identified, including homozygous missense variant Asn95Lys in patient 1 (family 1), compound heterozygous missense variants Met139Thr and Arg246His in patient 2 (family 2), and homozygous nonsense variant Cys320Ter in patient 3 (family 3). The four *MOS* variants were inherited and identified via Sanger sequencing. WT indicates wild‐type.Distribution of *MOS* variants in genome and the corresponding amino acid sequences. Multiple sequence alignment of four segments of MOS and the orthologs, with mutated residue marked in yellow.
*MOS* variants encoding amino acid disrupted the ion pairs formed by wild‐type MOS protein.The morphology of oocytes and embryos from female patients with *MOS* variants. The day of oocyte retrieval was defined as day 0. Polar bodies were indicated by black arrows. Scale bar = 20 µm. Source data are available online for this figure.

We recruited three independent female probands with infertility of unknown causes. All affected individuals belong to Chinese Han population and are from three families without potential fertility problems in China. They had normal menstrual cycles and sex hormone levels (Appendix Table S1). Proband II‐1 from a consanguineous family (family 1) had undergone one IVF and two ICSI failed attempts. All 15 oocytes retrieved in the three attempts were mature. However, half of the retrieved oocytes displayed multiple polar bodies or enriched granules in the cytoplasm, with a low normal fertilization (2PN) rate (20%, 25%, and 50%, respectively), and nine zygotes were cleaved at the two‐ to seven‐cell stage on day 3 and arrested during subsequent blastocyst culture. Three of nine arrested embryos were accompanied by severe fragmentation at the cleavage stage (Table 1 and Fig 1D). Proband II‐1 in family 2 had undergone four separate ICSI attempts, and three previous failed attempts in other hospitals were diagnosed as embryonic arrest (arrested at the two‐ to six‐cell stage, with embryo fragmentation). In her fourth ICSI attempt, we used time‐lapse to observe embryonic development. Overall, 11 of the 14 oocytes retrieved were mature, and the couple chose to freeze four MII oocytes in this attempt. Then, three 2PN, two 1PN, and two 3PN zygotes were formed; all zygotes consistently arrested at the two‐ to five‐cell stage, and six of seven arrested embryos with severe fragmentation were produced during the first cleavage (Table 1 and Fig 1D). Proband II‐1 from a consanguineous family (family 3) had undergone one IVF with ICSI rescue in other hospital and one ICSI attempt in our hospital. In her two attempts, partial immature oocytes were observed, including 2 metaphase I (MI) oocytes of 12 retrieved oocytes and 2 MI oocytes of 5 retrieved oocytes. In her first attempt, four zygotes were derived yet only one developed to five‐cell stage accompanied by severe fragmentation and other three were arrested at one‐cell stage. In her second attempt, all three 2PN zygotes arrested at two‐ and three‐cell stage (Table 1 and Fig 1D).

**Table 1:** Oocyte and embryo characteristics of IVF and ICSI attempts for the affected individuals.

Family NO.	Age (years)	Duration of infertility (years)	IVF/ICSI Attempt	Retrieved oocytes	Immature oocytes	MII oocytes	Oocyte with abnormal morphology	Fertilized outcomes (2PN + 1PN + MPN + 0PN)	Cleaved/Fragmented embryo	Embryo outcomes
1	40	12	IVF	5	0	5	multi‐polar body	1 + 0+1 + 3	3/0	2*3‐cell, 1*2‐cell, arrested
			ICSI	4	0	4	multi‐polar body, dark cytoplasm	1 + 0+0 + 3	3/2	1*6‐cell, 1*4‐cell, 1*2‐cell, arrested
			ICSI	6	0	6	multi‐polar body, dark cytoplasm	3 + 0+0 + 3	3/1	1*7‐cell, 1*4‐cell, 1*5‐cell, arrested
2	31	4	ICSI	9	0	9	N/A	NA	3/N/A	All arrested at 2‐ to 6‐cell^a^
			ICSI	13	0	13	N/A	NA	2/2	All arrested at 2‐ to 6‐cell^a^
			ICSI+AOA	16	0	16	4 degradations	4 + 5+1 + 2	6/N/A	All arrested at 2‐ to 6‐cell^a^
			ICSI	14	3	11 (4 frozen)	N/A	3 + 2+2 + 0	7/6	3*5‐cell, 4*2‐cell, arrested
3	34	7	IVF+Rescue ICSI	12	2	10	N/A	4	1/1	1*5‐cell, arrested ^a^
			ICSI	5	2	3	N/A	3 + 0+0 + 0	3/0	2*3‐cell, 1*2‐cell, arrested

Arrested, arrested during subsequent blastocyst culture; MII, Metaphase II; MPN, multi‐pronucleus; N/A, not available; PN, pronucleus.

^a^ The attempt at other hospitals.

### Identification of *MOS* pathogenic variants

Whole‐exome sequencing (WES) was used to identify pathogenic variants. After filtering using the criteria described in the materials and methods section, only biallelic variants of *MOS* (OMIM ID: 190060; NM_005372.1) were found in all affected individuals yet absent in our 100 controls undergoing natural conception, suggesting the possible genetic contribution of *MOS* to human EEAF. Patient 1 and patient 3 had a homozygous missense variant c. 285C>A (p. Asn95Lys) and nonsense variant c. 960C>A (p. Cys320Ter), respectively, both variants inherited from their parents as confirmed by Sanger sequencing (Fig 1A). Patient 2 carried two missense variants, c. 416T>C (p. Met139Thr) and c. 737G>A (p. Arg246His), inherited from her father and mother, respectively (Fig 1A). All three families followed a recessive inheritance pattern. The variant p. Asn95Lys had a low frequency of 0.0000163 in the gnomAD exome database and 0.0000329 in the ExAC database (Table 2). The other three variants have not been reported in those public databases (Table 2). All four residues were located in the protein kinase domain, comprising amino acids 60^th^ to 341^st^, and they are highly conserved in different species, from *Xenopus laevis* to *Homo sapiens* (Fig 1B). Based on the three‐dimensional (3D) structures of the MOS protein used to assess the effect of missense variants, the three variants had no obvious effects on the protein structure but resulted in some new hydrogen bonds produced, such as Thr93, Glu140, and Thr263, to the residues Lys95, Thr139, and His246, respectively (Fig 1C), which might have further effects on MOS protein property. In addition, the four variants were predicted pathogenic by SFIT or Polyphen with possibly or probably damaging and all had CADD scores > 20 (Table 2). The nonsense variant c. 960C>A results in premature termination at cysteine at 320^th^ amino residue, disrupting the integrity of MOS kinase domain. Collectively, these results indicated that *MOS* variants are likely to be pathogenic.

**Table 2:** Overview of the *MOS* variants observed in the families.

Probands in Families	Genomic Position on chr 8 (bp)	cDNA Change	Protein Change	Mutation Type	Genotype	gnomAD Allele frequency	ExAC Allele frequency	Polyphen Score	Polyphen	SIFT Score	SIFT	CADD_phread Score
Family 1	57026257	c.285C>A	p. Asn95Lys	missense	homozygous	1.63x10^‐5^	3.29x10^‐5^	0.843	P	0	D	28.5
Family 2	57026126	c.416T>C	p. Met139Thr	missense	compound heterozygous	N/A	N/A	0.995	D	0	D	27.2
	57025805	c.737G>A	p. Arg246His	missense		N/A	N/A	0.874	D	0.001	D	28.1
Family 3	57025582	c.960C>A	p. Cys320Ter	nonsense	homozygous	N/A	N/A	N/A	N/A	N/A	N/A	41.0

D, Deleterious, *MOS*, MOS proto‐oncogene, serine/threonine kinase; N/A, not available; P, Possibly damaging.

### Pathogenic effects of *MOS* variants in protein expression and ERK1/2 activation

**Figure 2:**
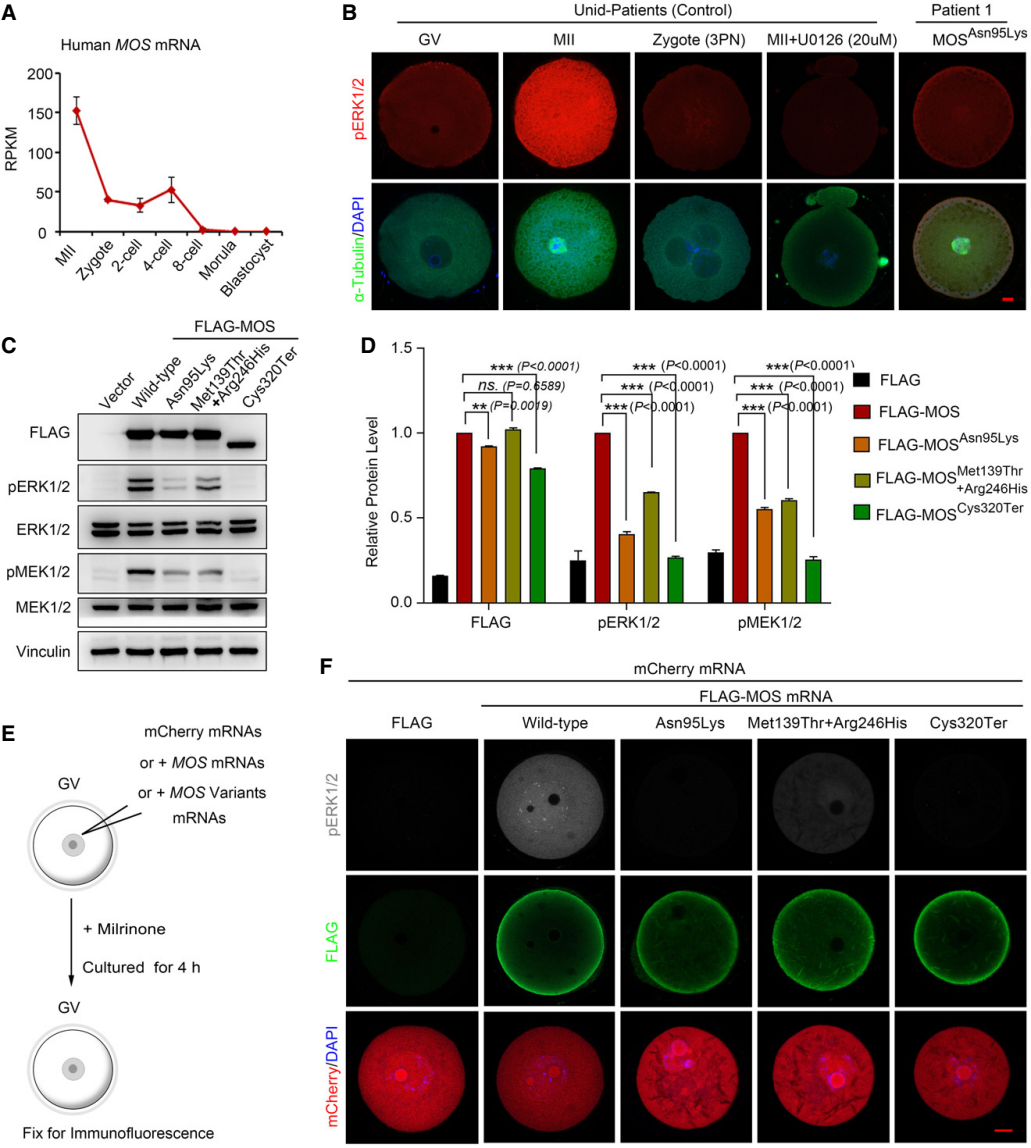
The effects of *MOS* variants on protein expression and ERK1/2 activation in cells and oocytes The per million mapped reads (RPKM) values (extracted from GSE36552) showing the *MOS* expression level in human oocytes and early embryos (*n* = over 3 biological replicates).The immunofluorescence images show the pERK1/2 (red) dynamics in immature (GV, *n* = 5), mature (MII, *n* = 10), and fertilized oocytes from unidentified control patients (*n* = 6), as well as in mature oocytes from patient 1 carrying homozygous MOS^Asn95Lys^ variants. pERK1/2 activation was inhibited after U0126 treatment (*n* = 6). FITC‐α‐tubulin (Green) and DAPI (blue) were used for co‐staining. Scale bar = 10 µm.Western blot analysis of the MEK1/2 and ERK1/2 activation after transfection of FLAG‐tagged wild‐type *MOS* and identified *MOS* variants in HEK 293 cells.Statistical analysis of the protein expression level of FLAG, pERK1/2, and pMEK1/2 (*n* = 2 technical replicates). The protein intensities were quantified by Image J software.Diagram showing the experimental design, including procedure of *MOS* mRNAs injection and oocyte culture. GV oocytes were overexpressed via microinjection with wild‐type *MOS* or *MOS* variant mRNAs combined with mCherry mRNAs, followed by culture for 4 h in medium containing 2.5 µM milrinone.Immunofluorescence of FLAG (green) and pERK1/2 (grey) in oocytes after injection of different *MOS* variant mRNAs combined with mCherry mRNAs. The signal of mCherry was directly captured after fixation, with DAPI staining for DNA visualization (blue). (*n* = over 30 oocytes in each group.) Scale bar = 10 µm. Data information: In A and D, data are presented as mean ± SD. **P < 0.01, ***P < 0.001; *ns*., no significance (two‐way ANOVA with Tukey’s multiple comparisons test in D). Detailed *P* value as indicated. Source data are available online for this figure.

Moloney sarcoma oncogene translation mediates MEK1/2 phosphorylation, leading to ERK1/2 activation during oocyte maturation, which has been demonstrated in various vertebrate oocytes (Sagata *et al*, 1988, 1989; Verlhac *et al*, 2000), except humans. Thus, we examined the expression of *MOS* in human oocytes. Through analysis of the reported RNA‐seq data of human oocytes and early embryos (Data ref: Yan *et al*, 2013), we found that *MOS* mRNA was highly expressed in oocytes, remarkably decreased after fertilization, and slightly increased in four‐cell stage embryos, followed by a gradual decrease from eight‐cell to blastocyst embryos (Fig 2A). Next, we examined ERK1/2 activation by staining for phosphorylated ERK1/2 (pERK1/2). The pERK1/2 signal was weak in human GV oocytes and zygotes, but was strong in MII oocytes. The staining signal is highly specific to pERK1/2, as it is undetectable in *in vitro* matured MII oocytes with U0126 (20 µM) treatment for 24 h (Fig 2B). Moreover, the pERK1/2 levels were significantly decreased in the unfertilized oocytes of patient 1 compared with those in the control oocytes from unidentified patients (Fig 2B). These results indicate that ERK1/2 is highly activated during human oocyte maturation and is suddenly inactivated after fertilization, and the *MOS* ^Asn95Lys^ variant affects ERK1/2 activation.

Next, we used HEK293 cells to assess the functional properties of these *MOS* variants. Transfection of FLAG‐labeled expression plasmids revealed that wild‐type *MOS* remarkably enhanced pERK1/2 levels, whereas overexpression of the *MOS* variants could not effectively activate pMEK1/2 and pERK1/2 in HEK293 cells (Fig 2C and D). In addition, MOS^Asn95Lys^ and MOS^Cys320Ter^ variants encoded MOS protein with slight decrease (Fig 2C and D). The four *MOS* variants exhibited similar subcellular localization but could not activate pERK1/2 in HEK293 cells except for MOS^Arg246His^, whose overexpression just caused a decrease in pERK1/2 level (Appendix Fig S1A). Since MOS directly interacts and phosphorylates MEK1/2 to activate ERK1/2, the binding capacity of these four *MOS* variants with MEK1 was assessed by co‐immunoprecipitation. We found MOS^Cys320Ter^ variant hardly interacted with MEK1, and both MOS^Asn95Lys^ and MOS^Arg246His^ variants interacted with MEK1 weaker than that of wild‐type MOS (Appendix Fig S1B and C). Therefore, we concluded that *MOS* variants mainly lead to MEK/ERK inactivation through the decreased MOS protein or weaker MEK1 interaction.

Since *MOS* is an oocyte‐specific gene, we overexpressed wild‐type *MOS* or four *MOS* variants into mouse GV oocytes to determine their expression. The *MOS* variants plasmids were transcribed into mRNA, and then, the same amounts of mRNAs were microinjected into GV oocytes and maintained in M2 medium with 2.5 µM milrinone for 4 h to assure the mRNA translation. The milrinone is a PDE3 inhibitor, which is used to prevent oocyte from meiotic resumption by maintaining elevated cAMP levels in oocytes. The mCherry mRNAs were co‐injected with *MOS* variants mRNAs to indicate successful translation. After the mCherry signal was observed by fluorescent microscope at 4 h after microinjection, the GV oocytes were collected for immunofluorescent staining to detect pERK1/2 and FLAG level (Fig 2E). We found that wild‐type *MOS* mRNA group activated much higher pERK1/2 than all four *MOS* variants in mCherry‐positive oocytes (Fig 2F).

**Figure EV1:**
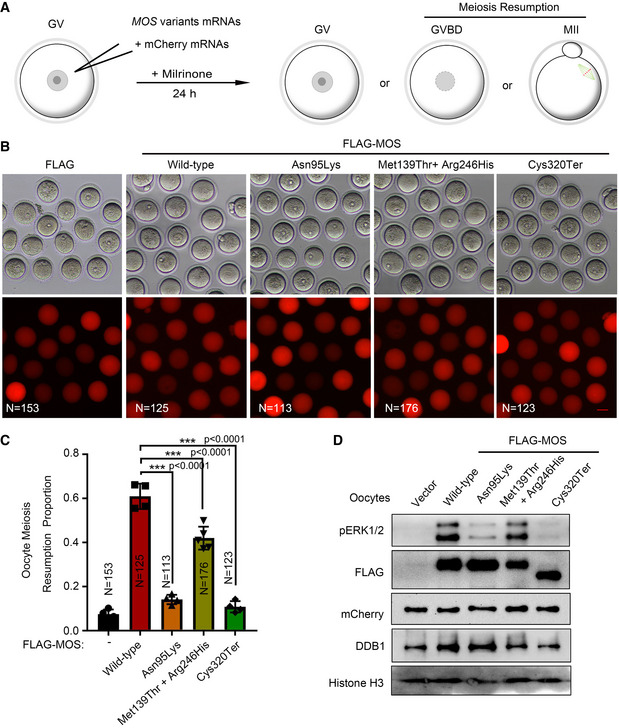
The effect of *MOS* variants on promoting oocyte meiosis maturation The diagram showing the experimental procedure. Geminal vesical (GV) oocytes were microinjected with FLAG‐tagged *MOS*, *MOS* ^Asn95Lys^, *MOS* ^Met139Thr^ and *MOS* ^Arg246His^, *MOS* ^Cys320Ter^, or FLAG mRNAs combined with mCherry mRNAs, and maintained in milrinone‐treated M2 medium for 24 h. The number of GV oocytes, geminal vesical breakdown (GVBD), or metaphase II (MII) oocytes was counted.The representative bright‐field images and fluorescent images were captured at 24 h after microinjection using a Nikon microscope. Over 100 oocytes in each group. Scale bar = 100 µm.The percentage of meiosis resumption (GVBD and MII) oocytes at 24 h after microinjection with different *MOS* mRNAs in (B). N number was labeled in each graph.Western blot analysis of FLAG, pERK1/2, and mCherry using oocytes after microinjection with different *MOS* mRNAs combined with mCherry mRNAs for 24 h. Histone H3 and DDB1 were used as loading controls. *N* = 100 oocytes per sample. Data information: For C, data are expressed as mean ± SD. One‐way ANOVA followed by *post hoc* Tukey’s test for multiple comparisons, ***P < 0.0001. Source data are available online for this figure.

Despite MOS protein’s translation after GVBD, previous studies have revealed that precocious *MOS* translation in GV oocytes facilitates meiotic maturation (Cao *et al*, 2020). To test whether these *MOS* variants altered this function, we microinjected mCherry and *MOS* variants mRNAs (500 ng/ul for the homozygous variants, and each 250 ng/µl with mixed total 500 ng/µl for the compound heterozygous variants) in GV oocytes and maintained in milrinone‐treated medium for 24 h, and then counted the numbers of GV, GVBD, and MII oocytes (Fig EV1A). Although oocytes were cultured in the presence of milrinone, meiosis resumption (GVBD or MII stage) occurs in 7.5% oocytes in the vector group. In contrast, approximately 60% of oocytes resumed meiosis maturation in the wild‐type *MOS* group, which was significantly higher than those in the other three groups with *MOS* variants (50% reduction with MOS^Cys320Ter^ or MOS^Asn95Lys^ and only 20% reduction with the combined MOS^Met139Thr^ and MOS^Arg246His^) (Fig EV1B and C). By western blot analysis using these oocytes, we found pERK1/2 level deceased with varying degrees in three *MOS* variants group than wild‐type *MOS* group, as the MOS^Cys320Ter^ variant almost completely inactivated pERK1/2, however, combined MOS^Met139Thr^ and MOS^Arg246His^ variants just had slight decrease (Fig EV1D).

**Figure EV2:**
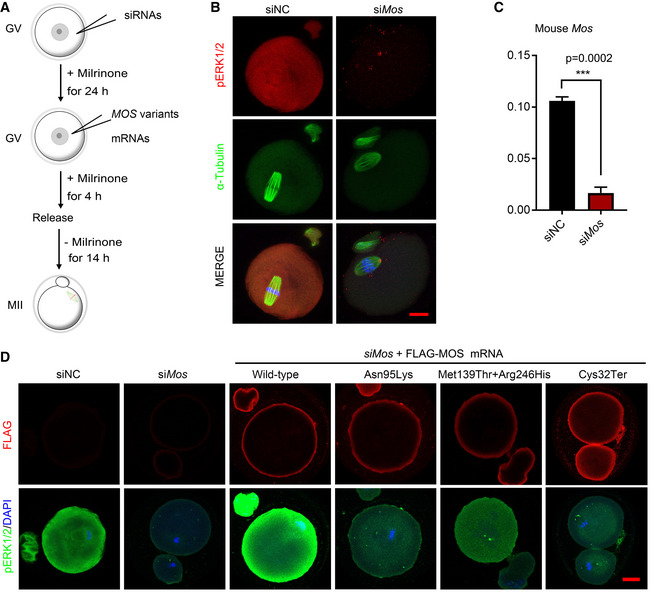
The RNAi effect of *Mos* and the rescuing effects of different *MOS* variants in oocytes Schematic diagram of oocyte cultures and experimental procedure. Mouse GV oocytes were microinjected with siRNAs and cultured for 24 h in the presence of 2.5 µM milrinone, followed by microinjection of *MOS* variants mRNAs, and cultured for 4 h before release to fresh M2 medium for another 14 h culture. Then, the RNAi efficiency of *siRNA* targeting mouse *Mos* and rescuing effects of different human *MOS* variants were determined.The representative images showing the decreased pERK1/2 (red) and abnormal spindle (FITC‐α‐tubulin, green) after *Mos* siRNA injection. DAPI (blue) was stained for visualization of DNA. Scale bar = 10 µm.RT‐qPCR results showing the mouse *Mos* mRNA expression levels in oocytes with negative control siRNAs or *Mos* siRNAs (*n* = 3).Representative immunofluorescence images showing the FLAG (red) and pERK1/2 (green) levels in mature oocytes after microinjection of *siMos* or combined with indicated human *MOS* variants in GV oocytes. At least 30 oocytes each group were used for immunofluorescence. Scale bar = 10 µm. Data information: In C, data are expressed as mean ± SD of three biological replicates. ***P = 0.0002 (unpaired two‐tailed Student’s *t*‐test). Source data are available online for this figure.

We further used another method to verify the pathogenic effects of *MOS* variants in oocytes. We injected negative control or mouse *Mos* siRNAs for 24 h, and then complemented with different *MOS* variants mRNAs into mouse GV oocytes in a medium with milrinone, 4 h later, followed by *in vitro* maturation (Fig EV2A). We found that pERK1/2 was completely undetectable in the *siMos* group with high knockdown efficiency (Fig EV2B and C), and complement of wild‐type human *MOS* mRNA reversed the pERK1/2 level (Fig EV2D). However, pERK1/2 levels were much lower in other three groups supplemented with *MOS* ^Asn95Lys^, *MOS* ^Cys320Ter^, or *MOS* ^Met139Thr^ combined with *MOS* ^Arg246His^ mRNAs than that in the wild‐type *MOS* mRNAs after depletion of inner *Mos* in mouse oocytes (Fig EV2D).

### Oocyte MOS‐ERK pathway is required for cytoskeleton assembly and prevents embryo from fragmentation

**Figure 3:**
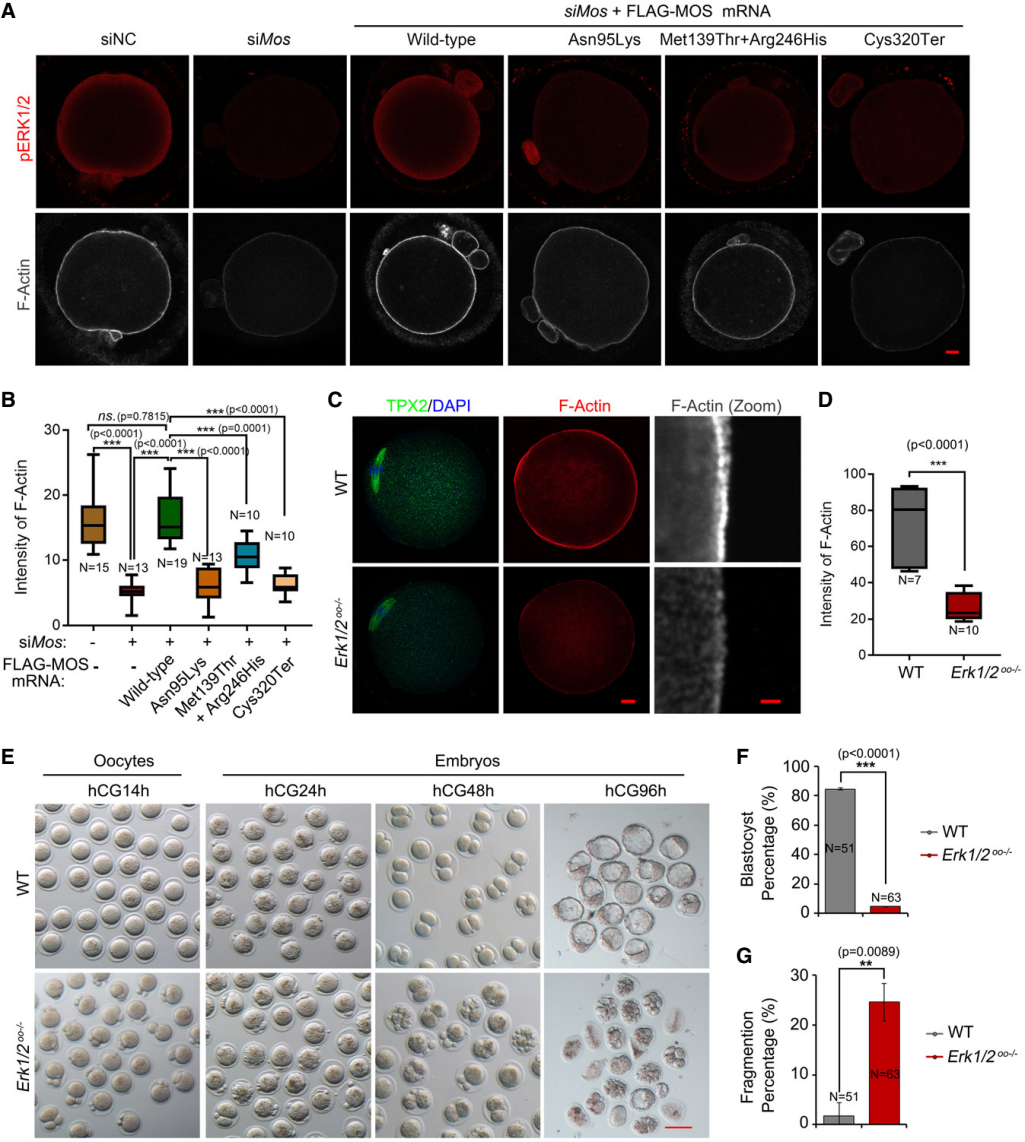
Oocyte MOS‐ERK1/2 pathway inactivation perturbs cortical F‐actin assembly and causes early embryonic arrest and fragmentation AImmunofluorescence of FLAG (red) and F‐actin (gray) in oocytes after microinjection of negative control or mouse *Mos* siRNAs combined with or without wild‐type human *MOS* or *MOS* variants mRNAs and culture in medium with 2.5 µM milrinone for 24 h, followed by release to maturation (*n* = 15–20 oocytes each group). Scale bar = 10 µm.BThe relative intensities of F‐actin were measured and compared among the indicated groups. The oocyte numbers for analyzed were labeled in each group. The box plot showing the F‐actin intensities distribution in each group quantified using Image J (*n* = 10–19/group).CThe immunofluorescence of F‐actin (red) and TPX2 (green) in WT and *Erk1/2^oo^* ^−/−^ oocytes, using DAPI (blue) co‐staining (*n* = 15–20 oocytes for each group). The zoomed images of F‐actin are displayed in gray. Scale bar = 10 µm.DThe box plot summarizing the relative intensities of F‐actin between control and *ERK1/2^oo^* ^−/−^ oocytes (*n* = 7–10 oocytes for each group).ERepresentative bright‐field images showing the morphology of oocytes and embryos from wild‐type and *ERK1/2^oo^* ^−/−^ mice. The MII oocyte, zygote, two‐cell, and blastocyst embryos were harvested *in vivo* at 14, 24, 48, and 96 h post‐hCG, respectively (*n* = 3 mice for each time points in two groups). Scale bar = 100 µm.F, GBar graphs shows the blastocyst percentage (F) and fragment percentage (G) in wild‐type (*n* = 51) and maternal ERK1/2 deletion embryos (*n* = 63). Data information: For B and D, the line in the box plot represents the median value of F‐actin intensity. Main box represents the values from the lower to upper quartile (25^th^ to 75^th^ percentile). The lower and upper whiskers indicate the min to max values. For (F) and (G), data are represented as mean ± SD. One‐way ANOVA with Tukey’s multiple comparisons test (B) and unpaired two‐tailed Student’s *t*‐test (D, F, and G) were used for statistical analysis. **P < 0.01, ***P < 0.001; *ns*., no significance. Source data are available online for this figure.

One of the most obvious phenotypes caused by *MOS* variants herein is embryonic arrest with sever fragmentation. Since the MOS‐ERK pathway is involved in the regulation of spindle assembly and cortical F‐actin thickness in mouse oocytes (Choi *et al*, 1996a; Chaigne *et al*, 2013), we investigated whether embryo fragmentation is derived from cytoskeleton assembly defects under the ERK1/2 inactivation. First, we evaluated the effects of *MOS* variants on the thickness of cortical F‐actin. We observed that F‐actin intensity in MII oocytes was associated with MOS and pERK1/2 levels, with much weakened F‐actin signal in *Mos*‐deficient oocytes; however, only wild‐type *MOS* supplement could reverse the *siMos*‐mediated F‐actin intensity (Fig 3A and B). We used U0126, a MEK1/2 inhibitor, to inactivate ERK1/2 during oocyte maturation and found decreased cortical F‐actin in both human and mouse oocytes (Appendix Fig S2A–C). We generated *ERK1* ^−/−^ *;ERK2^f/f^; Gdf9‐Cre* (*Erk1/2^oo^* ^−/−^) mice to specifically delete ERK1/2 in oocytes. As expected, ERK1/2 deficiency in oocytes caused very low cortical F‐actin and TPX2 signals, in which the latter one is a marker of spindle assembly (Fig 3C and D). In addition, *Mos* knockdown or ERK1/2 inactivation caused α‐tubulin instability (Fig EV2B and Appendix Fig S2). These results indicated that the MOS‐mediated ERK1/2 signaling pathway is essential for oocyte cytoskeleton homeostasis.

We then examined the fragmentation percentage derived from maternal wild‐type or *Erk1/2^oo^* ^−/−^ oocytes. All super‐ovulated *Erk1/2^oo^* ^−/−^ oocytes were mature, but with large and multiple polar bodies (Fig 3E). After fertilization, approximately 85% of embryos reached blastocysts from the wild‐type group 96 h post‐hCG injection (Fig 3F) with rarely fragmentation (1.7%, Fig 3G). However, among the embryos derived from maternal *Erk1/2^oo^* ^−/−^ oocyte, only 4.7% of the embryos developed into blastocysts (Fig 3F), 25% embryos were severely fragmented, and the remaining embryos are arrested or degraded (Fig 3E and G).

### Human MOS/ERK cascade governs maternal mRNA decay during oocyte maturation

**Figure 4:**
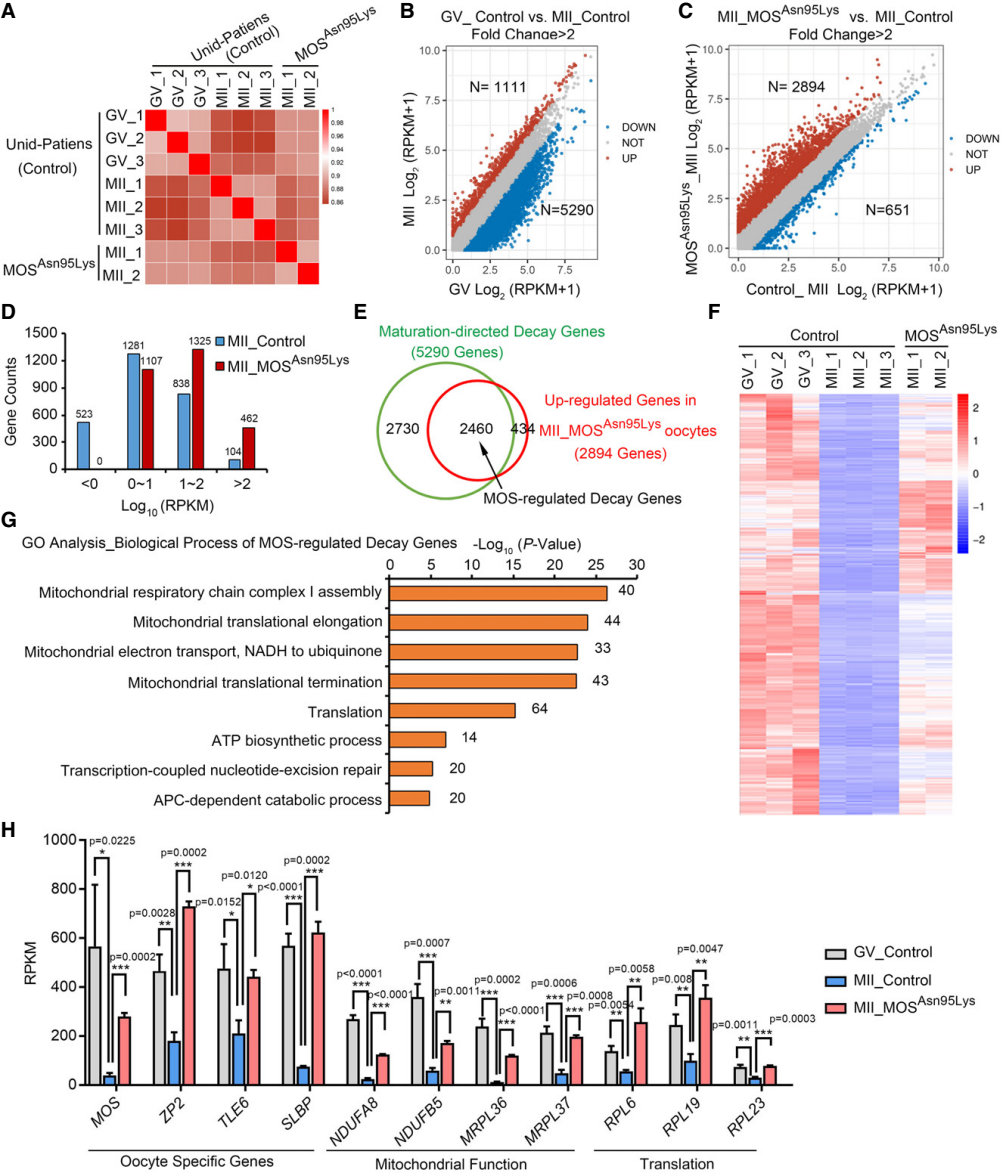
MOS deficiency retards maternal mRNA decay during human oocyte maturation AThe Pearson correlation analysis of RNA‐seq results of oocyte samples from unidentified control patients and *MOS* ^Asn95Lys^ patient 1.B, CScatter plots showing transcriptional changes between human GV and MII stage oocytes from unidentified control patients (B) and between control and *MOS* ^Asn95Lys^ MII oocytes (C), separately. Genes that were increased or decreased more than twofolds are indicated in red and blue, respectively. RPKM, reads per kilobase per million mapped reads.DUpregulated gene numbers based on different expression level in control and *MOS* ^Asn95Lys^ MII oocytes.EVenn diagram showing the MOS‐regulated decay genes, overlapped between the maturation‐directed decay genes (downregulated transcripts from GV to MII oocytes from control individuals) and the upregulated genes in *MOS* ^Asn95Lys^ oocytes.FHeatmap showing the expression dynamics of MOS‐regulated decay genes in GV and MII oocytes from control or *MOS* ^Asn95Lys^ patient.GThe enriched biological process of upregulated MOS‐regulated decay genes in *MOS* ^Asn95Lys^ oocytes, obtained via Gene Ontology (GO) analysis. The transcripts numbers of each biological process were labeled on the right.HThe bar graphs show the expression levels of selected MOS‐regulated decay genes (*n* = 2 or 3 biological replicates). Data information: In H, the RPKM values from RNA‐seq results are represented as mean ± SD. Unpaired two‐tailed Student’s *t*‐test was used for analysis. *P < 0.05, **P < 0.01, ***P < 0.001. Detailed *P*‐value as indicated. Source data are available online for this figure.

Our previous studies demonstrated that ERK1/2 regulates the maternal mRNA translation and decay in mouse oocytes from the GV to MII stage (Sha *et al*, 2017; Jiang *et al*, 2021), and defects in maternal mRNA degradation is associate with early embryonic arrest (Sha *et al*, 2020a, 2020b). However, whether MOS is required for maternal mRNA decay in human oocytes is unclear. Thus, we performed RNA sequencing (RNA‐seq) using GV and MII oocytes from unidentified control individuals and MII oocytes from the patient 1 carrying homozygous *MOS* ^Asn95Lys^ variant. Using Spearman correlation analysis, the mRNA profiles of replicates in the groups were determined to be comparable (Fig 4A). From GV to MII oocytes, 5,290 gene transcripts were downregulated and 1,111 transcripts were upregulated with a threshold of twofold, indicating that substantial mRNAs were robustly removed by mRNA decay during human oocyte maturation (Fig 4B). These 5,290 genes in this study were referred to as “maturation‐directed decay genes”.However, the mRNA expression profile of *MOS* ^Asn95Lys^ MII oocytes was very different from that of the three control MII oocytes, but it was more similar to that of GV stage oocytes (Fig 4A). By comparing the log‐transformed FPKM+1 of *MOS* ^Asn95Lys^ oocytes with those in the control oocytes, 2,894 and 813 genes were identified as upregulated and downregulated more than twofold, respectively (Fig 4C). Additionally, more transcripts with high abundance were observed in the 2,894 upregulated genes in *MOS* ^Asn95Lys^ oocytes compared with the control MII oocytes (Fig 4D). Among the 2,894 upregulated transcripts in *MOS* ^Asn95Lys^ oocytes, 2,460 genes belong to the maturation‐directed decay genes from human GV to MII oocytes (Fig 4 E and F and Dataset EV1), which were named “MOS‐regulated decay genes”. The Gene Oncology (GO) analysis results revealed that these MOS‐regulated decay genes were mainly enriched in mitochondrial function, mitochondrial translation, and translation (Fig 4G). We selected several MOS‐regulated decay genes, including genes responsible for mitochondrial function (*NDUFA8*, *NDUFB5*, *MRPL36*, and *MRPL37*) and translation (*RPL6*, *RPL19*, and *RPL23*), as well as maternal genes (*MOS*, *ZP2*, *TLE6*, and *SLBP*) that were highly expressed in oocytes, as shown in Fig 4H. Based on these results, we conclude that MOS governs maternal mRNA decay during human oocyte maturation.

**Figure 5:**
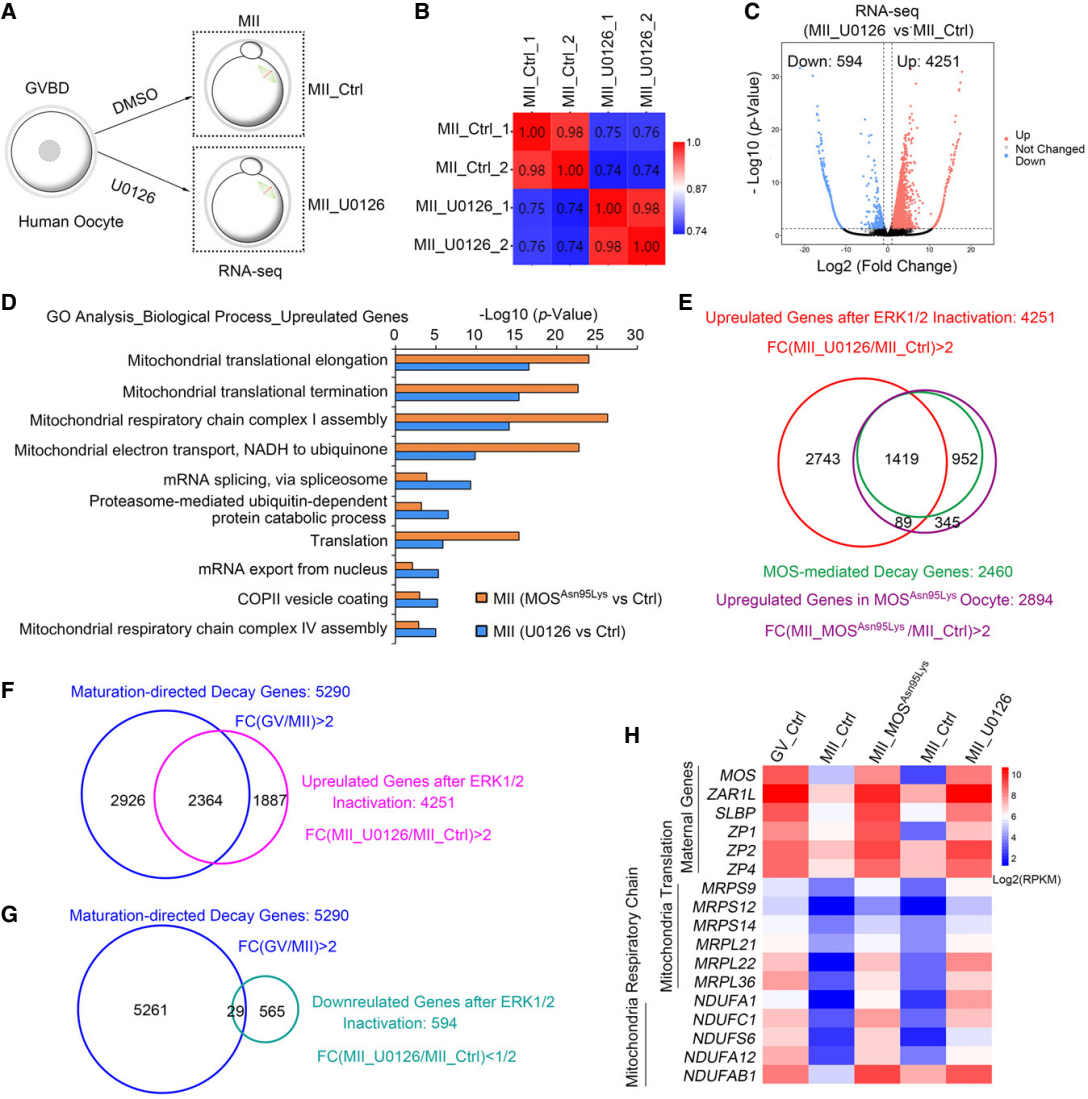
ERK1/2 inactivation disrupts maternal mRNA clearance during human oocyte maturation ASchematic presentation of human oocyte sample collection for RNA‐seq. Human immature oocytes (GVBD stage) from unidentified control women were *in vitro* matured with DMSO or U0126 treatment for 24 h, followed by collection of MII oocytes for RNA‐seq. Ctrl means control.BThe Pearson correlation analysis of RNA‐seq results of human oocyte samples from control and U0126 group (*n* = 2 samples for each group). Scale bar indicates the range of the correlation coefficients (*r*) displayed.CVolcano plot of RNA‐seq data obtained from human oocytes in control and U0126 group. “Up” and “Down” are the upregulated and downregulated transcripts in oocytes treated with U0126. The genes with upregulation and downregulation (abs[log_2_FC] ≥ 1; FDR ≤ 0.05) were labeled in red and blue, respectively.DGene Ontology (GO) analysis of upregulated genes from RNA‐seq data of U0126 group and MOS^Asn95Lys^ MII oocytes compared with each control oocytes.EVenn diagram showing the upregulated genes in oocytes after U0126 treatment versus MOS‐regulated decay genes (green) or upregulated genes in MOS^Asn95Lys^ oocytes (purple). FC, fold change.F, GThe overlapped genes between the maturation‐directed decay genes and the upregulated (F) or downregulated (G) genes in U0126‐treated MII oocytes. FC, fold change.HHeatmap showing the expression level of selected maternal genes, mitochondrial translation genes, and mitochondrial respiratory chain genes regulated by *MOS* mutation or ERK1/2 inactivation. Source data are available online for this figure.

To further determine the role of human MOS on regulating maternal mRNA clearance through ERK1/2 signal cascade, we performed RNA‐seq using *in vitro* matured human oocytes with U0126 treatment, a MEK1/2 inhibitor, to inhibit ERK1/2 activation. Samples from control and U0126 group were analyzed in duplicate, and the two independent replicates exhibited high correlations (Fig 5A and B). Gene expression levels were assessed as reads per kilobase of transcript per million mapped reads (RPKM) with ERCC as a spike‐in. As expected, substantial transcripts were upregulated compared with the control MII oocytes. Of the approximately 18,000 genes detected in RNA‐seq data of oocytes, 4,251 and 594 genes were upregulated and downregulated, respectively, by a twofold change threshold (Fig 5C and Dataset EV2). The gene ontology (GO) analysis revealed that the upregulated genes were relative to mitochondrial translation and mitochondrial respiratory chain complex assembly in oocytes either with MOS^Asn95Lys^ variant or U0126 inhibition (Fig 5D). Over half of the upregulated transcripts in MII oocytes with MOS^Asn95Lys^ variant (1,597 in 2,894) overlapped with the upregulated genes in oocytes with U0126 treatment (Fig 5E). Consistently, about 60% transcripts in MOS‐directed decay genes (1,505/2,460) belong to the transcripts whose level increased in U0126‐treated oocytes (Fig 5E). Among the 4,845 differential expressed genes (with 4,251 upregulation and 594 downregulation) in U0126‐treated oocytes, 2,364 genes with upregulation and 29 with downregulation belong to maturation‐directed decay genes (5,290, Fig 5F and G). Many genes that should be degraded during oocyte maturation were significantly upregulated both in MOS^Asn95Lys^ oocytes and U0126‐treated oocytes, including maternal genes with high expression in oocyte (*MOS*, *ZAR1L*, *ZP1*, *ZP2*, *ZP4,* and *SLBP*), genes relative to mitochondrial translation (*MRPSs* and *MRPLs*) and mitochondrial respiratory chain complex assembly (*NDUFs*) (Fig 5H and Appendix Fig S3A and B). Therefore, *MOS* mutation or ERK1/2 inhibition mainly prevented the decrease in transcripts that should be removed from human GV to MII oocytes. Together, these results suggest MOS‐ERK signal cascade governs maternal mRNA clearance during human oocyte maturation.

### MOS/ERK cascade maintains oocyte mitochondrial function through facilitating mitochondrial mRNA clearance

**Figure 6:**
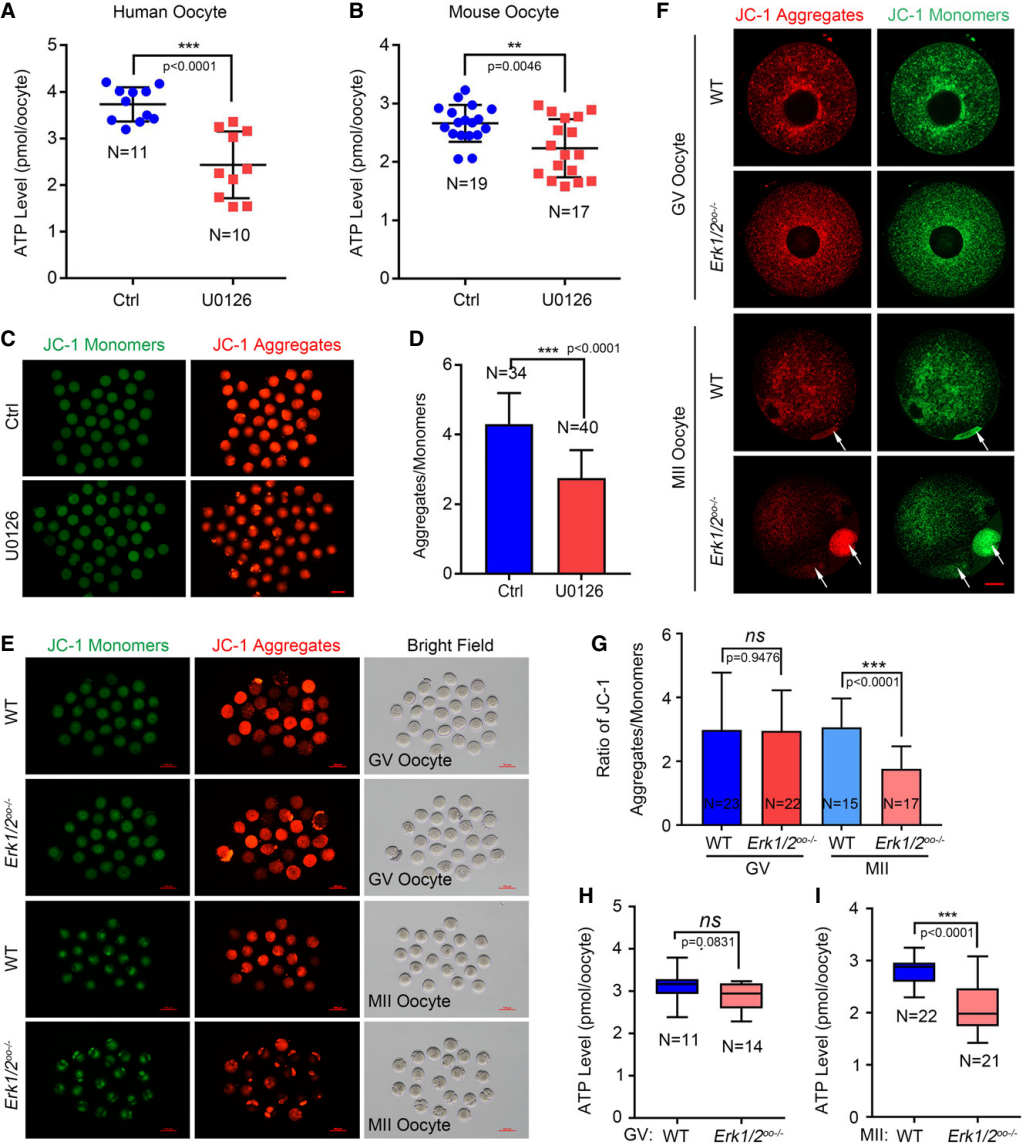
ERK1/2 inactivation causes oocyte mitochondrial dysfunction during meiotic maturation AThe scatter plots showing the ATP levels of human MII oocyte matured in medium with DMSO (control, Ctrl, *n* = 11) or 20 µM U0126 treatment (*n* = 12).BThe scatter plots showing the ATP levels of mouse MII oocytes after *in vitro* maturation with DMSO (control, Ctrl, *n* = 19) or 20 µM U0126 (*n* = 17).CThe representative images showing the JC‐1 monomers (green) and aggregates (red) of mouse oocytes after treatment with DMSO (control, Ctrl, *n* = 34) or U0126 (*n* = 40) in *in vitro* culture medium for 16 h. Scale bar = 100 µm.DThe bar graph of ratio of intensities of JC‐1 aggregates to those of monomers in (C). The intensities of JC‐1 were quantified by Image J software. The oocytes number used was labeled.ERepresentative images displaying JC‐1 monomers (green), aggregates (red), and bright filed images of GV and MII oocytes from wild‐type (WT) and *Erk1/2^oo^* ^−/−^ oocytes (*n* = 3 mice in each group). *Erk1/2^oo^* ^−/−^ oocytes exhibited big and multiple polar bodies. Scale bar = 100 µm.FConfocal images of JC‐1 monomers (green) and aggregates (red) of GV and MII oocytes from wild‐type (WT) and *Erk1/2^oo^* ^−/−^ oocytes (*n* = 3 mice in each group). *Erk1/2^oo^* ^−/−^ oocytes exhibited big and multiple polar bodies, indicated by red arrows. Hoechst 33342 (blue) was co‐stained to represent nucleus or chromosome. Scale bar = 10 µm.GThe graph showing the ratio of intensities of JC‐1 aggregates to those of monomers in (E).H, IBox plot showing the ATP contents of mouse GV oocytes (H) or MII oocytes (I) from wild‐type (WT) and *Erk1/2^oo^* ^−/−^ oocytes (N number was labeled in each graph). Data information: For A, B, D, and G, data represent mean ± SD. For H and I, the central line in the box indicates the median value of ATP level. The lower and upper limits of main boxes represent the first and third quartiles, respectively. The lower and upper whiskers represent the limits of extreme measurements. *ns*., no significance, **P < 0.01, ***P < 0.0001 (unpaired two‐tailed Student’s *t*‐test). Source data are available online for this figure.

As a vital organelle for energy metabolism, mitochondria become gradually activated during oocyte maturation by increasing mtDNA copy number, changing structure, and subcellular distribution to coordinate with oocyte meiosis division (Richani *et al*, 2021; Trebichalska *et al*, 2021). Mitochondrial dysfunction causes poor oocyte quality and interferes with early embryonic development (Morimoto *et al*, 2020; Zhang *et al*, 2021). Since the common retarded transcripts are enriched in mitochondrial biological process in oocytes with *MOS* mutation or after U0126 treatment indicated by RNA‐seq results. As expected, compared with the control group, ATP levels were significantly decreased in both human and mouse MII oocytes after U0126 treatment (Fig 6A and B), and the mitochondrial membrane potential was also obviously decreased (Fig 6C and D). In addition, we observed that the mitochondrial distribution in U0126‐treated oocytes was extremely different from the MII oocytes of control group (Fig 6C). We also determined the mitochondrial distribution and membrane potential using *in vivo* matured oocytes from wild‐type and *Erk1/2^oo^* ^−/−^ mice through staining JC‐1, which exhibits potential‐dependent accumulation from monomer to aggregates in mitochondria. Mitochondria distribution was comparable in either wild‐type or *Erk1/2^oo^* ^−/−^ GV oocytes. However, JC‐1 aggregates in mitochondria were enriched in the polar bodies of *Erk1/2^oo^* ^−/−^ oocytes, which is distinctly different from the uniform distribution of JC‐1 aggregates of mitochondria in wild‐type MII ooplasm (Fig 6E and F). Compared with wild‐type oocytes, the mitochondrial membrane potential and ATP level were significantly decreased in MII oocytes rather than GV oocytes from *Erk1/2^oo^* ^−/−^ mice (Fig 6G–I). Together, these results indicate human MOS/ERK cascade is required for maintaining mitochondrial function through accelerating mitochondrial mRNA clearance during oocyte maturation.

## Discussion

Early embryonic arrest is a cause of female infertility and occurs frequently in IVF or ICSI cycles. Fragmentation is commonly accompanied with early embryonic arrest; however, the genetic determinants of fragmentation formation are still unclear. In recent years, several maternal‐effect genes have been identified to cause human early embryonic arrest: the products of *TUBB8* is vital for oocyte spindle assembly and meiosis progression (Feng *et al*, 2016); the products of *TLE6*, *PADI6*, *NLRP5*, *NLRP2*, and *KHDC3L* can form subcortical maternal complex in oocytes (Alazami *et al*, 2015; Xu *et al*, 2016; Mu *et al*, 2019; Zhang *et al*, 2019b); and the product of *BTG4* is responsible for maternal mRNA decay during oocyte maturation and fertilization (Alazami *et al*, 2015; Feng *et al*, 2016; Mu *et al*, 2019; Zhang *et al*, 2019b; Sha *et al*, 2020a; Zheng *et al*, 2020b). These present studies have not elucidated the origination of embryo fragmentation. In this study, we described three infertile patients characterized by EEAF, carrying *MOS* biallelic variants followed by a recessive inheritance pattern.

Moloney sarcoma oncogene was initially discovered as a cytostatic factor (CSF) in oocytes to maintain MII arrest, in 1989 (Sagata *et al*, 1989). In the last four decades, the physiological function of the MOS‐mediated ERK signaling pathway has been well characterized in studies on *Xenopus* and rodents. Oocytes from *Mos* or *Erk1/2* knockout mice show parthenogenetic activation or fragmentation with longer duration of culture (Colledge *et al*, 1994; Hashimoto *et al*, 1994), while displaying a high frequency of abnormal spindle assembly, large and multiple polar bodies, and an even asymmetric division, resulting in a low fertilization rate and few offspring numbers (Araki *et al*, 1996; Choi *et al*, 1996a, 1996b). Similar to other species, human *MOS* mRNA is abundantly transcribed and stored in GV oocytes, and then, it is translationally activated after GVBD (Prasad *et al*, 2008). Only one study demonstrates that microinjection of *MOS* siRNA prevents meiosis maturation in human oocytes (Hashiba *et al*, 2001). To date, little is known about the function of human MOS in oocyte maturation and early embryos. In this study, we observed that the affected female individuals have similar reproductive phenotype previously identified in *Mos* ^−/−^ mice. The retrieved oocytes extruded multiple polar bodies and large polar body after fertilization in proband from family 1 with homozygous *MOS* variant (c.285C>A, p. Asn95Lys) (Fig 1D). However, the oocytes are morphologically normal from other two individuals carrying *MOS* nonsense (c.960C>A, p. Cys320Ter, family 3) or compound missense variants (c.416T>C, p. Met139Thr and c.737G>A, p. Arg246His, family 2), we speculate the phenotype difference among these three individuals is probably due to the variant locations. Consistent with the two‐cell stage arrest from *Mos* ^−/−^ mice (Colledge *et al*, 1994), most of the embryos from these three affected individuals displayed EEAF with development not past seven‐cell stage (Table 1), all arrested before embryonic genome activation (EGA). Moreover, the differences in the EEAF phenotype among the three patients are correlated with these differences of MOS variants in ERK1/2 inhibition. The patient 3 carries a homozygous nonsense variant (c.960C>A, p. Cys320Ter) in *MOS* gene, which displayed the weakest interaction with MEK1 (Appendix Fig S1B and C) and resulted in lowest ERK1/2 activation (Figs 2C and EV1D). Consistently, the patient 3 exhibited the earliest embryonic arrest (two‐ to three‐cell stage) than other two patients (two‐ to seven‐cell stage). This study is the first to identify pathogenic *MOS* variants and provides direct evidence of the role of MOS in oocyte maturation and early embryo development in human.

In addition, we observed a minor inhibition of MEK1/2 and ERK1/2 activation when we microinjected the same amount of mRNAs of MOS^Met139Thr^ and MOS^Arg246His^ variants (each with 250 ng/µl) into mouse immature oocytes (Fig EV1C). Based on the results in Appendix Fig S1, the MOS^Met139Thr^ variant loss function on ERK1/2 activation, similar to the other two homozygous variants (MOS^Asn95Lys^ and MOS^Cys320Ter^). However, the MOS^Arg246His^ variant displayed reduced binding ability with MEK1, leading to decreased pERK1/2 level to some extent (Fig Appendix S1A–C). On the one hand, we neglected there existed allelic gene expression imbalance in human. We guess that MOS^Met139Thr^ variant is expressed more than that of MOS^Arg246His^ variant in oocytes of patient 2. This needs further investigation and validation due to lack of oocyte samples so far. On the other hand, 500 ng/µl mRNAs for microinjection is a commonly used concentration, which is sufficient and even overdosed. We performed a range of concentration of *MOS* mRNAs for microinjection and found *MOS* mRNAs from 125 ng/µl to 500 ng/µl had similar effects (50–60%) on promoting oocyte maturation, whereas the 50 ng/µl *MOS* mRNAs only promotes 35% oocyte to resume meiosis maturation (Appendix Fig S4A). This suggests that there exists a threshold of *MOS* mRNAs to activate ERK1/2 in oocyte. Then, a total of 125 ng/µl mRNAs with combined MOS^Met139Thr^ and MOS^Arg246His^ variants were microinjected into GV oocytes with milrinone, and only 15% oocytes resumed meiotic maturation (Appendix Fig S4B and C), significantly lower than that (40% maturation) after 500 ng/µl mRNAs microinjection (Fig EV1C). Thus, we supposed that the minor inhibition of ERK1/2 activation of combined MOS^Met139Thr^ and MOS^Arg246His^ variants might result from these two factors: the imbalanced allelic gene expression and overdose of microinjected mRNAs.

Fragmentation is often used as a morphological parameter to assess human embryo quality. However, the cause of embryo fragmentation remains unclear. Firstly, cytoskeleton disorders along with membrane instability have been proposed to involve in fragmentation as the importance of actin filaments–microtubules interplay in coordinating human oocytes spindle assembly (Kawahara *et al*, 2002; Fujimoto *et al*, 2011; Stigliani *et al*, 2013; Holubcova *et al*, 2015; Roeles & Tsiavaliaris, 2019). *Mos* ^−/−^ mouse oocytes displayed cytoskeleton assembly defects during meiotic maturation and severe fragmentation with long‐time culture (Choi *et al*, 1996a; Chaigne *et al*, 2013). In our study, human MOS functions as a driver for normal cytoskeleton assembly, and all the four *MOS* variants displayed pathogenic roles in cortical F‐actin assembly (Fig 3A and B). The oocytes from ERK1/2‐deficient mice exhibited cortical F‐actin and spindle assembly defects, leading to EEAF as patients (Fig 3C–G). We suggest that MOS/ERK pathway activation is required for cytoskeleton assembly and oocyte membrane stability maintenance. Mitochondria, the primary source of ATP generation, is another factor to be associated with embryo fragmentation, as more mitochondrial DNAs have been detected in the culture medium of embryos having higher fragmentation rates (Stigliani *et al*, 2013). Decreased mitochondrial membrane potential and increased reactive oxygen species are observed in fragmented embryos compared with normal embryos (Fujimoto *et al*, 2011). A recent study demonstrated mitochondrial transfer from induced pluripotent stem cells rescues developmental potential of *in vitro* fertilized embryos from aging female mouse, further validating the vital role of oocyte mitochondria in promoting early embryo development. Through our RNA‐seq analysis, we also found dysregulated mitochondrial function in *MOS* ^Asn95Lys^ oocytes or U0126‐treated oocytes, such as mitochondrial respiratory chain complex I assembly and mitochondrial translation elongation and termination (Fig 4G). We further found that MOS‐ERK signal cascade inactivation caused decreases in mitochondrial membrane potential and ATP content in MII oocytes (Fig 6). MOS‐ERK signal pathway inactivation in oocytes caused embryo fragmentation probably due to the dual roles of cytoskeleton disorder and mitochondrial dysregulation. Since each independent role of mitochondria or cytoskeleton on fragmentation is unclear, the molecular basis underlying oocyte‐caused embryo fragmentation requires further investigation.

Abundant maternal mRNAs are synthesized and stored during oocyte growth, and were translationally activated followed by degradation during maternal to zygotic transition (MZT) (Sha *et al*, 2019). We previously found that ERK1/2 facilitates CPEB1 phosphorylation and protein degradation, accelerating maternal mRNA translation and subsequent decay process during mouse oocyte maturation. When maternal mRNA decay is blocked by the deletion of relative genes, such as *Btg4*, MZT failure results in early embryonic arrest (Yu *et al*, 2016; Zhao *et al*, 2017, 2020). As MOS is a maternal‐effect gene that is transiently translated during oocyte maturation, and has been reported to regulate the translation of some mRNAs (de Moor & Richter, 1997), we suspected that MOS‐ERK signal cascade may participate in mRNA decay from the GV to MII stage in human oocyte. Using *in vivo* matured oocyte with *MOS* mutation and *in vitro* matured oocytes by blocking ERK1/2 activation, this study demonstrated MOS/ERK signal pathway facilitated maternal mRNA decay during human oocyte maturation, as substantial transcripts that should be degraded from GV to MII oocytes were abolished both in *MOS* ^Asn95Lys^ oocytes (Fig 4C–F) and U0126‐treated oocytes (Fig 5C–E). EGA in early embryos is dependent on maternal mRNA translation and followed by degradation (Chan *et al*, 2019; Sha *et al*, 2019). The abnormality of maternal mRNA removal is associated with human early embryonic arrest and EGA failure (Sha *et al*, 2020a). We infer that *MOS* variants‐caused early embryonic arrest is probably due to maternal mRNA decay disorder. Collectively, this study provides the first and direct evidence that the human MOS/ERK pathway governs maternal mRNA decay during human oocytes maturation.

ERK1/2 signal pathway is the best known for its role in controlling cell proliferation, differentiation, and cell survival and cell fate decision through connecting activated growth factor receptors to change gene expression (Cook *et al*, 2017). ERK1/2 pathway also promotes genes expression of pro‐survival BCL2 family proteins (such as BCL2 and MCL1) and represses or inhibits pro‐death proteins (such as BAD and BMF) by a variety mechanism, and thereby inhibits mitochondrial pathway of apoptosis (Boucher *et al*, 2000; She *et al*, 2002; VanBrocklin *et al*, 2009; Booy *et al*, 2011). Activated ERK1/2 also translocates to mitochondria and interacts with mitochondrial proteins. For example, ERK1/2 directly phosphorylates DRP1 and MFN1 to promote mitochondrial fission and fragmentation (Kashatus *et al*, 2015; Pyakurel *et al*, 2015). Moreover, a study reported that ERK1/2 activation‐mediated mitochondrial fission and fragmentation is required for reprogramming somatic cells to induced pluripotent stem cells (Prieto *et al*, 2016). Our study demonstrated that MOS‐ERK pathway facilitates genes degradation relative to mitochondria in oocyte, unveiling a new way of ERK1/2 signal pathway for regulating mitochondrial function. However, it needs further investigation that the underlying mechanism of MOS/ERK signal pathway regulates mitochondria‐relative transcripts’ stability.

In conclusion, we identified and verified the pathogenic *MOS* variants in infertile females with EEAF. Further experiments elucidated the critical role of the MOS‐ERK pathway in the regulation of human oocyte cytoplasmic maturation, including cytoskeleton assembly, maternal mRNA decay, and mitochondrial function. Inactivation of the MOS‐ERK signaling pathway in human and mouse oocytes results in embryo fragmentation. We believe that this study will provide a better understanding of human oocyte maturation, embryo development, and embryo fragment formation.

## Materials and Methods

### Clinical samples

We recruited 120 infertile individuals diagnosed with EEAF and controls undergoing natural conception from the Sir Run Run Shaw Hospital and Reproductive and Genetic Hospital of CITIC‐Xiangya. The EEAF patients were screened according to the following criteria: primary infertility; normal karyotype in both couples; male factors excluded; normal endocrine level in female; experienced recurrent failure of IVF/ICSI attempts suffering embryonic arrest; and more than 50% of the cleaved embryo represented severer embryo fragmentation that occurred in at least one attempt. All blood samples and immature oocytes were donated for investigation after informed consent was obtained. Studies of human subjects were approved by the Ethics Committee of Sir Run Run Shaw Hospital and the Ethics Committee of the Reproductive and Genetic Hospital of CITIC‐Xiangya. Informed consents were obtained from all human subjects and the experiments were performed according to the principles set out in the World Medical Association (WMA) Declaration of Helsinki and the Department of Health and Human Services Belmont Report.

### Whole‐exome sequencing (WES) and variant screening

Blood samples from patients were subjected to WES. The variant score and functional prediction were assessed using the SIFT, Polyphen, and CADD programs. Variants were filtered using the following criteria (Zheng *et al*, 2020b): (i) variations with minor allele frequencies < 1% in the genome aggregation database (gnomAD) and Exome Aggregation Consortium (ExAC) databases; (ii) exonic non‐synonymous or splice site variants or coding indels; (iii) biallelic variants in the proband; and (iv) mRNAs/proteins that were highly expressed or specifically expressed in oocytes.

### Sanger sequencing

Specific primers flanking the variants in the *MOS* gene were used for amplification by PCR, followed by analysis using an ABI 3100 DNA analyzer (Applied Biosystems, Foster City, CA, USA).

### Molecular modeling and evolutionary conservation analysis

The three‐dimensional structure of wild‐type MOS (NP_005363.1) was predicted using the Swiss Model web server. Molecular graphics and analysis were performed using the PyMol software. Evolutionary conservation analysis was performed using the UniProt software.

### Animals

Wild‐type C57BL/6 mice were purchased from the Shanghai SLAC Laboratory Animal Co., Ltd (Shanghai, China). *Erk1* ^−/−^ *; Erk2^f/f^; Gdf9‐Cre* mice were generated as previously described (Zhang *et al*, 2015). Female mice (7–8 weeks old) were used in all experiments. These mice were maintained under specific pathogen‐free (SPF) conditions on a 12 h light/12 h dark cycle at 21–23°C with free access to food and water. All animal experiments were performed in accordance with the guidelines of the Animal Committee of Zhejiang University.

### Plasmid construction and cell transfection

Full‐length human *MOS* cDNA (NM_005372.1) was obtained from the human Ultimate ORF clone library. After sequencing, the QuikChange II site‐directed mutagenesis kit (200524, Agilent Technologies, Santa Clara, CA, USA) was used to introduce four variants (c.285C>A, c.416T>C, c.737G>A, and c.960C>A). The primers for mutagenesis are listed in Appendix Table S2. FLAG‐labeled expression plasmids were constructed using LR clonase (11791019, Thermo Fisher Scientific, Waltham, MA, USA), according to the manufacturer’s instructions. The pcDNA3.1+‐mCherry plasmid was kindly provided by Prof. Heng‐Yu Fan. All plasmids were verified by sequencing prior to transfection.

Human HEK293 cells, obtained from American Tissue Culture and Collection (ATCC), were cultured in DMEM medium (Gibco, Waltham, MA, USA) supplemented with 10% fetal bovine serum (FBS, Gibco), penicillin (100 U/ml), and streptomycin (100 μg/ml) at 37°C in 5% CO_2_. When cells reached 60–70% confluence, the same amount of plasmids was transfected using Lipofectime2000 (11668500, Thermo Fisher Scientific) according to the manufacturer’s instructions. Approximately 24–48 h after transfection, cells were fixed for immunofluorescence, western blotting, or immunoprecipitation.

### Oocyte culture and embryo collection

The 7‐ to 8‐week‐old female mice were injected with 7.5 IU pregnant mare serum gonadotropin (PMSG, Ningbo San Sheng Biotech, Ningbo, China) and humanly killed 44–48 h later. GV oocytes were harvested by puncturing the large ovarian follicles in L15 medium containing 0.2% BSA, 100 U/ml penicillin, 100 μg/ml streptomycin, and 2.5 µM milrinone (HY14252, MCE, Neodesha, KS, USA) to prevent oocyte from undergoing germinal vesicle breakdown (GVBD). The GV oocytes were maintained in milrinone‐treated M2 medium (M7167, Sigma‐Aldrich, St. Louis, MO, USA) for subsequent microinjection. For collection of MII oocytes *in vivo*, each mouse was injected with 7.5 IU human chorionic gonadotropin (hCG, Ningbo San Sheng Biotech) at 44–48 h post‐PMSG, and the mice were then euthanized after 14 h. The oocyte and cumulus cell complexes were digested with 3 mg/ml hyaluronidase (H4272, Sigma‐Aldrich) dissolved in M2 medium. Wild‐type and *Erk1* ^−/−^ *; Erk2^f/f^; Gdf9‐Cre* female mice were mated with wild‐type adult male mice and sacrificed at 24, 48, and 96 h post‐hCG for collection of zygotes, two‐cell embryos, and blastocyst embryos, respectively.

For *in vitro* maturation of mouse oocyte, GV oocytes were cultured in M2 medium with indicated drugs for 14 h in an incubator at 37°C in 5% CO_2_. For *in vitro* maturation of human oocyte, immature oocyte (GVBD stage) was cultured in a commercial maturation medium (M2215, Easy Check, China) containing DMSO (C6164, Sigma‐Aldrich) or U0126 (HY‐12031A, MCE) for 24 h in an incubator at 37°C with 6% CO_2_.

### *In vitro* mRNA transcription

*MOS* mRNA variants were synthesized *in vitro* as previously described (Rong *et al*, 2019). Briefly, FLAG‐tagged *MOS* plasmids were liberalized using *KpnI* restriction enzymes. 5'‐capped mRNAs were transcribed using Sp6 or T7 mMESSAGE mMACHINE Kits (AM1340, AM1344, Invitrogen, Carlsbad, CA, USA), followed by poly(A) tail addition using a Poly (A) Tailing Kit (AM1350, Invitrogen). Synthesized poly(A) mRNAs were recovered with lithium chloride precipitation at −20°C, cleared with 70% ethanol, and finally, dissolved in nuclease‐free water.

### Microinjection of siRNAs or mRNAs

All microinjections were performed using a Narishige micromanipulator. Approximately 10 pl of mRNAs (˜500 ng/µl), siRNAs (10 µM), or their mixture was microinjected into the ooplasm. To mimic the compound heterozygous variants in patient 2, we microinjected 250 ng/µl mRNAs for each variant, reaching a total dose of 500 ng/µl *MOS* mRNAs as another two patients with homozygous variants. GV oocytes were cultured in M2 medium with 2.5 µM milrinone (HY14252, MCE) for 24 h for mRNA translation or siRNA knockdown, prior to being released into fresh M2 medium. The sequences of siRNAs are listed in Appendix Table S2.

### Real‐time quantitative PCR (qRT‐PCR)

The mRNA levels were determined using qRT‐PCR and calculated using the comparative Ct method, as previously described (Zhang *et al*, 2015). Total RNA from 20 oocytes was extracted using the RNA‐easy Micro Kit (74004, Qiagen, Hilden, Germany), according to the manufacturer’s protocol. First‐strand cDNA was generated using SuperScript II Reverse Transcriptase (18064014, Thermo Fisher Scientific). Real‐time PCR was conducted with SYBR Green mix using the CFX96 Real‐Time System (Bio‐Rad, Hercules, CA, USA). Mouse *Gapdh* or human *ACTB* expression was used as an internal control. The primers used are listed in Appendix Table S2.

### Immunofluorescence

Human or mouse oocytes were fixed in 3.7% paraformaldehyde diluted in PBS for 30 min at room temperature and then permeabilized with 0.2% TritonX‐100 for 15 min. After incubation for 1 h in blocking buffer (1% BSA diluted in PBS with 0.1% TritonX‐100), oocytes were stained with the indicated primary antibodies diluted in blocking buffer overnight at 4°C. After three washes, samples were incubated with Alexa Fluor 568‐conjugated goat anti‐rabbit (A11036, Invitrogen), Alexa Fluor 488‐conjugated goat anti‐rabbit (A11001, Invitrogen), Alexa Fluor 568‐conjugated donkey anti‐mouse (A10037, Invitrogen) secondary antibodies with dilution of 1:300, in combination with 1 µg/ml DAPI (236276, Roche, Basel, Switzerland), with or without FITC‐α‐tubulin (1:400, F2168, Sigma‐Aldrich), or Alexa Fluor 647‐conjugated phalloidin (1:200, A22287, Invitrogen) for 1 h at room temperature. After washing four times, the oocytes were mounted on slides with anti‐fade medium and imaged using a laser‐scanning confocal microscope (LSM800, Carl Zeiss, Jena, Germany). The primary antibodies used were as follows: mouse monoclonal anti‐FLAG (1:500, F1804, Sigma‐Aldrich), rabbit monoclonal anti‐pERK1/2 (1:400, #4370, Cell Signaling Technology, Danvers, MA, USA), and rabbit polyclonal anti‐TPX2 (1:200, NB500‐179, Novus Biologicals, Littleton, CO, USA).

### Western blotting

Cells transiently expressing the indicated plasmids were lysed using 1× Laemmli sample buffer (1610747, Bio‐Rad). Proteins were separated via SDS‐PAGE and transferred onto a polyvinylidene difluoride (PVDF) membrane. After blocking in TBS containing 5% defat milk for 1 h at room temperature, membranes were incubated with primary antibodies (1:1,000 dilution) overnight at 4°C. After washing three times in TBS‐Tween‐20 (0.05%), membranes were incubated with goat anti‐rabbit horseradish peroxidase‐conjugated (1:5,000, 111‐035‐003, Jackson ImmunoResearch, West Grove, PA, USA) or goat anti‐mouse (1:5,000, 115‐035‐003, Jackson ImmunoResearch) secondary antibodies for 1 h at room temperature. Signals were detected using enhanced chemiluminescence (ECL; Millipore, Burlington, MA, USA). The primary antibodies used were as follows: mouse monoclonal anti‐FLAG (1:1,000, F1804, Sigma‐Aldrich), rabbit monoclonal anti‐pERK1/2 (1:1,000, #4370, Cell Signaling Technology), rabbit monoclonal anti‐ERK1/2 (1:1,000, #4695, Cell Signaling Technology), rabbit anti‐pMEK1/2 (1:500, #9154, Cell Signaling Technology), mouse anti‐MEK1/2 (1:500, #4694, Cell Signaling Technology), rabbit anti‐Histone H3 (1:1,000, #4499, Cell Signaling Technology), rabbit anti‐mCherry (1:1,000, ab183628, Abcam), rabbit anti‐Vinculin (1:1,000, ab207440, Abcam), and mouse polyclonal anti‐α‐tubulin (1:1,000, 32‐2500, Thermo Fisher Scientific).

### Co‐immunoprecipitation

Co‐immunoprecipitation assays were performed as previously described (Zhang *et al*, 2018). Briefly, after transfection of the indicated plasmids for 36 h, cells were washed with cold PBS and then lysed using NP‐40 lysis buffer (P0013F, Beyotime Biotech, China) containing freshly added proteinase inhibitor cocktail (Roche) and 1 mM PMSF (Beyotime Biotech) at 4°C for 30 min. Subsequently, FLAG‐M2 magnetic beads (M8823, Sigma‐Aldrich) were prepared and added to the cell extracts and incubated overnight at 4°C. Beads were washed four to five times with lysis buffer, and the immune complexes were subjected to western blotting.

### Mitochondrial function measurement

ATP level was assayed in denuded human or mouse oocytes at the MII stage, after *in vitro* maturation. ATP content measurement was performed using Enhanced ATP Assay Kit (S0027, Beyotime Biotech) based on the luciferin–luciferase reaction as described previously (Ma *et al*, 2021). Briefly, a standard curve was generated for each series of analyses. MII oocytes after treatment with or without U0126 were harvested, followed by zona pellucida removal in acidic Tyrode's solution (T1788, Sigma). After three washes in 0.1% BSA in PBS, each oocyte was lysed directly with 20 µl lysis buffer on ice for 5 min. Then, 100 μl of ice‐cold ATP assay working solution was added to the oocyte lysis solution and incubated for 10 min at room temperature in the dark. Next, the luminescence of each oocyte was measured using a luminometer (Model TD‐20/20, Turner Biosystems, Madison, WI, USA). The ATP level was calculated using the formula derived from the linear regression of the standard curve.

The mitochondrial membrane potential was determined using a commercial kit with JC‐1 (C2006, Beyotime Biotech) according to the manufacturer's protocol. Briefly, MII oocytes after *in vitro* maturation were transferred to M2 medium with JC‐1 and cultured for 1 h in a 5% CO_2_ incubator at 37°C. Then, oocytes were rinsed several times in fresh M2 medium. Next, the fluorescence images of JC‐1 aggregates and monomer were captured using a fluorescence microscope (Nikon, Japan, Eclipse ts2R), respectively. Notably, we strictly controlled the washing intensities and the exposure time of each group. Fluorescence intensities of aggregates and monomer were quantified using Image J Software (National Institutes of Health, Bethesda, MD, USA). The ratio of aggregates and monomer was calculated to indicate the mitochondrial membrane potential.

### Single oocyte RNA sequencing (RNA‐seq)

Human GV and MII oocytes were donated from unidentified control patients and patients with the *MOS* ^Asn95Lys^ homozygous variant. A single oocyte was lysed using 4 µl cell lysis buffer, and cDNA was obtained via reverse transcription using the SMART‐seq2 method, as previously described (Zhang *et al*, 2019a). In brief, first‐strand cDNA synthesis and amplification were performed using SuperScript II (18064014, Thermo Fisher Scientific) and KAPA Hotstart Hifi ready mix (KK2601, Roche). Sequencing libraries were constructed using TruePrep DNA Library Prep Kit V2 for Illumina (TD503, Vazyme Biotech, Nanjing, China), followed by sequencing on an Illumina NovaSeq 6000 with 150‐bp long paired‐end reads.

### RNA‐seq data analysis

RNA‐seq data were processed using standard procedures as previously described (Zhang *et al*, 2019a). In brief, raw reads were first preprocessed using Trimmomatic (v0.35) to remove adapters, trim low‐quality bases from both read ends, and remove reads < 36 bp in length. Clean reads were then mapped to the human reference genome of GRCh38 using STAR aligner (v2.5.2b). The gene counts were calculated using HTSeq (v0.6.1p1). The expression levels of each gene were quantified using normalized reads per kilobase per million mapped reads (RPKM). Spearman correlation coefficients were analyzed in R. Scatter plots and volcano plot were created using the ggplot2 packages in R, and heatmaps were plotted using pheaetmap packages in R. Gene Ontology (GO) term analysis was performed using DAVID online tool (https://david.ncifcrf.gov). The Venn diagram plots were drawn based on the results of Bioinformatics and Evolutionary Genomics online tool (http://bioinformatics.psb.ugent.be/webtools/Venn/).

### Image acquisition and quantification

For bright‐field image acquisition of oocytes and embryos, a Nikon Ts2R microscope with a Hoffman system was used. Fluorescent images were captured using an LSM800 laser scanning confocal microscope (Carl Zeiss). For oocyte F‐actin acquisition, a layer with maximal intensity was chosen. ImageJ software was used for signal quantification. The signal intensity was measured in an area with an equivalent size selected close to the F‐actin signal, and the mean intensity of the F‐actin was calculated.

### Statistical analysis

All collected oocytes from wild‐type mice were randomized into experimental groups for subsequent microinjection. Then, the survived oocytes in each group were used for experiments and analysis. For the oocyte or embryo phenotype analysis from wild‐type and *Erk1* ^−/−^ *; Erk2^f/f^; Gdf9‐Cre* mice, age‐matched animals were grouped based on their genotype. For the studies using oocytes, N indicates oocyte number or experimental replicates. Two to three independent experiments were conducted.

Statistical analysis was conducted using the GraphPad Prism 7.0 software (GraphPad Software Inc., La Jolla, CA, USA) or Microsoft Office Excel 2016. For the bar graphs, data are expressed as mean ± standard error (SD) for biological replicates or technical replicates. Box plots show the medians, quartiles, and range of continuous data to demonstrate the variability of data and the degree of normality. Differences between two groups were compared using the unpaired two‐tailed Student’s *t*‐test. In the case of more than two groups, one‐way ANOVA was used, followed by *post hoc* Tukey’s test for multiple comparisons. *P* < 0.05 was considered statistically significant.

## Author contributions

SZ, H‐YF, and GL conceived the project. Y‐LZ, WZ, and H‐YF designed the experiments. Y‐LZ and WZ performed most of the experiments and analyzed the data. PR microinjected mRNAs or siRNAs in mouse oocytes and performed western blot analysis. XL analyzed the RNA‐seq data. HH, XT, and S‐PZ recruited the patients. HW and JJ collected the human samples. WY, YZ, and YH constructed libraries of single cell RNA‐seq. J‐CJ and YM assisted for animal housing and genotyping for *Erk1/2* deficient mice. LC constructed one of the plasmids. YG, LH, KL, FG, and G‐XL assisted for human embryos phenotype monitoring. Y‐LZ and WZ wrote the draft of manuscript, with contributions and approval from all authors. H‐YF, SZ, and GL critically revised the manuscript. All authors read and approved the final manuscript.

## Conflict of interest

The authors declare that they have no conflict of interest.

## For more information

(i) GenBank, https://www.ncbi.nlm.nih.gov/genbank/
(ii) OMIM, https://www.omim.org/
(iii) Uniprot, https://www.uniprot.org/
(iv) dbSNP, https://www.ncbi.nlm.nih.gov/projects/SNP/
(v) PyMol software, https://pymol.org/2/
(vi) Swiss Model web server, https://swissmodel.expasy.org/
(vii) ExAC Browser, http://exac.broadinstitute.org/
(viii) gnomAD, https://gnomad.broadinstitute.org/
(ix) PolyPhen‐2, http://genetics.bwh.harvard.edu/pph2/
(x) SIFT, https://sift.bii.a‐star.edu.sg/
(xi) DAVID, https://david.ncifcrf.gov/
(xii) UCSC Genome Browser, http://genome.ucsc.edu/
(xiii) CADD, https://cadd.gs.washington.edu
(xiv) PrimerBank, https://pga.mgh.harvard.edu/primerbank/

## Supporting information



Appendix

Expanded View Figures PDF

Dataset EV1

Dataset EV2

Source Data for Expanded View and Appendix

Source Data for Figure 1

Source Data for Figure 2

Source Data for Figure 3

Source Data for Figure 4

Source Data for Figure 5

Source Data for Figure 6

## Data Availability

The processing single‐cell RNA‐seq dataset supporting conclusion is included within the supplemental data. The single‐cell RNA‐seq raw data generated in the manuscript is available in GSA‐Human under the accession code HRA001269 (https://bigd.big.ac.cn/gsa‐human/browse/HRA001269) or from the corresponding author upon reasonable request.

## References

[emmm202114887-bib-0001] Alazami AM , Awad SM , Coskun S , Al‐Hassan S , Hijazi H , Abdulwahab FM , Poizat C , Alkuraya FS (2015) TLE6 mutation causes the earliest known human embryonic lethality. Genome Biol 16: 240 26537248 10.1186/s13059-015-0792-0 PMC4634911

[emmm202114887-bib-0002] Araki K , Naito K , Haraguchi S , Suzuki R , Yokoyama M , Inoue M , Aizawa S , Toyoda Y , Sato E (1996) Meiotic abnormalities of c‐mos knockout mouse oocytes: activation after first meiosis or entrance into third meiotic metaphase. Biol Reprod 55: 1315–1324 8949889 10.1095/biolreprod55.6.1315

[emmm202114887-bib-0003] Booy EP , Henson ES , Gibson SB (2011) Epidermal growth factor regulates Mcl‐1 expression through the MAPK‐Elk‐1 signalling pathway contributing to cell survival in breast cancer. Oncogene 30: 2367–2378 21258408 10.1038/onc.2010.616 PMC3145838

[emmm202114887-bib-0004] Boucher MJ , Morisset J , Vachon PH , Reed JC , Laine J , Rivard N (2000) MEK/ERK signaling pathway regulates the expression of Bcl‐2, Bcl‐X(L), and Mcl‐1 and promotes survival of human pancreatic cancer cells. J Cell Biochem 79: 355–369 10972974

[emmm202114887-bib-0005] Cao LR , Jiang JC , Fan HY (2020) Positive feedback stimulation of Ccnb1 and Mos mRNA translation by MAPK cascade during mouse oocyte maturation. Front Cell Dev Biol 8: 609430 33282880 10.3389/fcell.2020.609430 PMC7691486

[emmm202114887-bib-0006] Chaigne A , Campillo C , Gov NS , Voituriez R , Azoury J , Umaña‐Diaz C , Almonacid M , Queguiner I , Nassoy P , Sykes C *et al* (2013) A soft cortex is essential for asymmetric spindle positioning in mouse oocytes. Nat Cell Biol 15: 958–966 23851486 10.1038/ncb2799

[emmm202114887-bib-0007] Chan SH , Tang Y , Miao L , Darwich‐Codore H , Vejnar CE , Beaudoin J‐D , Musaev D , Fernandez JP , Benitez MDJ , Bazzini AA *et al* (2019) Brd4 and P300 confer transcriptional competency during zygotic genome activation. Dev Cell 49: 867–881 31211993 10.1016/j.devcel.2019.05.037 PMC7201981

[emmm202114887-bib-0008] Choi T , Fukasawa K , Zhou R , Tessarollo L , Borror K , Resau J , Vande Woude GF (1996a) The Mos/mitogen‐activated protein kinase (MAPK) pathway regulates the size and degradation of the first polar body in maturing mouse oocytes. Proc Natl Acad Sci USA 93: 7032–7035 8692939 10.1073/pnas.93.14.7032 PMC38930

[emmm202114887-bib-0009] Choi T , Rulong S , Resau J , Fukasawa K , Matten W , Kuriyama R , Mansour S , Ahn N , Vande Woude GF (1996b) Mos/mitogen‐activated protein kinase can induce early meiotic phenotypes in the absence of maturation‐promoting factor: a novel system for analyzing spindle formation during meiosis I. Proc Natl Acad Sci USA 93: 4730–4735 8643471 10.1073/pnas.93.10.4730 PMC39347

[emmm202114887-bib-0010] Colledge WH , Carlton MB , Udy GB , Evans MJ (1994) Disruption of c‐mos causes parthenogenetic development of unfertilized mouse eggs. Nature 370: 65–68 8015609 10.1038/370065a0

[emmm202114887-bib-0011] Cook SJ , Stuart K , Gilley R , Sale MJ (2017) Control of cell death and mitochondrial fission by ERK1/2 MAP kinase signalling. FEBS J 284: 4177–4195 28548464 10.1111/febs.14122 PMC6193418

[emmm202114887-bib-0012] de Moor CH , Richter JD (1997) The Mos pathway regulates cytoplasmic polyadenylation in Xenopus oocytes. Mol Cell Biol 17: 6419–6426 9343404 10.1128/mcb.17.11.6419 PMC232494

[emmm202114887-bib-0013] Ebner T , Yaman C , Moser M , Sommergruber M , Polz W , Tews G (2001) Embryo fragmentation *in vitro* and its impact on treatment and pregnancy outcome. Fertil Steril 76: 281–285 11476773 10.1016/s0015-0282(01)01904-5

[emmm202114887-bib-0014] Feng R , Sang Q , Kuang Y , Sun X , Yan Z , Zhang S , Shi J , Tian G , Luchniak A , Fukuda Y *et al* (2016) Mutations in TUBB8 and human oocyte meiotic arrest. N Engl J Med 374: 223–232 26789871 10.1056/NEJMoa1510791 PMC4767273

[emmm202114887-bib-0015] Fujimoto VY , Browne RW , Bloom MS , Sakkas D , Alikani M (2011) Pathogenesis, developmental consequences, and clinical correlations of human embryo fragmentation. Fertil Steril 95: 1197–1204 21146166 10.1016/j.fertnstert.2010.11.033

[emmm202114887-bib-0016] Hashiba Y , Asada Y , Heikinheimo O , Lanzendorf SE , Mizutani S (2001) Microinjection of antisense c‐mos oligonucleotides prevents the progression of meiosis in human and hamster oocytes. Fertil Steril 76: 143–147 11438333 10.1016/s0015-0282(01)01821-0

[emmm202114887-bib-0017] Hashimoto N , Watanabe N , Furuta Y , Tamemoto H , Sagata N , Yokoyama M , Okazaki K , Nagayoshi M , Takedat N , Ikawatll Y *et al* (1994) Parthenogenetic activation of oocytes in c‐mos‐deficient mice. Nature 370: 68–71 8015610 10.1038/370068a0

[emmm202114887-bib-0018] Holubcova Z , Blayney M , Elder K , Schuh M (2015) Human oocytes. Error‐prone chromosome‐mediated spindle assembly favors chromosome segregation defects in human oocytes. Science 348: 1143–1147 26045437 10.1126/science.aaa9529 PMC4477045

[emmm202114887-bib-0019] Jiang JC , Zhang H , Cao LR , Dai XX , Zhao LW , Liu HB , Fan HY (2021) Oocyte meiosis‐coupled poly(A) polymerase alpha phosphorylation and activation trigger maternal mRNA translation in mice. Nucleic Acids Res 49: 5867–5880 34048556 10.1093/nar/gkab431 PMC8191758

[emmm202114887-bib-0020] Kashatus JA , Nascimento A , Myers LJ , Sher A , Byrne FL , Hoehn KL , Counter CM , Kashatus DF (2015) Erk2 phosphorylation of Drp1 promotes mitochondrial fission and MAPK‐driven tumor growth. Mol Cell 57: 537–551 25658205 10.1016/j.molcel.2015.01.002 PMC4393013

[emmm202114887-bib-0021] Kawahara M , Mori T , Tanaka H , Shimizu H (2002) The suppression of fragmentation by stabilization of actin filament in porcine enucleated oocytes. Theriogenology 58: 1081–1095 12240912 10.1016/s0093-691x(02)00939-1

[emmm202114887-bib-0022] Ma Y , Yang W , Ren P , Li X , Jin J , Dai Y , Pan Y , Jiang L , Fan H , Zhang Y , & *et al* (2021) Lysophosphatidic acid improves oocyte quality during IVM by activating the ERK1/2 pathway in cumulus cells and oocytes. Mol Hum Reprod 27: gaab032 33944929 10.1093/molehr/gaab032

[emmm202114887-bib-0023] Morimoto N , Hashimoto S , Yamanaka M , Nakano T , Satoh M , Nakaoka Y , Iwata H , Fukui A , Morimoto Y , Shibahara H (2020) Mitochondrial oxygen consumption rate of human embryos declines with maternal age. J Assist Reprod Genet 37: 1815–1821 32740687 10.1007/s10815-020-01869-5 PMC7467997

[emmm202114887-bib-0024] Mu J , Wang W , Chen B , Wu L , Li B , Mao X , Zhang Z , Fu J , Kuang Y , Sun X *et al* (2019) Mutations in NLRP2 and NLRP5 cause female infertility characterised by early embryonic arrest. J Med Genet 56: 471–480 30877238 10.1136/jmedgenet-2018-105936

[emmm202114887-bib-0025] Prasad CK , Mahadevan M , MacNicol MC , MacNicol AM (2008) Mos 3' UTR regulatory differences underlie species‐specific temporal patterns of Mos mRNA cytoplasmic polyadenylation and translational recruitment during oocyte maturation. Mol Reprod Dev 75: 1258–1268 18246541 10.1002/mrd.20877 PMC2440637

[emmm202114887-bib-0026] Prieto J , Leon M , Ponsoda X , Sendra R , Bort R , Ferrer‐Lorente R , Raya A , Lopez‐Garcia C , Torres J (2016) Early ERK1/2 activation promotes DRP1‐dependent mitochondrial fission necessary for cell reprogramming. Nat Commun 7: 11124 27030341 10.1038/ncomms11124 PMC4821885

[emmm202114887-bib-0027] Pyakurel A , Savoia C , Hess D , Scorrano L (2015) Extracellular regulated kinase phosphorylates mitofusin 1 to control mitochondrial morphology and apoptosis. Mol Cell 58: 244–254 25801171 10.1016/j.molcel.2015.02.021 PMC4405354

[emmm202114887-bib-0028] Richani D , Dunning KR , Thompson JG , Gilchrist RB (2021) Metabolic co‐dependence of the oocyte and cumulus cells: essential role in determining oocyte developmental competence. Hum Reprod Update 27: 27–47 33020823 10.1093/humupd/dmaa043

[emmm202114887-bib-0029] Roeles J , Tsiavaliaris G (2019) Actin‐microtubule interplay coordinates spindle assembly in human oocytes. Nat Commun 10: 4651 31604948 10.1038/s41467-019-12674-9 PMC6789129

[emmm202114887-bib-0030] Rong Y , Ji SY , Zhu YZ , Wu YW , Shen L , Fan HY (2019) ZAR1 and ZAR2 are required for oocyte meiotic maturation by regulating the maternal transcriptome and mRNA translational activation. Nucleic Acids Res 47: 11387–11402 31598710 10.1093/nar/gkz863 PMC6868374

[emmm202114887-bib-0031] Sagata N , Oskarsson M , Copeland T , Brumbaugh J , Vande Woude GF (1988) Function of c‐mos proto‐oncogene product in meiotic maturation in *Xenopus oocytes* . Nature 335: 519–525 2971141 10.1038/335519a0

[emmm202114887-bib-0032] Sagata N , Watanabe N , Vande Woude GF , Ikawa Y (1989) The c‐mos proto‐oncogene product is a cytostatic factor responsible for meiotic arrest in vertebrate eggs. Nature 342: 512–518 2531292 10.1038/342512a0

[emmm202114887-bib-0033] Sako K , Suzuki K , Isoda M , Yoshikai S , Senoo C , Nakajo N , Ohe M , Sagata N (2014) Emi2 mediates meiotic MII arrest by competitively inhibiting the binding of Ube2S to the APC/C. Nat Commun 5: 3667 24770399 10.1038/ncomms4667

[emmm202114887-bib-0034] Sha QQ , Dai XX , Dang Y , Tang F , Liu J , Zhang YL , Fan HY (2017) A MAPK cascade couples maternal mRNA translation and degradation to meiotic cell cycle progression in mouse oocytes. Development 144: 452–463 27993988 10.1242/dev.144410

[emmm202114887-bib-0035] Sha QQ , Zhang J , Fan HY (2019) A story of birth and death: mRNA translation and clearance at the onset of maternal‐to‐zygotic transition in mammalsdagger. Biol Reprod 101: 579–590 30715134 10.1093/biolre/ioz012

[emmm202114887-bib-0036] Sha QQ , Zheng W , Wu YW , Li S , Guo L , Zhang S , Lin G , Ou XH , Fan HY (2020a) Dynamics and clinical relevance of maternal mRNA clearance during the oocyte‐to‐embryo transition in humans. Nat Commun 11: 4917 33004802 10.1038/s41467-020-18680-6 PMC7530992

[emmm202114887-bib-0037] Sha QQ , Zhu YZ , Li S , Jiang Y , Chen L , Sun XH , Shen L , Ou XH , Fan HY (2020b) Characterization of zygotic genome activation‐dependent maternal mRNA clearance in mouse. Nucleic Acids Res 48: 879–894 31777931 10.1093/nar/gkz1111 PMC6954448

[emmm202114887-bib-0038] She QB , Ma WY , Zhong S , Dong Z (2002) Activation of JNK1, RSK2, and MSK1 is involved in serine 112 phosphorylation of Bad by ultraviolet B radiation. J Biol Chem 277: 24039–24048 11983683 10.1074/jbc.M109907200

[emmm202114887-bib-0039] Shoji S , Yoshida N , Amanai M , Ohgishi M , Fukui T , Fujimoto S , Nakano Y , Kajikawa E , Perry AC (2006) Mammalian Emi2 mediates cytostatic arrest and transduces the signal for meiotic exit via Cdc20. EMBO J 25: 834–845 16456547 10.1038/sj.emboj.7600953 PMC1383546

[emmm202114887-bib-0040] Stigliani S , Anserini P , Venturini PL , Scaruffi P (2013) Mitochondrial DNA content in embryo culture medium is significantly associated with human embryo fragmentation. Hum Reprod 28: 2652–2660 23887072 10.1093/humrep/det314

[emmm202114887-bib-0041] Suzuki T , Suzuki E , Yoshida N , Kubo A , Li H , Okuda E , Amanai M , Perry AC (2010) Mouse Emi2 as a distinctive regulatory hub in second meiotic metaphase. Development 137: 3281–3291 20724447 10.1242/dev.052480 PMC2934736

[emmm202114887-bib-0042] Trebichalska Z , Kyjovska D , Kloudova S , Otevrel P , Hampl A , Holubcova Z (2021) Cytoplasmic maturation in human oocytes: an ultrastructural study dagger. Biol Reprod 104: 106–116 33404651 10.1093/biolre/ioaa174 PMC7786262

[emmm202114887-bib-0043] VanBrocklin MW , Verhaegen M , Soengas MS , Holmen SL (2009) Mitogen‐activated protein kinase inhibition induces translocation of Bmf to promote apoptosis in melanoma. Cancer Res 69: 1985–1994 19244105 10.1158/0008-5472.CAN-08-3934 PMC2665192

[emmm202114887-bib-0044] Verlhac MH , Lefebvre C , Kubiak JZ , Umbhauer M , Rassinier P , Colledge W , Maro B (2000) Mos activates MAP kinase in mouse oocytes through two opposite pathways. EMBO J 19: 6065–6074 11080153 10.1093/emboj/19.22.6065 PMC305841

[emmm202114887-bib-0045] Watanabe N , Hunt T , Ikawa Y , Sagata N (1991) Independent inactivation of MPF and cytostatic factor (Mos) upon fertilization of *Xenopus eggs* . Nature 352: 247–248 1830371 10.1038/352247a0

[emmm202114887-bib-0046] Xu Y , Shi Y , Fu J , Yu M , Feng R , Sang Q , Liang BO , Chen B , Qu R , Li B *et al* (2016) Mutations in PADI6 cause female infertility characterized by early embryonic arrest. Am J Hum Genet 99: 744–752 27545678 10.1016/j.ajhg.2016.06.024 PMC5010645

[emmm202114887-bib-0048] Yan L , Yang M , Guo H , Yang L , Wu J , Li R , Liu P , Lian Y , Zheng X , Yan J *et al* (2013) Gene Expression Omnibus GSE36552 (https://www.ncbi.nlm.nih.gov/geo/query/acc.cgi?acc=GSE36552). [DATASET]

[emmm202114887-bib-0049] Yu C , Ji S‐Y , Sha Q‐Q , Dang Y , Zhou J‐J , Zhang Y‐L , Liu Y , Wang Z‐W , Hu B , Sun Q‐Y *et al* (2016) BTG4 is a meiotic cell cycle‐coupled maternal‐zygotic‐transition licensing factor in oocytes. Nat Struct Mol Biol 23: 387–394 27065194 10.1038/nsmb.3204

[emmm202114887-bib-0050] Zhang C , Tao L , Yue Y , Ren L , Zhang Z , Wang X , Tian J , An L (2021) Mitochondrial transfer from induced pluripotent stem cells rescues developmental potential of *in vitro* fertilized embryos from aging femalesdagger. Biol Reprod 104: 1114–1125 33511405 10.1093/biolre/ioab009

[emmm202114887-bib-0051] Zhang J , Zhang Y‐L , Zhao L‐W , Guo J‐X , Yu J‐L , Ji S‐Y , Cao L‐R , Zhang S‐Y , Shen LI , Ou X‐H *et al* (2019a) Mammalian nucleolar protein DCAF13 is essential for ovarian follicle maintenance and oocyte growth by mediating rRNA processing. Cell Death Differ 26: 1251–1266 30283081 10.1038/s41418-018-0203-7 PMC6748096

[emmm202114887-bib-0052] Zhang W , Chen Z , Zhang D , Zhao B , Liu L , Xie Z , Yao Y , Zheng P (2019b) KHDC3L mutation causes recurrent pregnancy loss by inducing genomic instability of human early embryonic cells. PLoS Biol 17: e3000468 31609975 10.1371/journal.pbio.3000468 PMC6812846

[emmm202114887-bib-0053] Zhang YL , Liu XM , Ji SY , Sha QQ , Zhang J , Fan HY (2015) ERK1/2 activities are dispensable for oocyte growth but are required for meiotic maturation and pronuclear formation in mouse. J Genet Genomics 42: 477–485 26408092 10.1016/j.jgg.2015.07.004

[emmm202114887-bib-0054] Zhang YL , Zhao LW , Zhang J , Le R , Ji SY , Chen C , Gao Y , Li D , Gao S , Fan HY (2018) DCAF13 promotes pluripotency by negatively regulating SUV39H1 stability during early embryonic development. EMBO J 37: e98981 30111536 10.15252/embj.201898981 PMC6138440

[emmm202114887-bib-0055] Zhao BS , Wang X , Beadell AV , Lu Z , Shi H , Kuuspalu A , Ho RK , He C (2017) m(6)A‐dependent maternal mRNA clearance facilitates zebrafish maternal‐to‐zygotic transition. Nature 542: 475–478 28192787 10.1038/nature21355 PMC5323276

[emmm202114887-bib-0056] Zhao LW , Zhu YZ , Chen H , Wu YW , Pi SB , Chen L , Shen L , Fan HY (2020) PABPN1L mediates cytoplasmic mRNA decay as a placeholder during the maternal‐to‐zygotic transition. EMBO Rep 21: e49956 32558204 10.15252/embr.201949956 PMC7403729

[emmm202114887-bib-0057] Zheng W , Chen L , Dai J , Dai C , Guo J , Lu C , Gong F , Lu G , Lin G (2020a) New biallelic mutations in PADI6 cause recurrent preimplantation embryonic arrest characterized by direct cleavage. J Assist Reprod Genet 37: 205–212 31664658 10.1007/s10815-019-01606-7 PMC7000584

[emmm202114887-bib-0058] Zheng W , Zhou Z , Sha Q , Niu X , Sun X , Shi J , Zhao L , Zhang S , Dai J , Cai S *et al* (2020b) Homozygous mutations in BTG4 cause zygotic cleavage failure and female infertility. Am J Hum Genet 107: 24–33 32502391 10.1016/j.ajhg.2020.05.010 PMC7332666

