# Supplementary material for: Biallelic mutations in MOS cause female infertility characterized by human early embryonic arrest and fragmentation
Source: EMBO Mol Med. 2021 Nov 15;13(12):e14887. doi: 10.15252/emmm.202114887 (PMC8649871; doi:10.15252/emmm.202114887)
Supplement: Supplementary file 1 — Appendix [file EMMM-13-e14887-s003.pdf]

APPENDIX

Biallelic mutations in *MOS* cause female infertility characterized by human early embryonic arrest and fragmentation

Yin-Li Zhang[1,4,7], Wei Zheng[2,5,7], Peipei Ren[1,4], Huiling Hu[2,5], Xiaomei Tong[1,4], Shuoping Zhang[2,5], Xiang Li[1,4], Haichao Wang[1,4], Junchao Jiang[3], Jiamin Jin[1,4], Weijie Yang[1,4], Lanrui Cao[3], Yuanlin He[6], Yerong Ma[1,4], Yingyi Zhang[1,4], Yifan Gu[2,5], Liang Hu[2,5], Keli Luo[2,5], Fei Gong[2,5], Guangxiu Lu[2,5], Ge Lin[2,5,*], Heng-Yu Fan[3,4,*], Songying Zhang[1,4,*]

1. Assisted Reproduction Unit, Department of Obstetrics and Gynecology, Sir Run Run Shaw Hospital, School of Medicine, Zhejiang University, Hangzhou, 310016, China.
2. Clinical Research Center for Reproduction and Genetics in Hunan Province, Reproductive and Genetic Hospital of CITIC-Xiangya, Changsha, 410078, China.
3. Life Sciences Institute, Zhejiang University, Hangzhou, 310058, China.
4. Key Laboratory of Reproductive Dysfunction Management of Zhejiang Province, Hangzhou, China.
5. Laboratory of Reproductive and Stem Cell Engineering, NHC Key Laboratory of Human Stem Cell and Reproductive Engineering, Central South University, Changsha, 410008, China.
6. Department of Epidemiology, Center for Global Health, School of Public Health, Nanjing Medical University, Nanjing, 211166, China.
7. These authors contributed equally to this work.

*Corresponding authors:

Songying Zhang, zhangsongying@zju.edu.cn

Heng-Yu Fan, hyfan@zju.edu.cn

Ge Lin, linggf@hotmail.com

Table of contents

Appendix Figure S1: The effects of *MOS* variants on protein localization, ERK1/2 activation and the binding capacity with MEK1.

Appendix Figure S2: ERK1/2 inactivation causes oocyte cytoskeleton assembly disorder during meiotic maturation.

Appendix Figure S3: RT-qPCR analysis of selective genes with or without U0126 treatment during human oocyte maturation.

Appendix Figure S4: The dose effect of *MOS* mRNAs for promotion of meiosis maturation.

Appendix Table S1: Menstrual cycles and sex hormone levels of patients with EEAF.

Appendix Table S2: Primers and siRNA Oligos List

Appendix Figure S1

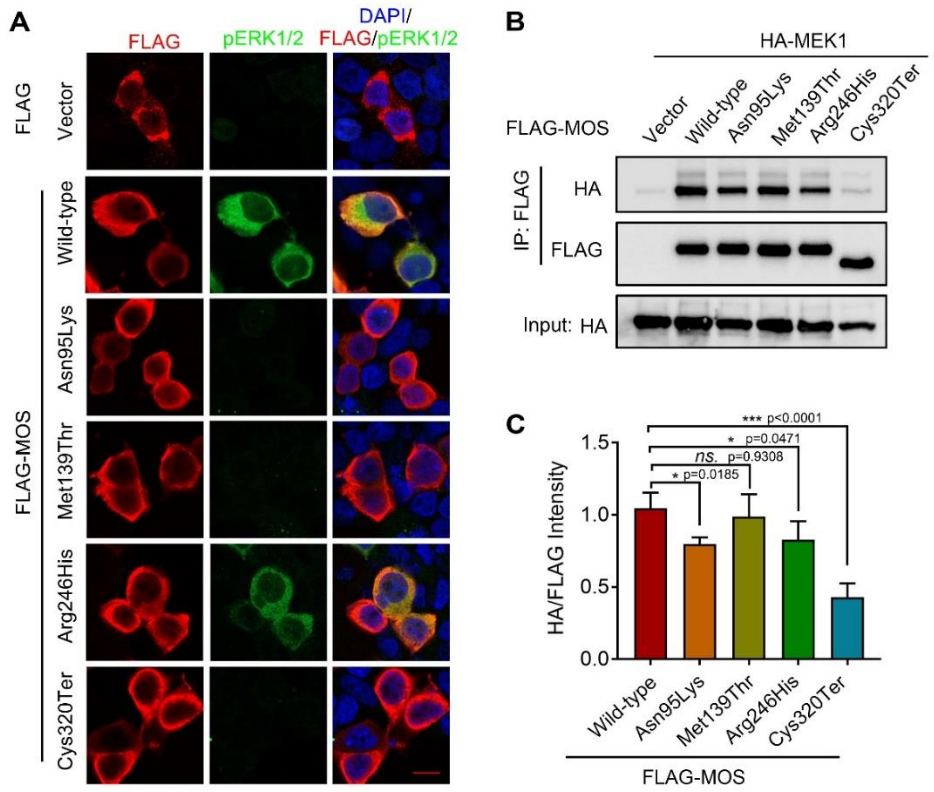

Appendix Figure S1. The effects of *MOS* variants on subcellular localization, ERK1/2 activation and the binding capacity with MEK1.

A. Representative confocal images labeled with FLAG (red), pERK1/2 (green) and DAPI (blue) in HEK293 cells after transfection of indicated *MOS* variants. Scale bar=10 µm.

B. Anti-FLAG immunoprecipitation of extracts from HEK293 cells transiently expressing HA-MEK1 in combination with FLAG-tagged *MOS* or indicated *MOS* variants. Input and immunoprecipitates (IP) were analyzed using specified antibodies. Two independent experiments were performed.

C. Bar graph quantifying the mean intensity for HA relative to FLAG signal after IP described in (B).

Data information: For (C), data are expressed as mean ± SD. One-way ANOVA, followed by *post hoc* Tukey's test for multiple comparisons, ns., no significance; *, *P*<0.05; ***, *P*<0.001.

Appendix Figure S2

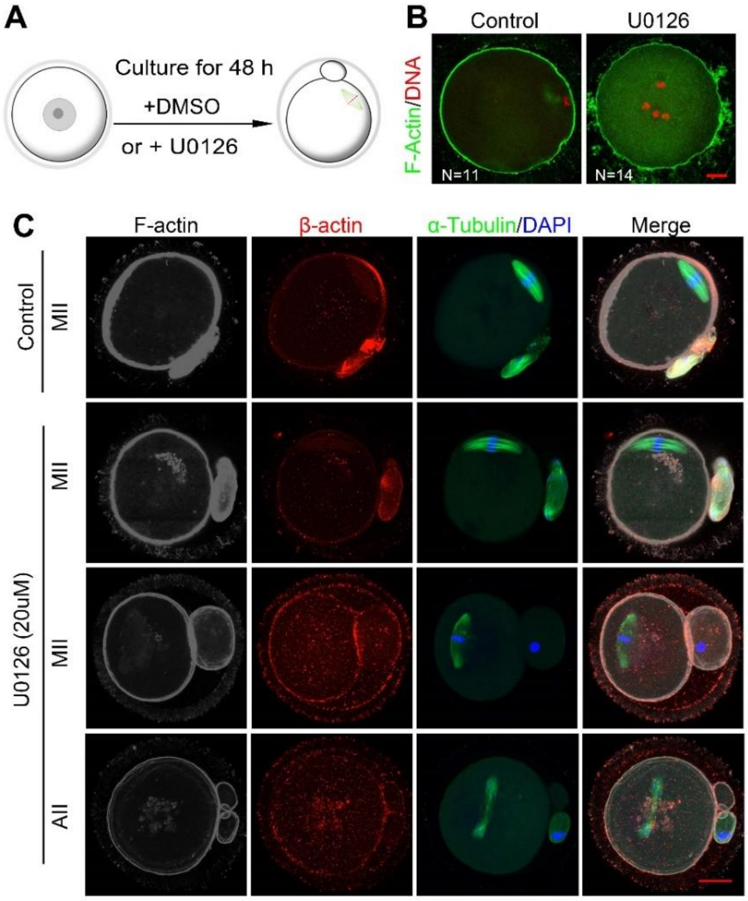

Appendix Figure S2. ERK1/2 inactivation causes oocyte cytoskeleton assembly disorder during meiotic maturation.

A. The diagram of experimental procedure. The immature (geminal vesical breakdown, GVBD) human oocytes were cultured in a medium containing DMSO or 20 μM U0126 for 48 h.

B. The immunofluorescence of F-actin (green) and DNA (red) in human oocytes. Scale bar=20 μm.

C. Mouse GV oocytes were cultured in M2 medium with or without 20 μM U0126 for 14 h *in vitro*. Mature oocytes were fixed and immuno-stained with antibodies against β-actin (red) and FITC-α-tubulin (green), as well as co-stained with phalloidin (gray) and DAPI (blue). Scale bar=20 μm. MII, metaphase II; AII, anaphase II.

Appendix Figure S3

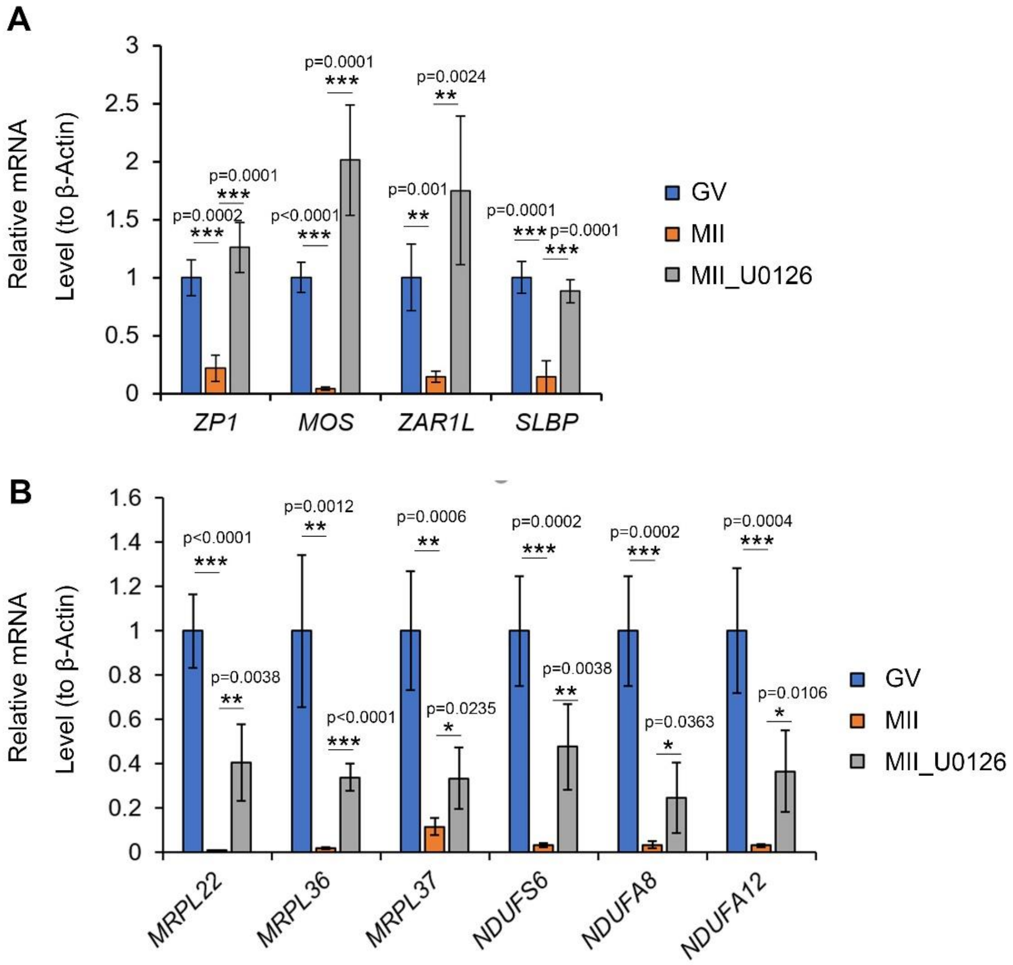

Appendix Figure S3. ERK1/2 inactivation retarded some maternal genes degradation during human oocyte maturation.

A-B. RT-qPCR results showing the relative mRNA level of selected oocyte-specific genes (A) and genes relative to mitochondrial function (B). β-Actin was used as an internal control.

Data information: Data are expressed as mean ± SD of two biological replicates. Unpaired two-tailed Student's *t* test was used for statistical analysis. *, *P*<0.05; **, *P*<0.01; ***, *P*<0.001. Detailed *P* value as indicated.

Appendix Figure S4.

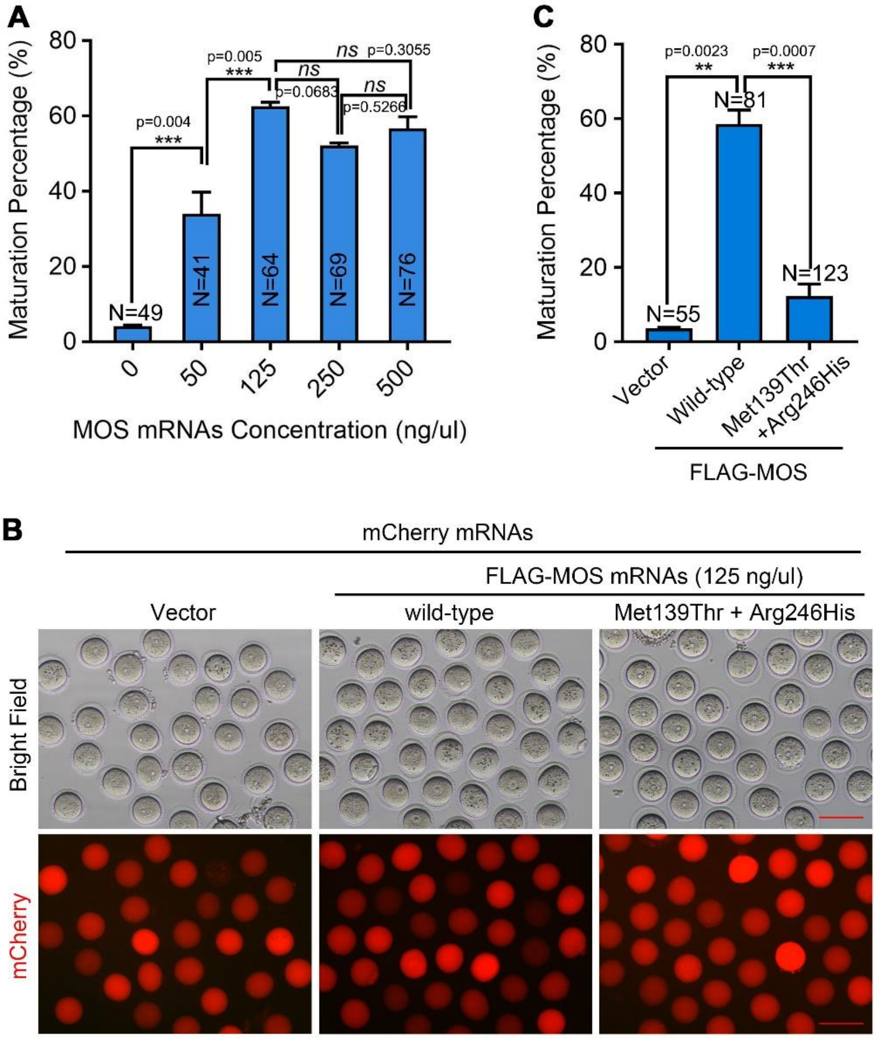

Appendix Figure S4. The dose effect of MOS mRNAs for promotion of meiosis maturation.

A. The statistical result of oocyte meiotic maturation percentage after microinjection with mCherry (500 ng/ul) and different concentrations (0, 50, 125, 250 and 500 ng/ul) of wild-type *MOS* mRNAs. The oocyte number per group was labeled.

B. The representative images showing the oocyte morphology and mCherry signal after microinjection with mCherry in combined with 125 ng/ul wild-type *MOS* mRNAs or the two variants in patient 2. Scale bar=100 µm.

C. The statistical result of oocyte meiotic maturation percentage after microinjection with mCherry (500 ng/ul) and 125 ng/ul wild-type *MOS* mRNAs or the combined two variants found in patient 2. The oocyte number per group was labeled.

Data information: Data are expressed as mean±SD. One-way ANOVA with *post hoc* Tukey's test for multiple comparisons in (A) and unpaired two-tailed Student's *t* test (C) were used for statistical analysis. ns., no significance; **, $P<0.01$; ***, $P<0.001$. Detailed *P* value as indicated.

Appendix Table S1. Menstrual Cycles and Basic Sex Hormone Levels of Patients with EEAF.

**Patients**	**Menstrual cycles (days)**	**hFSH (mIU/mL)**	**hLH (mIU/mL)**	**E2 (pg/mL)**	**Prog (ng/mL)**	**Testo (ng/mL)**	**PRL (ng/mL)**
Patient 1	29-30	6.02	6.30	41.03	0.45	4.67	12.61
Patient 2	29-30	8.70	3.95	14.00	0.5	0.51	12.79
Patient 3	26-28	6.77	4.41	53.84	0.76	0.4	21.86

Abbreviations are as follows: hFSH, human follicle-stimulating hormone; hLH, human luteinizing hormone; E2, estradiol; Testo, testosterone; Prog, progestin; PRL, prolactin.

Appendix Table S2. Primers and siRNA Oligos List

Target Gene	Species	Oligo Sequences (5'-3')	Application
MOS	Human	Forward: ATTGACTGGGAGCAGGTGTG<br>Reverse: AACTCCATGATGATGGTCCCTA	PCR primers for identification of c.285C>A (p.Asn95Lys) variant
MOS	Human	Forward: CTTACCGCGGTGTTCCTGTG<br>Reverse: CCACCACCGCGTACAGTAT	PCR primers for identification of c.416T>C (p.Met139Thr) and c.737G>A, (p.Arg246His) variant
MOS	Human	Forward: GTACTCACTAGATGTTGTGAACGG<br>Reverse: TTCTTCGACATCTCCACTTCC	PCR primers for identification of c.960C>A, (p.Cys320Ter) variant
MOS	Human	Forward: CAAGTGCACCAAGAAACGACTAGCATCTCG<br>Reverse: CGAGATGCTAGTCGTTTCTTGGTGCACTTG	Mutagenesis primers for c.285C>A (p.Asn95Lys) variant
MOS	Human	Forward: GGGACCATCATCACGGAGTTCGGTGG<br>Reverse: CCACCGAACTCCGTGATGATGGTCCC	Mutagenesis primers for c.416T>C (p.Met139Thr) variant
MOS	Human	Forward: CATACACCCACCACGCCCCGGAG<br>Reverse: CTCCGGGGCGTGGTGGGTGTATG	Mutagenesis primers for c.737G>A (p.Arg246His) variant
MOS	Human	Forward: CGTCATCCAGCGCTGATGGAGACCCAGCGC<br>Reverse: GCGCTGGGTCTCCATCAGCGCTGGATGACG	Mutagenesis primers for c.960C>A, (p.Cys320Ter) variant
Mos	Mouse	Forward: TGGCTGGTTTTGAGAATCAAGG<br>Reverse: GTCACATGAGACACTAGGGAGA	RT-qPCR primers
Gapdh	Mouse	Forward: AGGTCGGTGTGAACGGATTTG<br>Reverse: TGTAGACCATGTAGTTGAGGTCA	RT-qPCR primers

N/A	N/A	Sense: TTCTCCGAACGTGTCACGTTT	siRNA against negative control
Mos	Mouse	Sense: GGAAAGACACTGCAGAACA	siRNAs against mouse *Mos*
		Sense: GCCTAGGTACCATAATCAT	
		Sense: CCATCAAGCAAGTAAACAA	
MOS	Human	Forward: TCACTAGATGTTGTGAACGGC	RT-qPCR primers
		Reverse: TCAGAGCAACCGAAGTCACTAA	
ZP1	Human	Forward: ACGACCTGGGGTTACCCTG	RT-qPCR primers
		Reverse: AGCTGCATTCCCTTGATCCC	
ZAR1L	Human	Forward: CTATGGCTTGTACCAGGGTTATG	RT-qPCR primers
		Reverse: CTTGTAAGGGTCAATGCAGTAGT	
SLBP	Human	Forward: GACCCGAGAGCTTTACCACTC	RT-qPCR primers
		Reverse: GGCACAGTAGACATAGACTCCT	
MRPL22	Human	Forward: CGAGGAATGTCTATTGACCAGGC	RT-qPCR primers
		Reverse: CGGATGCGTTTCAGGCACT	
MRPL36	Human	Forward: TGCGCTCACTTCTCTCACC	RT-qPCR primers
		Reverse: ACAGTAGACGTACCACCGACC	
MRPL37	Human	Forward: TCCCCTGGATAGGGTGTACG	RT-qPCR primers
		Reverse: GAGCGGTAGAACCTTGGGT	
NDUFS6	Human	Forward: TTCGGTTTGTAGGTCGTCAGA	RT-qPCR primers
		Reverse: CCATCGCACGCTATCACCC	
NDUFA8	Human	Forward: CCCAACAAGGAGTTTATGCTCT	RT-qPCR primers
		Reverse: CACAGTGACGTTTTATCTGCCT	
NDUFA12	Human	Forward: GGTCTCCGAGGCTATCTACGG	RT-qPCR primers
		Reverse: GGAGGCACCATGCTTCCATC	
ACTB	Human	Forward: CATGTACGTTGCTATCCAGGC	RT-qPCR primers
		Reverse: CTCCTTAATGTCACGCACGAT	
