# Supplementary material for: Biallelic mutations in MOS cause female infertility characterized by human early embryonic arrest and fragmentation
Source: EMBO Mol Med. 2021 Nov 15;13(12):e14887. doi: 10.15252/emmm.202114887 (PMC8649871; doi:10.15252/emmm.202114887)
Supplement: Supplementary file 2 — Expanded View Figures PDF [file EMMM-13-e14887-s010.pdf]

Expanded View Figures

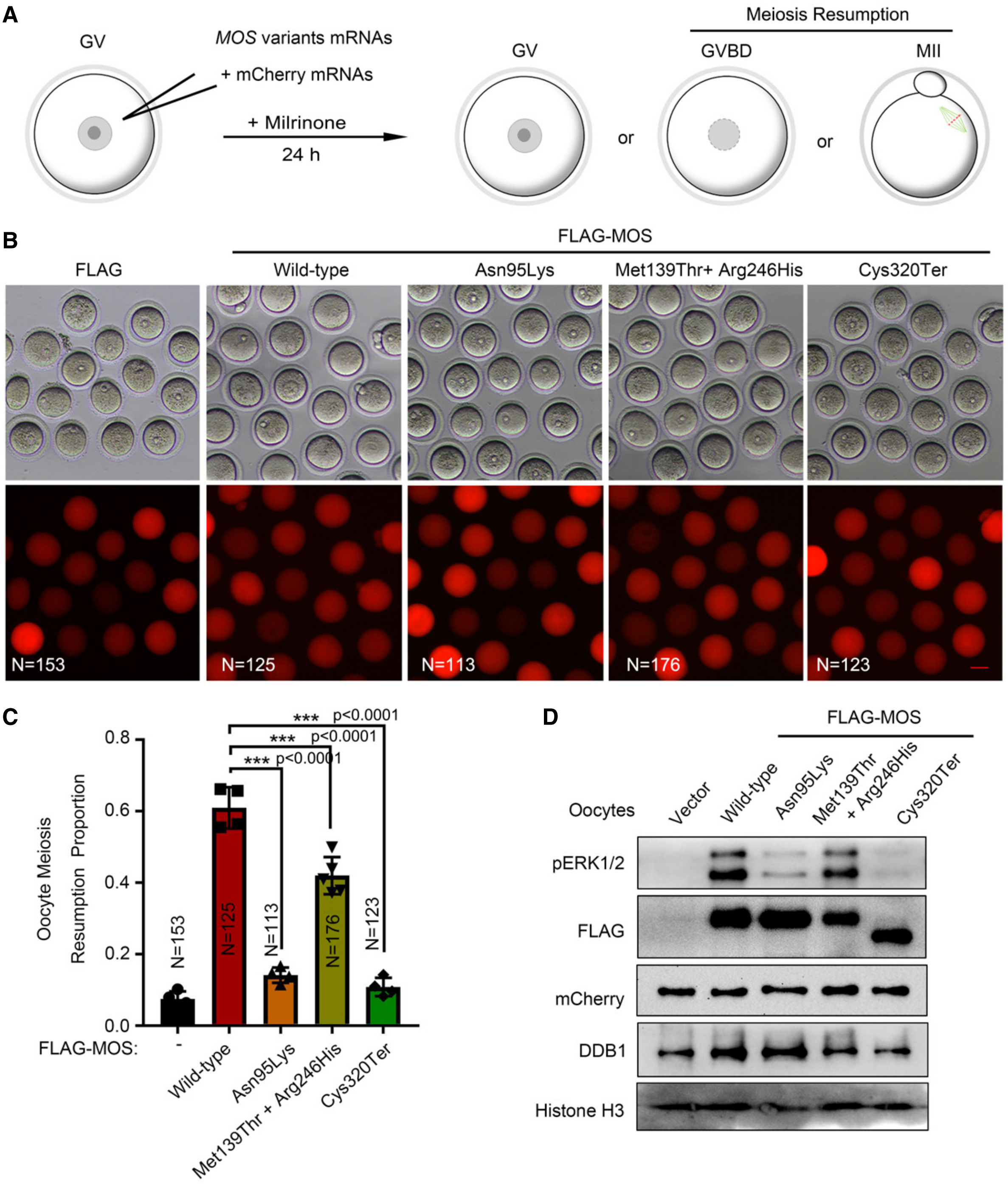

Figure EV1.

 © 2021 The Authors

Figure EV1. The effect of *MOS* variants on promoting oocyte meiosis maturation.

A The diagram showing the experimental procedure. Geminal vesical (GV) oocytes were microinjected with FLAG-tagged *MOS*, $MOS^{Asn95Lys}$, $MOS^{Met139Thr}$ and $MOS^{Arg246His}$, $MOS^{Cys320Ter}$, or FLAG mRNAs combined with mCherry mRNAs, and maintained in milrinone-treated M2 medium for 24 h. The number of GV oocytes, geminal vesical breakdown (GVBD), or metaphase II (MII) oocytes was counted.

B The representative bright-field images and fluorescent images were captured at 24 h after microinjection using a Nikon microscope. Over 100 oocytes in each group. Scale bar = 100 μm.

C The percentage of meiosis resumption (GVBD and MII) oocytes at 24 h after microinjection with different *MOS* mRNAs in (B). N number was labeled in each graph.

D Western blot analysis of FLAG, pERK1/2, and mCherry using oocytes after microinjection with different *MOS* mRNAs combined with mCherry mRNAs for 24 h. Histone H3 and DDB1 were used as loading controls. N = 100 oocytes per sample.

Data information: For C, data are expressed as mean ± SD. One-way ANOVA followed by *post hoc* Tukey's test for multiple comparisons, ***$P < 0.0001$.
Source data are available online for this figure.

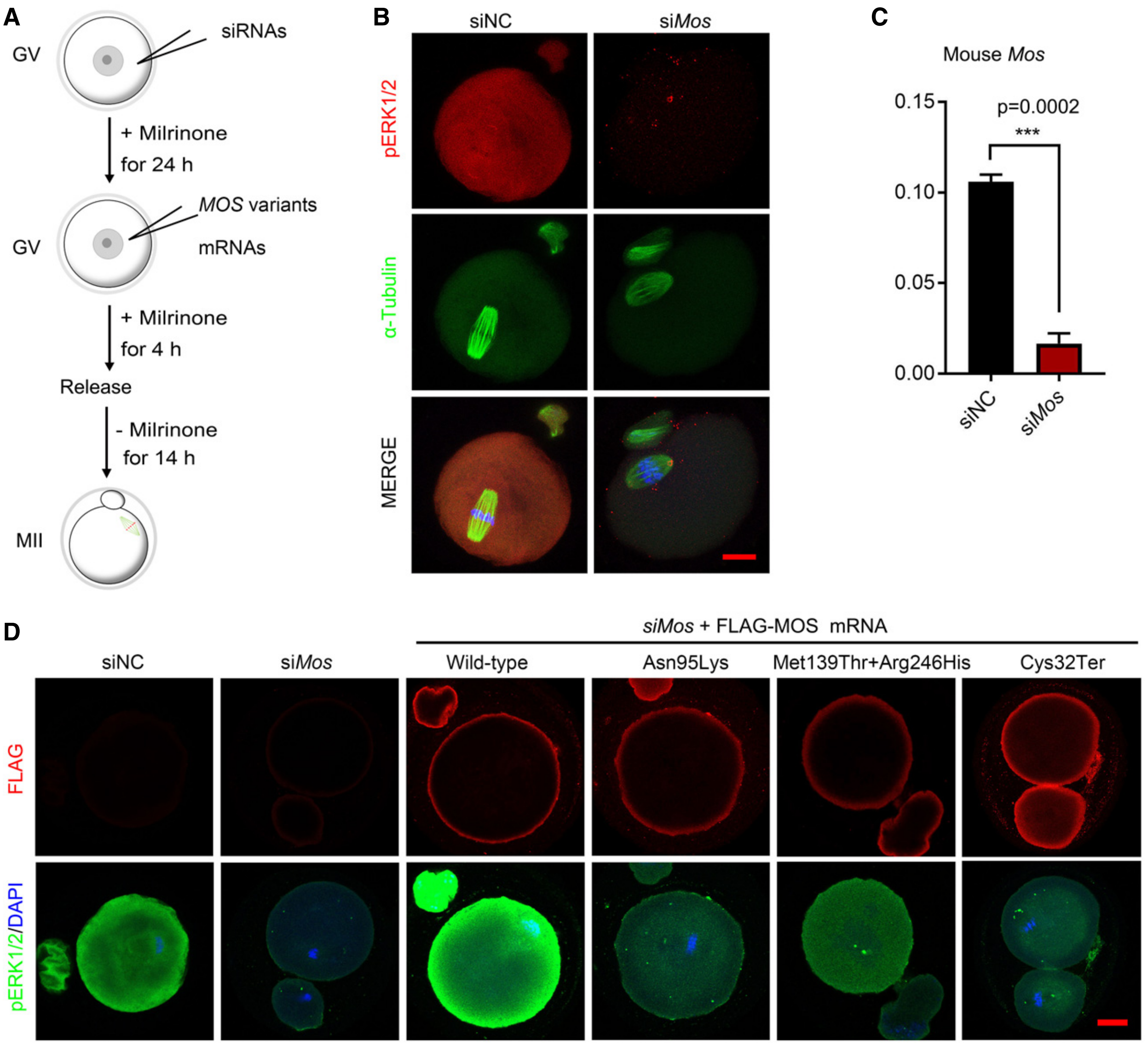

Figure EV2.

Figure EV2. The RNAi effect of *Mos* and the rescuing effects of different *MOS* variants in oocytes.

A Schematic diagram of oocyte cultures and experimental procedure. Mouse GV oocytes were microinjected with siRNAs and cultured for 24 h in the presence of 2.5 μM milrinone, followed by microinjection of *MOS* variants mRNAs, and cultured for 4 h before release to fresh M2 medium for another 14 h culture. Then, the RNAi efficiency of *siRNA* targeting mouse *Mos* and rescuing effects of different human *MOS* variants were determined.

B The representative images showing the decreased pERK1/2 (red) and abnormal spindle (FITC-α-tubulin, green) after *Mos* siRNA injection. DAPI (blue) was stained for visualization of DNA. Scale bar = 10 μm.

C RT-qPCR results showing the mouse *Mos* mRNA expression levels in oocytes with negative control siRNAs or *Mos* siRNAs ($n = 3$).

D Representative immunofluorescence images showing the FLAG (red) and pERK1/2 (green) levels in mature oocytes after microinjection of *siMos* or combined with indicated human *MOS* variants in GV oocytes. At least 30 oocytes each group were used for immunofluorescence. Scale bar = 10 μm.

Data information: In C, data are expressed as mean ± SD of three biological replicates. ***$P = 0.0002$ (unpaired two-tailed Student's *t*-test).
Source data are available online for this figure.

 © 2021 The Authors
