# Supplementary figures and images for: Biallelic mutations in MOS cause female infertility characterized by human early embryonic arrest and fragmentation (part 1 of 2)
Source: EMBO Mol Med. 2021 Nov 15;13(12):e14887. doi: 10.15252/emmm.202114887 (PMC8649871; doi:10.15252/emmm.202114887)

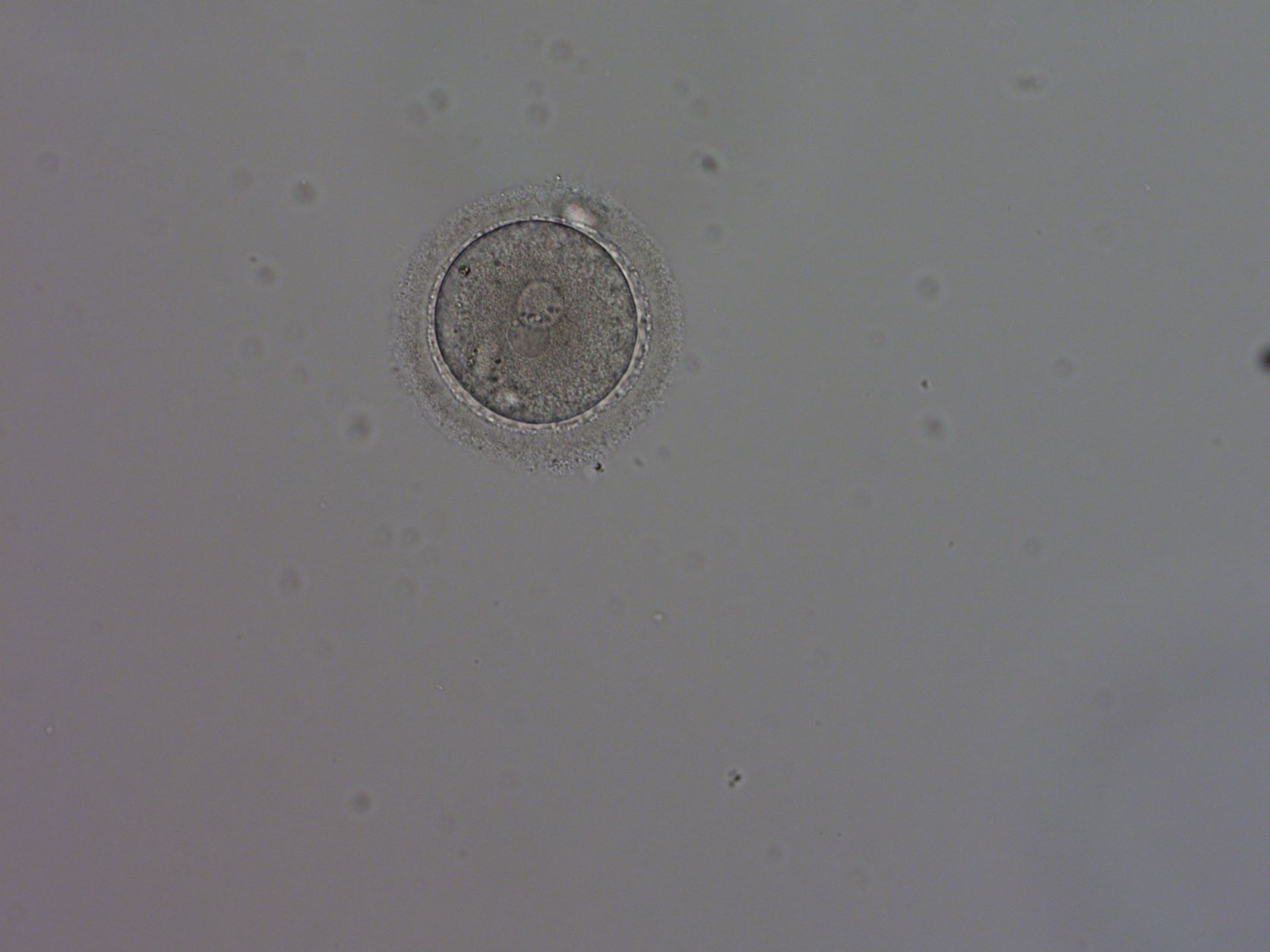

Supplement: Supplementary file 6 — Source Data for Figure 1 [file EMMM-13-e14887-s009.zip › EMM-2021-14887_SDataFig1/Control-Day1_Zygote.jpg]

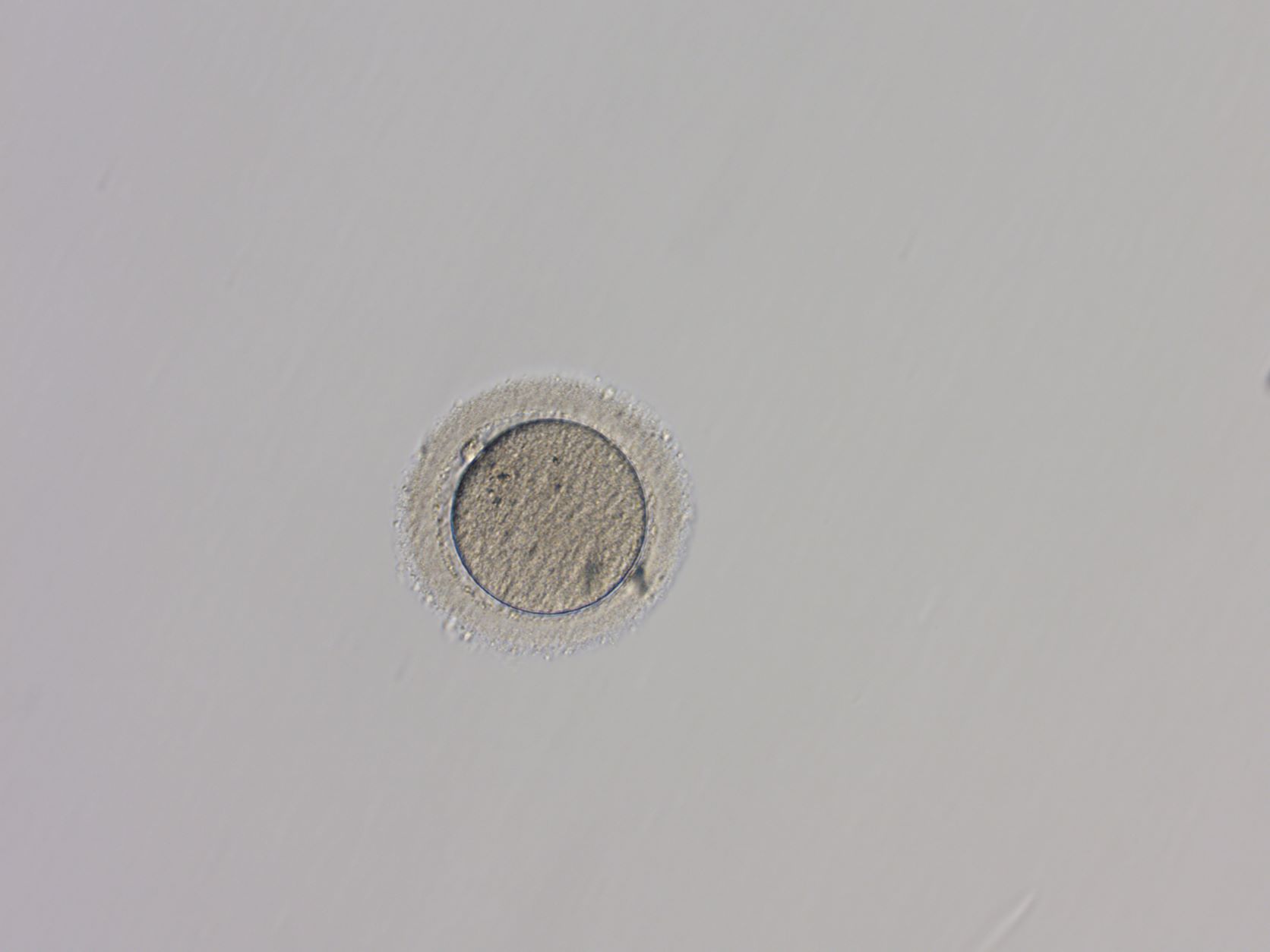

Supplement: Supplementary file 6 — Source Data for Figure 1 [file EMMM-13-e14887-s009.zip › EMM-2021-14887_SDataFig1/Control_Day0_MII.jpg]

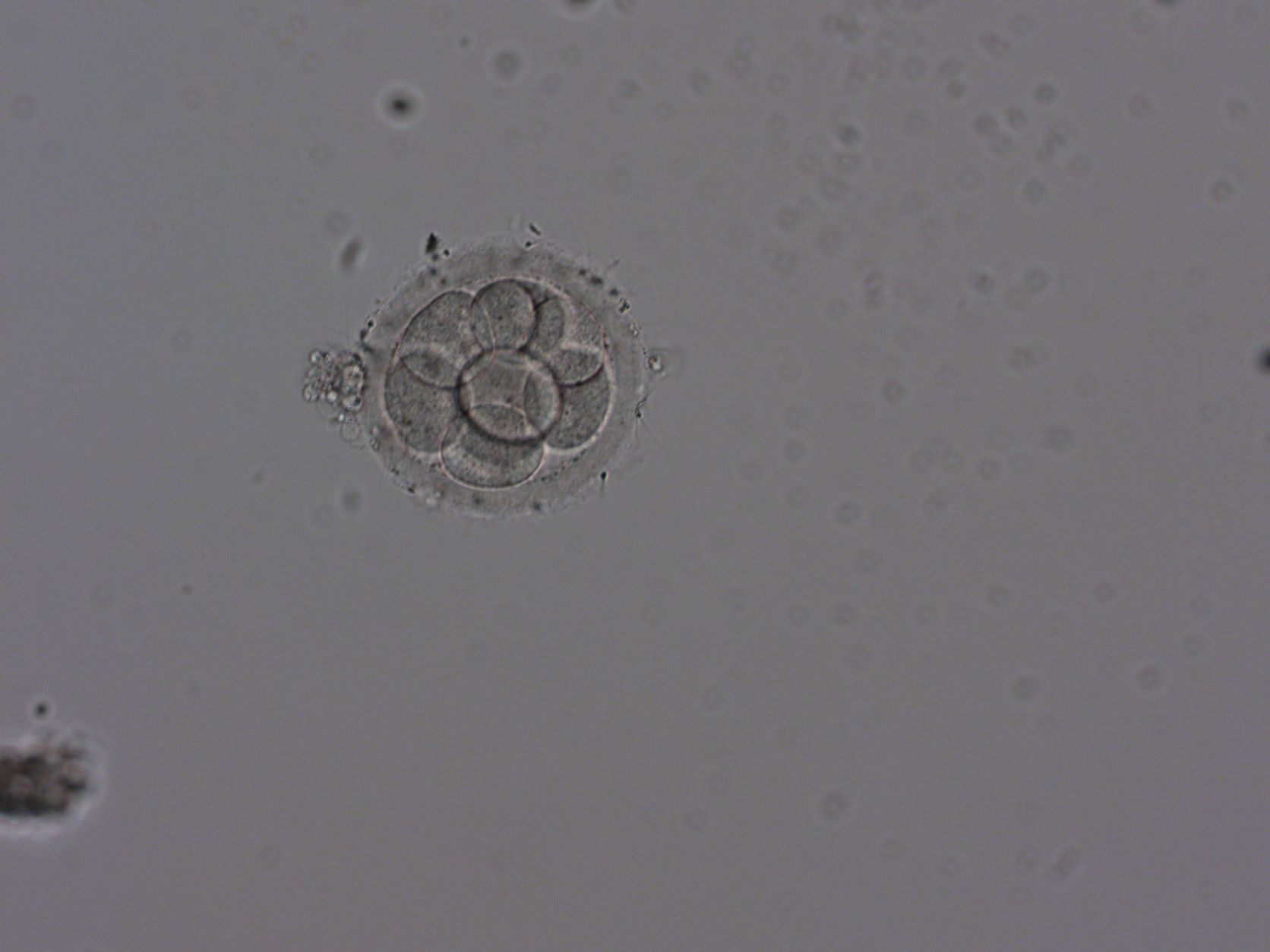

Supplement: Supplementary file 6 — Source Data for Figure 1 [file EMMM-13-e14887-s009.zip › EMM-2021-14887_SDataFig1/Control_Day3_8-cell.jpg]

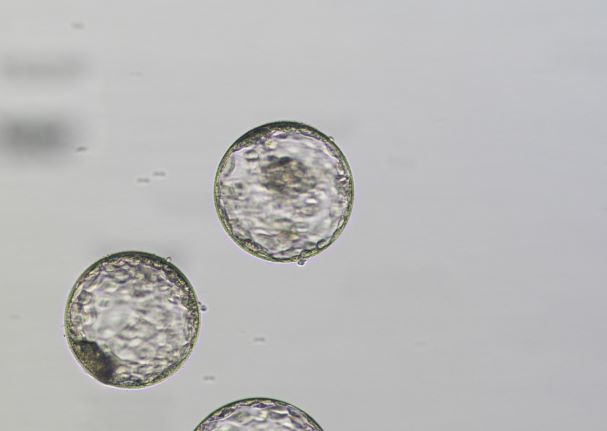

Supplement: Supplementary file 6 — Source Data for Figure 1 [file EMMM-13-e14887-s009.zip › EMM-2021-14887_SDataFig1/Control_Day5_Blastocyst.jpg]

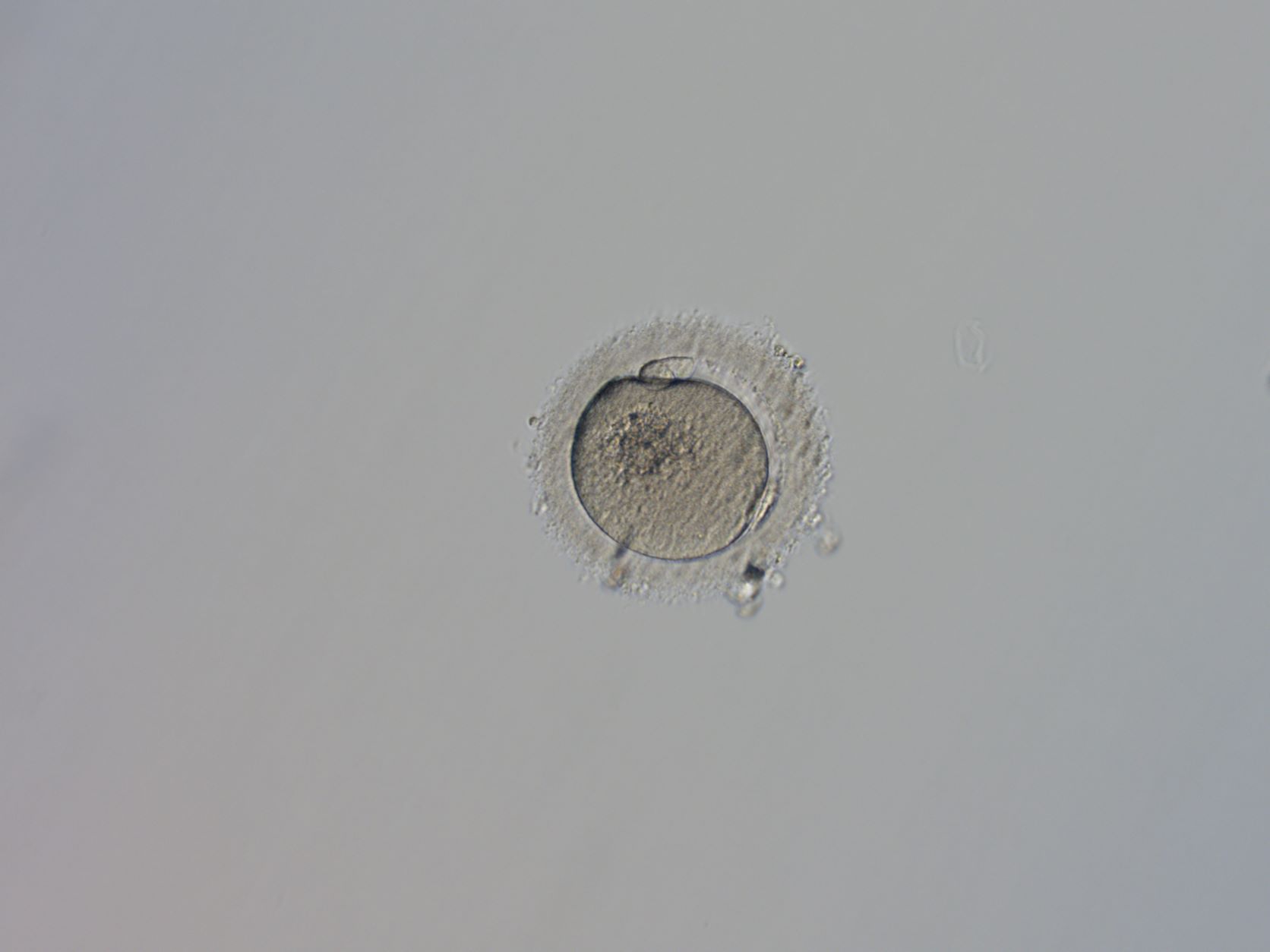

Supplement: Supplementary file 6 — Source Data for Figure 1 [file EMMM-13-e14887-s009.zip › EMM-2021-14887_SDataFig1/Family 1_Asn95Lys_Day0_MII_1.jpg]

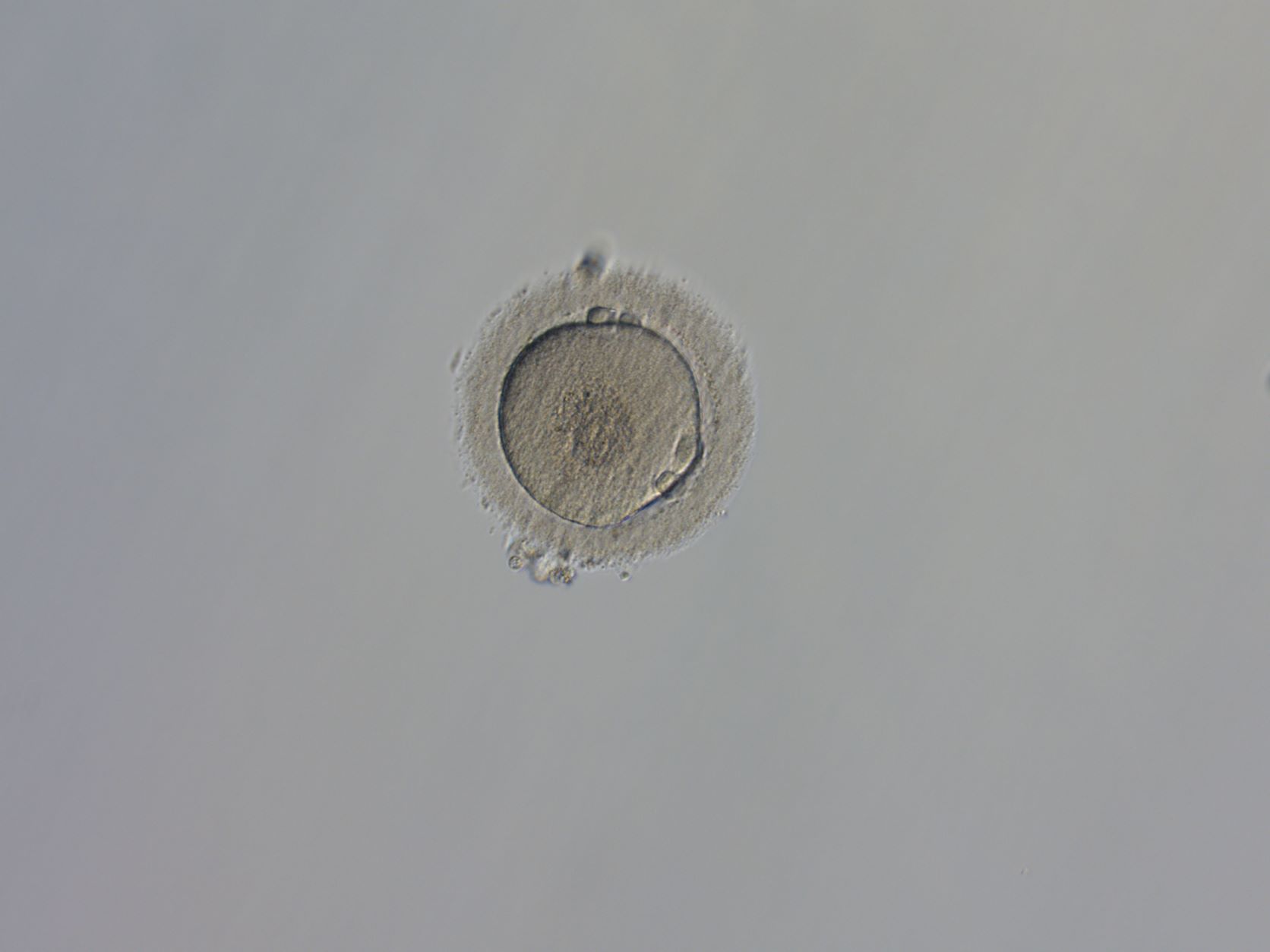

Supplement: Supplementary file 6 — Source Data for Figure 1 [file EMMM-13-e14887-s009.zip › EMM-2021-14887_SDataFig1/Family 1_Asn95Lys_Day0_MII_2.jpg]

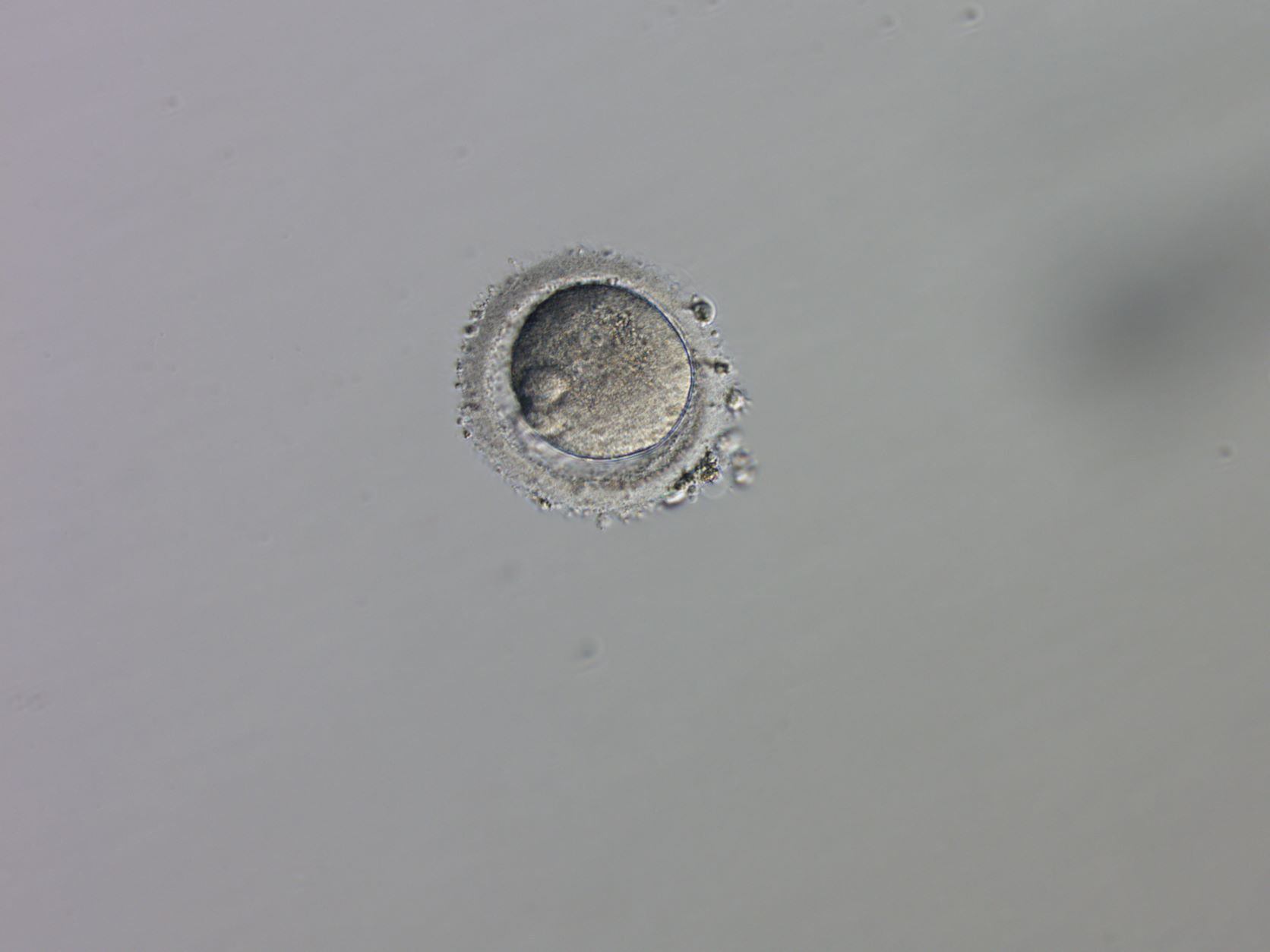

Supplement: Supplementary file 6 — Source Data for Figure 1 [file EMMM-13-e14887-s009.zip › EMM-2021-14887_SDataFig1/Family 1_Asn95Lys_Day1_Zygote_1.jpg]

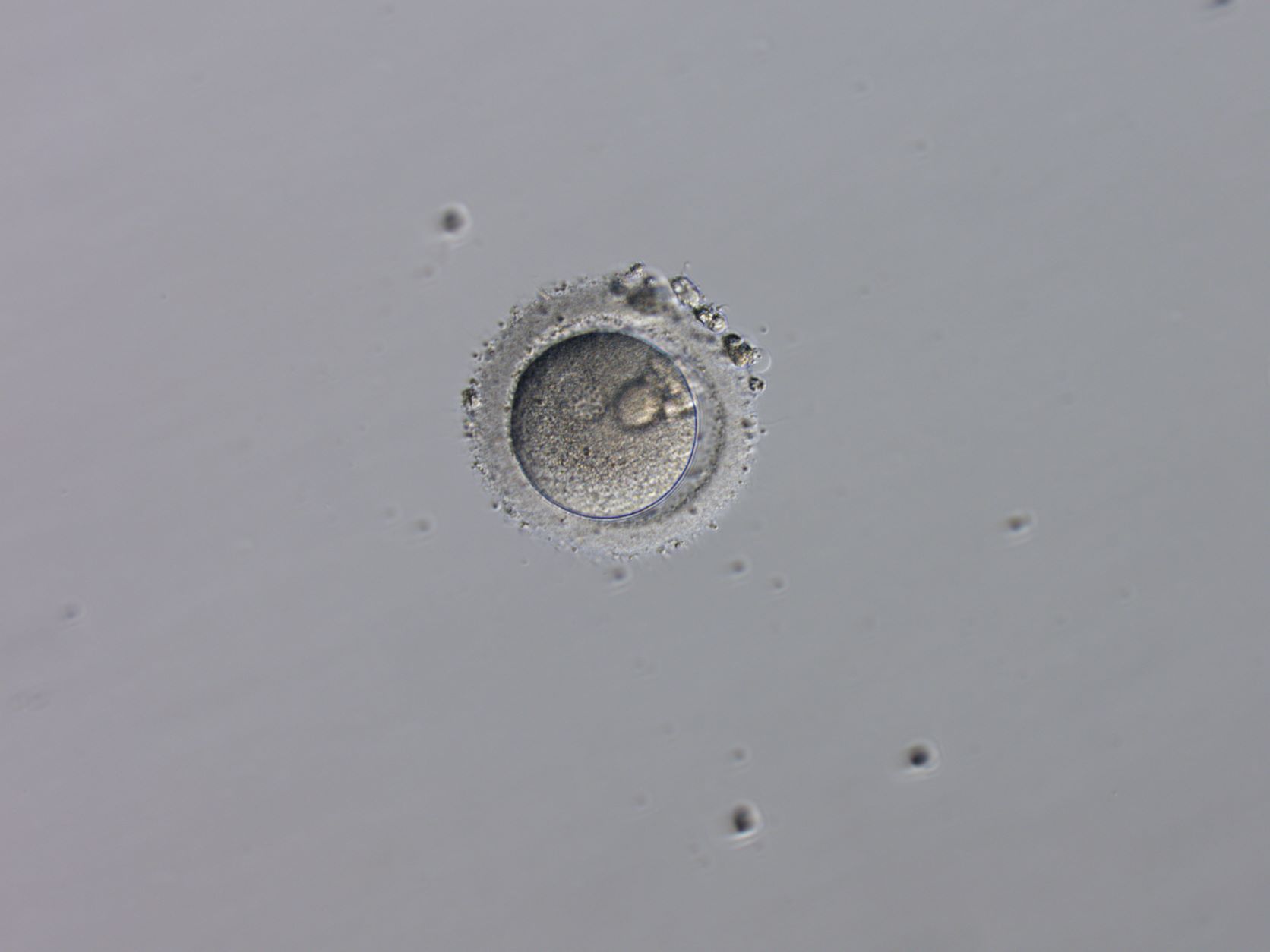

Supplement: Supplementary file 6 — Source Data for Figure 1 [file EMMM-13-e14887-s009.zip › EMM-2021-14887_SDataFig1/Family 1_Asn95Lys_Day1_Zygote_2.jpg]

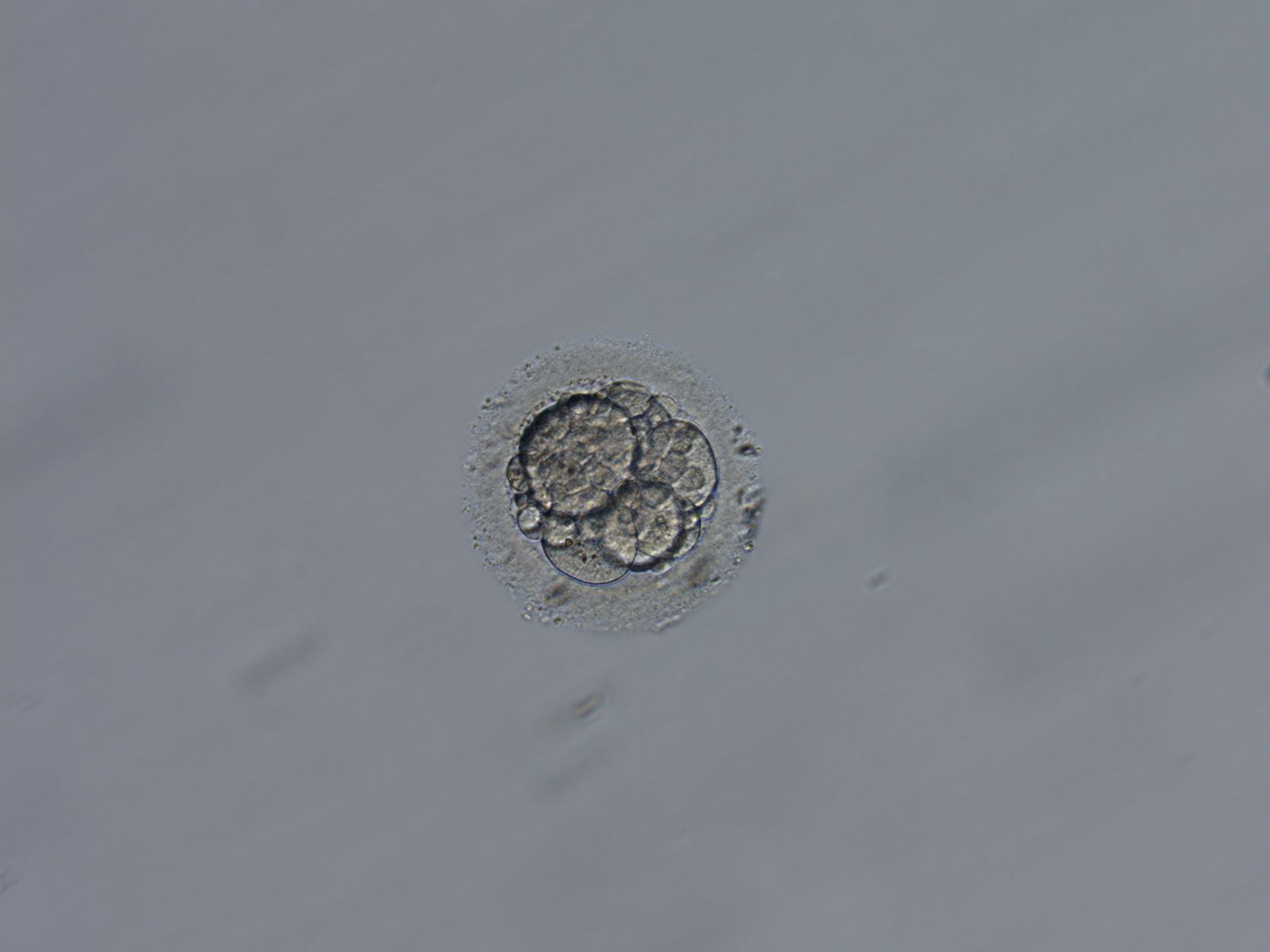

Supplement: Supplementary file 6 — Source Data for Figure 1 [file EMMM-13-e14887-s009.zip › EMM-2021-14887_SDataFig1/Family 1_Asn95Lys_Day3_embryo_1.jpg]

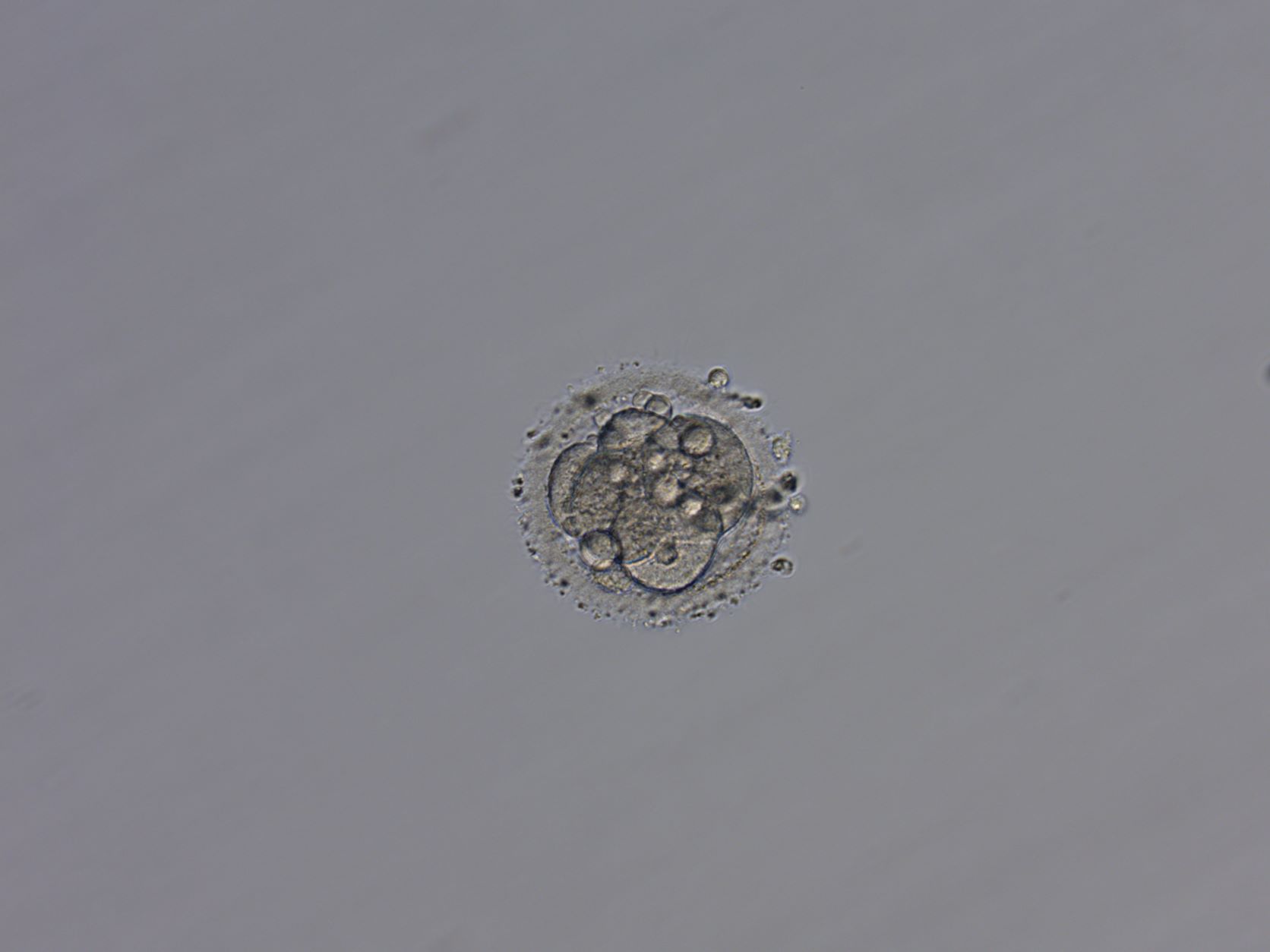

Supplement: Supplementary file 6 — Source Data for Figure 1 [file EMMM-13-e14887-s009.zip › EMM-2021-14887_SDataFig1/Family 1_Asn95Lys_Day3_embryo_2.jpg]

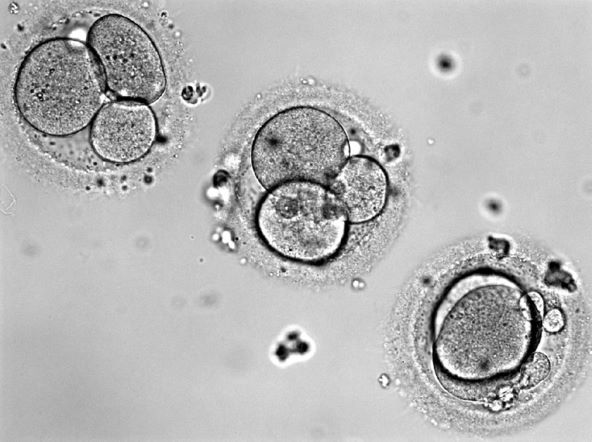

Supplement: Supplementary file 6 — Source Data for Figure 1 [file EMMM-13-e14887-s009.zip › EMM-2021-14887_SDataFig1/Family 3 C320Ter_Day3_Embryo.jpg]

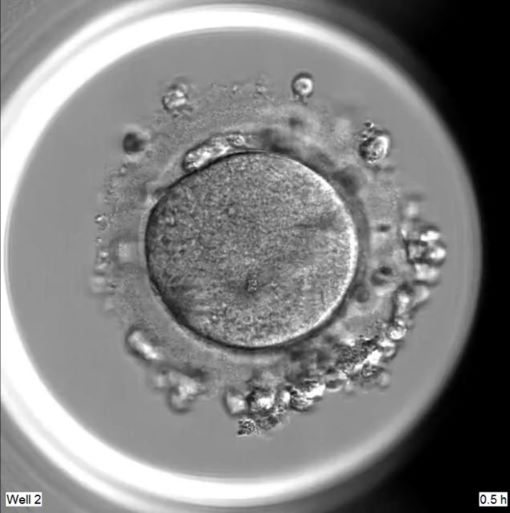

Supplement: Supplementary file 6 — Source Data for Figure 1 [file EMMM-13-e14887-s009.zip › EMM-2021-14887_SDataFig1/Family2_Day0_MII.jpg]

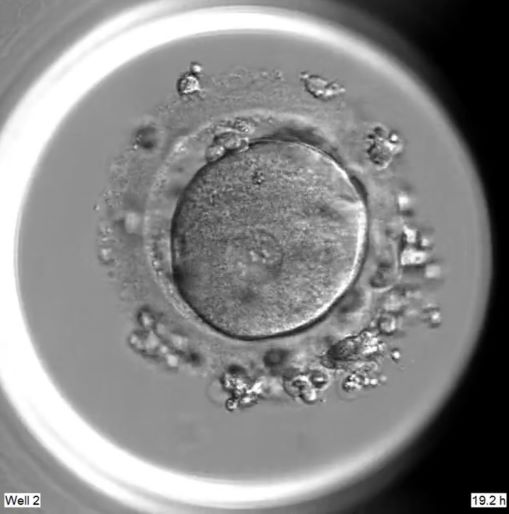

Supplement: Supplementary file 6 — Source Data for Figure 1 [file EMMM-13-e14887-s009.zip › EMM-2021-14887_SDataFig1/Family2_Day1_zygote.jpg]

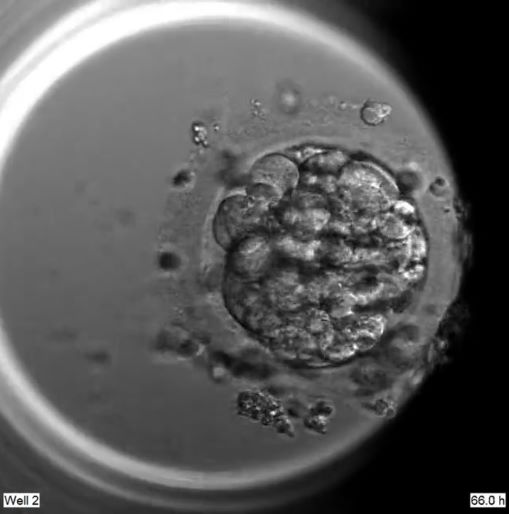

Supplement: Supplementary file 6 — Source Data for Figure 1 [file EMMM-13-e14887-s009.zip › EMM-2021-14887_SDataFig1/Family2_Day3_embryo.jpg]

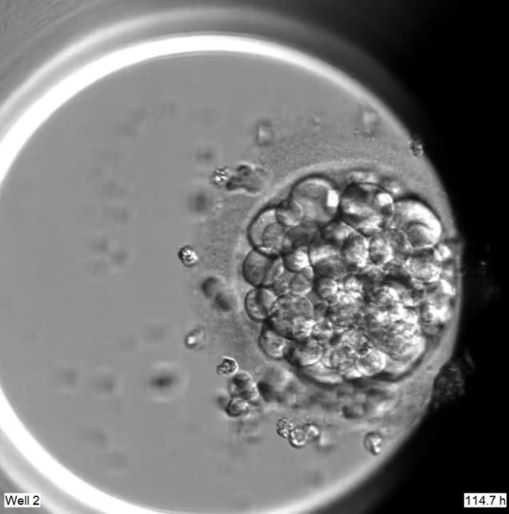

Supplement: Supplementary file 6 — Source Data for Figure 1 [file EMMM-13-e14887-s009.zip › EMM-2021-14887_SDataFig1/Family2_Day5_embryo.jpg]

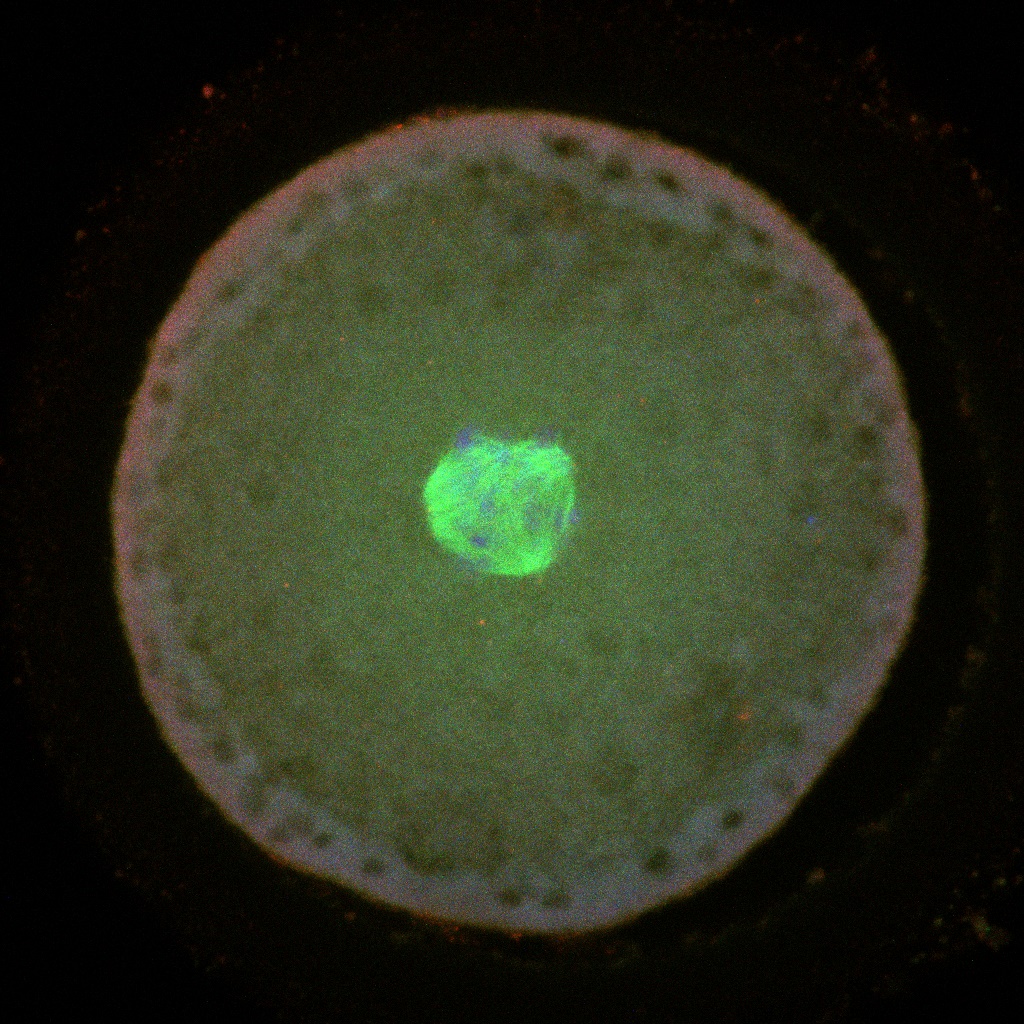

Supplement: Supplementary file 7 — Source Data for Figure 2 [file EMMM-13-e14887-s001.zip › EMM-2021-14887_SDataFig2/Fig. 2B Images/Mos-N95K-pERK12-MII-p-Image Export-17_c1-3.jpg]

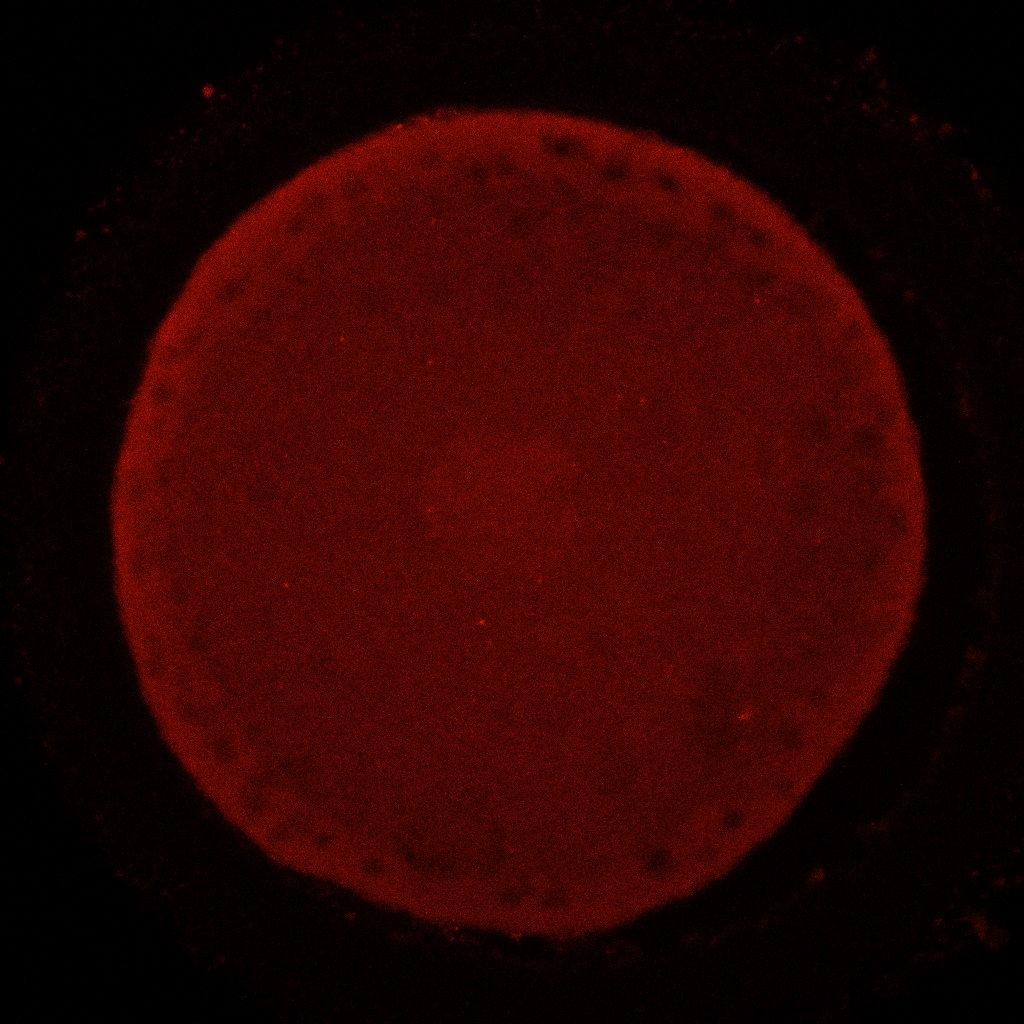

Supplement: Supplementary file 7 — Source Data for Figure 2 [file EMMM-13-e14887-s001.zip › EMM-2021-14887_SDataFig2/Fig. 2B Images/Mos-N95K-pERK12-MII-p-Image Export-17_c1.jpg]

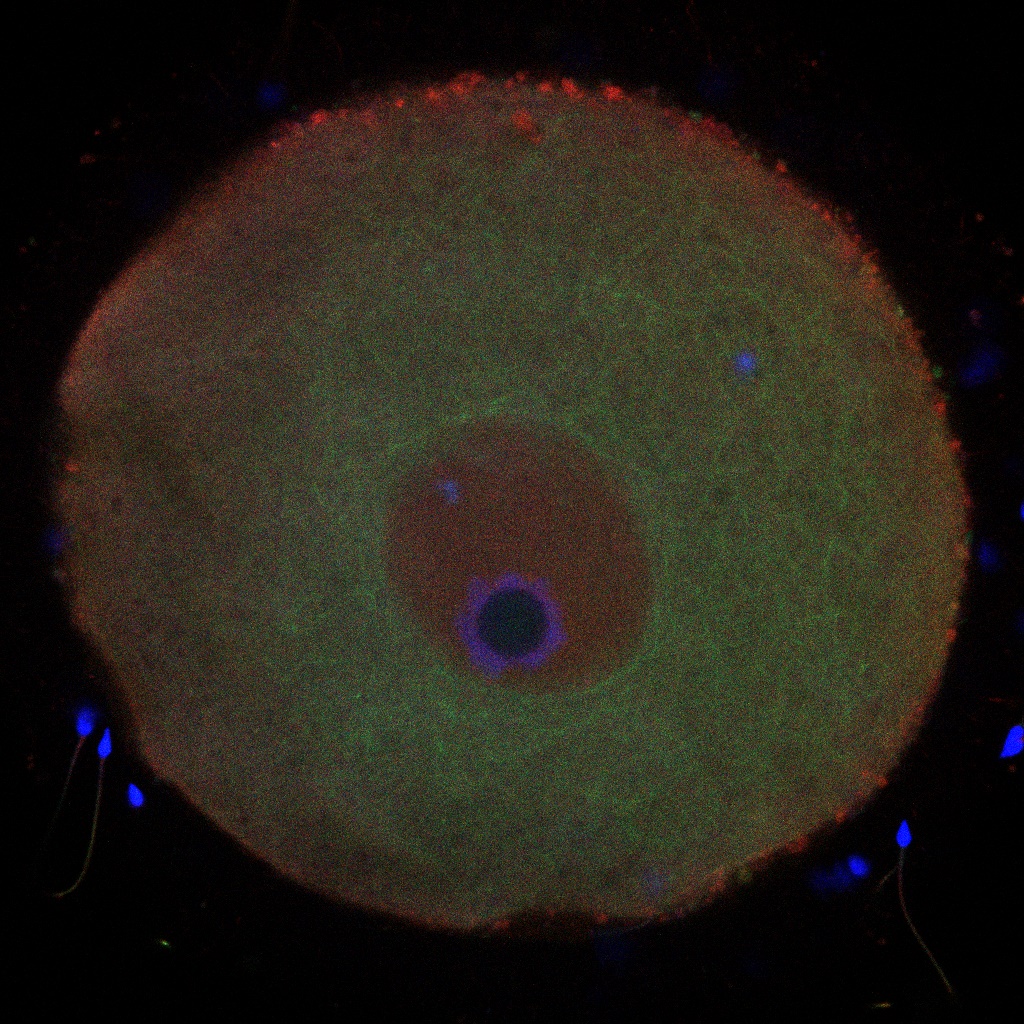

Supplement: Supplementary file 7 — Source Data for Figure 2 [file EMMM-13-e14887-s001.zip › EMM-2021-14887_SDataFig2/Fig. 2B Images/pERK12-GV-2-Image Export-22_c1-3.jpg]

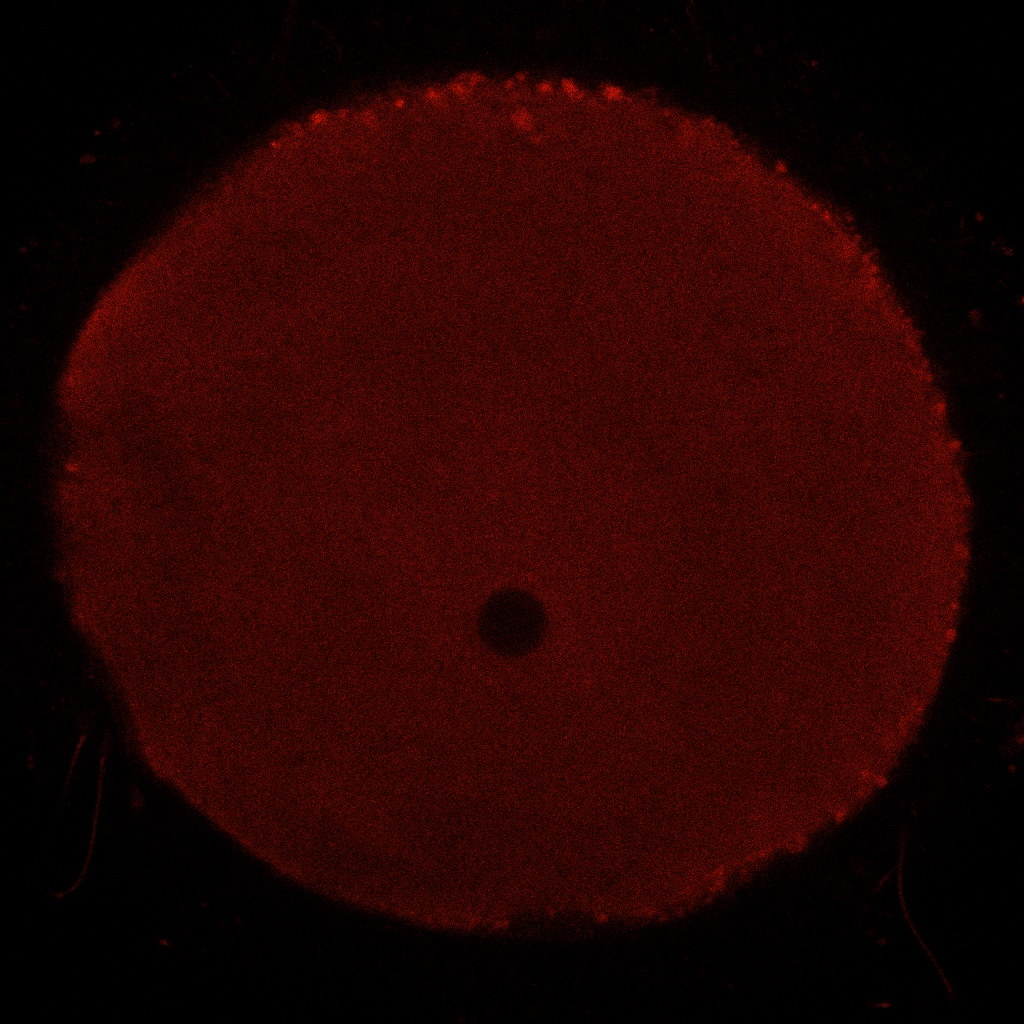

Supplement: Supplementary file 7 — Source Data for Figure 2 [file EMMM-13-e14887-s001.zip › EMM-2021-14887_SDataFig2/Fig. 2B Images/pERK12-GV-2-Image Export-22_c1.jpg]

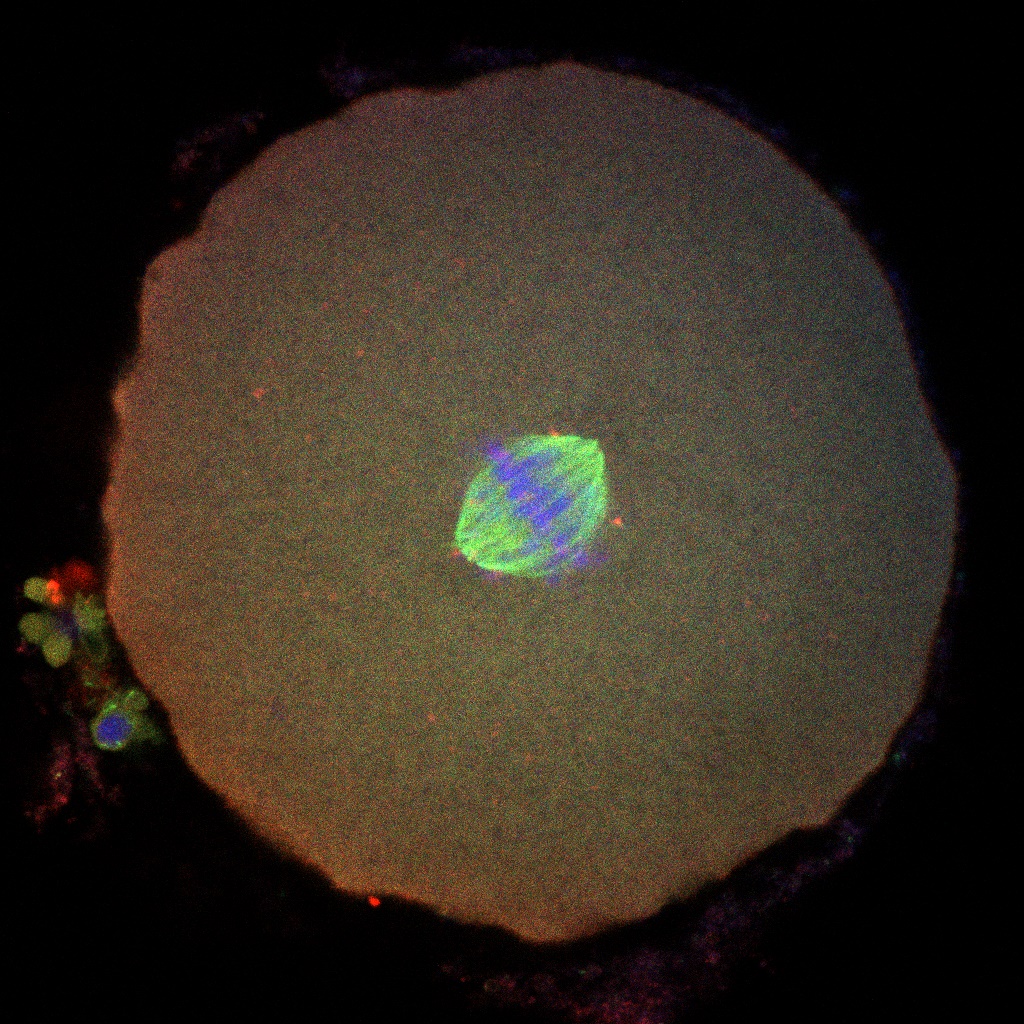

Supplement: Supplementary file 7 — Source Data for Figure 2 [file EMMM-13-e14887-s001.zip › EMM-2021-14887_SDataFig2/Fig. 2B Images/pERK12-MI-2-Image Export-24_c1-3.jpg]

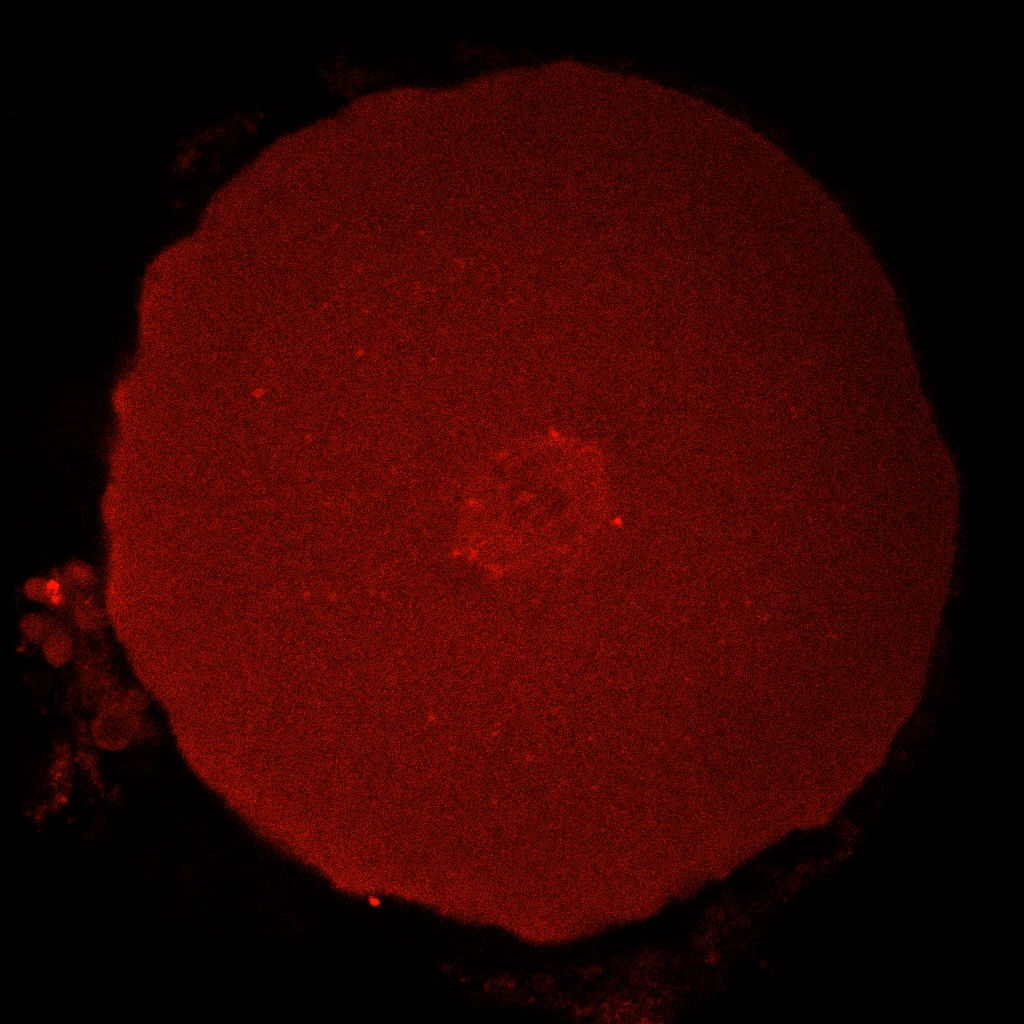

Supplement: Supplementary file 7 — Source Data for Figure 2 [file EMMM-13-e14887-s001.zip › EMM-2021-14887_SDataFig2/Fig. 2B Images/pERK12-MI-2-Image Export-24_c1.jpg]

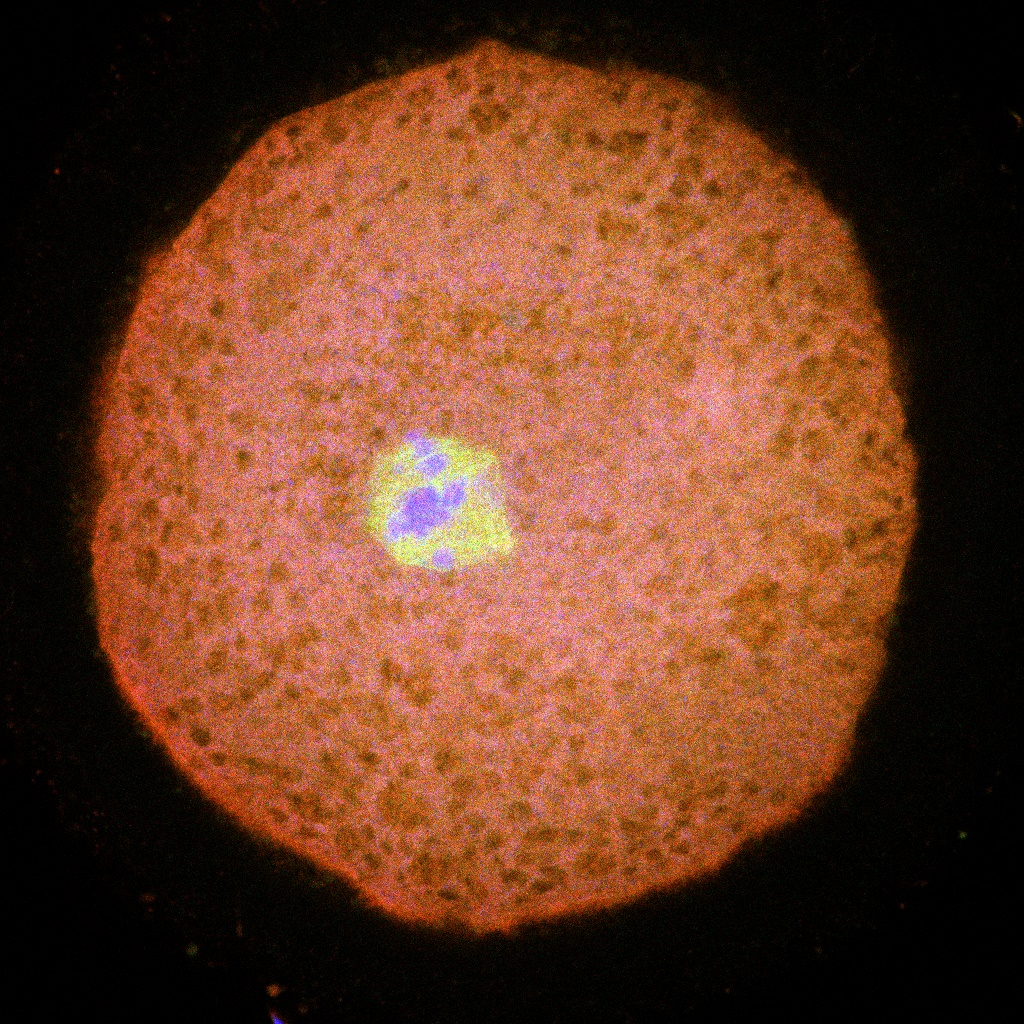

Supplement: Supplementary file 7 — Source Data for Figure 2 [file EMMM-13-e14887-s001.zip › EMM-2021-14887_SDataFig2/Fig. 2B Images/pERK12-MII-5-Image Export-30_c1-3.jpg]

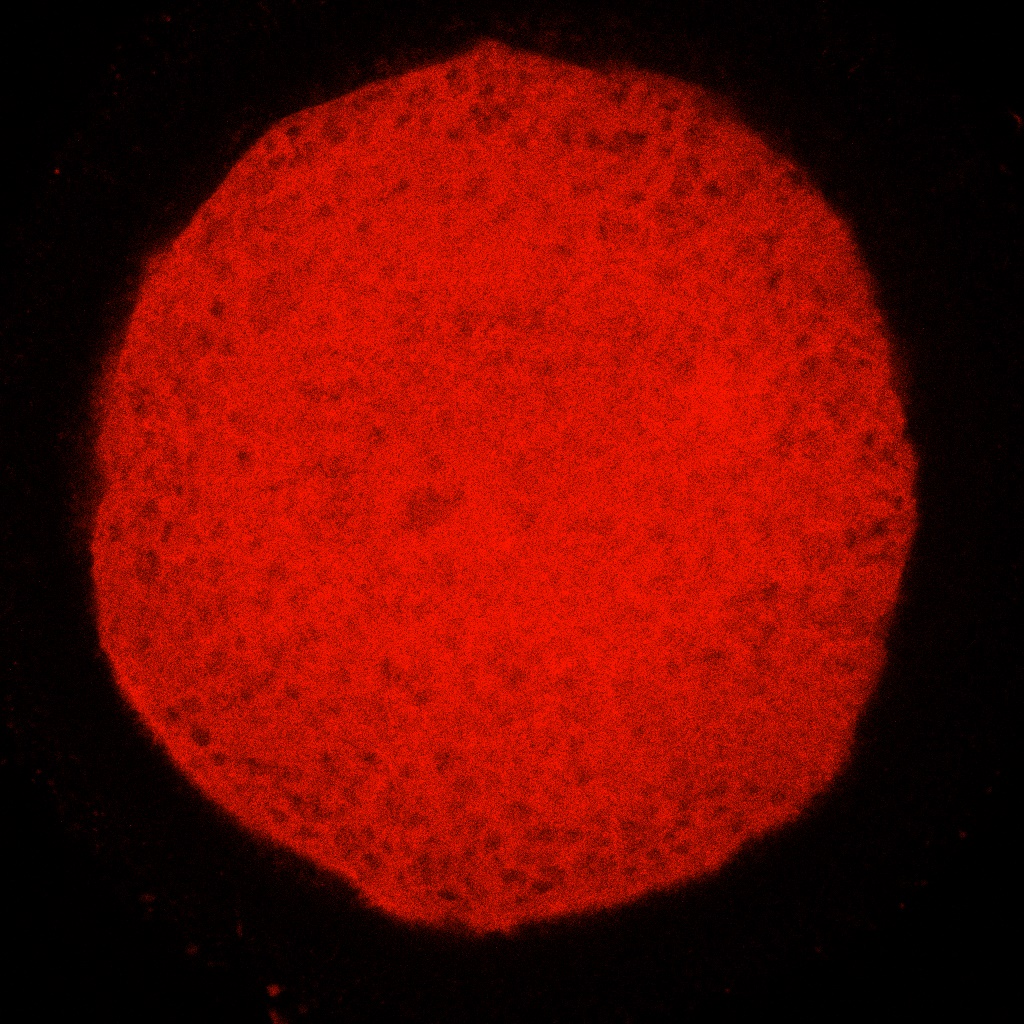

Supplement: Supplementary file 7 — Source Data for Figure 2 [file EMMM-13-e14887-s001.zip › EMM-2021-14887_SDataFig2/Fig. 2B Images/pERK12-MII-5-Image Export-30_c1.jpg]

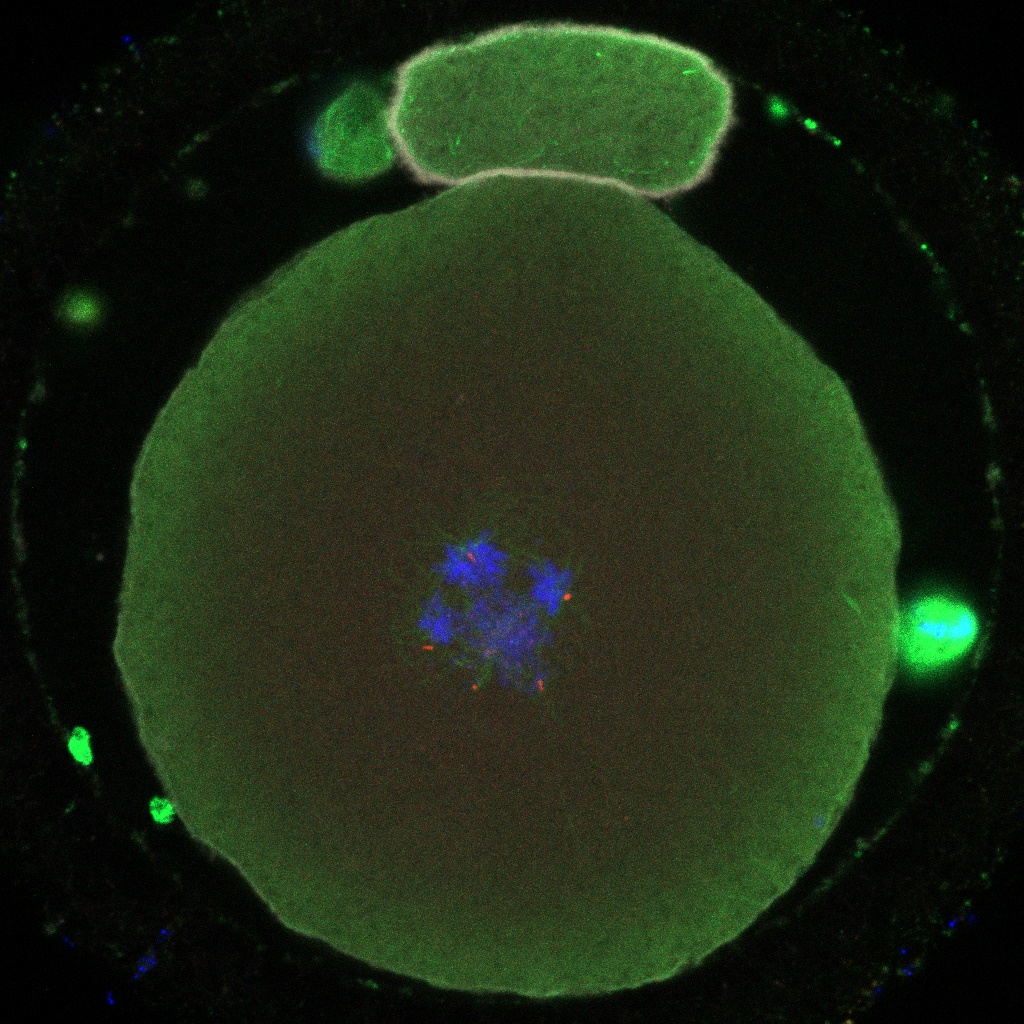

Supplement: Supplementary file 7 — Source Data for Figure 2 [file EMMM-13-e14887-s001.zip › EMM-2021-14887_SDataFig2/Fig. 2B Images/pERK12-u0126-Image Export-28_c1-3.jpg]

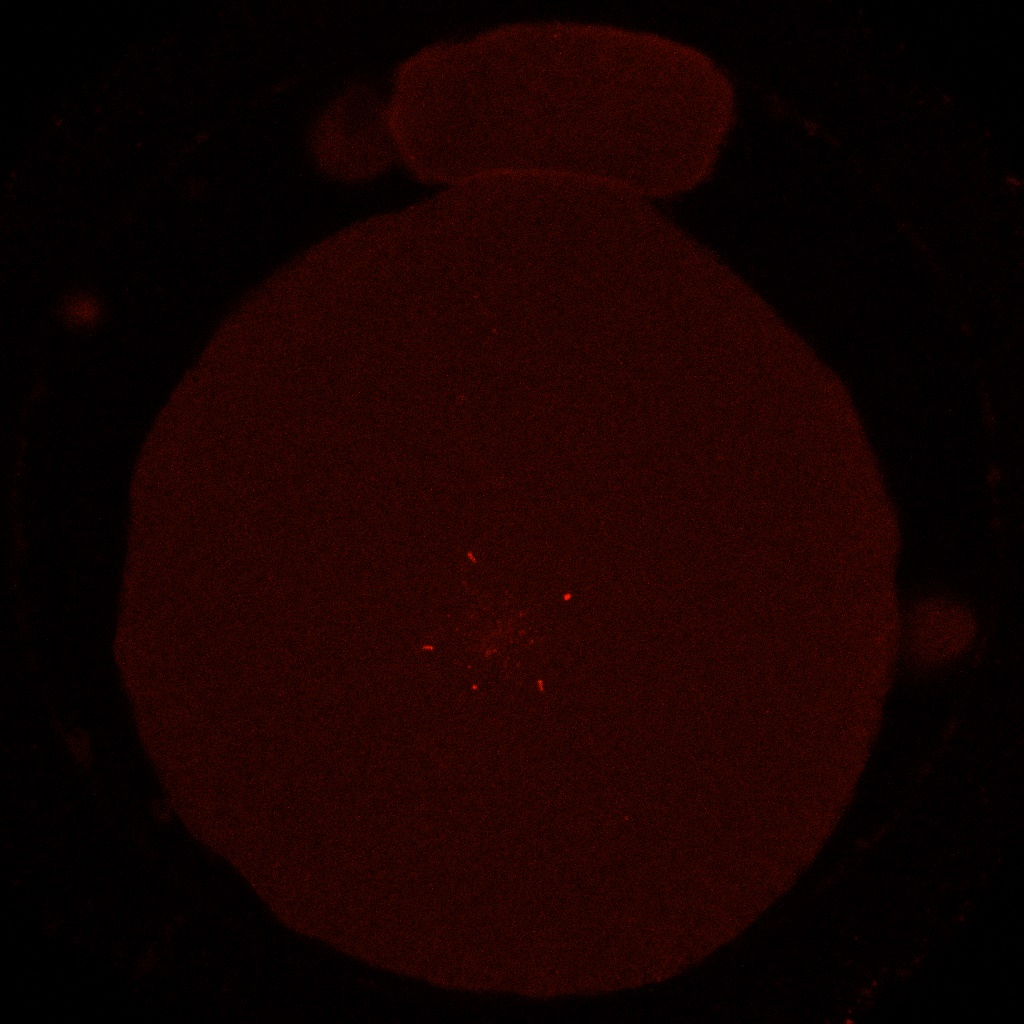

Supplement: Supplementary file 7 — Source Data for Figure 2 [file EMMM-13-e14887-s001.zip › EMM-2021-14887_SDataFig2/Fig. 2B Images/pERK12-u0126-Image Export-28_c1.jpg]

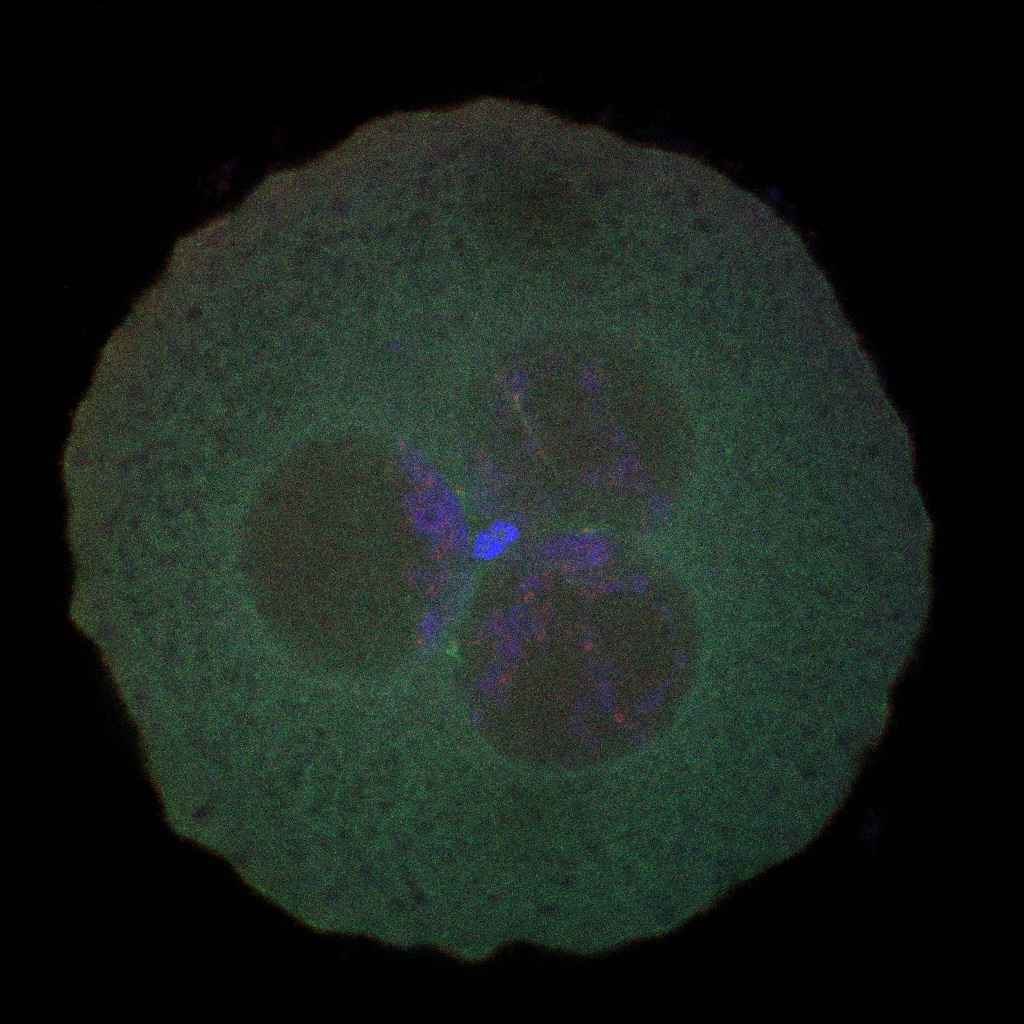

Supplement: Supplementary file 7 — Source Data for Figure 2 [file EMMM-13-e14887-s001.zip › EMM-2021-14887_SDataFig2/Fig. 2B Images/pERK12-zygote-2-Image Export-33_c1-3.jpg]

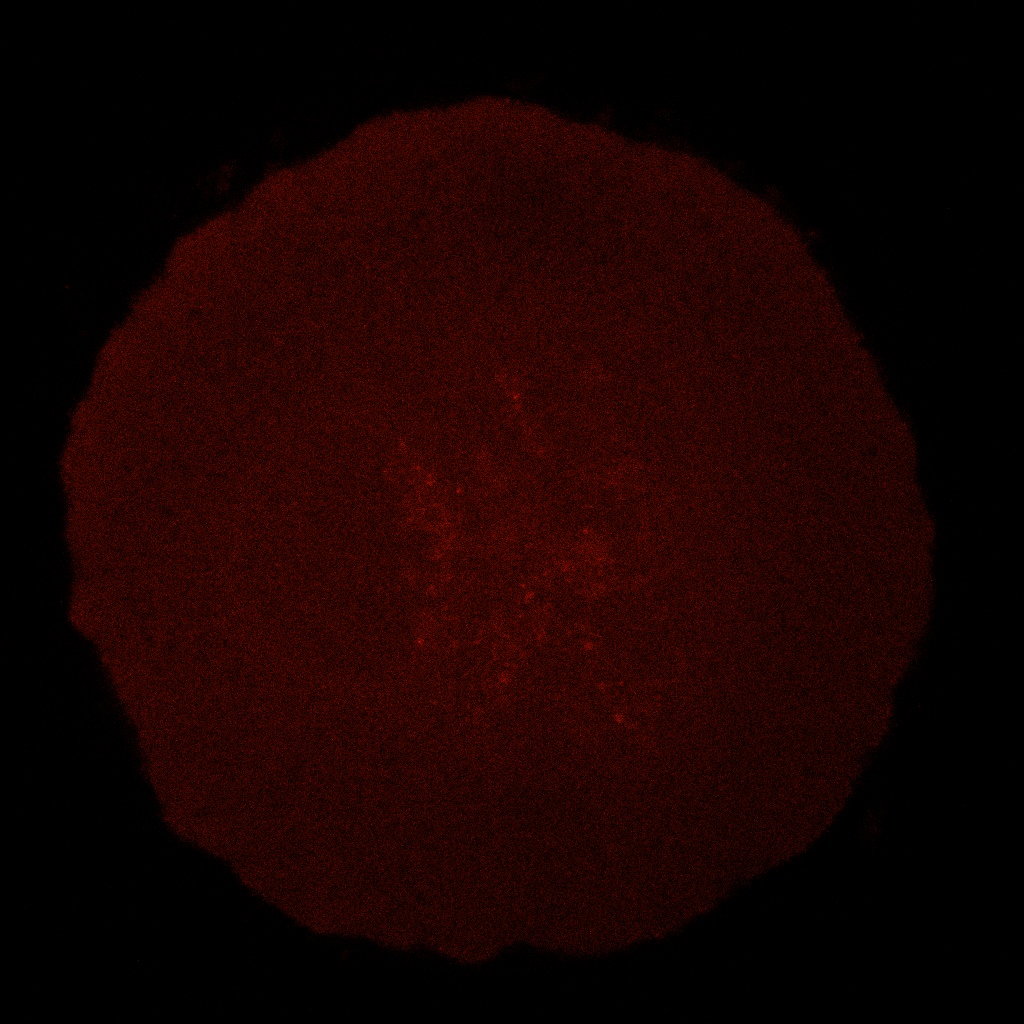

Supplement: Supplementary file 7 — Source Data for Figure 2 [file EMMM-13-e14887-s001.zip › EMM-2021-14887_SDataFig2/Fig. 2B Images/pERK12-zygote-2-Image Export-33_c1.jpg]

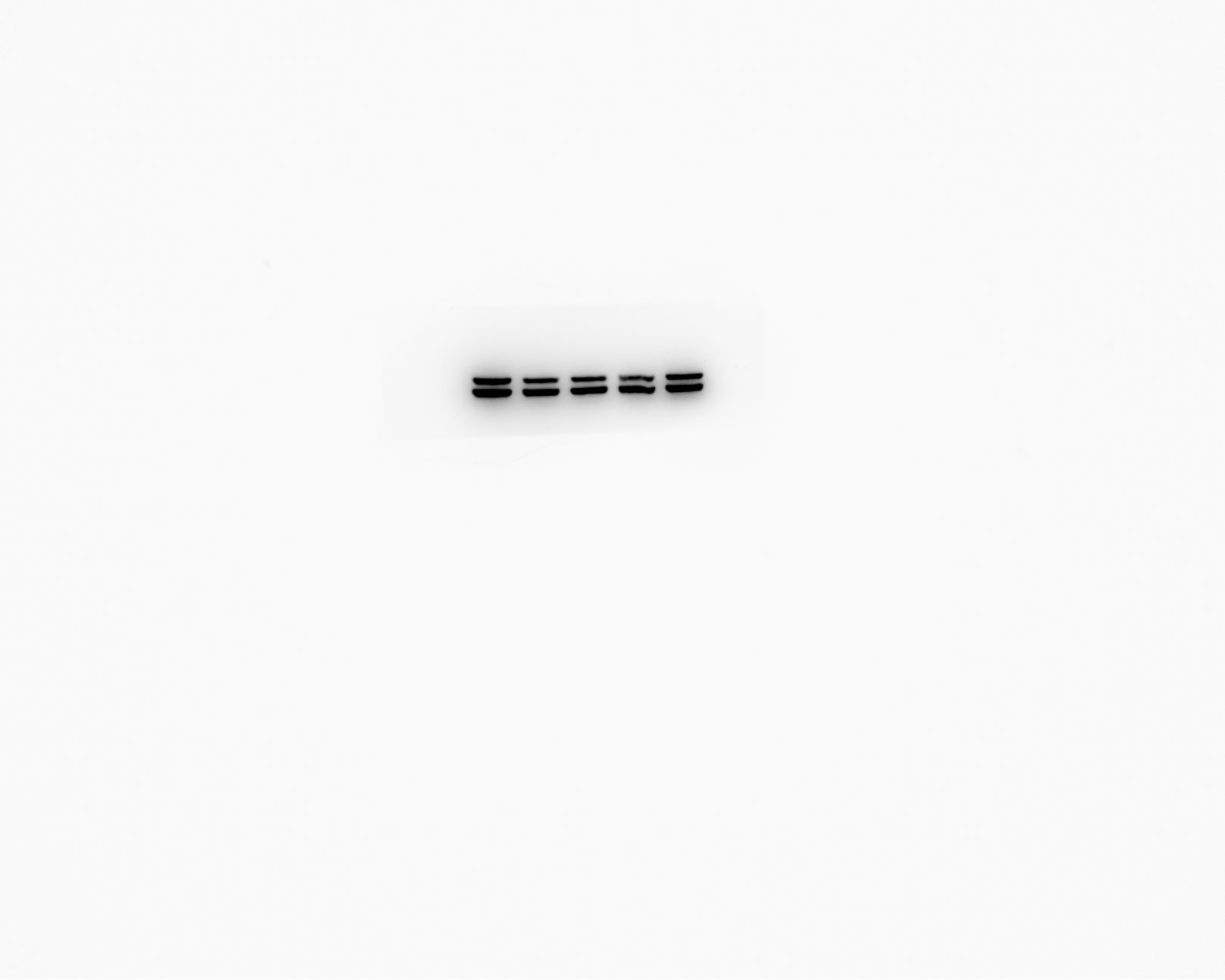

Supplement: Supplementary file 7 — Source Data for Figure 2 [file EMMM-13-e14887-s001.zip › EMM-2021-14887_SDataFig2/Fig. 2C_D WB and Intensity/293T_ERK12.jpg]

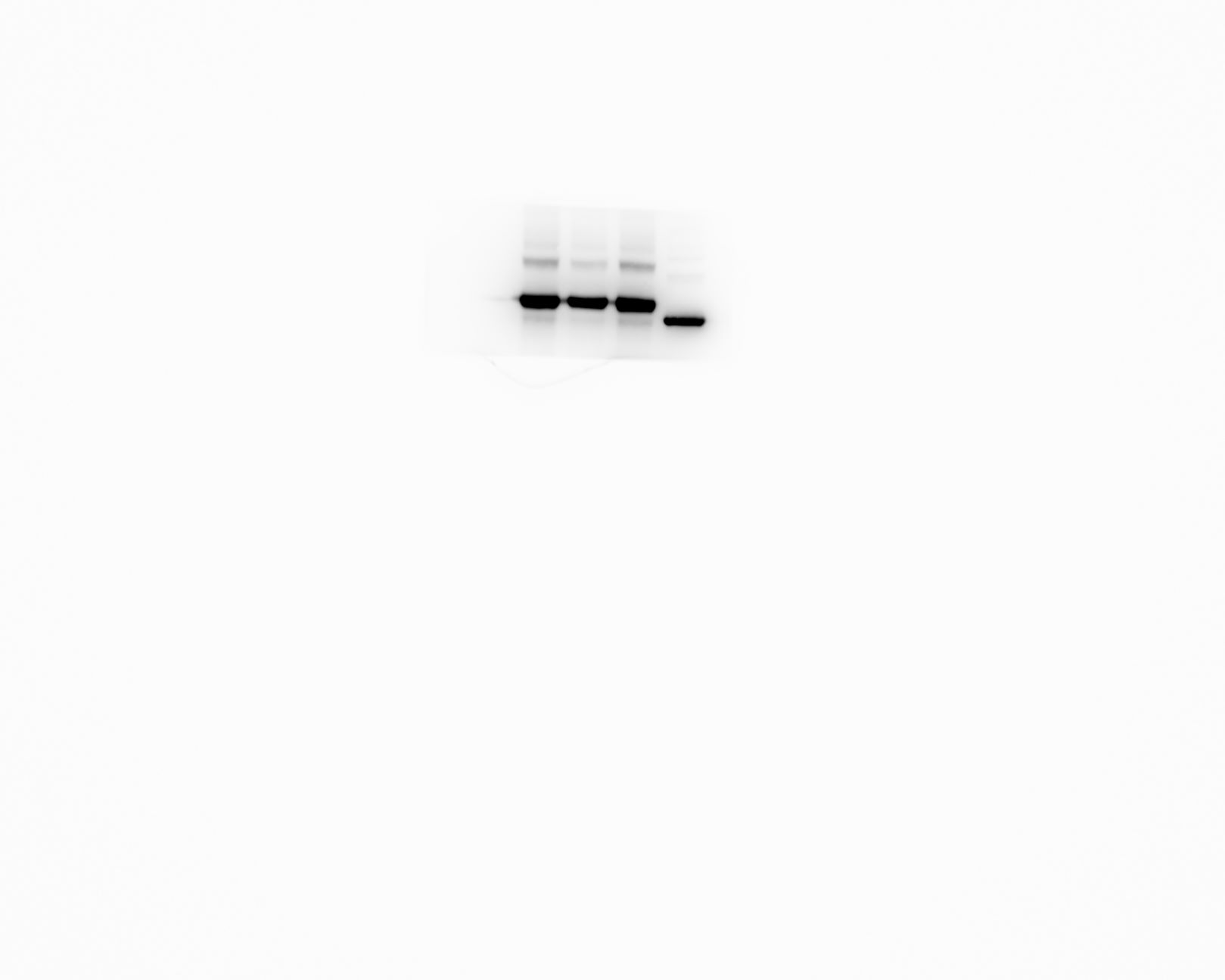

Supplement: Supplementary file 7 — Source Data for Figure 2 [file EMMM-13-e14887-s001.zip › EMM-2021-14887_SDataFig2/Fig. 2C_D WB and Intensity/293T_FLAG.jpg]

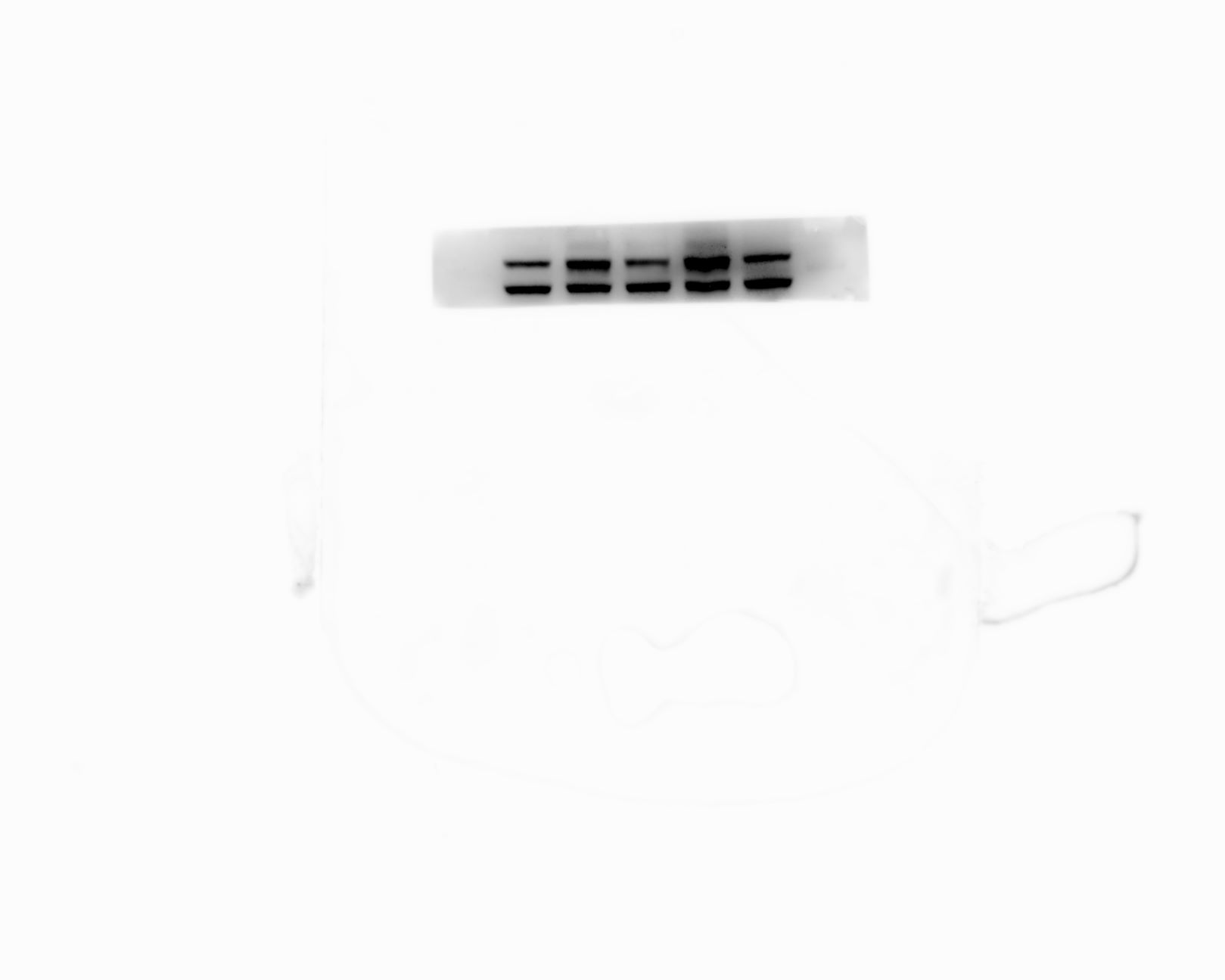

Supplement: Supplementary file 7 — Source Data for Figure 2 [file EMMM-13-e14887-s001.zip › EMM-2021-14887_SDataFig2/Fig. 2C_D WB and Intensity/293T_MEK.jpg]

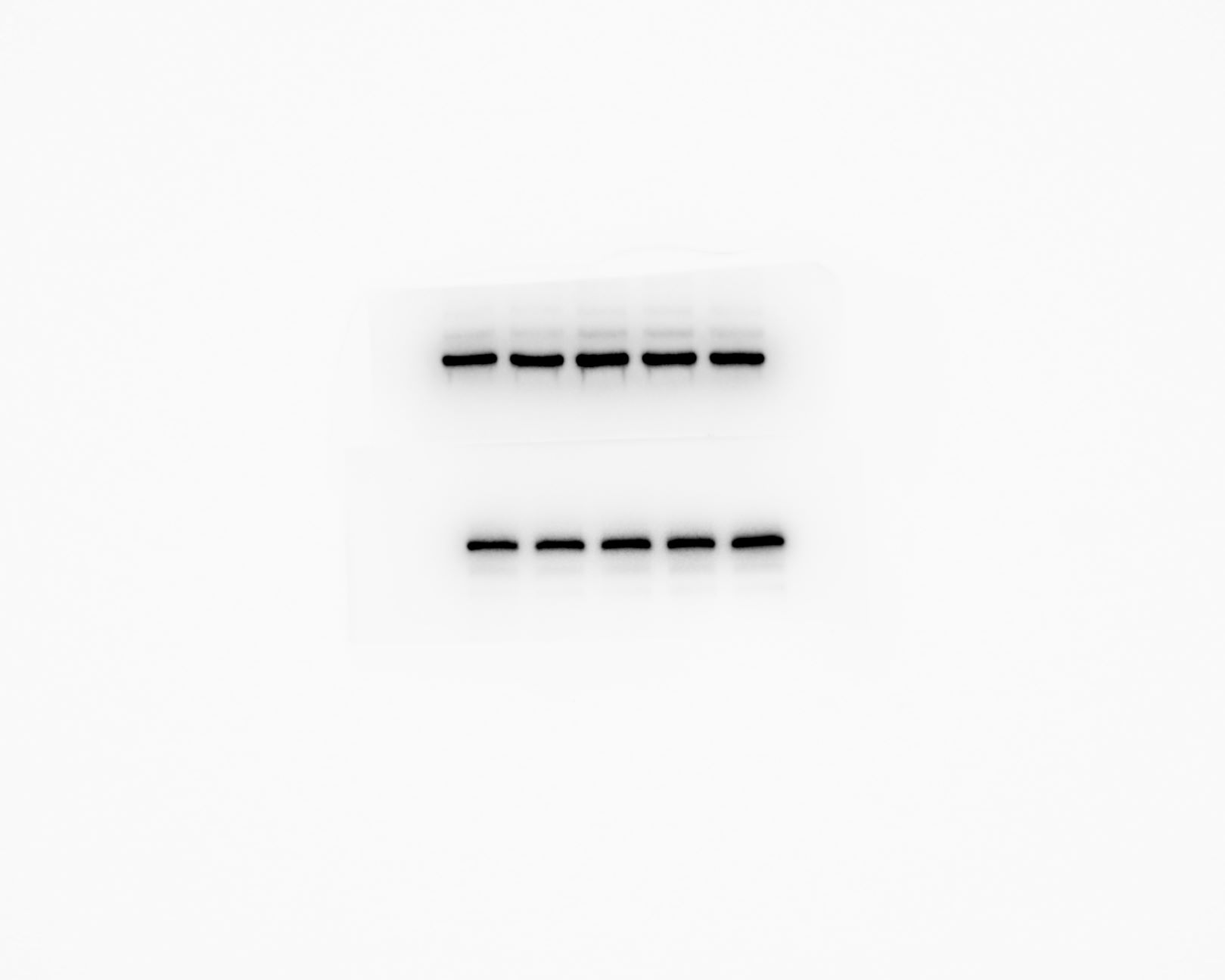

Supplement: Supplementary file 7 — Source Data for Figure 2 [file EMMM-13-e14887-s001.zip › EMM-2021-14887_SDataFig2/Fig. 2C_D WB and Intensity/293T_Vinculin.jpg]

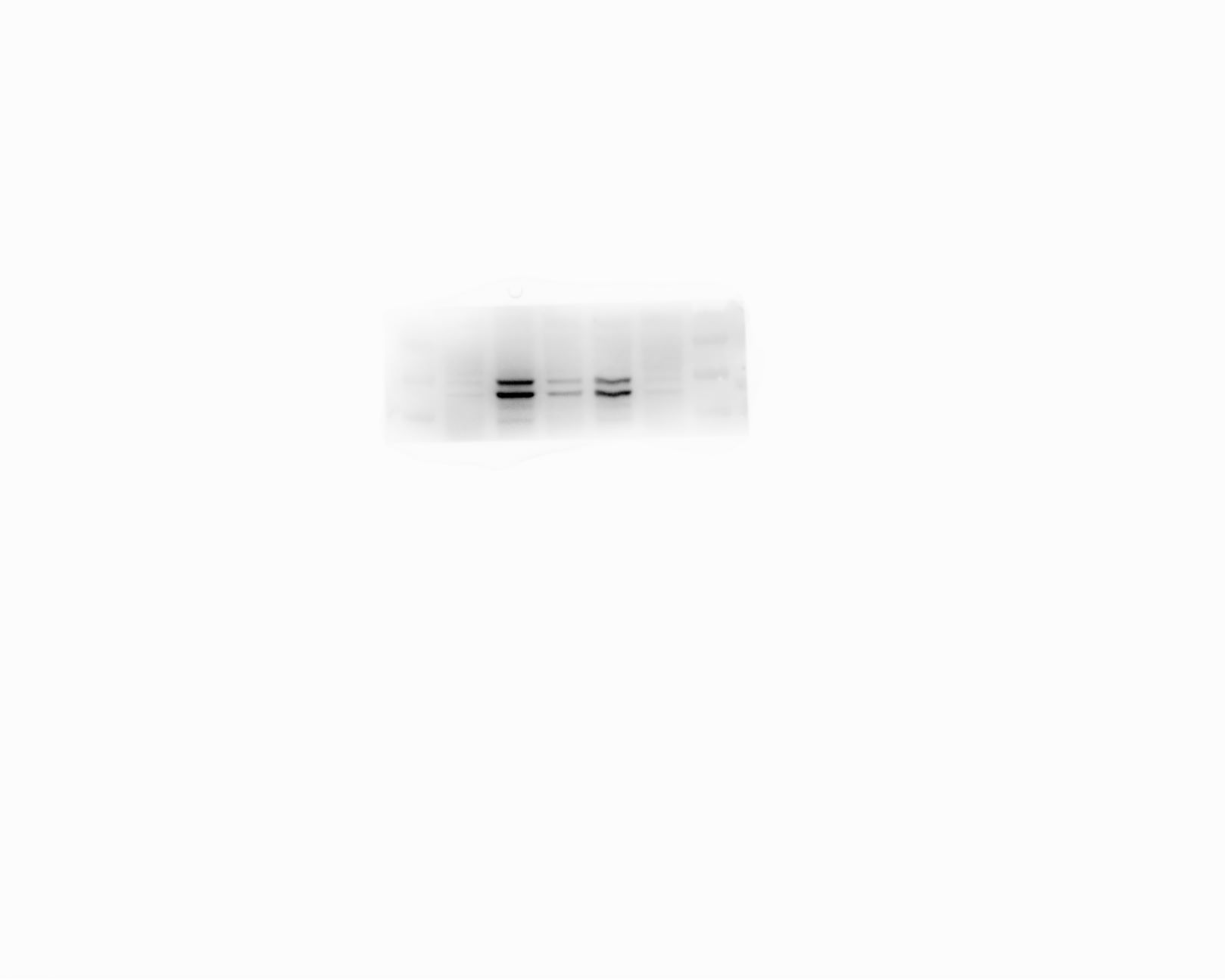

Supplement: Supplementary file 7 — Source Data for Figure 2 [file EMMM-13-e14887-s001.zip › EMM-2021-14887_SDataFig2/Fig. 2C_D WB and Intensity/293T_pERK.jpg]

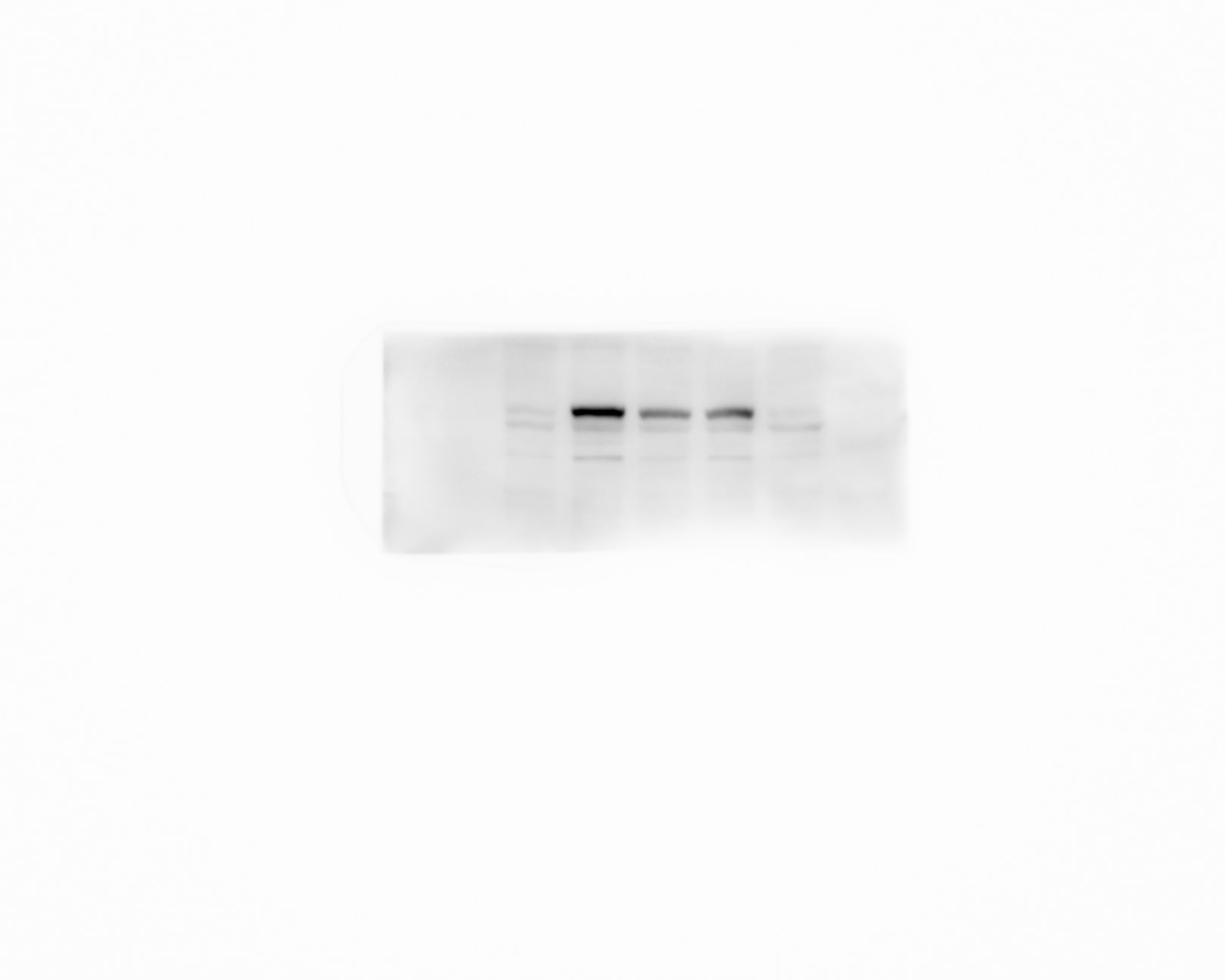

Supplement: Supplementary file 7 — Source Data for Figure 2 [file EMMM-13-e14887-s001.zip › EMM-2021-14887_SDataFig2/Fig. 2C_D WB and Intensity/293T_pMEK.jpg]

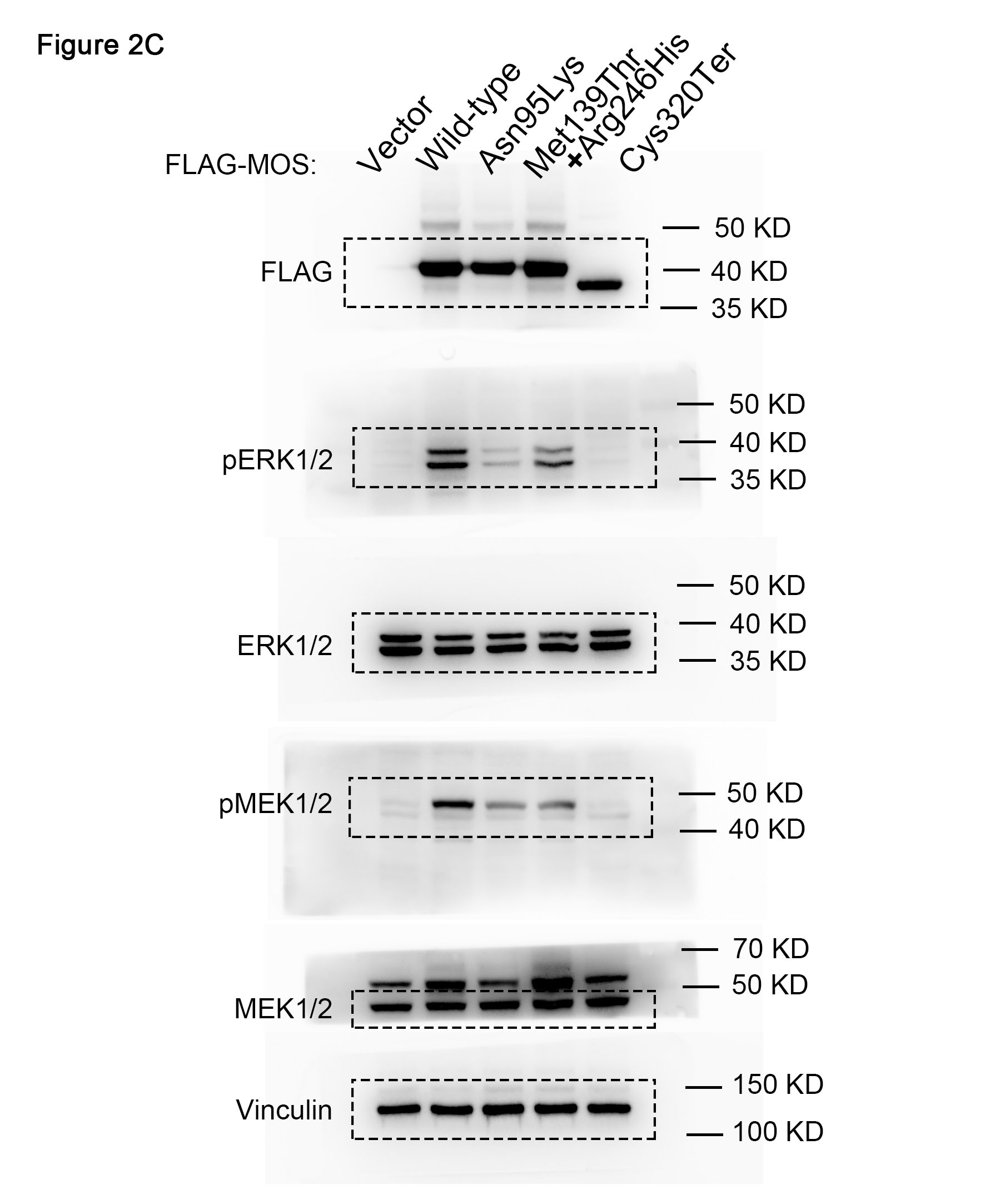

Supplement: Supplementary file 7 — Source Data for Figure 2 [file EMMM-13-e14887-s001.zip › EMM-2021-14887_SDataFig2/Fig. 2C_D WB and Intensity/FIgure 2C Source Data_Labeled.jpg]

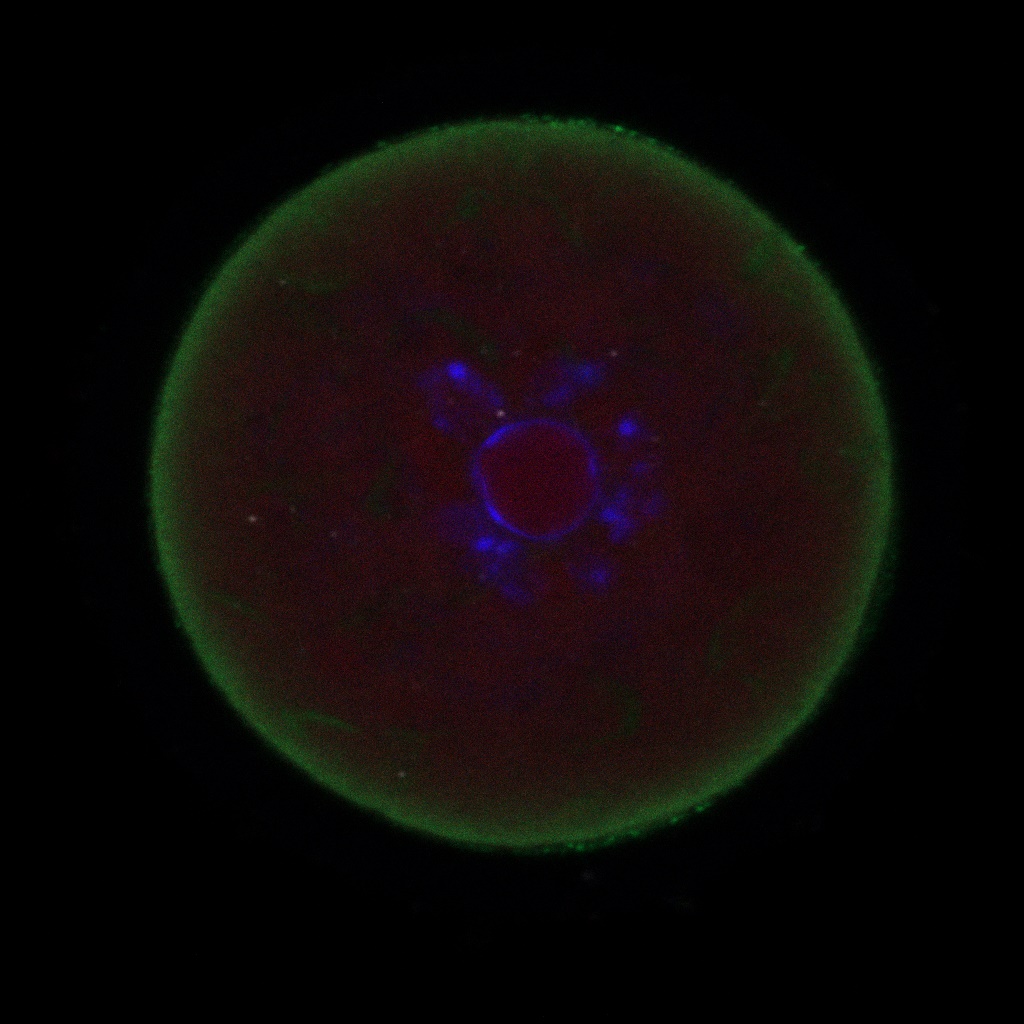

Supplement: Supplementary file 7 — Source Data for Figure 2 [file EMMM-13-e14887-s001.zip › EMM-2021-14887_SDataFig2/Fig. 2F Images/C320ter-flag-mcherry-pERK(647)-40x-3-Image Export-03_c1-4.jpg]

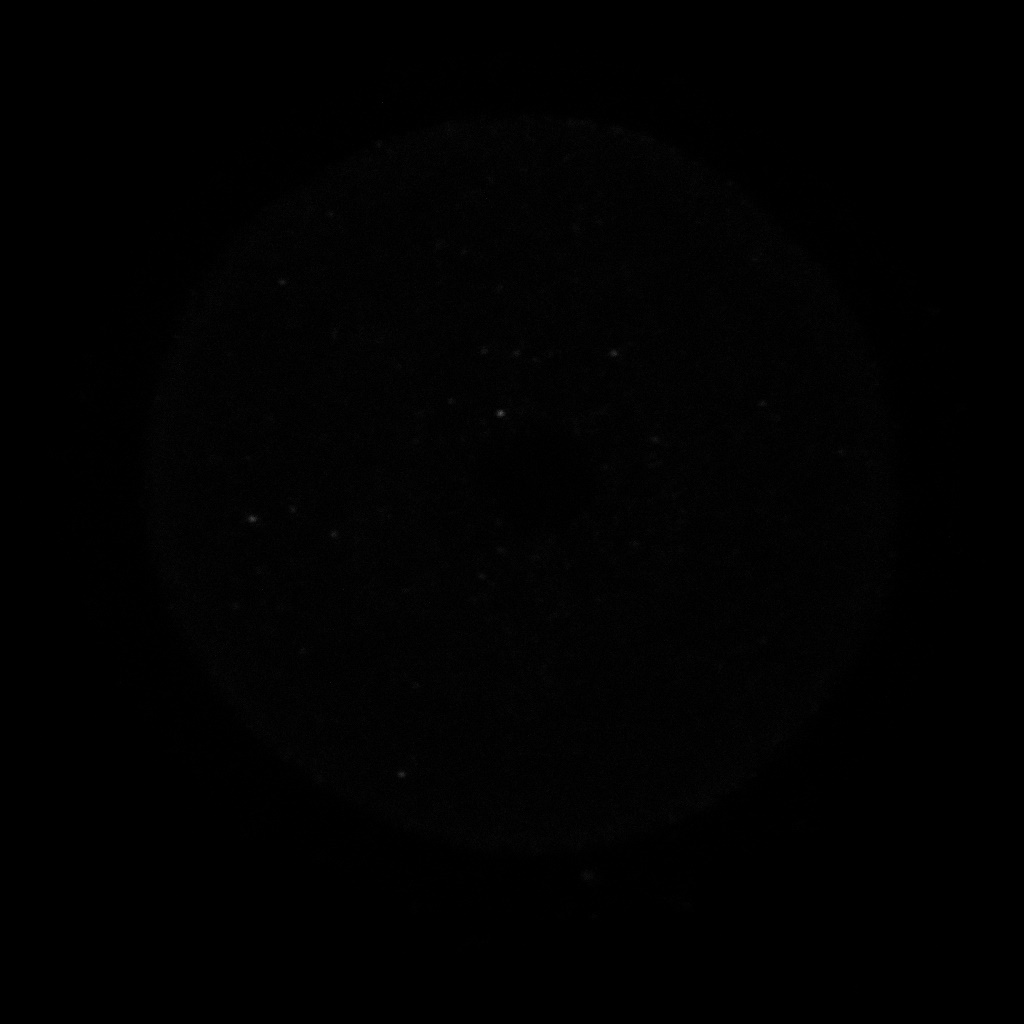

Supplement: Supplementary file 7 — Source Data for Figure 2 [file EMMM-13-e14887-s001.zip › EMM-2021-14887_SDataFig2/Fig. 2F Images/C320ter-flag-mcherry-pERK(647)-40x-3-Image Export-03_c1.jpg]

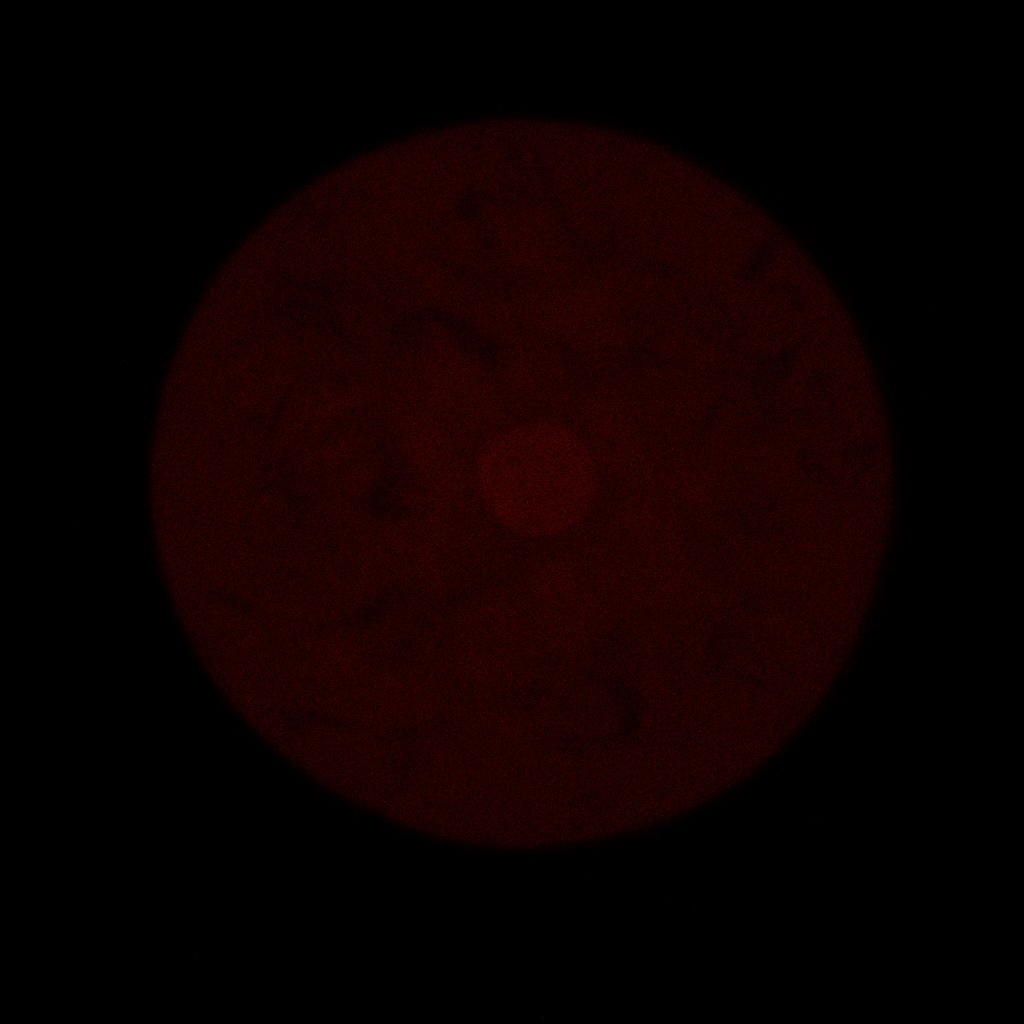

Supplement: Supplementary file 7 — Source Data for Figure 2 [file EMMM-13-e14887-s001.zip › EMM-2021-14887_SDataFig2/Fig. 2F Images/C320ter-flag-mcherry-pERK(647)-40x-3-Image Export-03_c2.jpg]

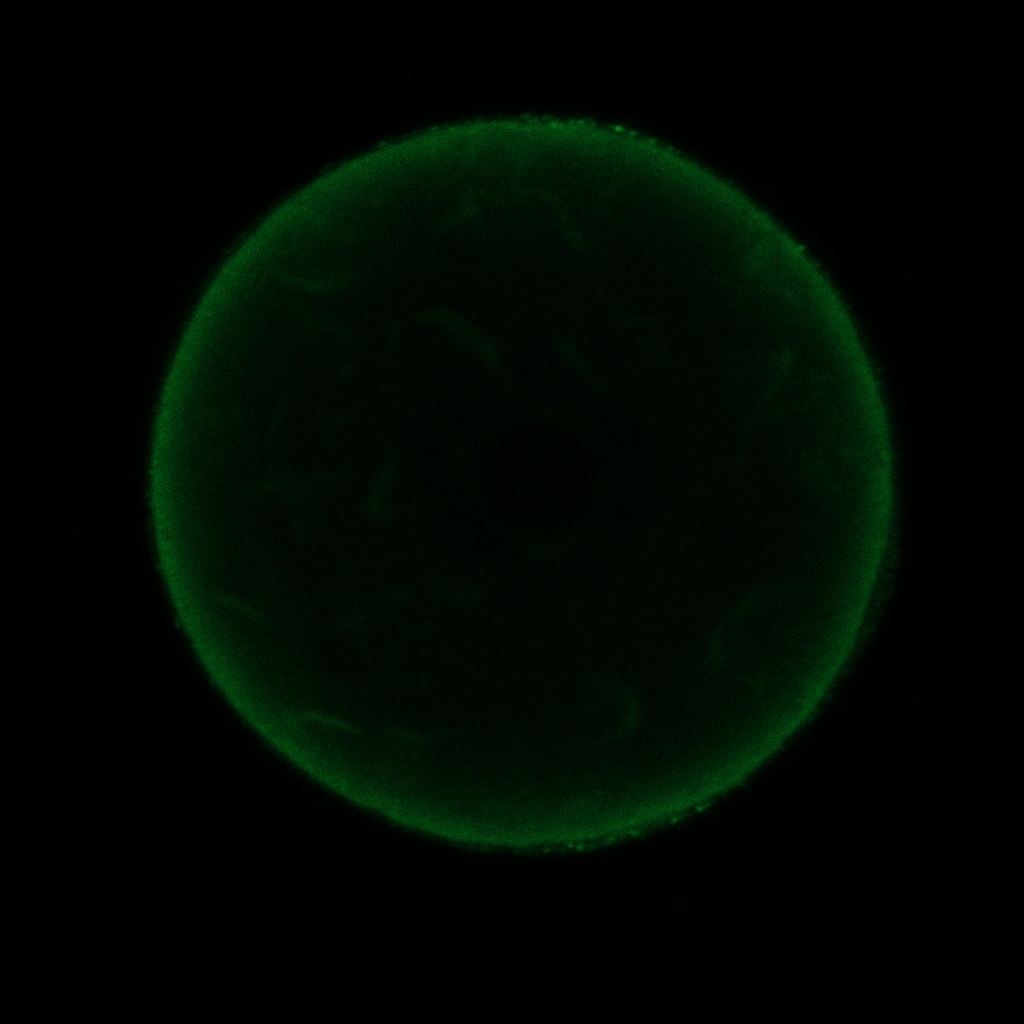

Supplement: Supplementary file 7 — Source Data for Figure 2 [file EMMM-13-e14887-s001.zip › EMM-2021-14887_SDataFig2/Fig. 2F Images/C320ter-flag-mcherry-pERK(647)-40x-3-Image Export-03_c3.jpg]

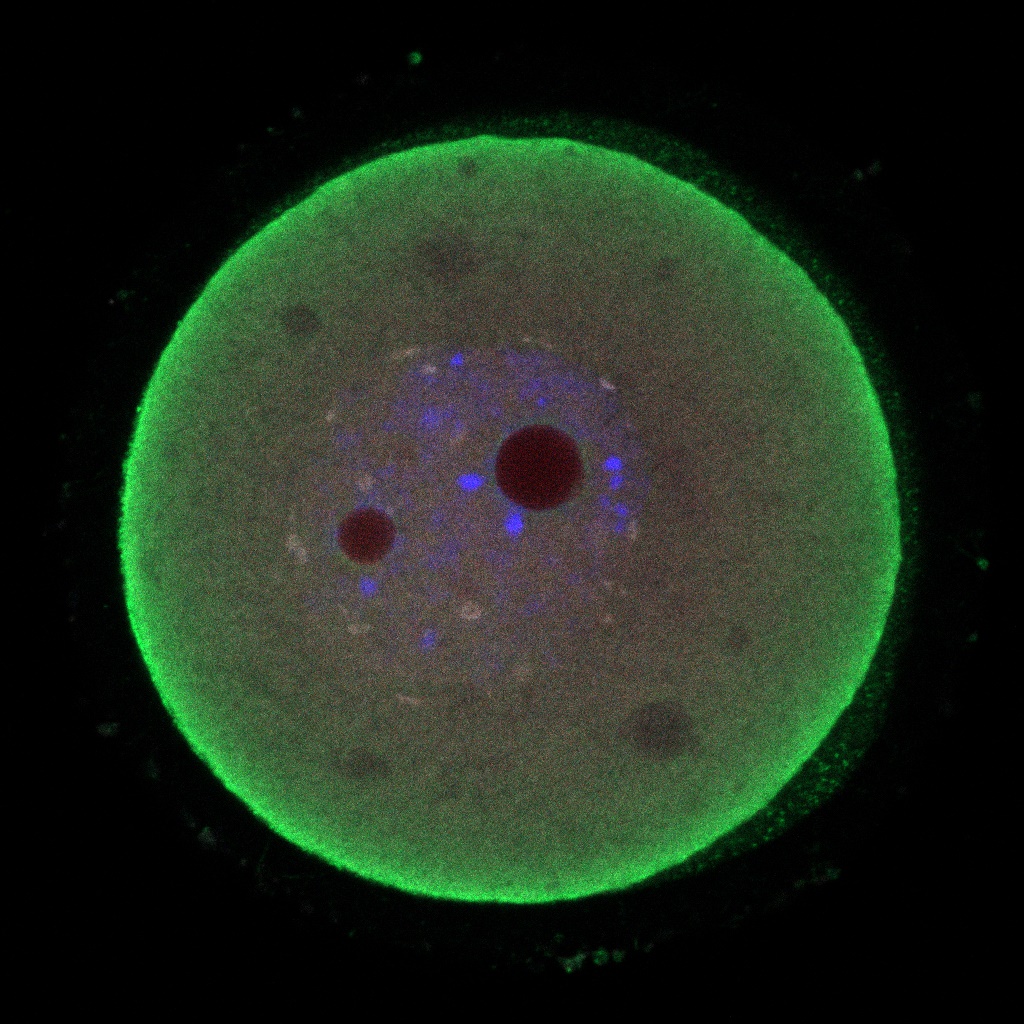

Supplement: Supplementary file 7 — Source Data for Figure 2 [file EMMM-13-e14887-s001.zip › EMM-2021-14887_SDataFig2/Fig. 2F Images/FLGA-MOS-pERK(647)-40x-1-Image Export-18_c1-4.jpg]

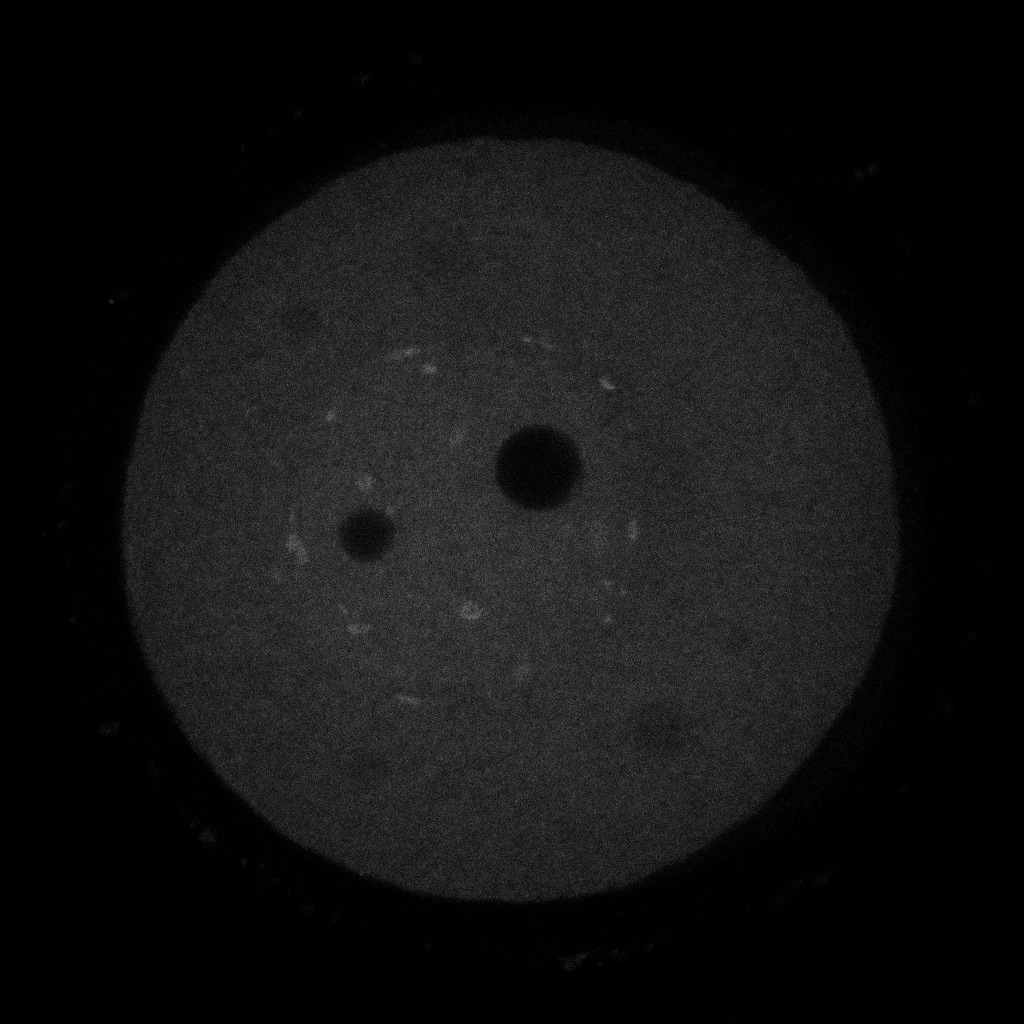

Supplement: Supplementary file 7 — Source Data for Figure 2 [file EMMM-13-e14887-s001.zip › EMM-2021-14887_SDataFig2/Fig. 2F Images/FLGA-MOS-pERK(647)-40x-1-Image Export-18_c1.jpg]

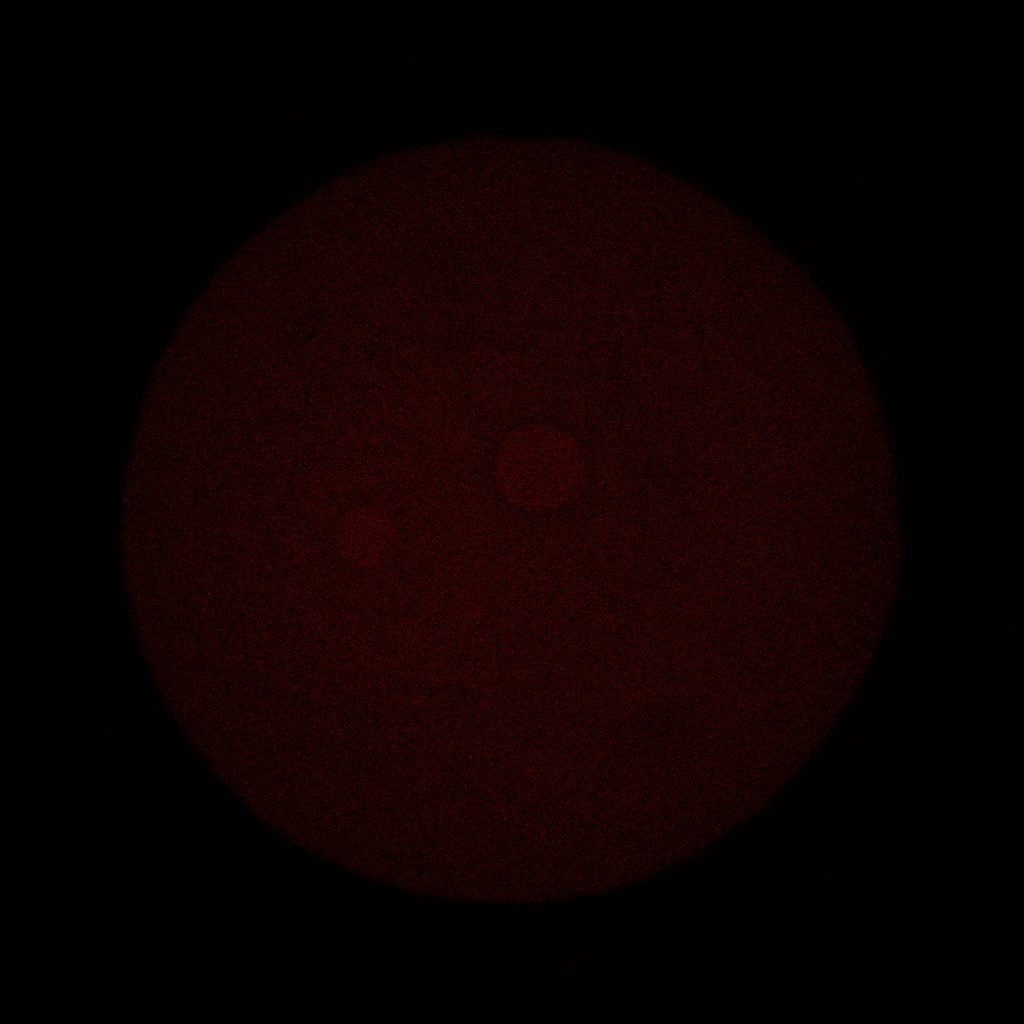

Supplement: Supplementary file 7 — Source Data for Figure 2 [file EMMM-13-e14887-s001.zip › EMM-2021-14887_SDataFig2/Fig. 2F Images/FLGA-MOS-pERK(647)-40x-1-Image Export-18_c2.jpg]

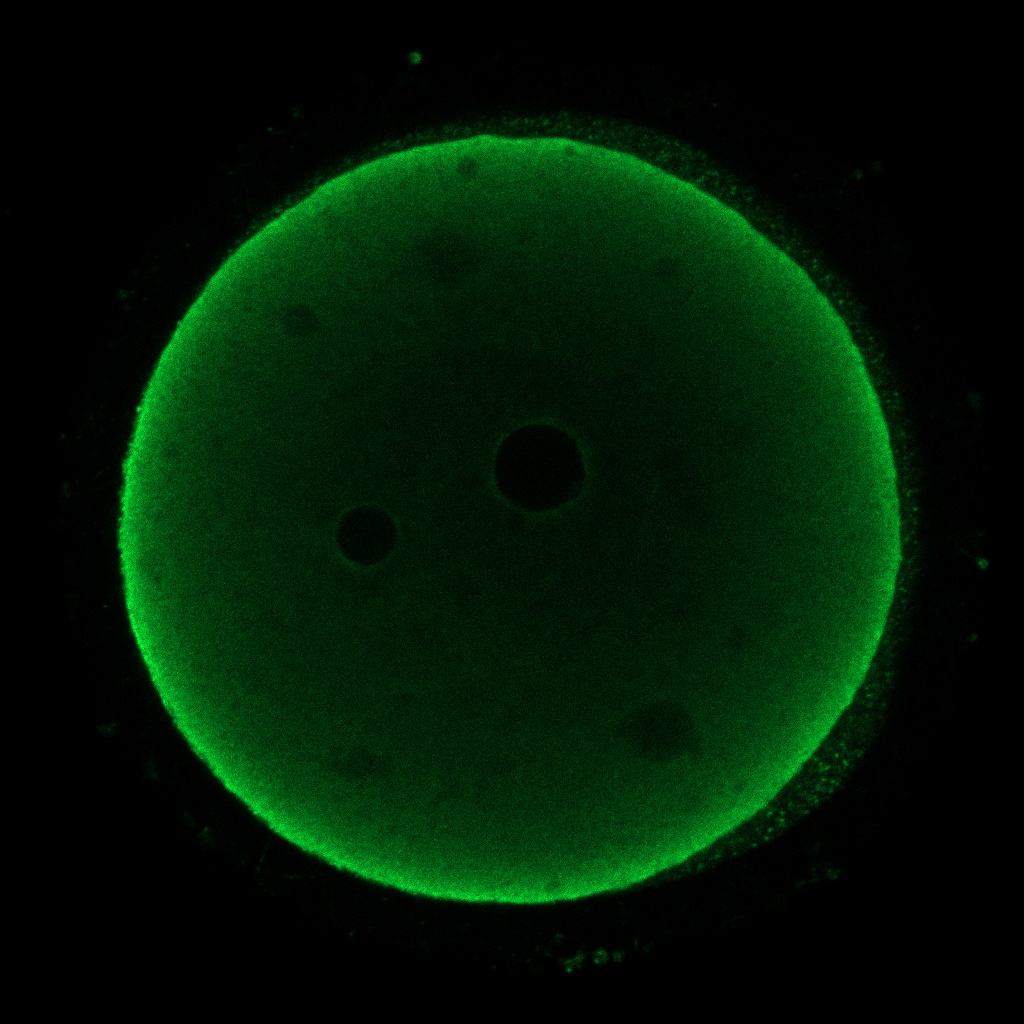

Supplement: Supplementary file 7 — Source Data for Figure 2 [file EMMM-13-e14887-s001.zip › EMM-2021-14887_SDataFig2/Fig. 2F Images/FLGA-MOS-pERK(647)-40x-1-Image Export-18_c3.jpg]

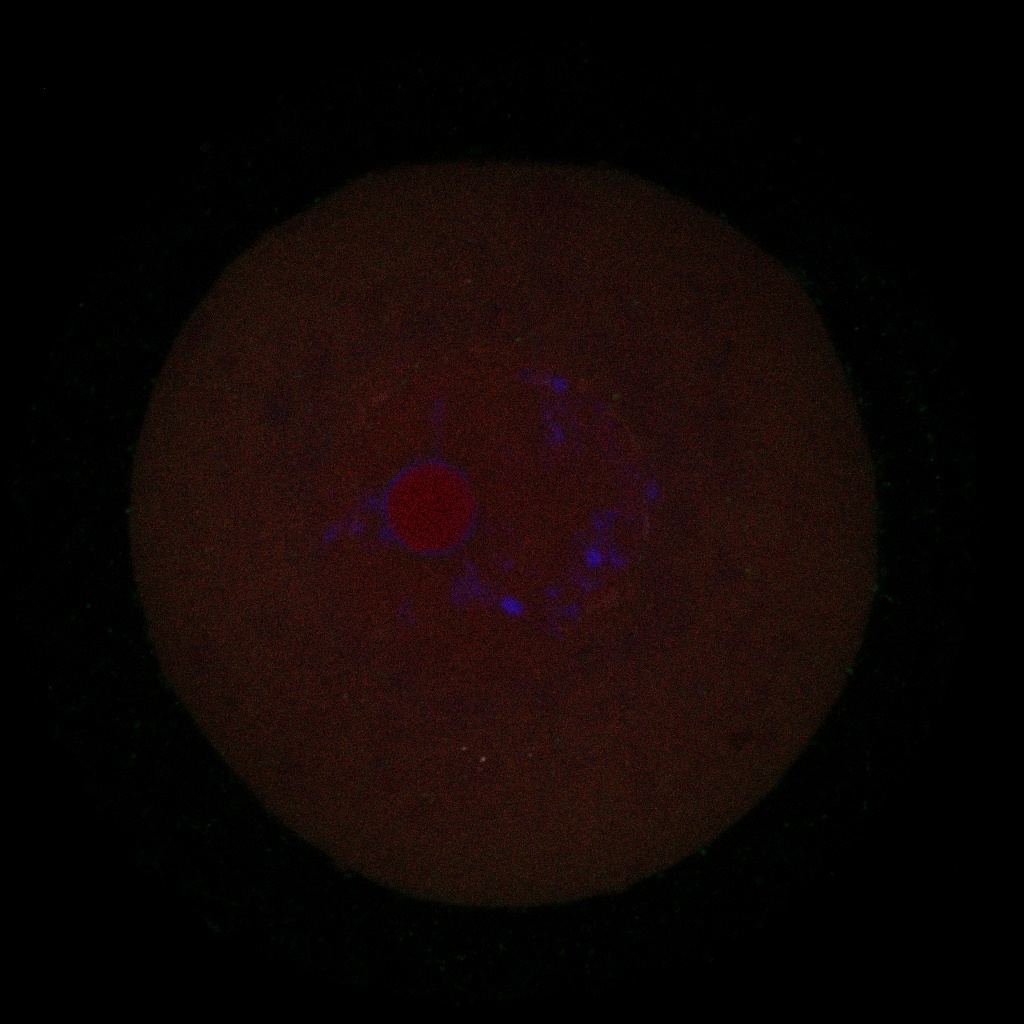

Supplement: Supplementary file 7 — Source Data for Figure 2 [file EMMM-13-e14887-s001.zip › EMM-2021-14887_SDataFig2/Fig. 2F Images/FLGA-mcherry-pERK(647)-40x-1-Image Export-01_c1-4.jpg]

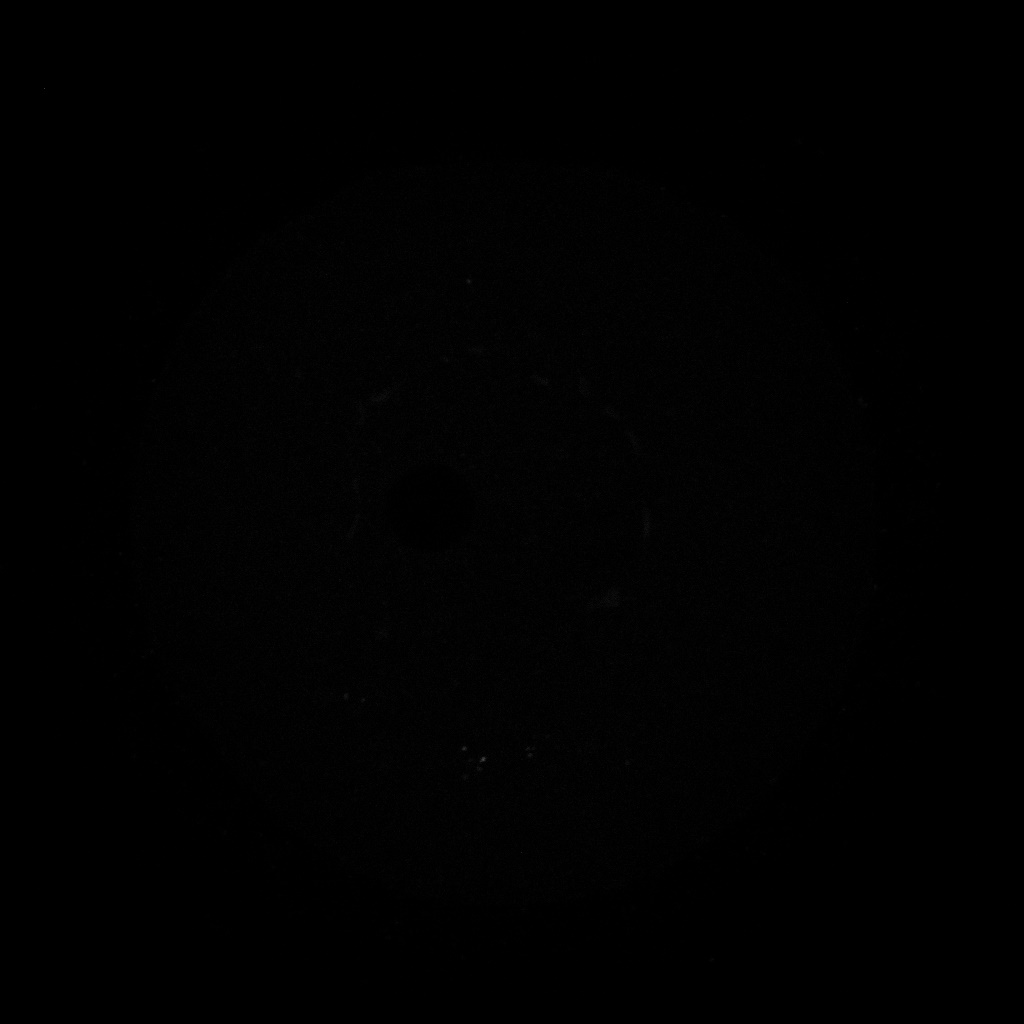

Supplement: Supplementary file 7 — Source Data for Figure 2 [file EMMM-13-e14887-s001.zip › EMM-2021-14887_SDataFig2/Fig. 2F Images/FLGA-mcherry-pERK(647)-40x-1-Image Export-01_c1.jpg]

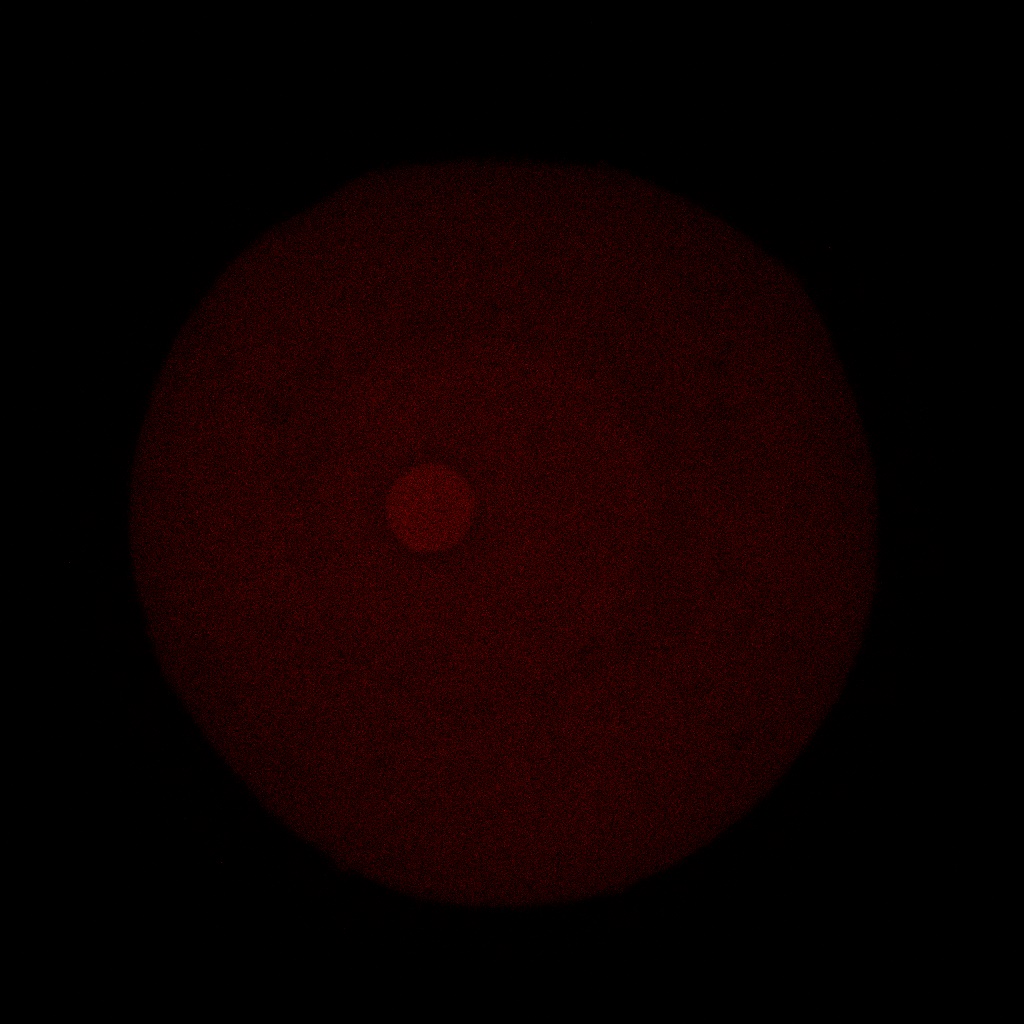

Supplement: Supplementary file 7 — Source Data for Figure 2 [file EMMM-13-e14887-s001.zip › EMM-2021-14887_SDataFig2/Fig. 2F Images/FLGA-mcherry-pERK(647)-40x-1-Image Export-01_c2.jpg]

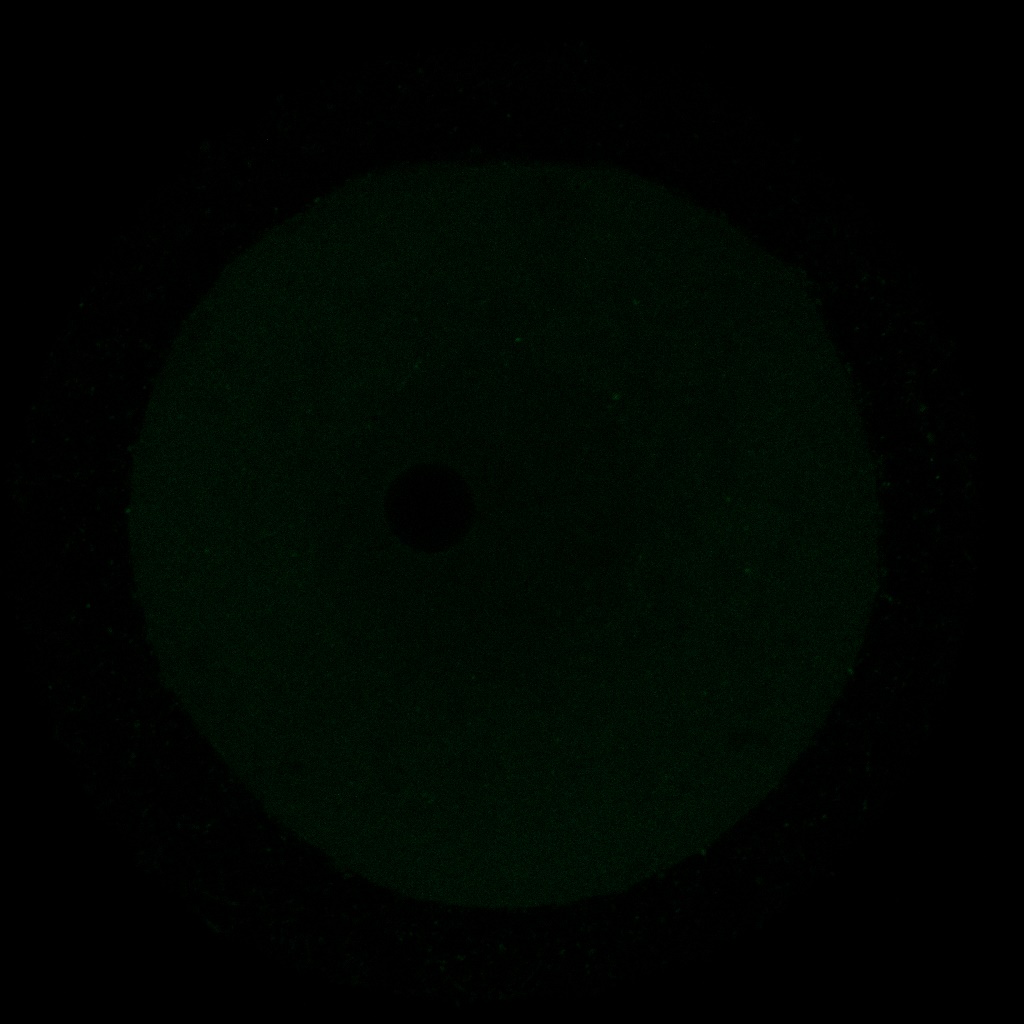

Supplement: Supplementary file 7 — Source Data for Figure 2 [file EMMM-13-e14887-s001.zip › EMM-2021-14887_SDataFig2/Fig. 2F Images/FLGA-mcherry-pERK(647)-40x-1-Image Export-01_c3.jpg]

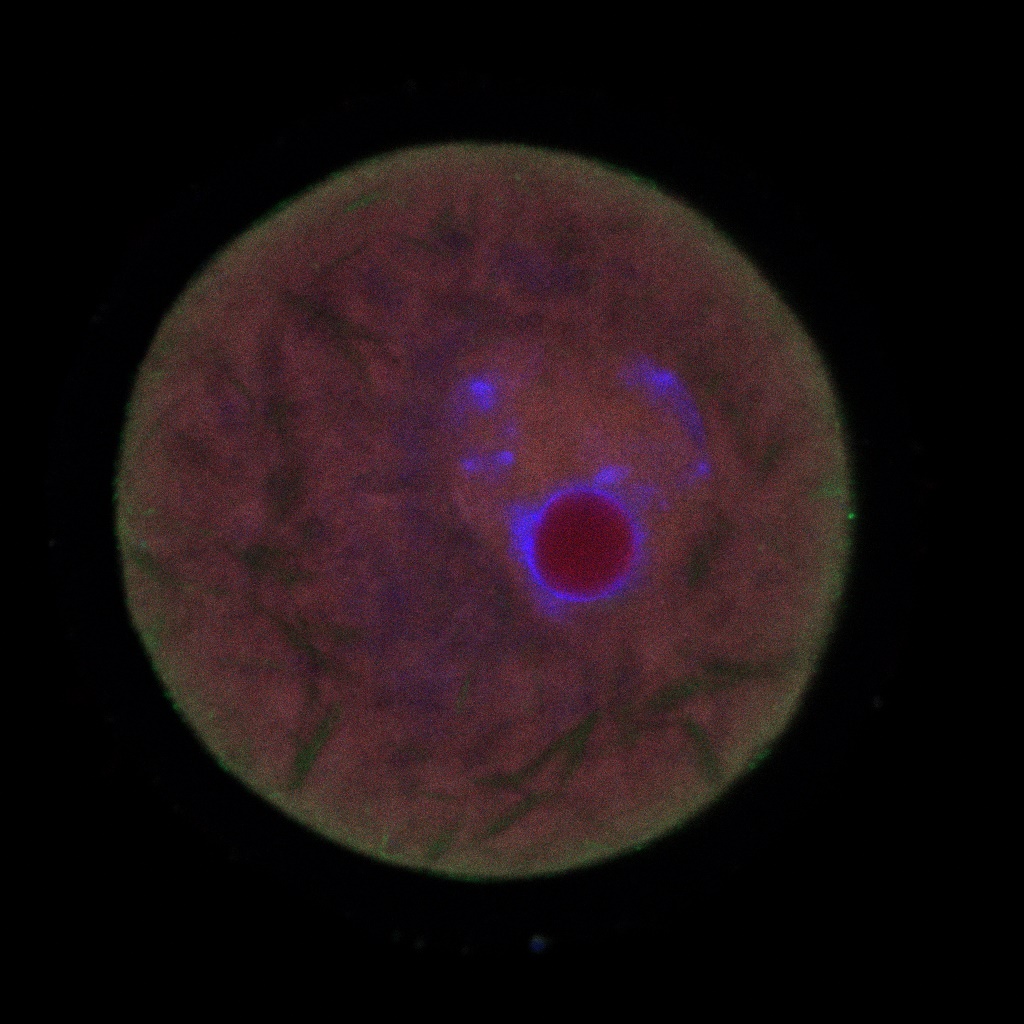

Supplement: Supplementary file 7 — Source Data for Figure 2 [file EMMM-13-e14887-s001.zip › EMM-2021-14887_SDataFig2/Fig. 2F Images/M139T-R246H_flag-mcherry-pERK(647)-40x-7-Image Export-24_c1-4.jpg]

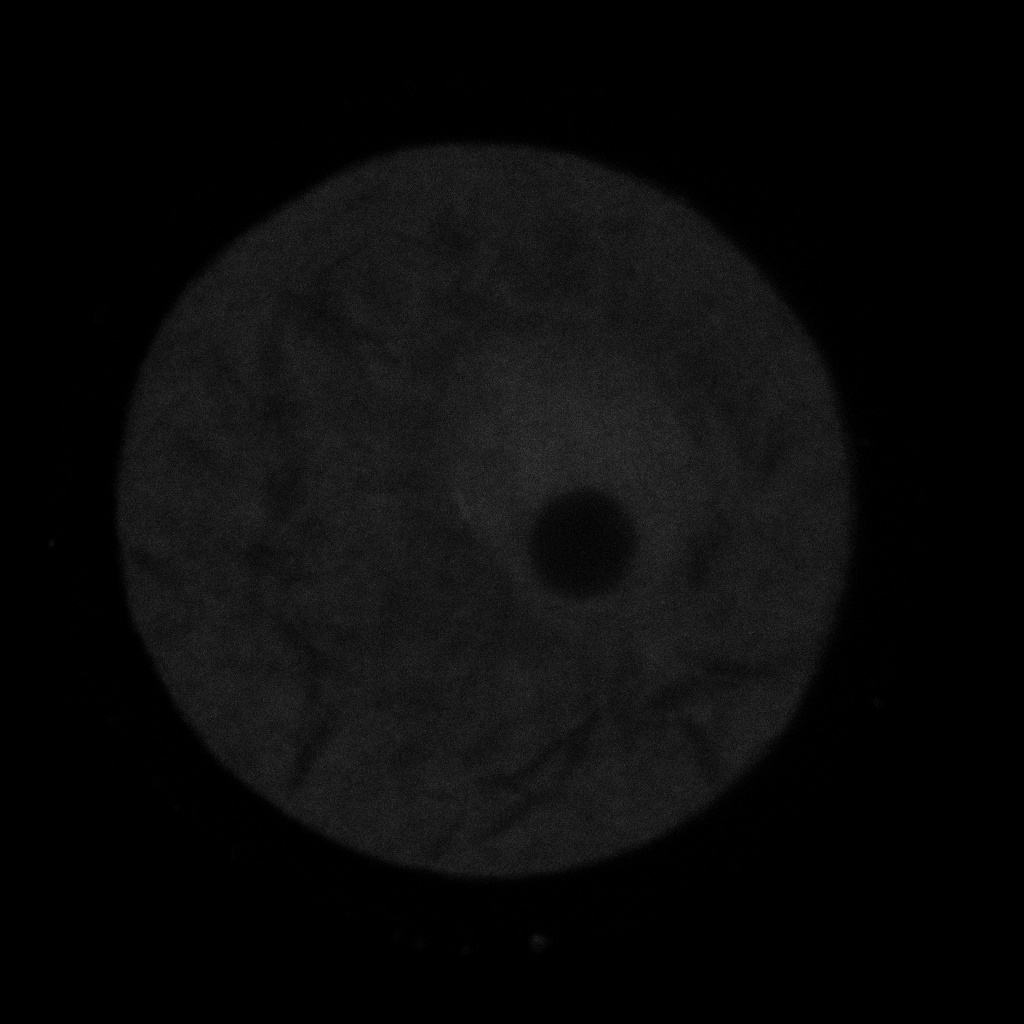

Supplement: Supplementary file 7 — Source Data for Figure 2 [file EMMM-13-e14887-s001.zip › EMM-2021-14887_SDataFig2/Fig. 2F Images/M139T-R246H_flag-mcherry-pERK(647)-40x-7-Image Export-24_c1.jpg]

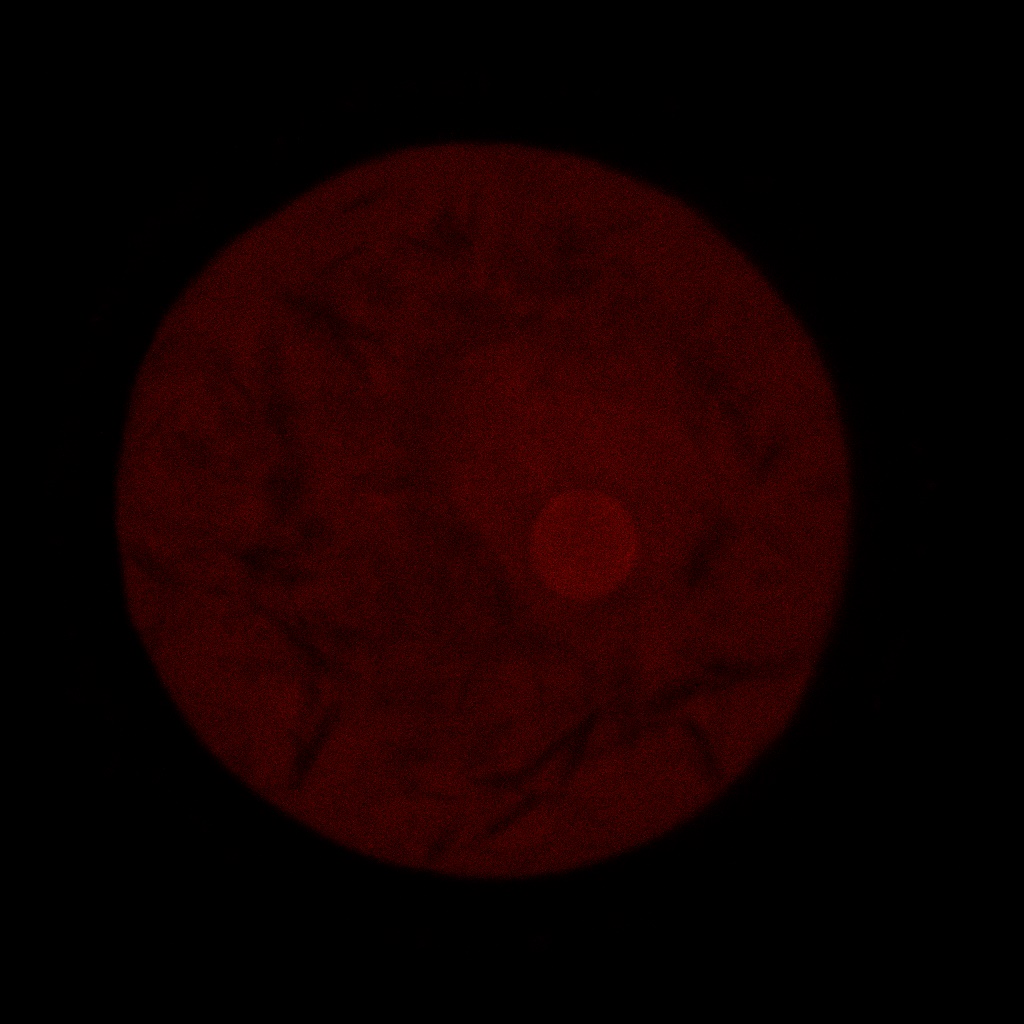

Supplement: Supplementary file 7 — Source Data for Figure 2 [file EMMM-13-e14887-s001.zip › EMM-2021-14887_SDataFig2/Fig. 2F Images/M139T-R246H_flag-mcherry-pERK(647)-40x-7-Image Export-24_c2.jpg]

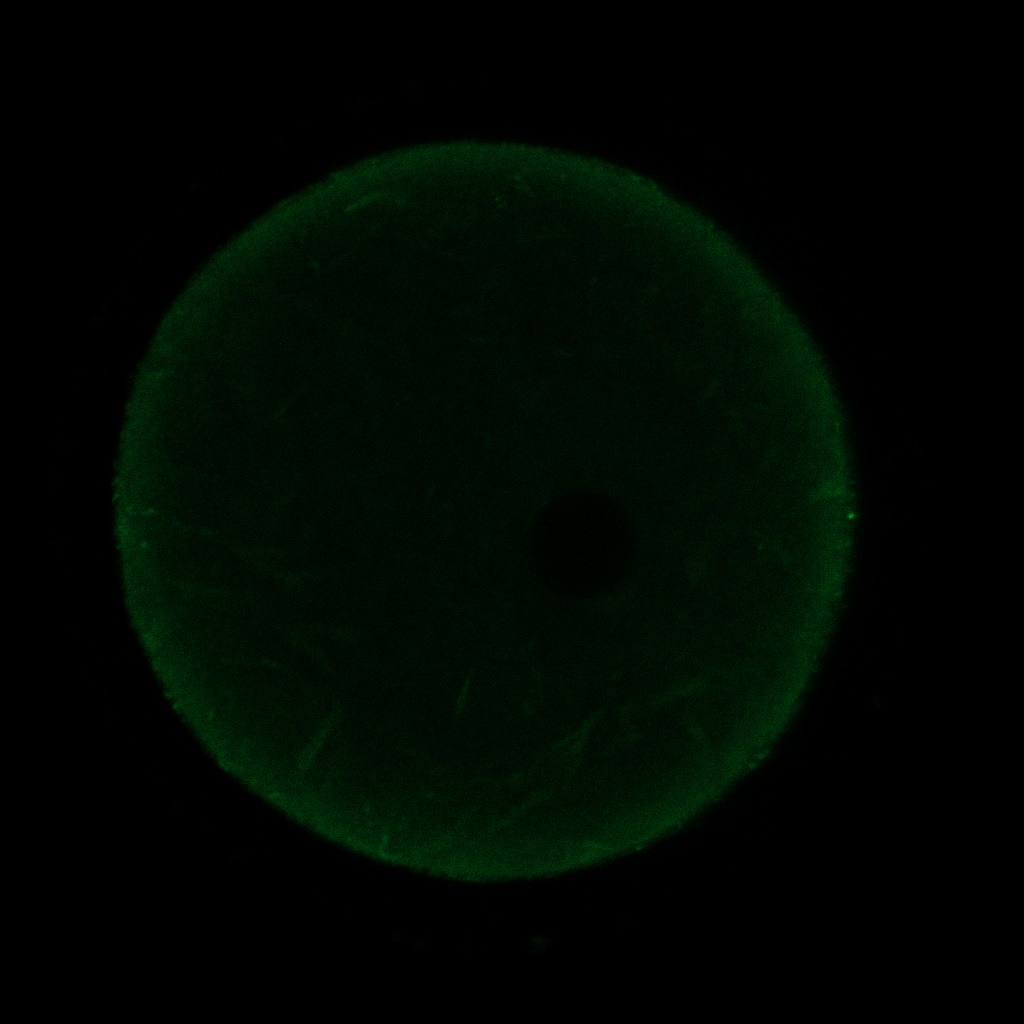

Supplement: Supplementary file 7 — Source Data for Figure 2 [file EMMM-13-e14887-s001.zip › EMM-2021-14887_SDataFig2/Fig. 2F Images/M139T-R246H_flag-mcherry-pERK(647)-40x-7-Image Export-24_c3.jpg]

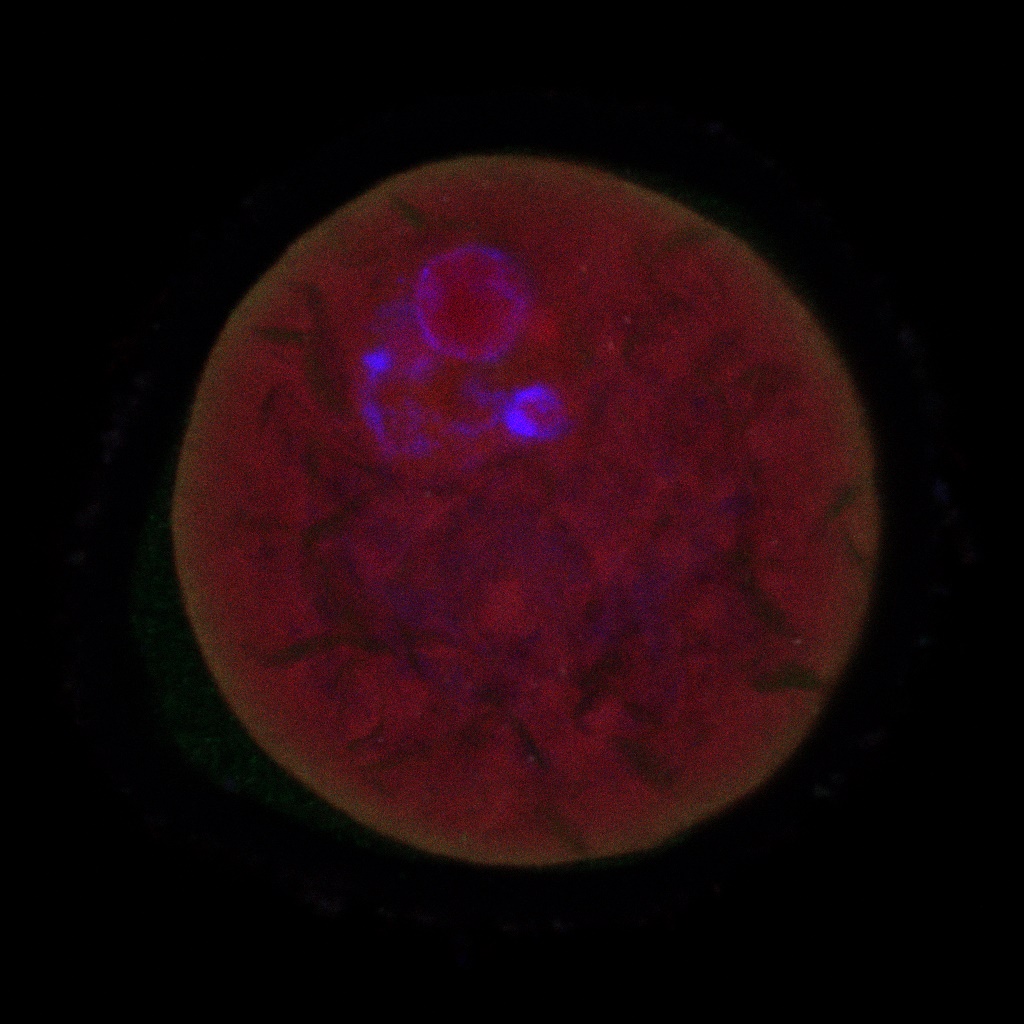

Supplement: Supplementary file 7 — Source Data for Figure 2 [file EMMM-13-e14887-s001.zip › EMM-2021-14887_SDataFig2/Fig. 2F Images/n95k-flag-mcherry-pERK(647)-40x-5-Image Export-22_c1-4.jpg]

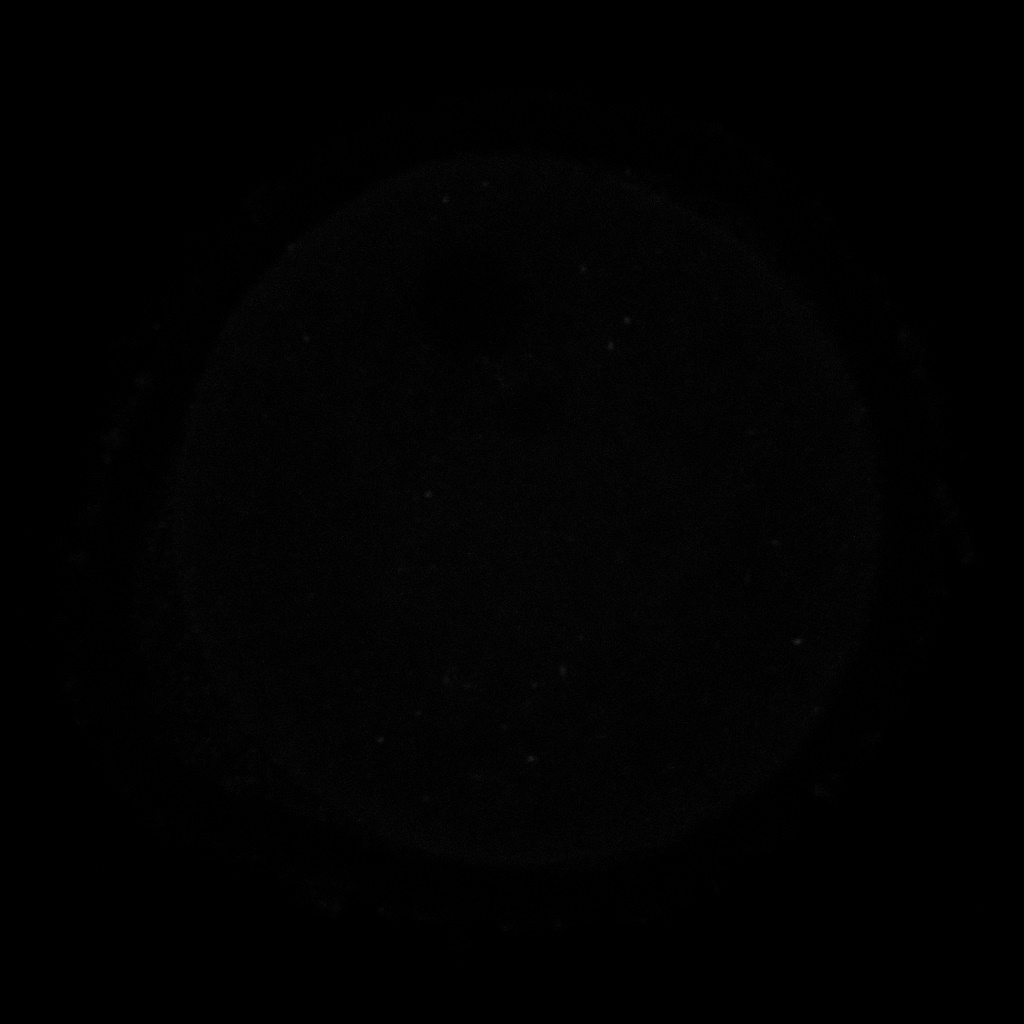

Supplement: Supplementary file 7 — Source Data for Figure 2 [file EMMM-13-e14887-s001.zip › EMM-2021-14887_SDataFig2/Fig. 2F Images/n95k-flag-mcherry-pERK(647)-40x-5-Image Export-22_c1.jpg]

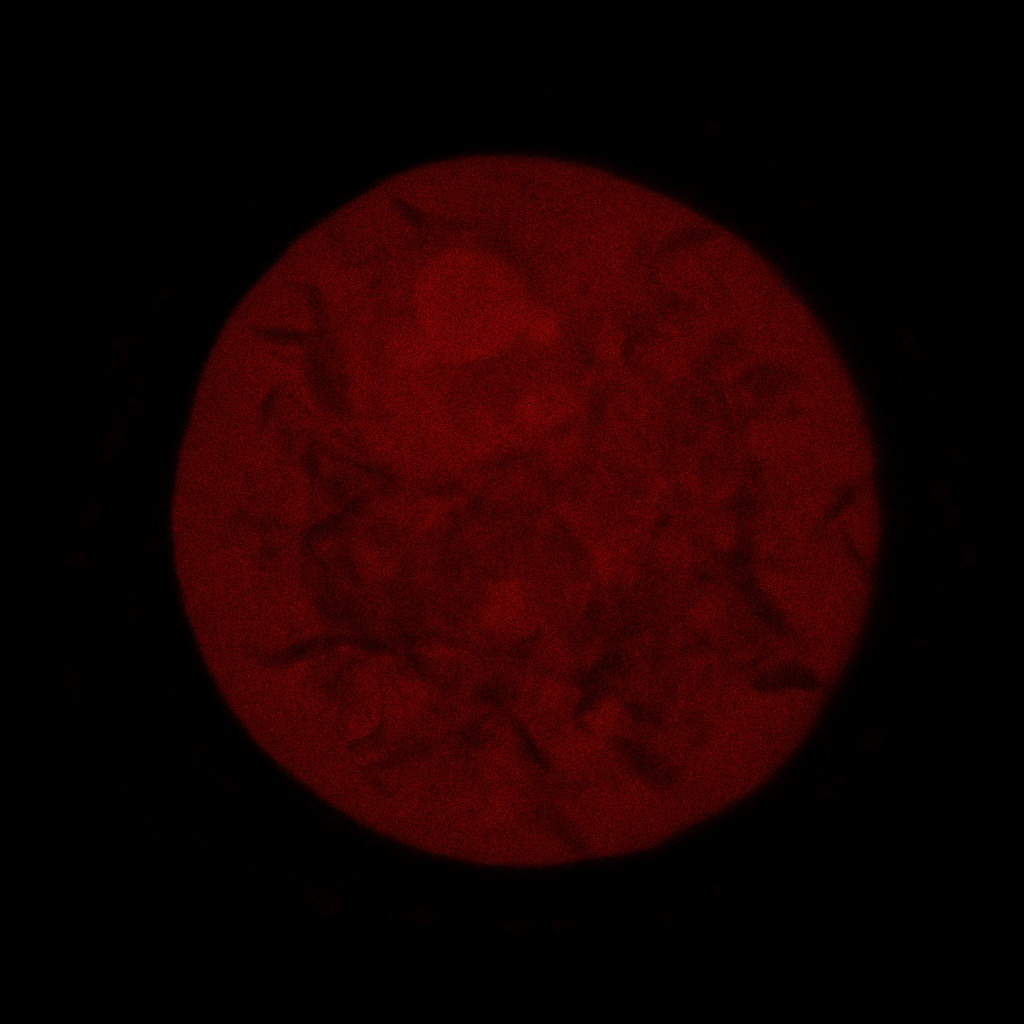

Supplement: Supplementary file 7 — Source Data for Figure 2 [file EMMM-13-e14887-s001.zip › EMM-2021-14887_SDataFig2/Fig. 2F Images/n95k-flag-mcherry-pERK(647)-40x-5-Image Export-22_c2.jpg]

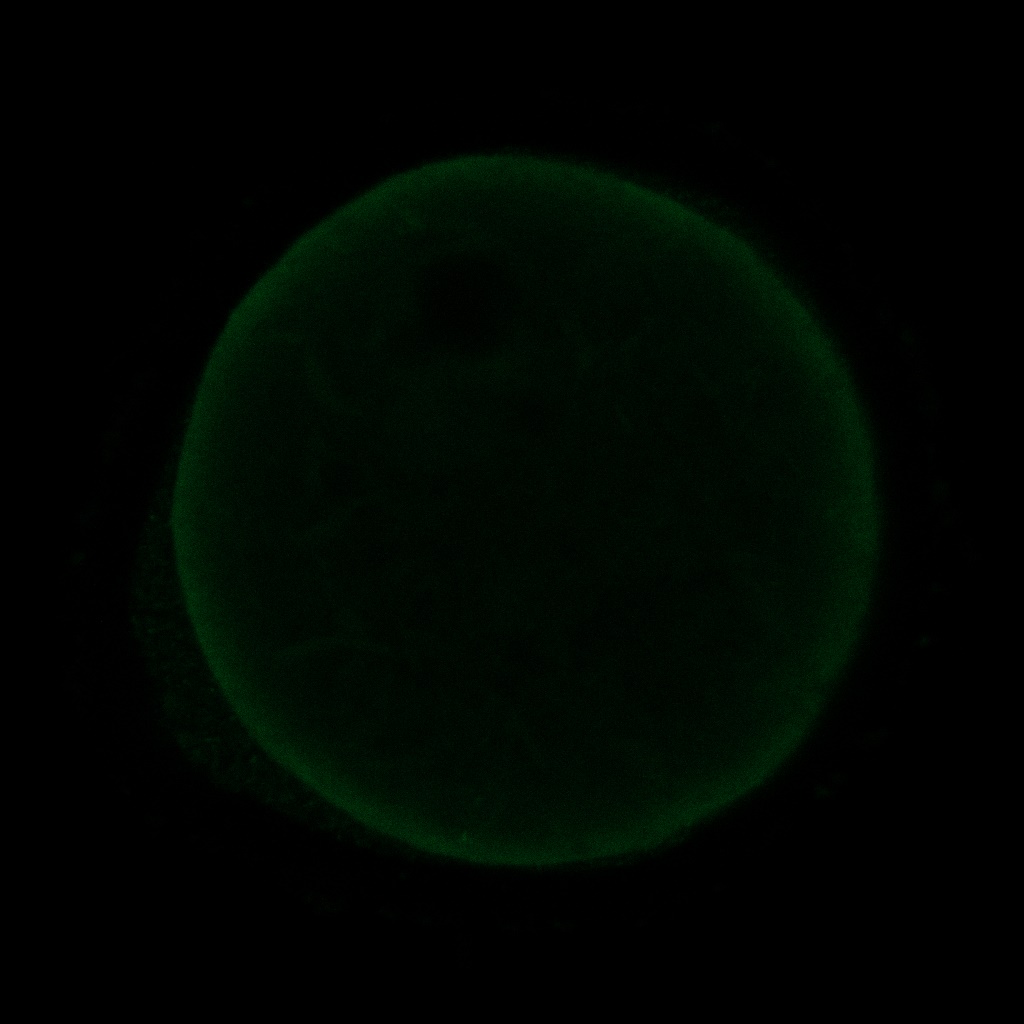

Supplement: Supplementary file 7 — Source Data for Figure 2 [file EMMM-13-e14887-s001.zip › EMM-2021-14887_SDataFig2/Fig. 2F Images/n95k-flag-mcherry-pERK(647)-40x-5-Image Export-22_c3.jpg]

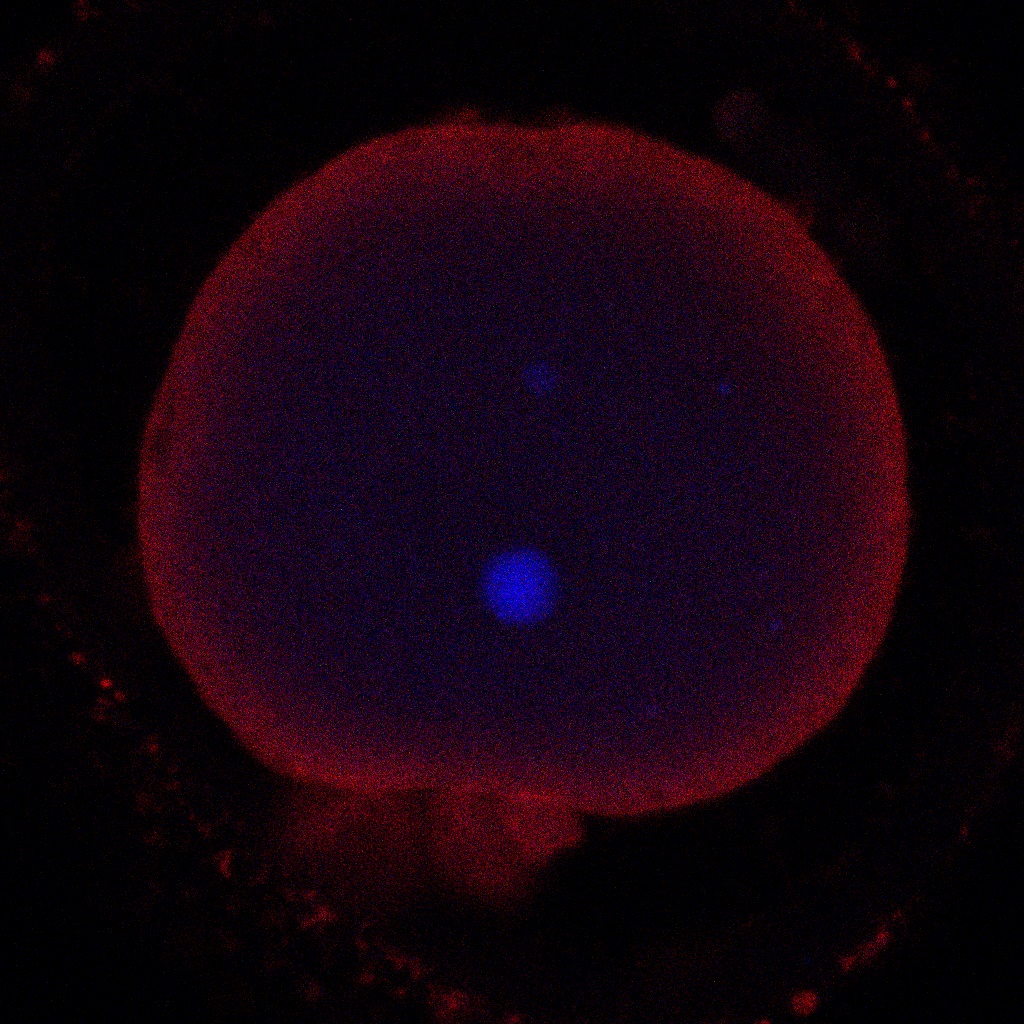

Supplement: Supplementary file 8 — Source Data for Figure 3 [file EMMM-13-e14887-s005.zip › EMM-2021-14887_SDataFig3/Fig. 3A/NC pERK-FITC-1/NC pERK-FITC-1-图像导出-01_h0t0z0c0-3x0-1024y0-1024.jpg]

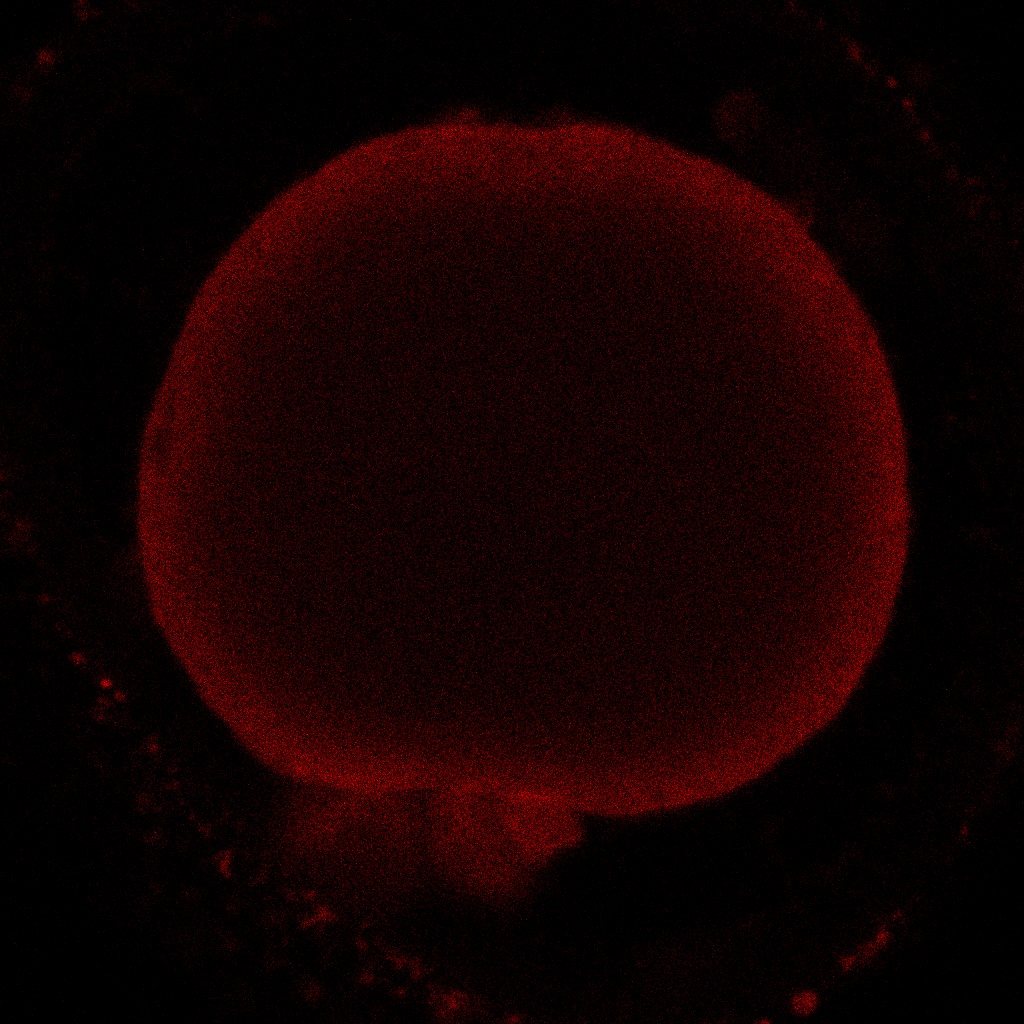

Supplement: Supplementary file 8 — Source Data for Figure 3 [file EMMM-13-e14887-s005.zip › EMM-2021-14887_SDataFig3/Fig. 3A/NC pERK-FITC-1/NC pERK-FITC-1-图像导出-01_h0t0z0c0x0-1024y0-1024.jpg]

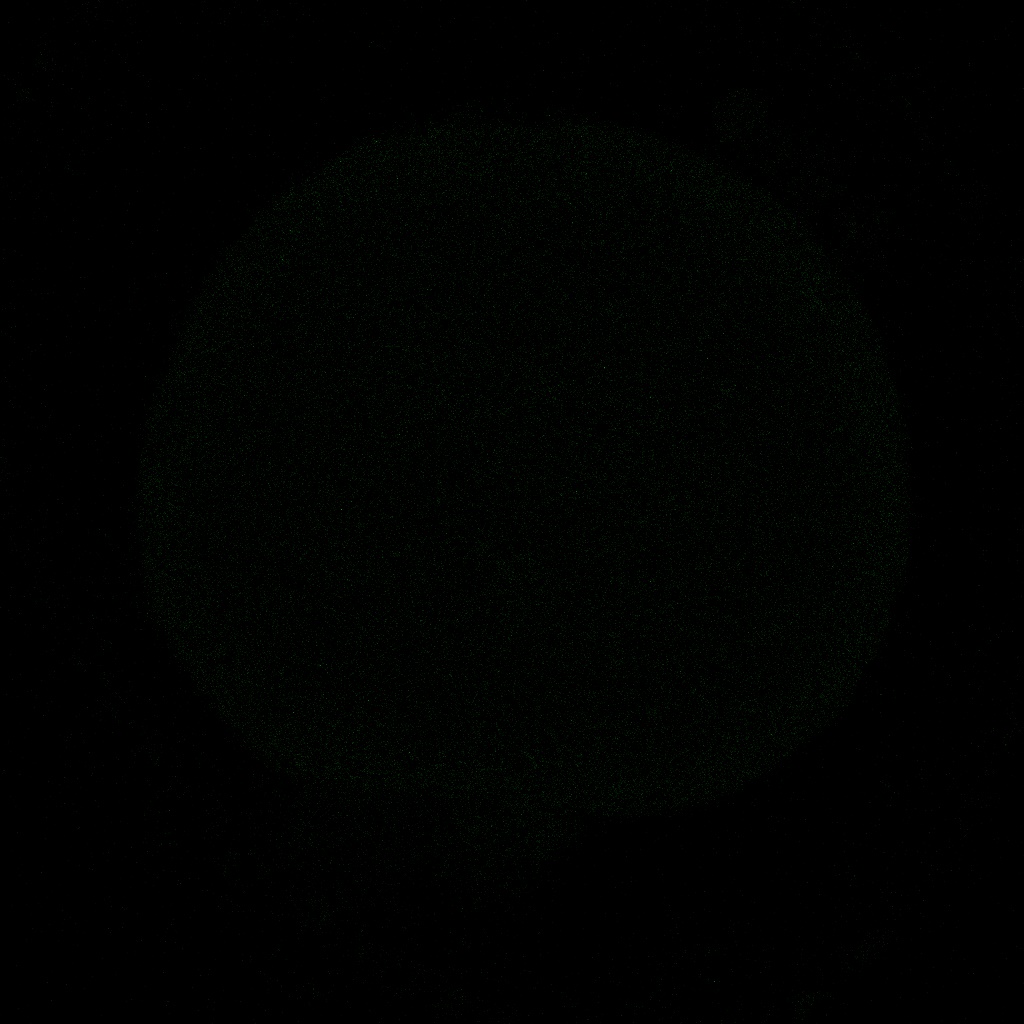

Supplement: Supplementary file 8 — Source Data for Figure 3 [file EMMM-13-e14887-s005.zip › EMM-2021-14887_SDataFig3/Fig. 3A/NC pERK-FITC-1/NC pERK-FITC-1-图像导出-01_h0t0z0c1x0-1024y0-1024.jpg]

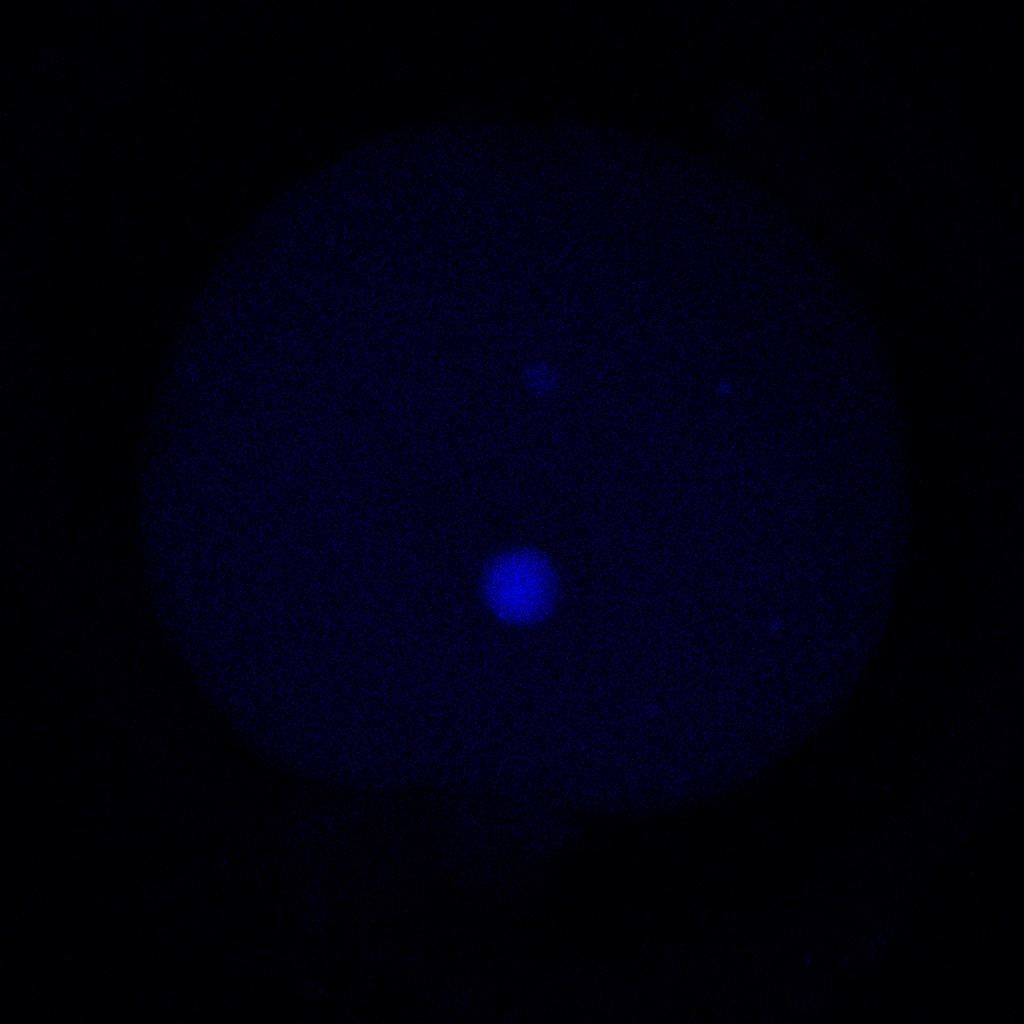

Supplement: Supplementary file 8 — Source Data for Figure 3 [file EMMM-13-e14887-s005.zip › EMM-2021-14887_SDataFig3/Fig. 3A/NC pERK-FITC-1/NC pERK-FITC-1-图像导出-01_h0t0z0c2x0-1024y0-1024.jpg]

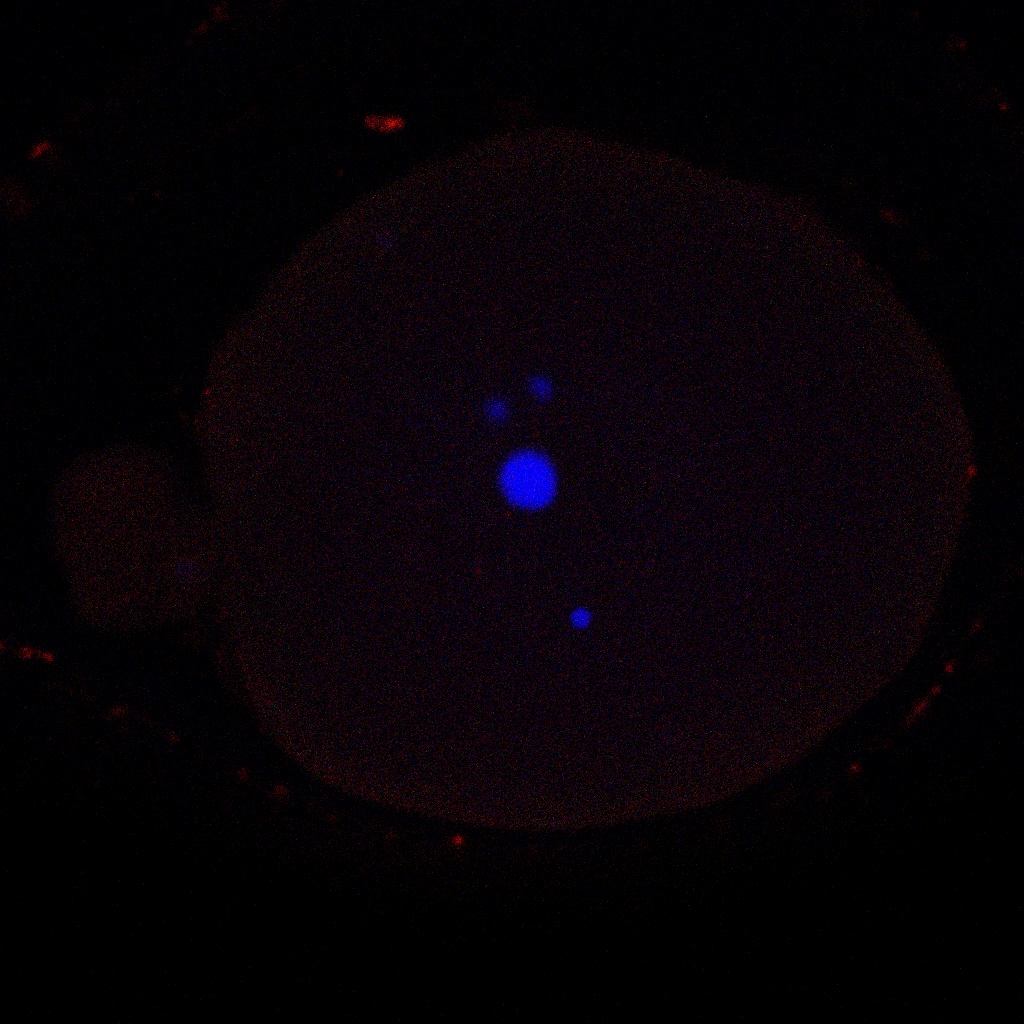

Supplement: Supplementary file 8 — Source Data for Figure 3 [file EMMM-13-e14887-s005.zip › EMM-2021-14887_SDataFig3/Fig. 3A/SiMOS pERK-FITC-4/SiMOS pERK-FITC-4-图像导出-04_h0t0z0c0-3x0-1024y0-1024.jpg]

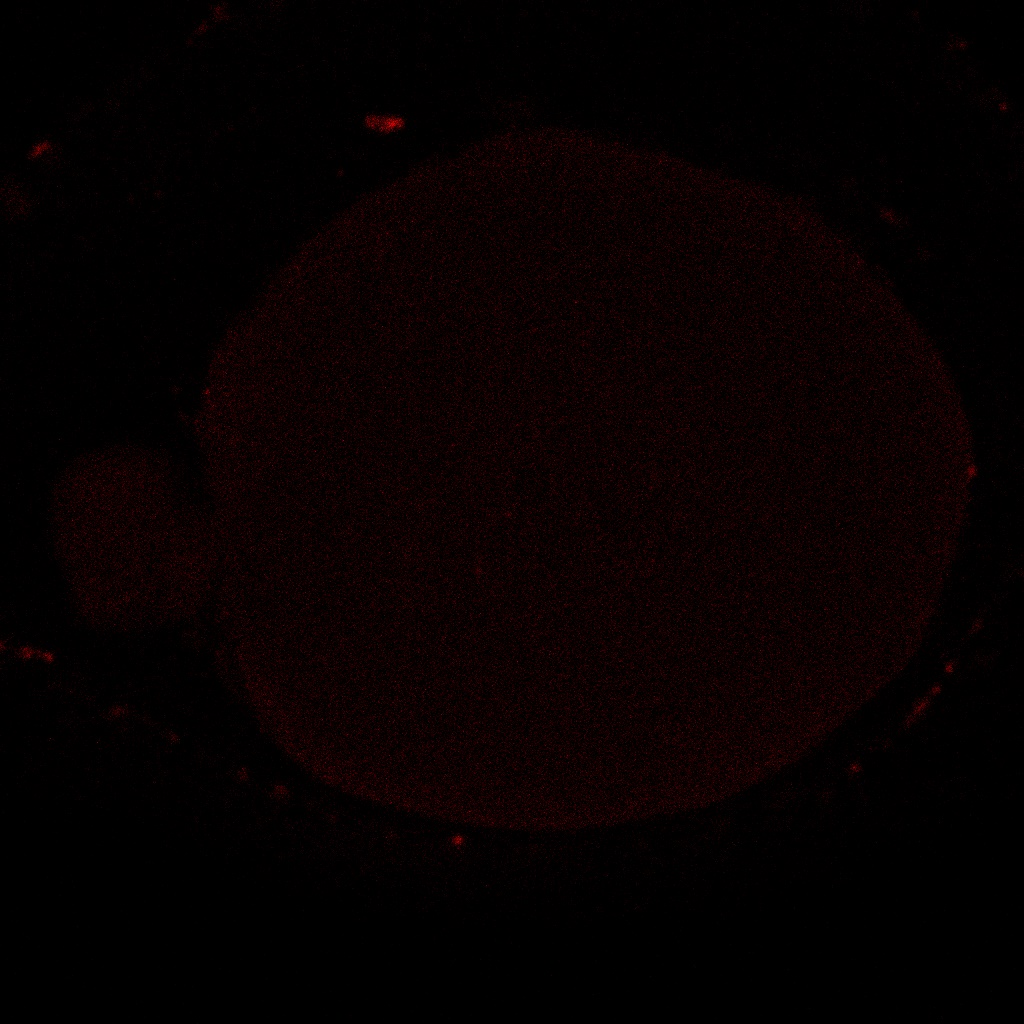

Supplement: Supplementary file 8 — Source Data for Figure 3 [file EMMM-13-e14887-s005.zip › EMM-2021-14887_SDataFig3/Fig. 3A/SiMOS pERK-FITC-4/SiMOS pERK-FITC-4-图像导出-04_h0t0z0c0x0-1024y0-1024.jpg]

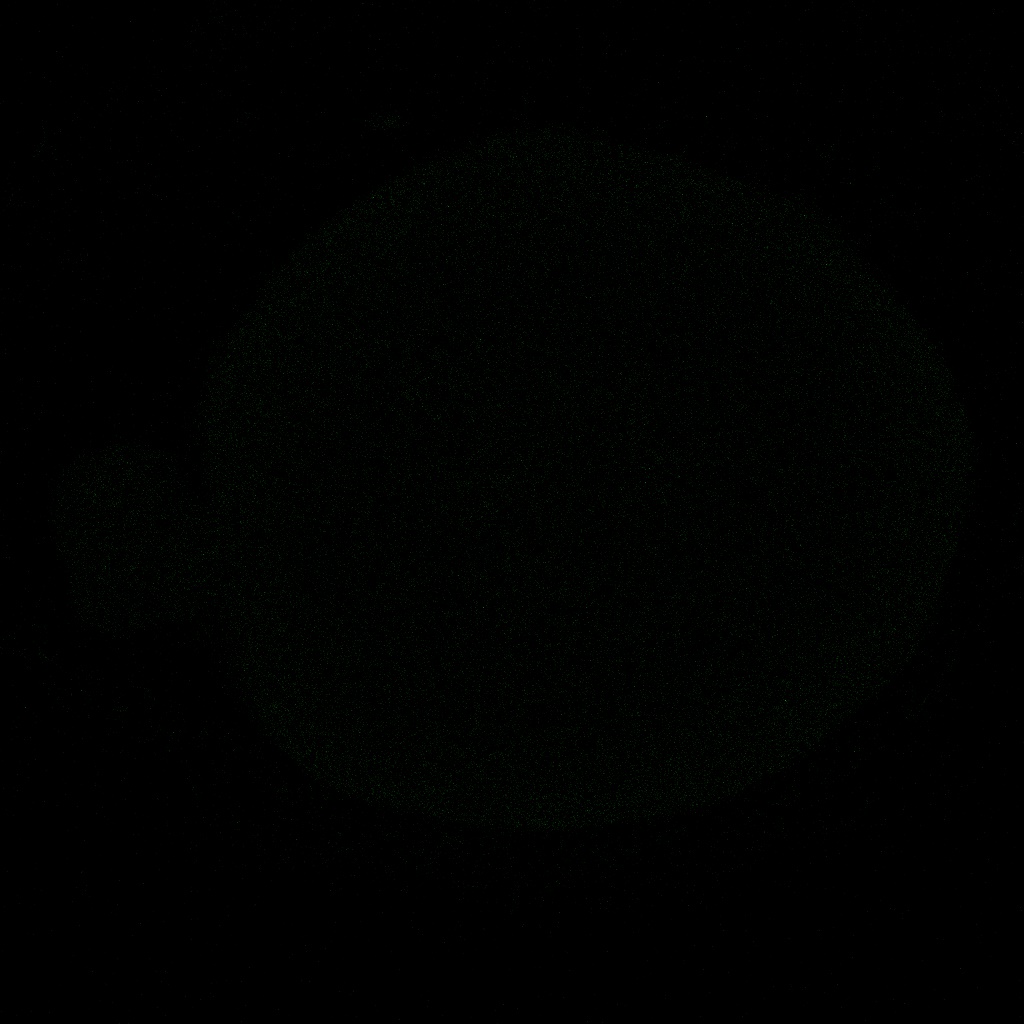

Supplement: Supplementary file 8 — Source Data for Figure 3 [file EMMM-13-e14887-s005.zip › EMM-2021-14887_SDataFig3/Fig. 3A/SiMOS pERK-FITC-4/SiMOS pERK-FITC-4-图像导出-04_h0t0z0c1x0-1024y0-1024.jpg]

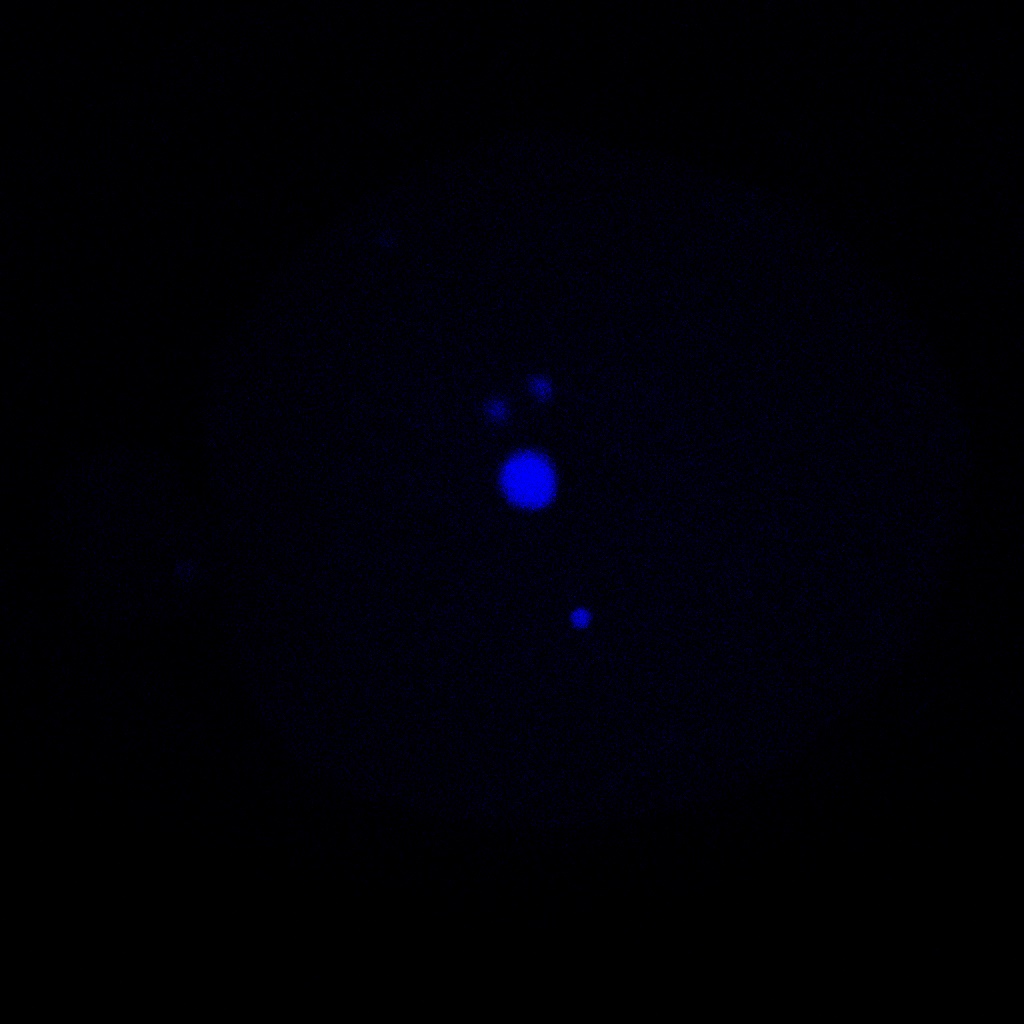

Supplement: Supplementary file 8 — Source Data for Figure 3 [file EMMM-13-e14887-s005.zip › EMM-2021-14887_SDataFig3/Fig. 3A/SiMOS pERK-FITC-4/SiMOS pERK-FITC-4-图像导出-04_h0t0z0c2x0-1024y0-1024.jpg]

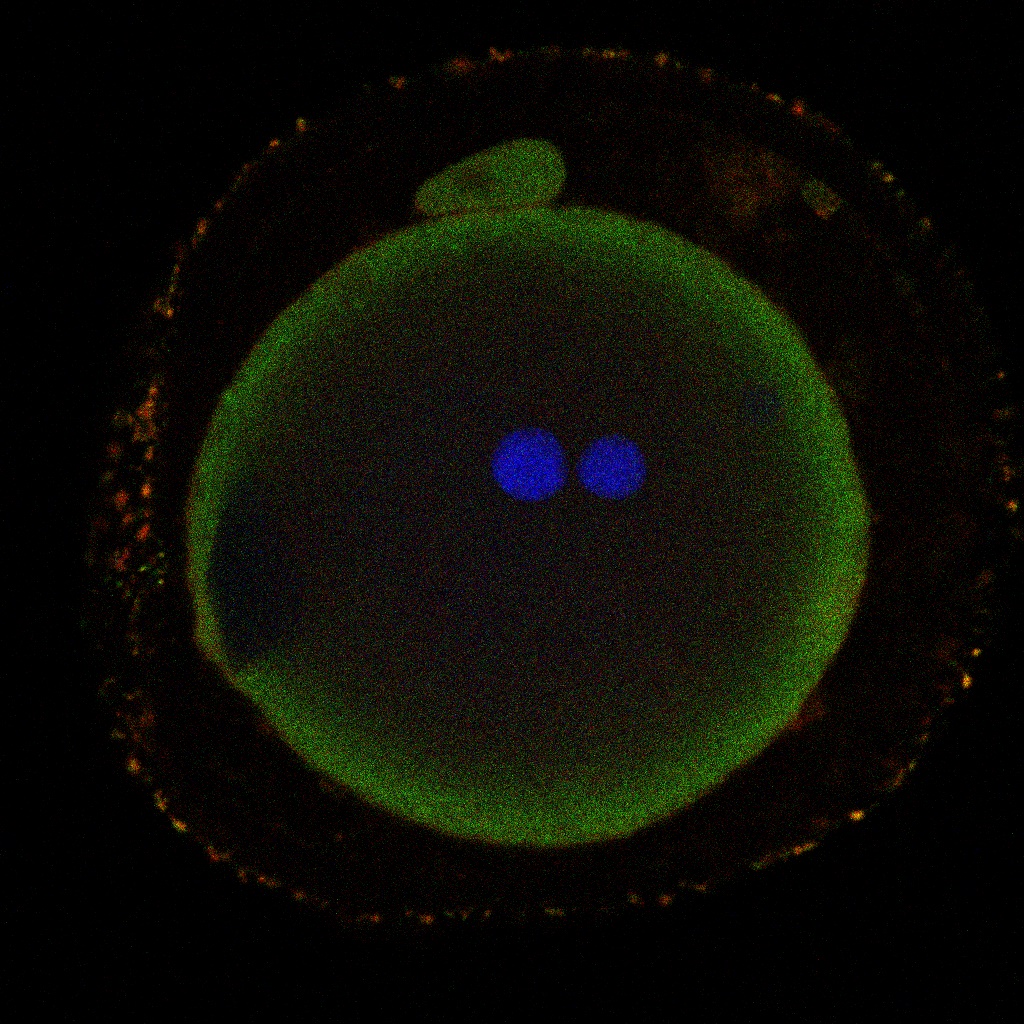

Supplement: Supplementary file 8 — Source Data for Figure 3 [file EMMM-13-e14887-s005.zip › EMM-2021-14887_SDataFig3/Fig. 3A/SiMOS+139-246 pERK-FITC-3/SiMOS+139-246 pERK-FITC-3-图像导出-03_h0t0z0c0-3x0-1024y0-1024.jpg]

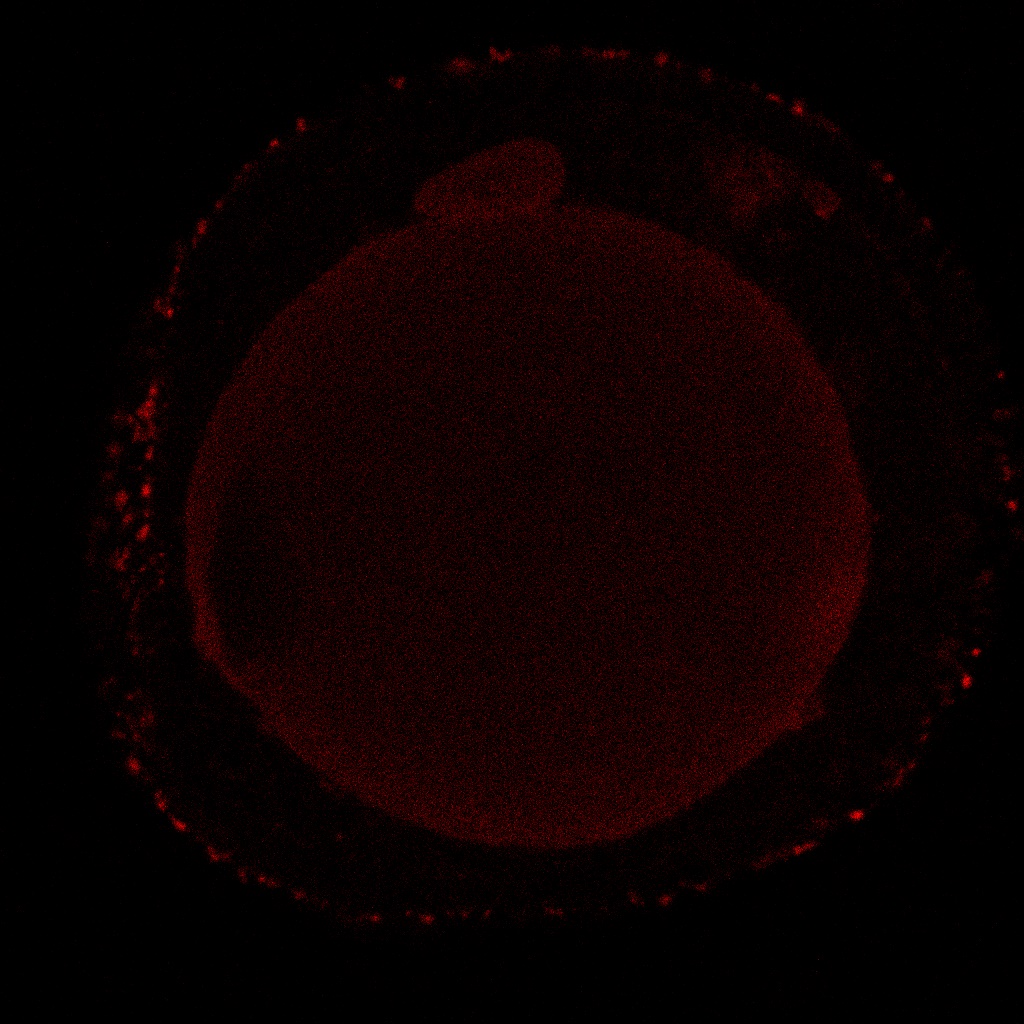

Supplement: Supplementary file 8 — Source Data for Figure 3 [file EMMM-13-e14887-s005.zip › EMM-2021-14887_SDataFig3/Fig. 3A/SiMOS+139-246 pERK-FITC-3/SiMOS+139-246 pERK-FITC-3-图像导出-03_h0t0z0c0x0-1024y0-1024.jpg]

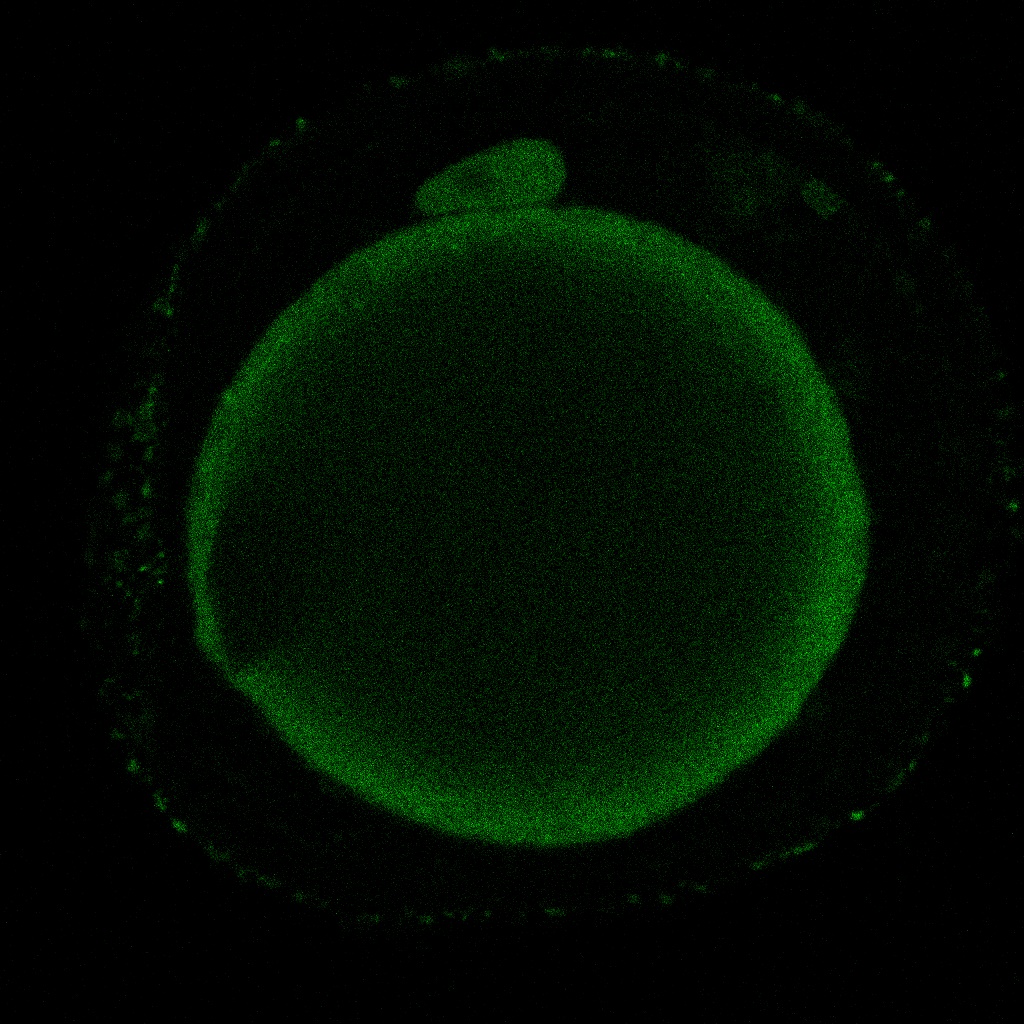

Supplement: Supplementary file 8 — Source Data for Figure 3 [file EMMM-13-e14887-s005.zip › EMM-2021-14887_SDataFig3/Fig. 3A/SiMOS+139-246 pERK-FITC-3/SiMOS+139-246 pERK-FITC-3-图像导出-03_h0t0z0c1x0-1024y0-1024.jpg]

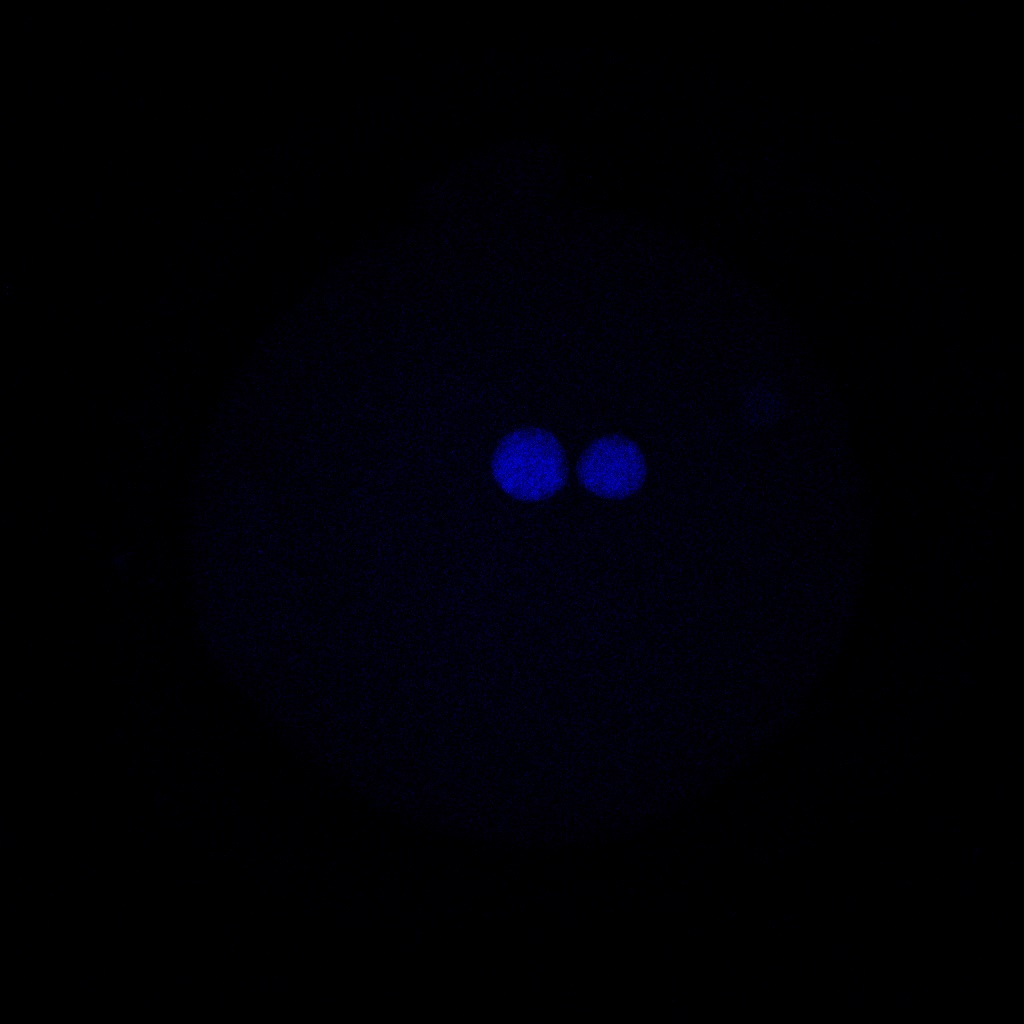

Supplement: Supplementary file 8 — Source Data for Figure 3 [file EMMM-13-e14887-s005.zip › EMM-2021-14887_SDataFig3/Fig. 3A/SiMOS+139-246 pERK-FITC-3/SiMOS+139-246 pERK-FITC-3-图像导出-03_h0t0z0c2x0-1024y0-1024.jpg]

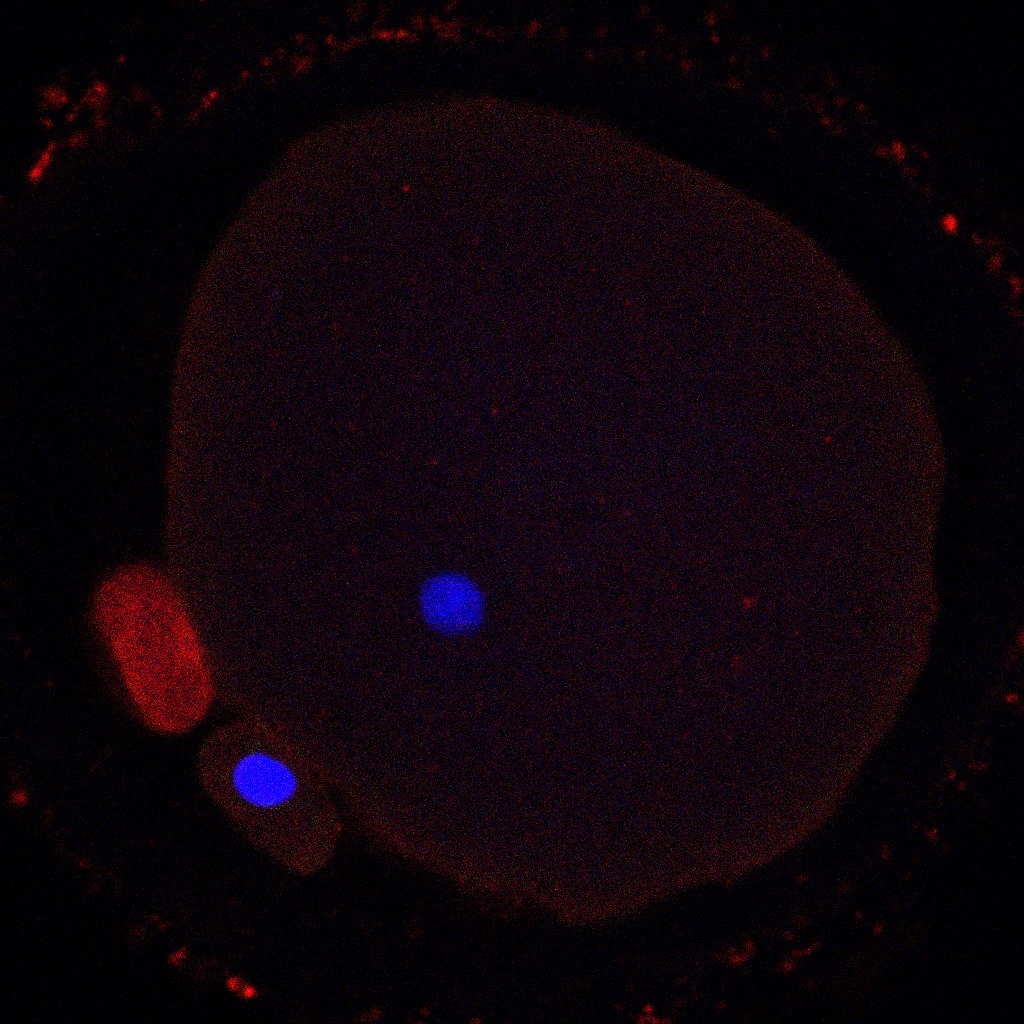

Supplement: Supplementary file 8 — Source Data for Figure 3 [file EMMM-13-e14887-s005.zip › EMM-2021-14887_SDataFig3/Fig. 3A/SiMOS+95 pERK-FITC-06/SiMOS+95 pERK-FITC-6-图像导出-06_h0t0z0c0-3x0-1024y0-1024.jpg]

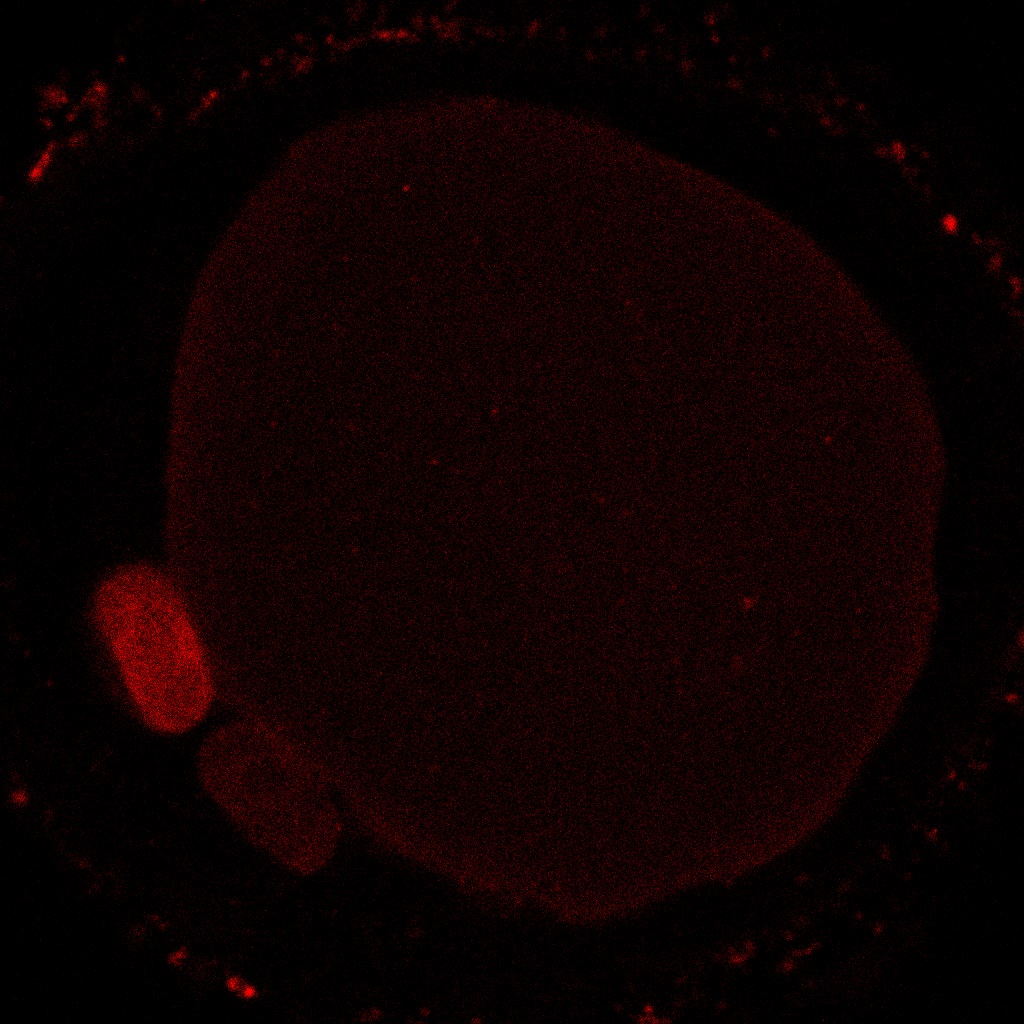

Supplement: Supplementary file 8 — Source Data for Figure 3 [file EMMM-13-e14887-s005.zip › EMM-2021-14887_SDataFig3/Fig. 3A/SiMOS+95 pERK-FITC-06/SiMOS+95 pERK-FITC-6-图像导出-06_h0t0z0c0x0-1024y0-1024.jpg]

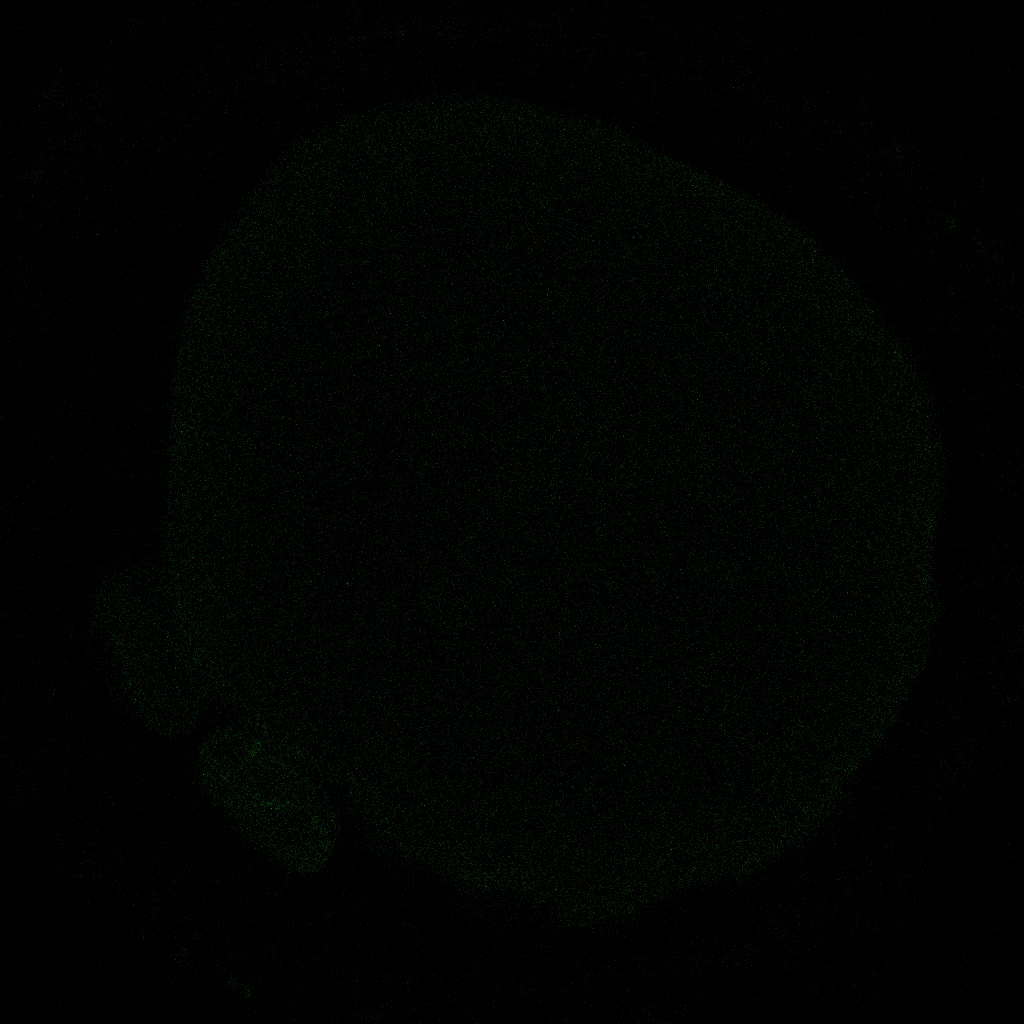

Supplement: Supplementary file 8 — Source Data for Figure 3 [file EMMM-13-e14887-s005.zip › EMM-2021-14887_SDataFig3/Fig. 3A/SiMOS+95 pERK-FITC-06/SiMOS+95 pERK-FITC-6-图像导出-06_h0t0z0c1x0-1024y0-1024.jpg]

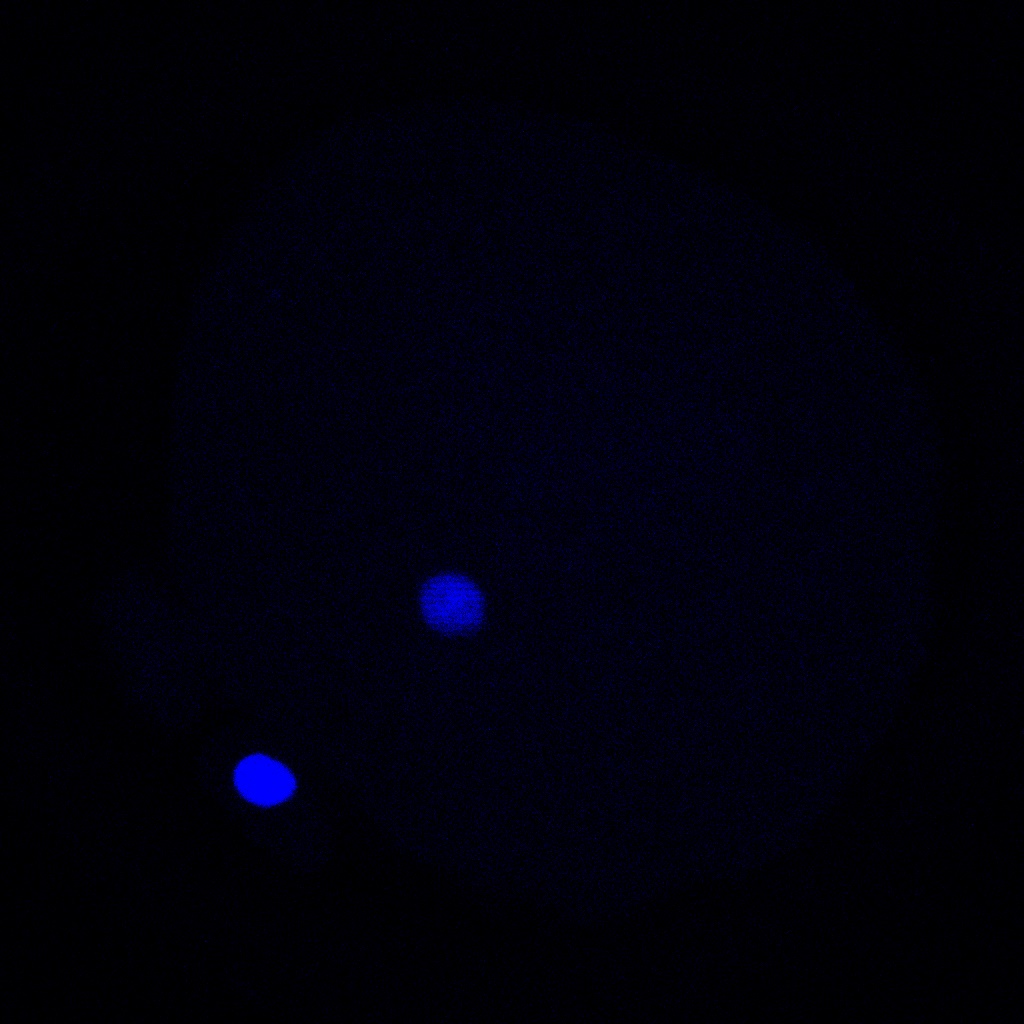

Supplement: Supplementary file 8 — Source Data for Figure 3 [file EMMM-13-e14887-s005.zip › EMM-2021-14887_SDataFig3/Fig. 3A/SiMOS+95 pERK-FITC-06/SiMOS+95 pERK-FITC-6-图像导出-06_h0t0z0c2x0-1024y0-1024.jpg]

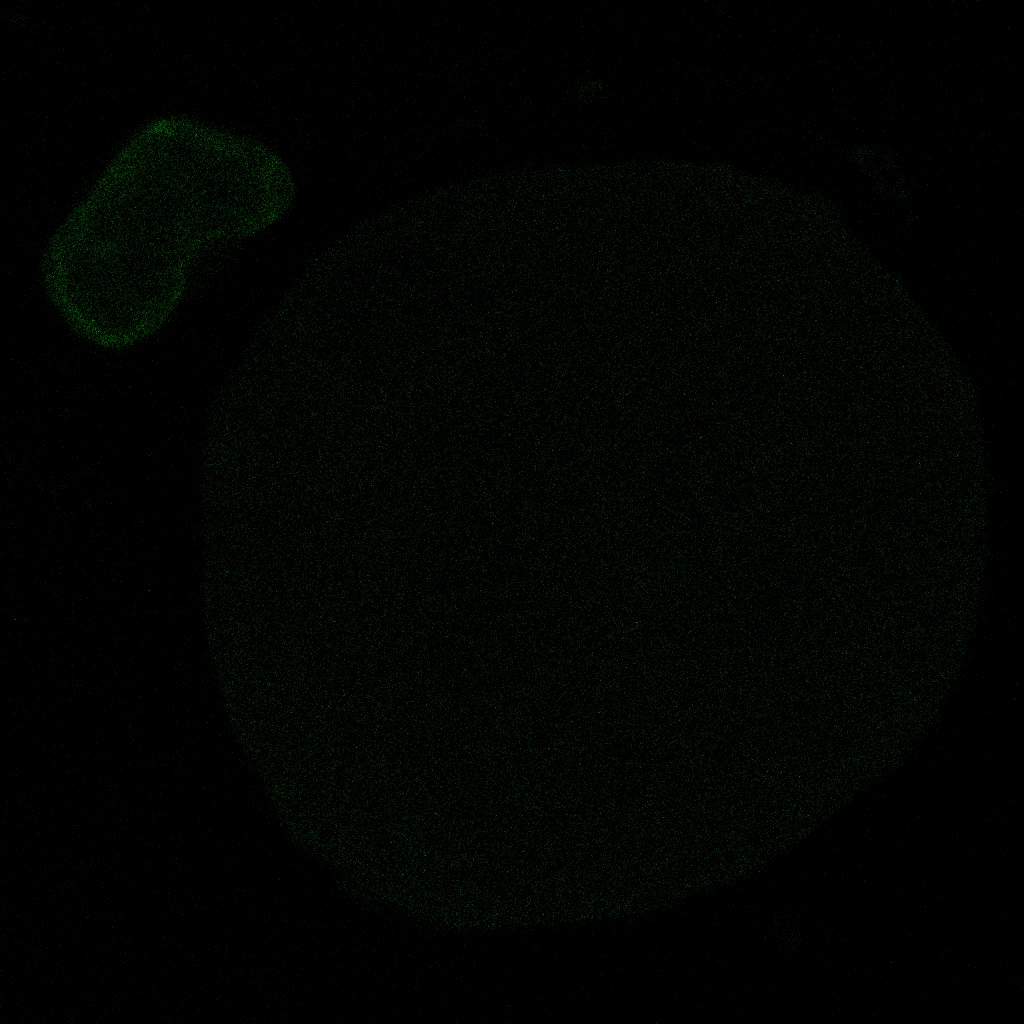

Supplement: Supplementary file 8 — Source Data for Figure 3 [file EMMM-13-e14887-s005.zip › EMM-2021-14887_SDataFig3/Fig. 3A/SiMOS+C320Ter pERK-FITC-13/SiMOS+ C320Ter pERK-FITC-13-图像导出-13_h0t0z0c1x0-1024y0-1024.jpg]

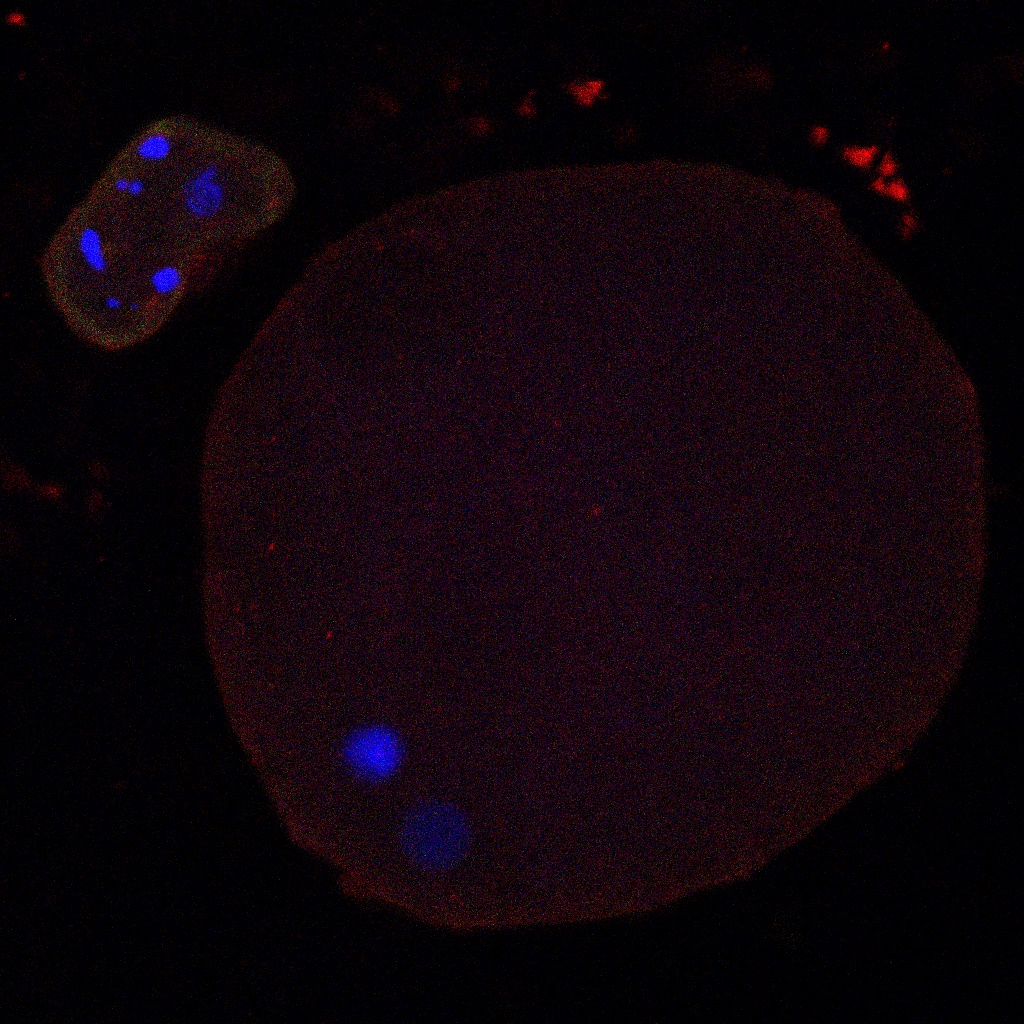

Supplement: Supplementary file 8 — Source Data for Figure 3 [file EMMM-13-e14887-s005.zip › EMM-2021-14887_SDataFig3/Fig. 3A/SiMOS+C320Ter pERK-FITC-13/SiMOS+C320Ter pERK-FITC-13-图像导出-13_h0t0z0c0-3x0-1024y0-1024.jpg]

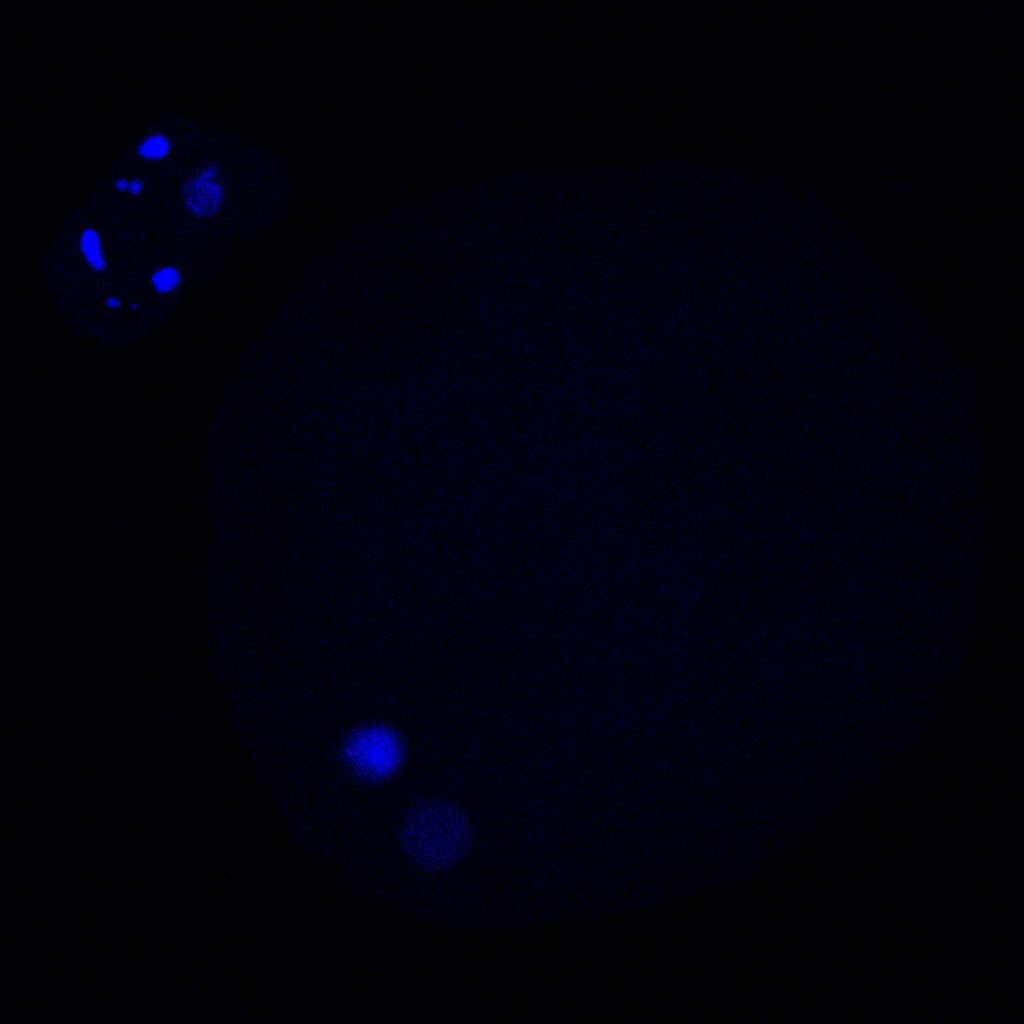

Supplement: Supplementary file 8 — Source Data for Figure 3 [file EMMM-13-e14887-s005.zip › EMM-2021-14887_SDataFig3/Fig. 3A/SiMOS+C320Ter pERK-FITC-13/SiMOS+C320Ter pERK-FITC-13-图像导出-13_h0t0z0c2x0-1024y0-1024.jpg]

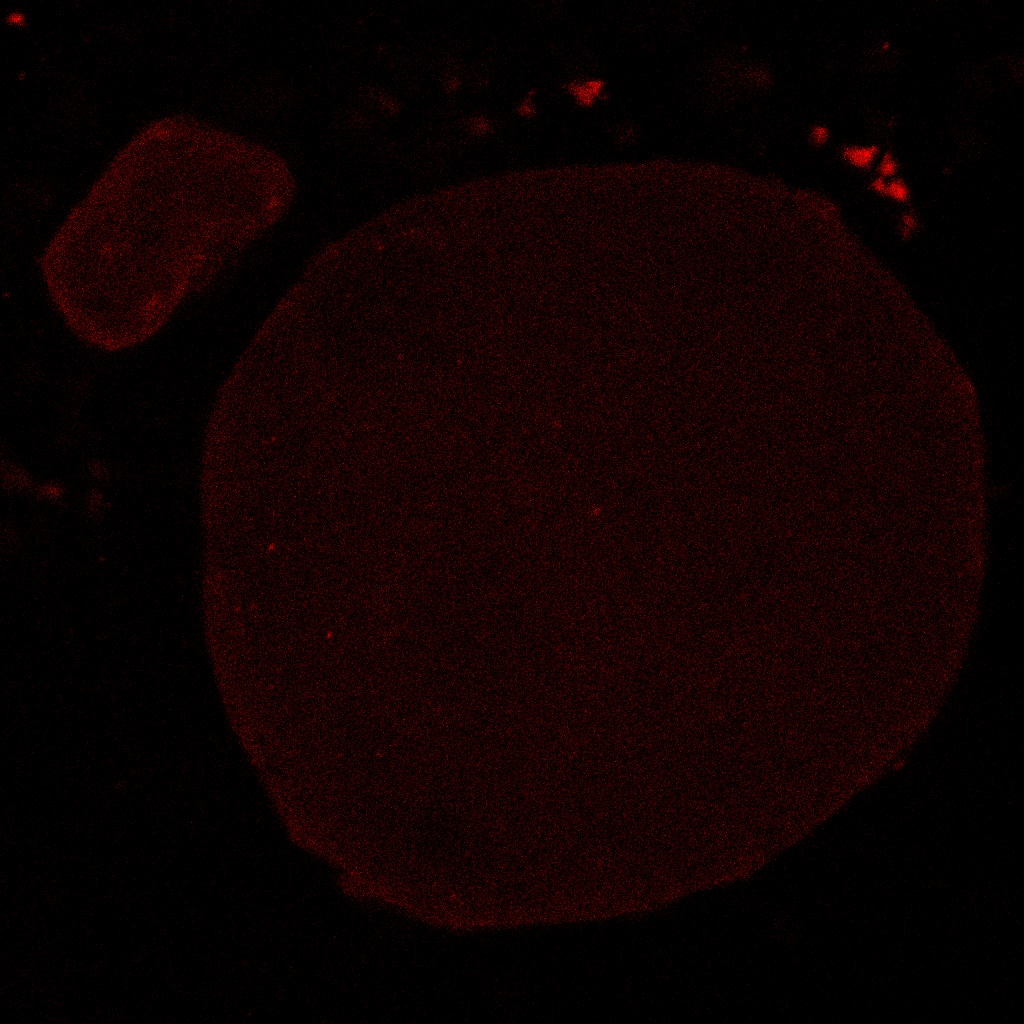

Supplement: Supplementary file 8 — Source Data for Figure 3 [file EMMM-13-e14887-s005.zip › EMM-2021-14887_SDataFig3/Fig. 3A/SiMOS+C320Ter pERK-FITC-13/SiMOS+C320Ter pERK-FITC-131024.jpg]

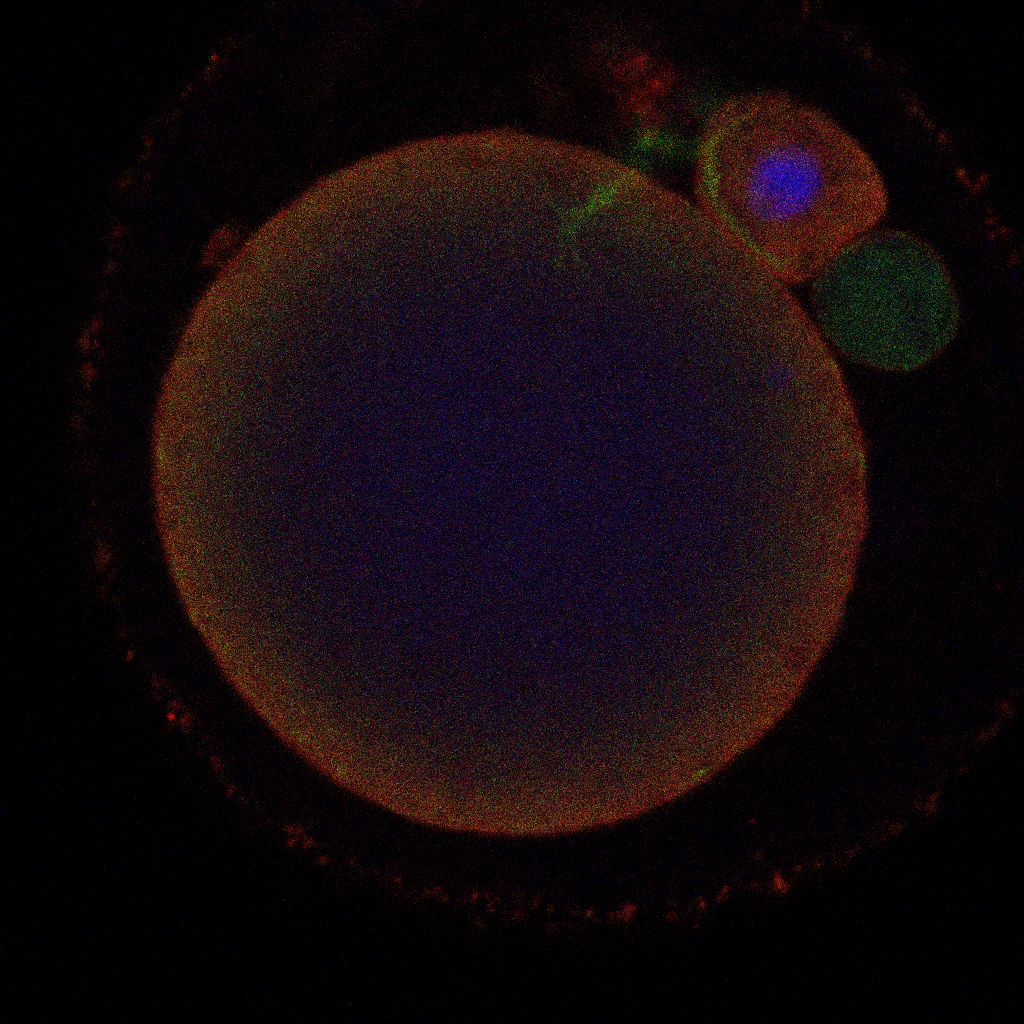

Supplement: Supplementary file 8 — Source Data for Figure 3 [file EMMM-13-e14887-s005.zip › EMM-2021-14887_SDataFig3/Fig. 3A/SiMOS+MOS pERK-FITC-3/SiMOS+MOS pERK-FITC-3-图像导出-03_h0t0z0c0-3x0-1024y0-1024.jpg]

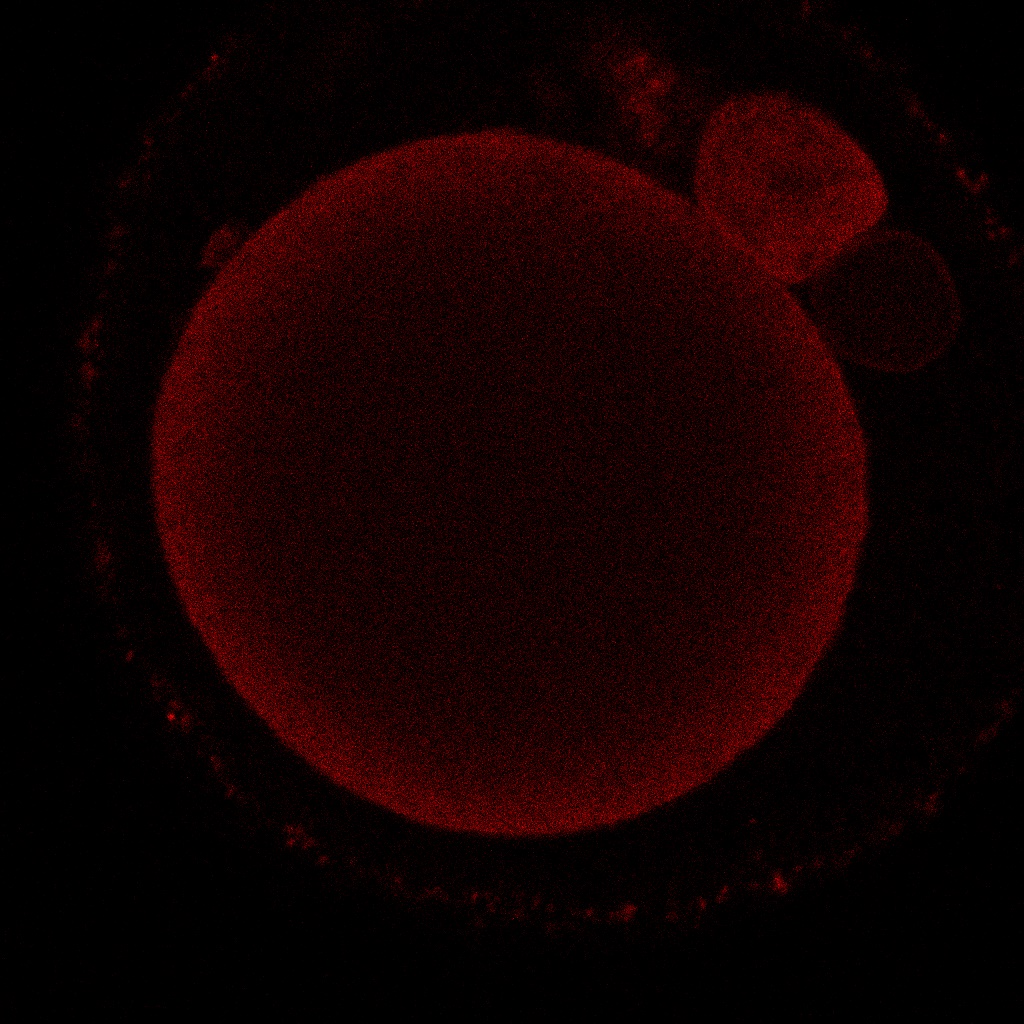

Supplement: Supplementary file 8 — Source Data for Figure 3 [file EMMM-13-e14887-s005.zip › EMM-2021-14887_SDataFig3/Fig. 3A/SiMOS+MOS pERK-FITC-3/SiMOS+MOS pERK-FITC-3-图像导出-03_h0t0z0c0x0-1024y0-1024.jpg]

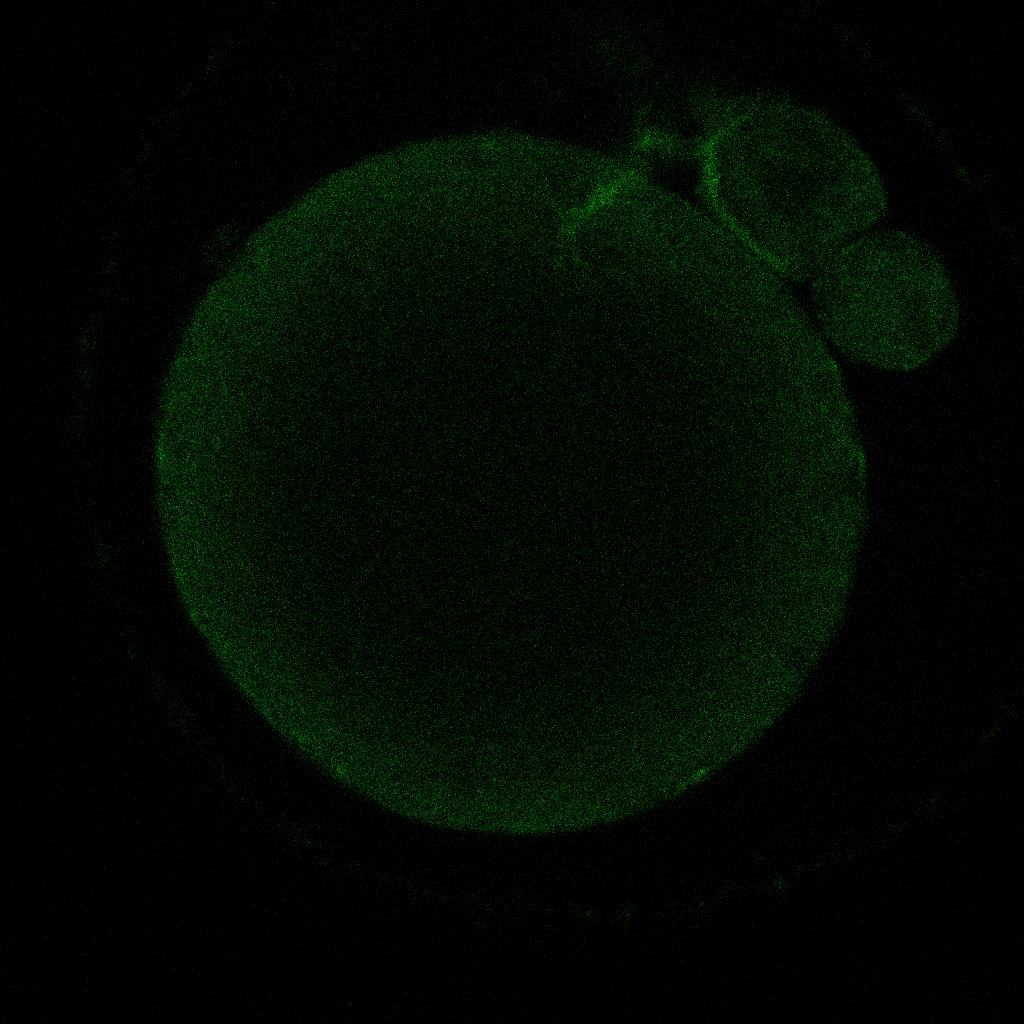

Supplement: Supplementary file 8 — Source Data for Figure 3 [file EMMM-13-e14887-s005.zip › EMM-2021-14887_SDataFig3/Fig. 3A/SiMOS+MOS pERK-FITC-3/SiMOS+MOS pERK-FITC-3-图像导出-03_h0t0z0c1x0-1024y0-1024.jpg]

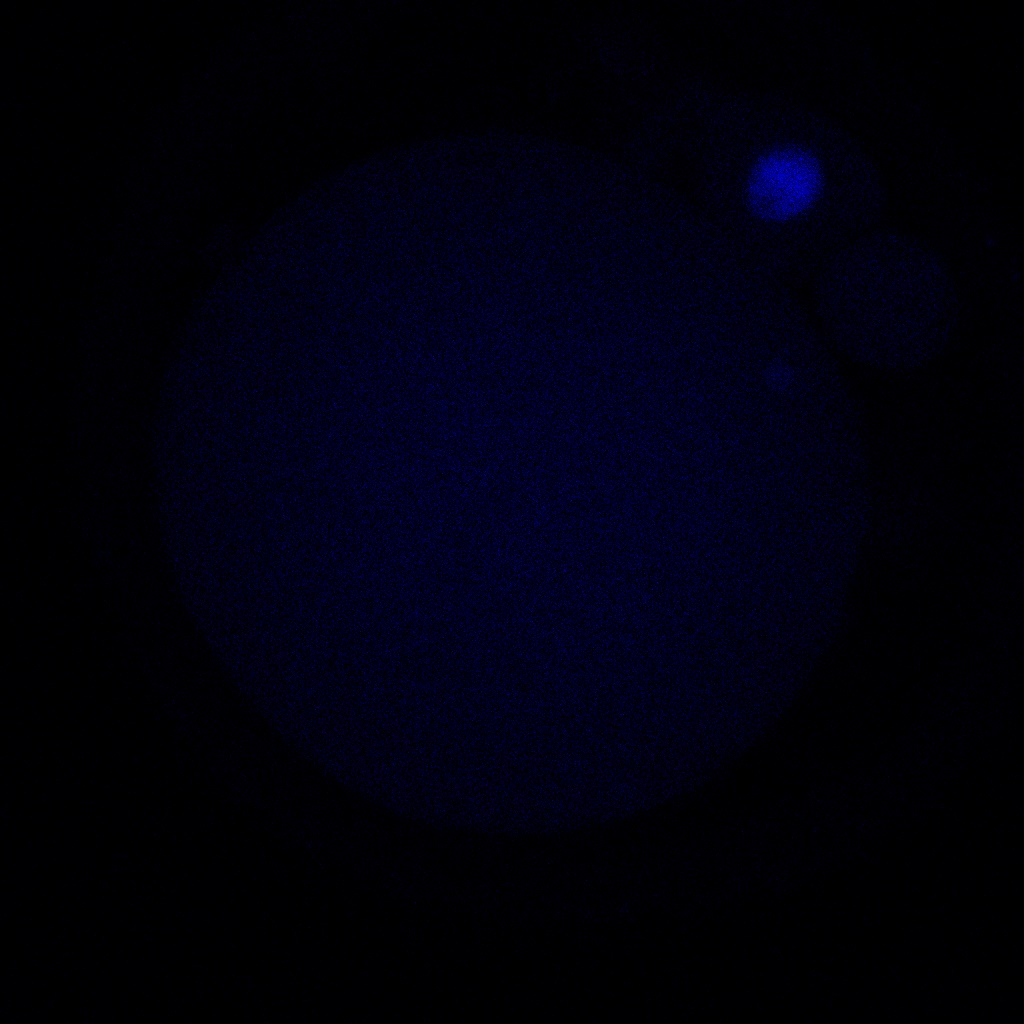

Supplement: Supplementary file 8 — Source Data for Figure 3 [file EMMM-13-e14887-s005.zip › EMM-2021-14887_SDataFig3/Fig. 3A/SiMOS+MOS pERK-FITC-3/SiMOS+MOS pERK-FITC-3-图像导出-03_h0t0z0c2x0-1024y0-1024.jpg]

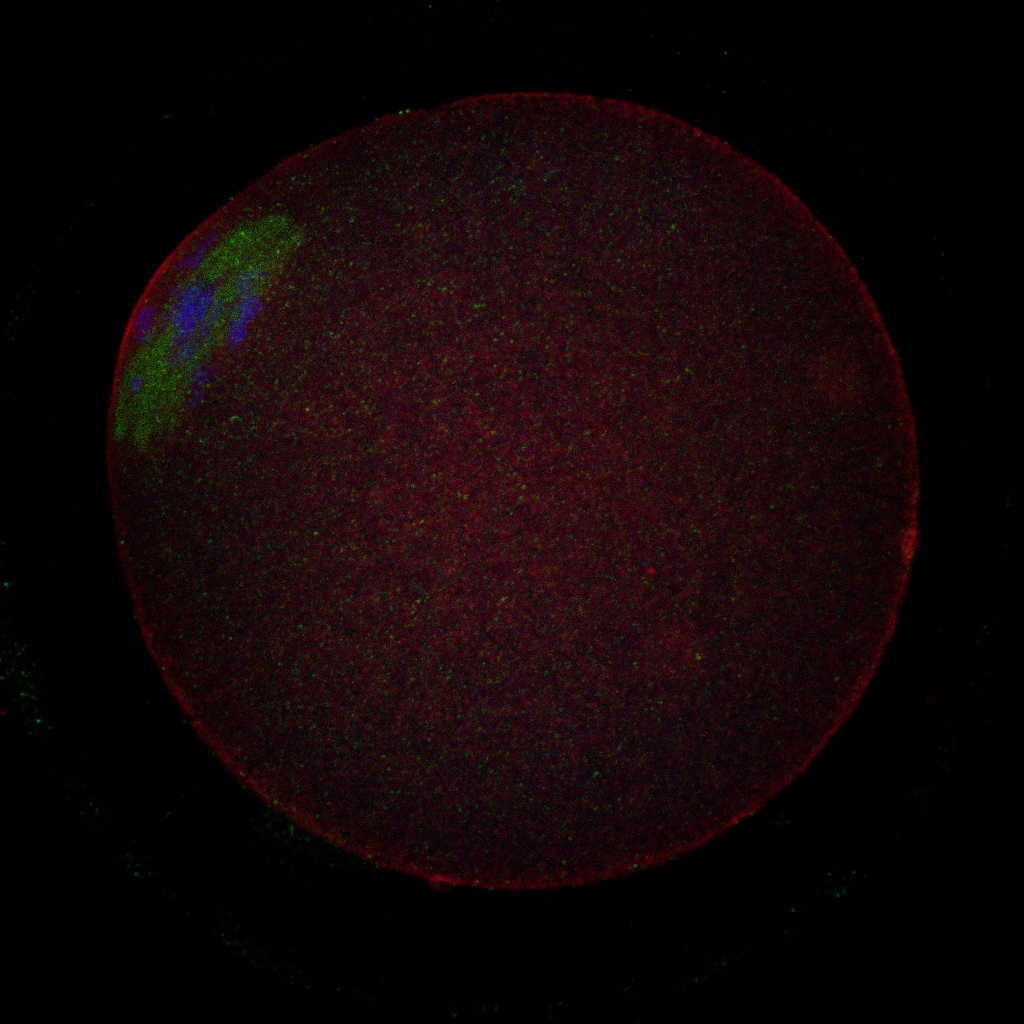

Supplement: Supplementary file 8 — Source Data for Figure 3 [file EMMM-13-e14887-s005.zip › EMM-2021-14887_SDataFig3/Fig. 3C Images/erk12 cko-phalluidin-tpx1-40x-9-Image Export-09_c1-3.jpg]

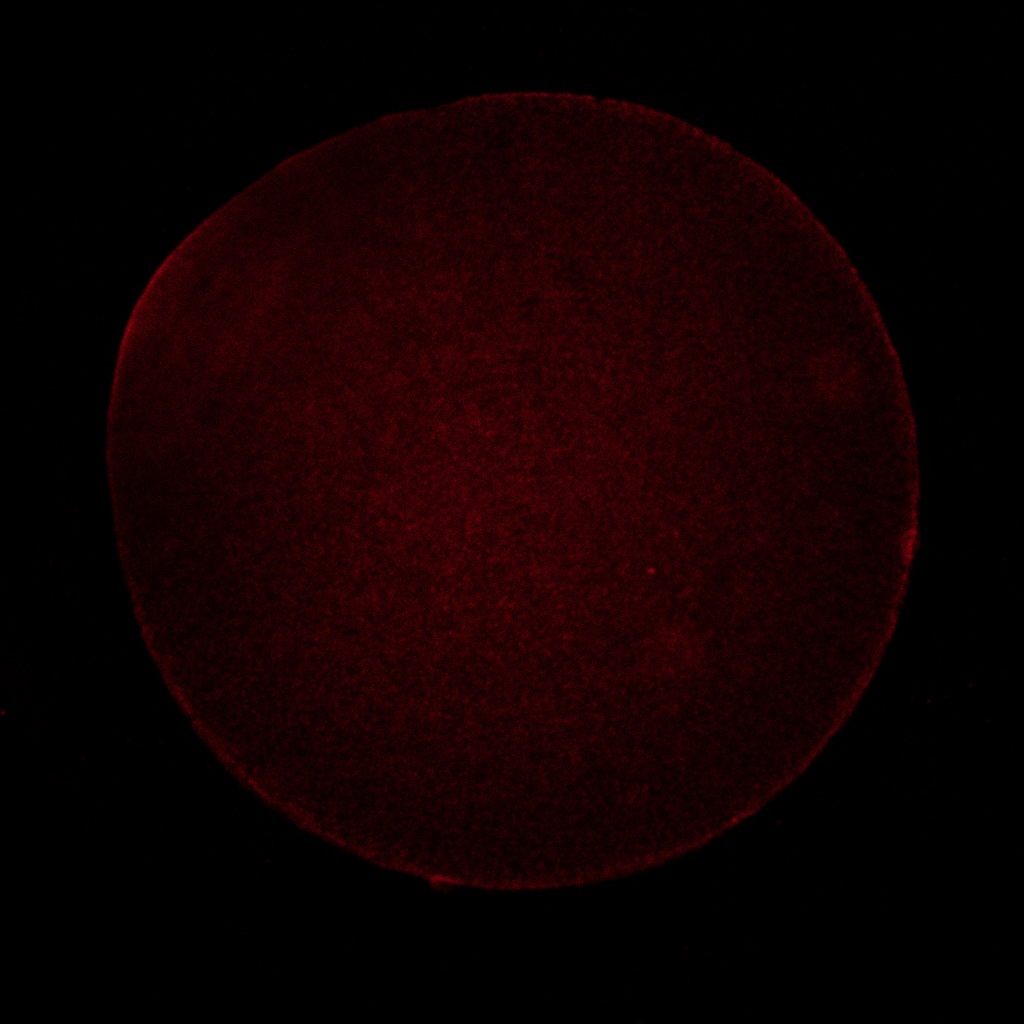

Supplement: Supplementary file 8 — Source Data for Figure 3 [file EMMM-13-e14887-s005.zip › EMM-2021-14887_SDataFig3/Fig. 3C Images/erk12 cko-phalluidin-tpx1-40x-9-Image Export-09_c1.jpg]

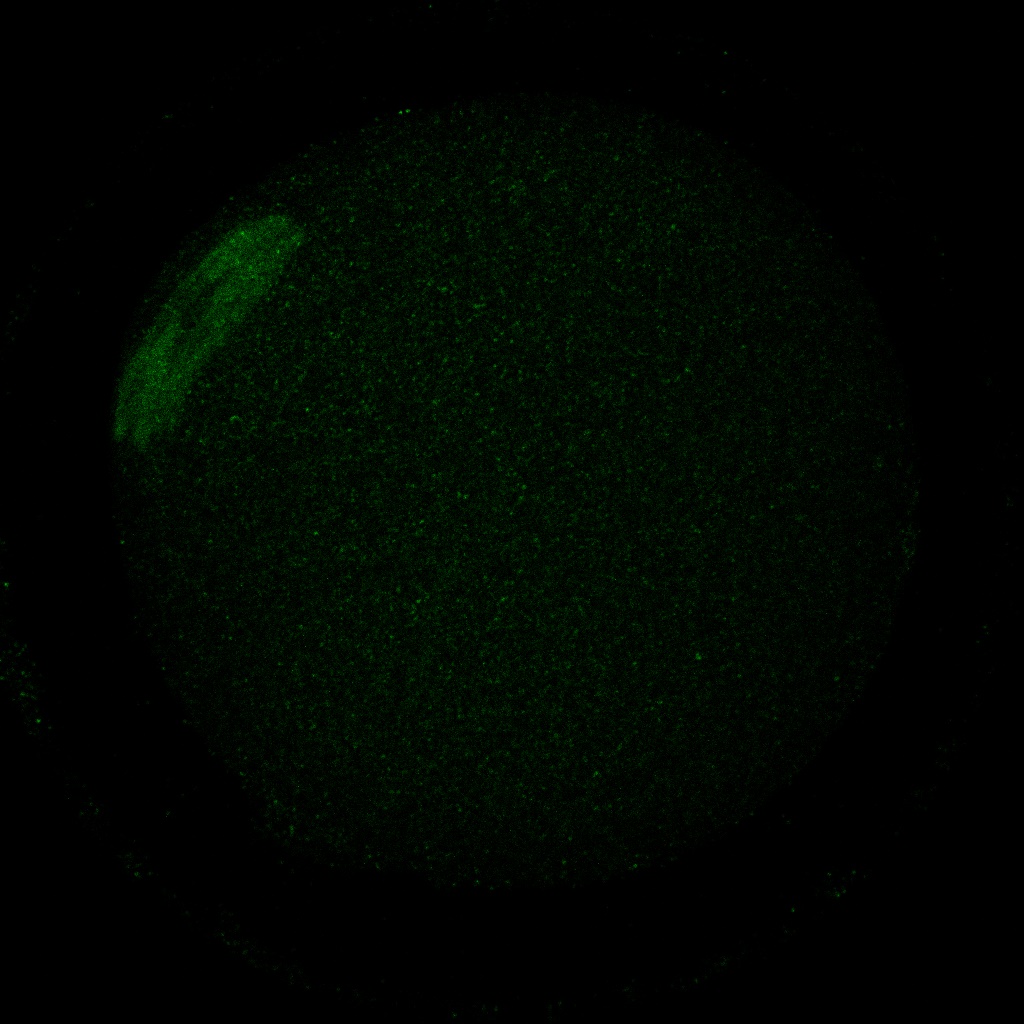

Supplement: Supplementary file 8 — Source Data for Figure 3 [file EMMM-13-e14887-s005.zip › EMM-2021-14887_SDataFig3/Fig. 3C Images/erk12 cko-phalluidin-tpx1-40x-9-Image Export-09_c2.jpg]

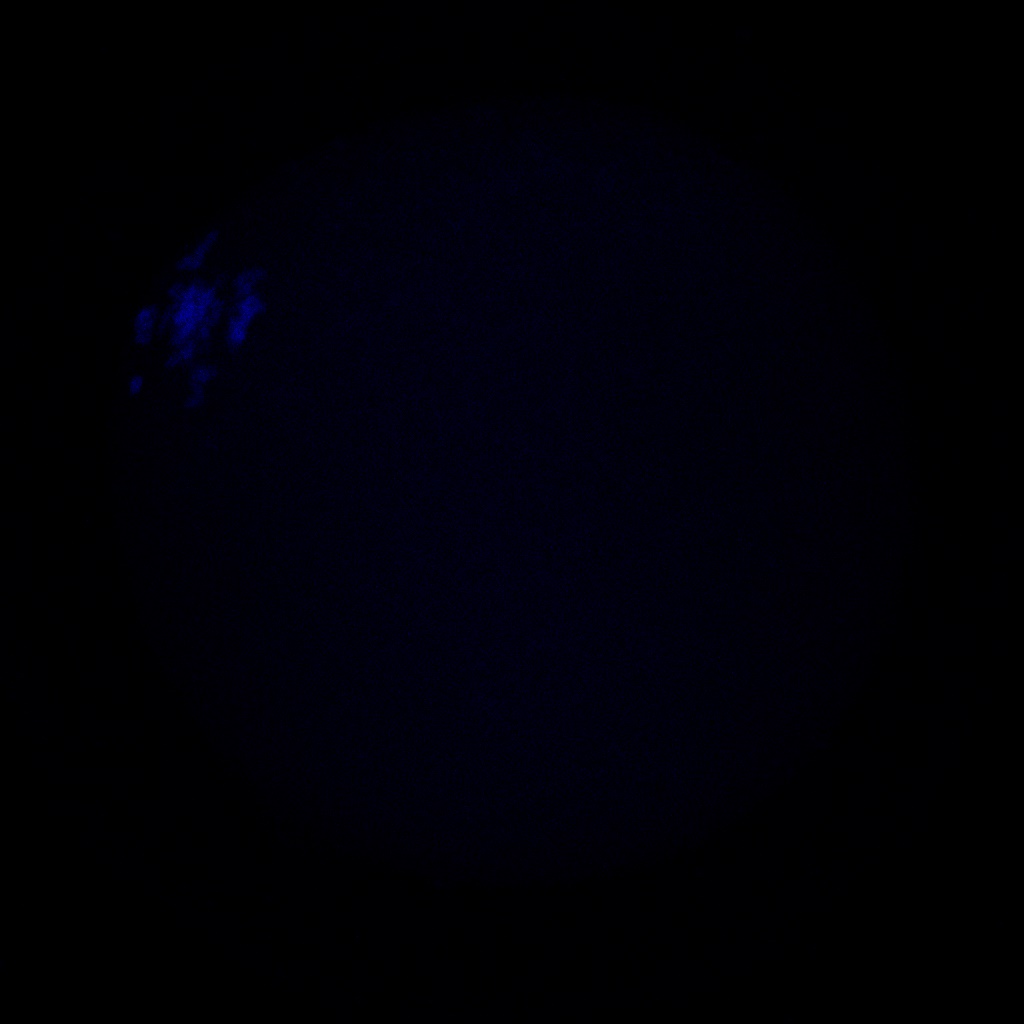

Supplement: Supplementary file 8 — Source Data for Figure 3 [file EMMM-13-e14887-s005.zip › EMM-2021-14887_SDataFig3/Fig. 3C Images/erk12 cko-phalluidin-tpx1-40x-9-Image Export-09_c3.jpg]

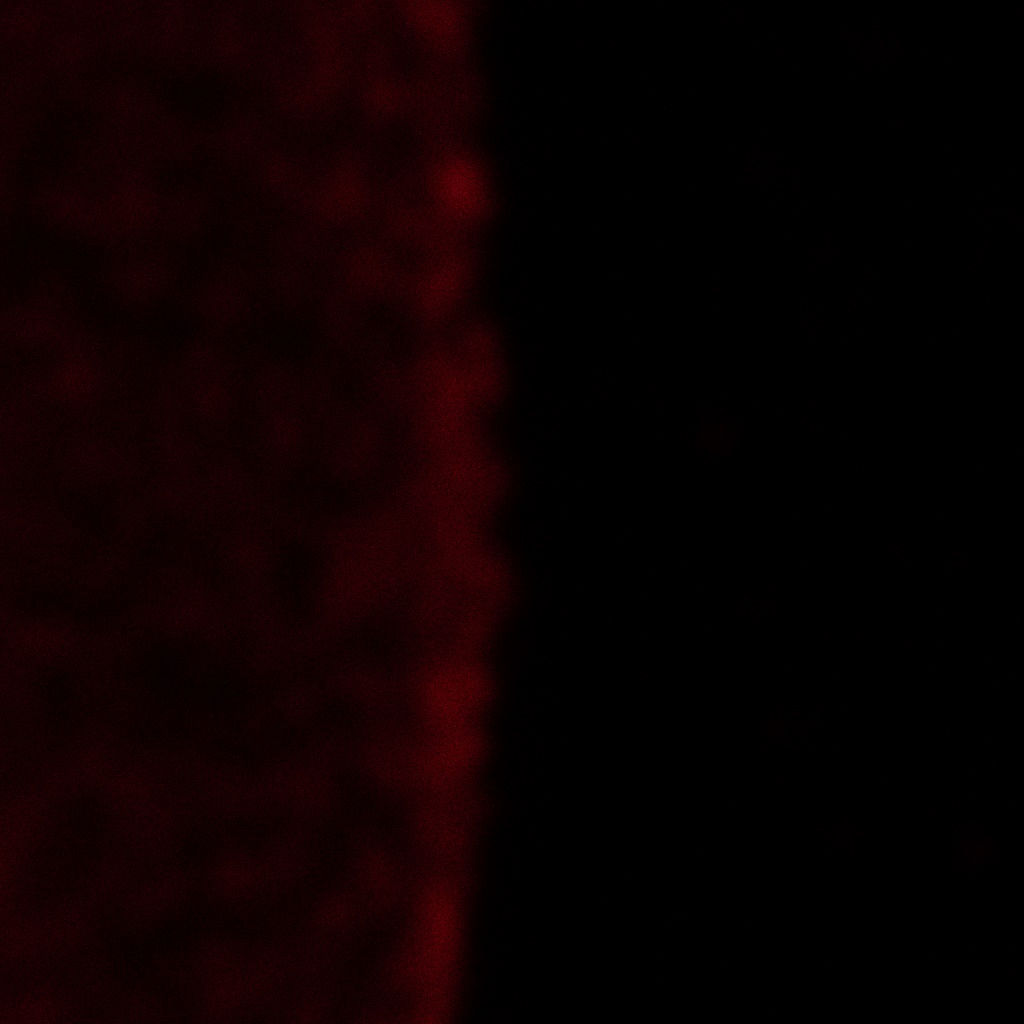

Supplement: Supplementary file 8 — Source Data for Figure 3 [file EMMM-13-e14887-s005.zip › EMM-2021-14887_SDataFig3/Fig. 3C Images/erk12 cko-phalluidin-tpx1-40x-zoom-9-Image Export-20.jpg]

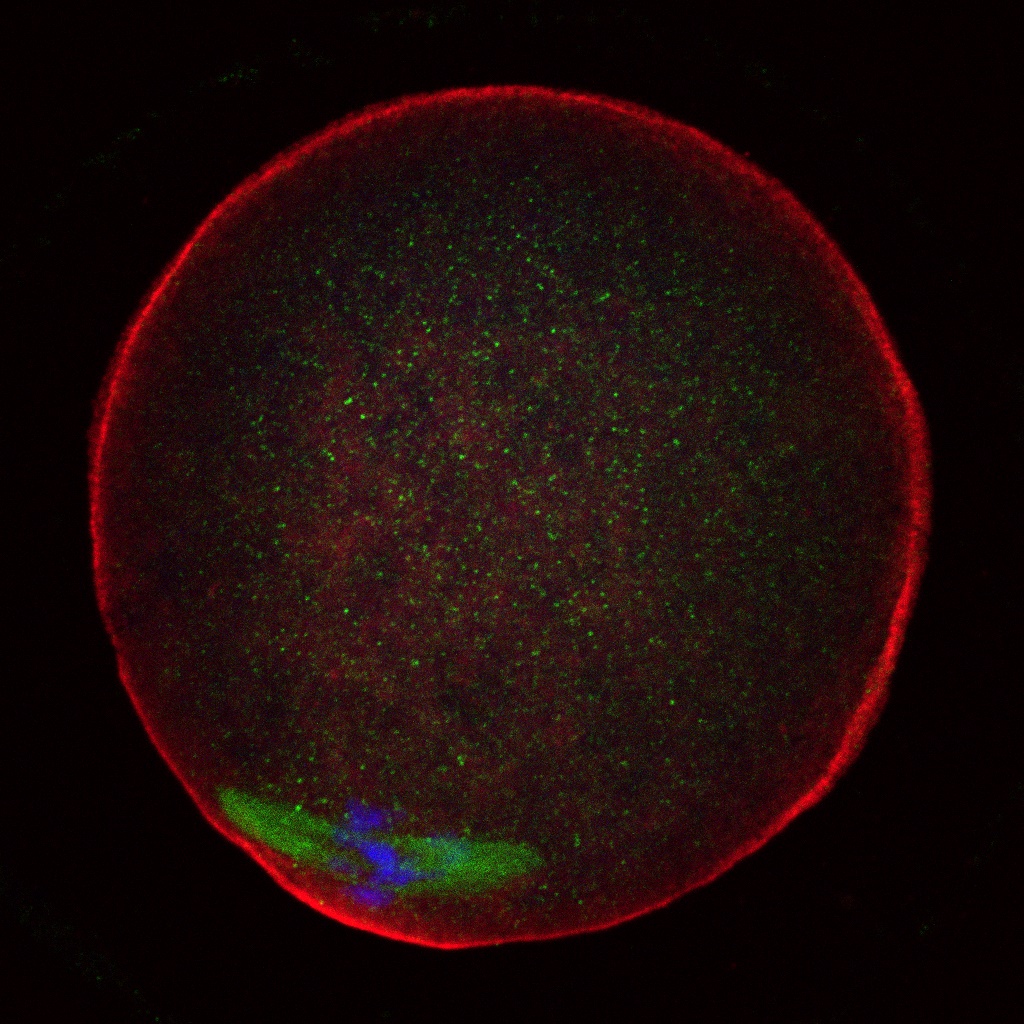

Supplement: Supplementary file 8 — Source Data for Figure 3 [file EMMM-13-e14887-s005.zip › EMM-2021-14887_SDataFig3/Fig. 3C Images/erk12flox-phalluidin-tpx1-40x-2-Image Export-25_c1-3.jpg]

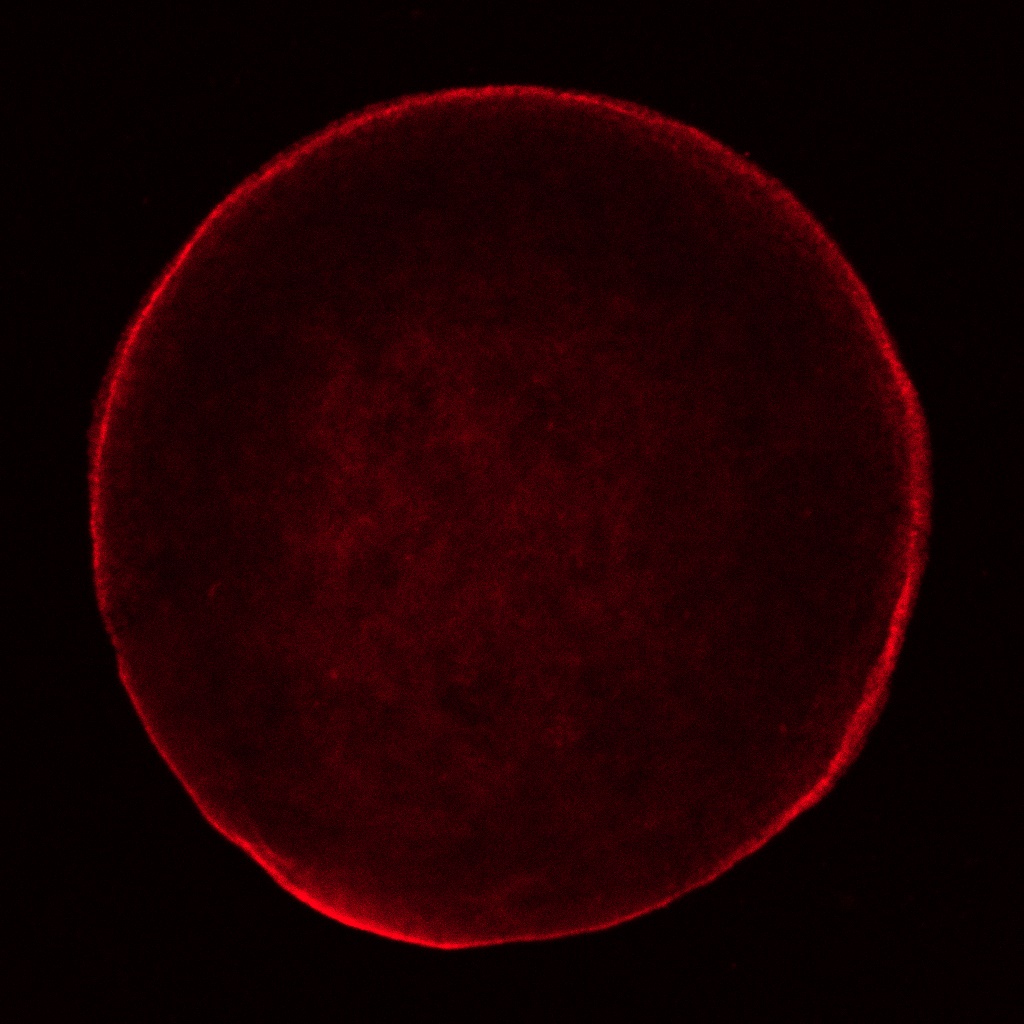

Supplement: Supplementary file 8 — Source Data for Figure 3 [file EMMM-13-e14887-s005.zip › EMM-2021-14887_SDataFig3/Fig. 3C Images/erk12flox-phalluidin-tpx1-40x-2-Image Export-25_c1.jpg]

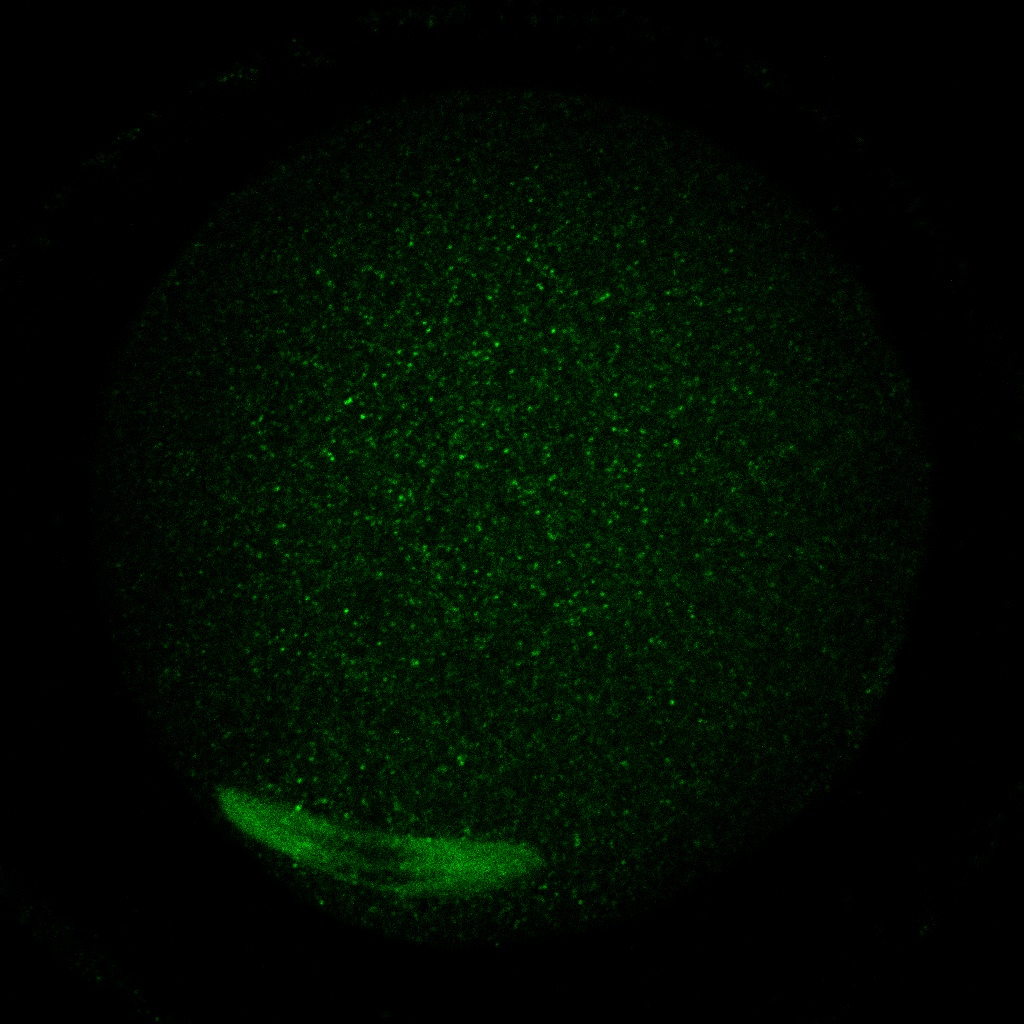

Supplement: Supplementary file 8 — Source Data for Figure 3 [file EMMM-13-e14887-s005.zip › EMM-2021-14887_SDataFig3/Fig. 3C Images/erk12flox-phalluidin-tpx1-40x-2-Image Export-25_c2.jpg]

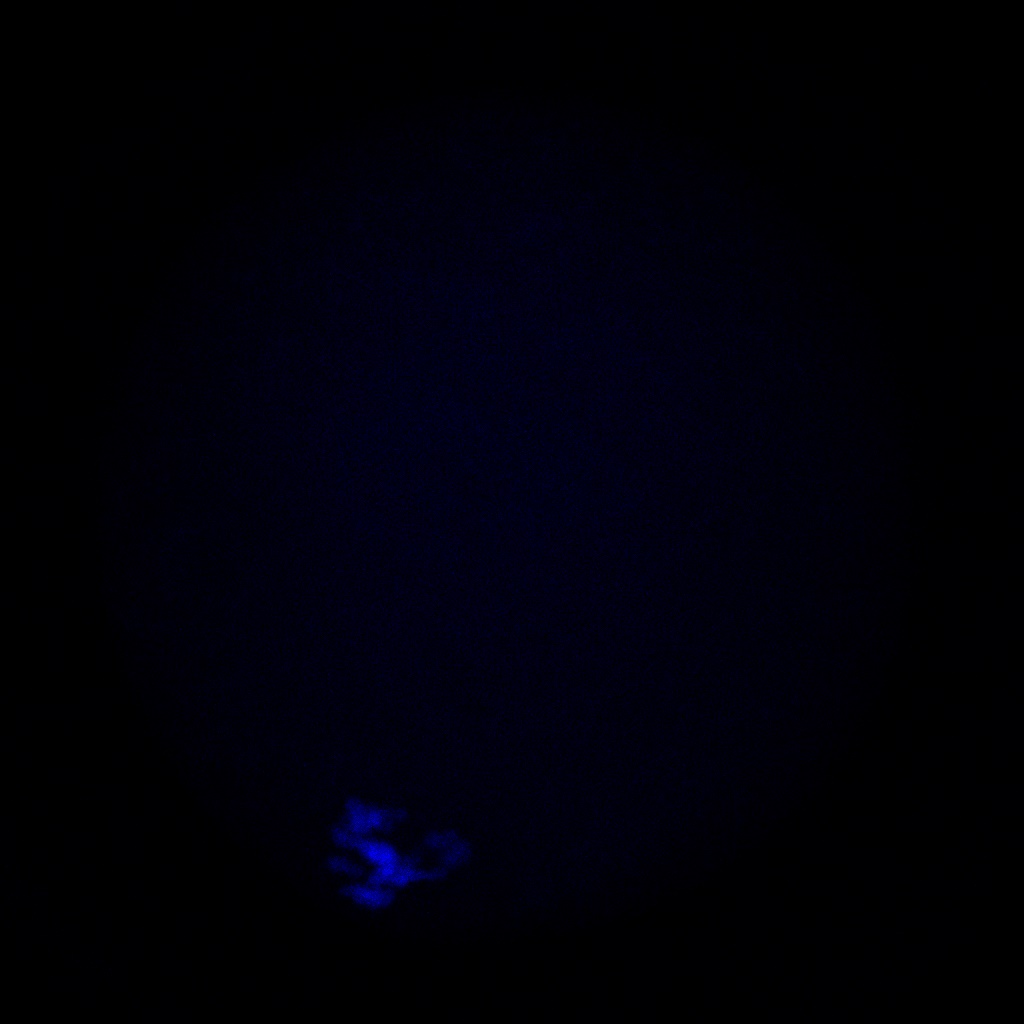

Supplement: Supplementary file 8 — Source Data for Figure 3 [file EMMM-13-e14887-s005.zip › EMM-2021-14887_SDataFig3/Fig. 3C Images/erk12flox-phalluidin-tpx1-40x-2-Image Export-25_c3.jpg]

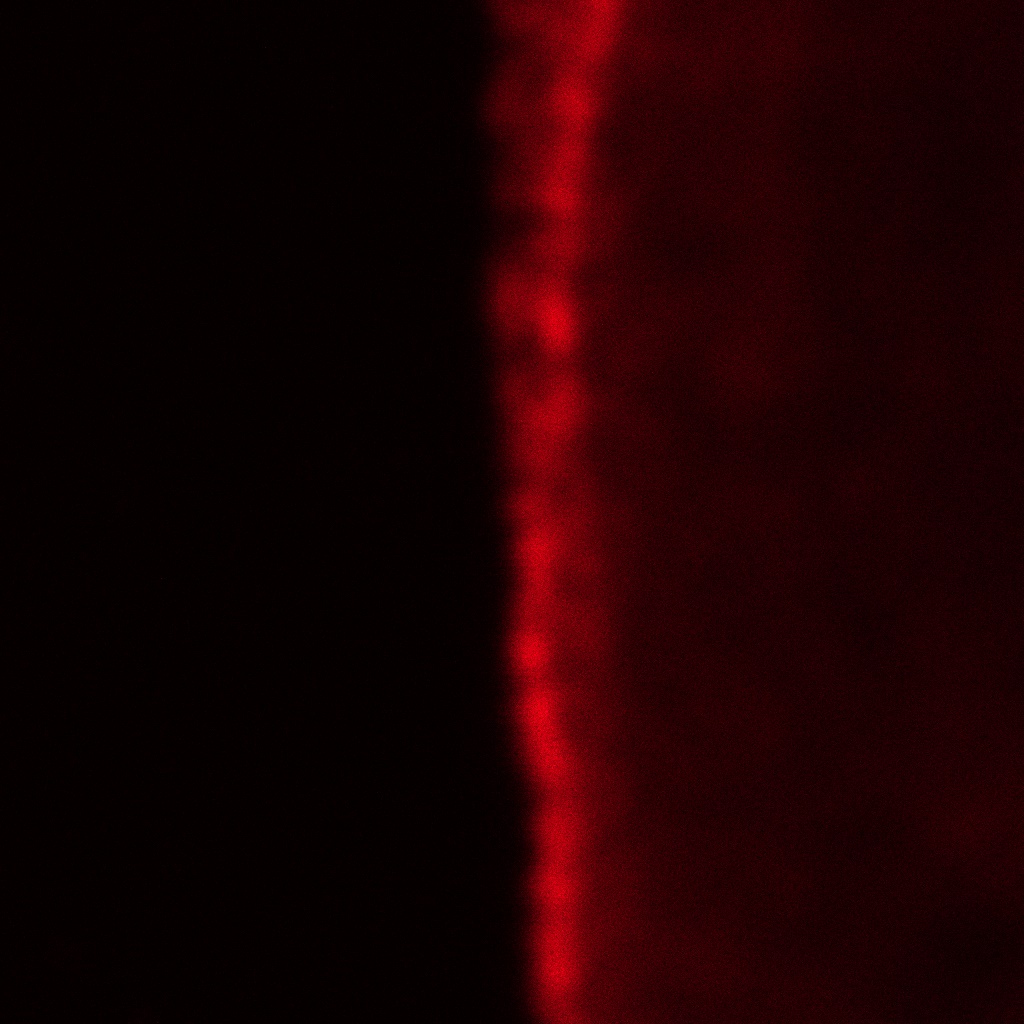

Supplement: Supplementary file 8 — Source Data for Figure 3 [file EMMM-13-e14887-s005.zip › EMM-2021-14887_SDataFig3/Fig. 3C Images/erk12flox-phalluidin-tpx1-40x-zoom-2-Image Export-33.jpg]

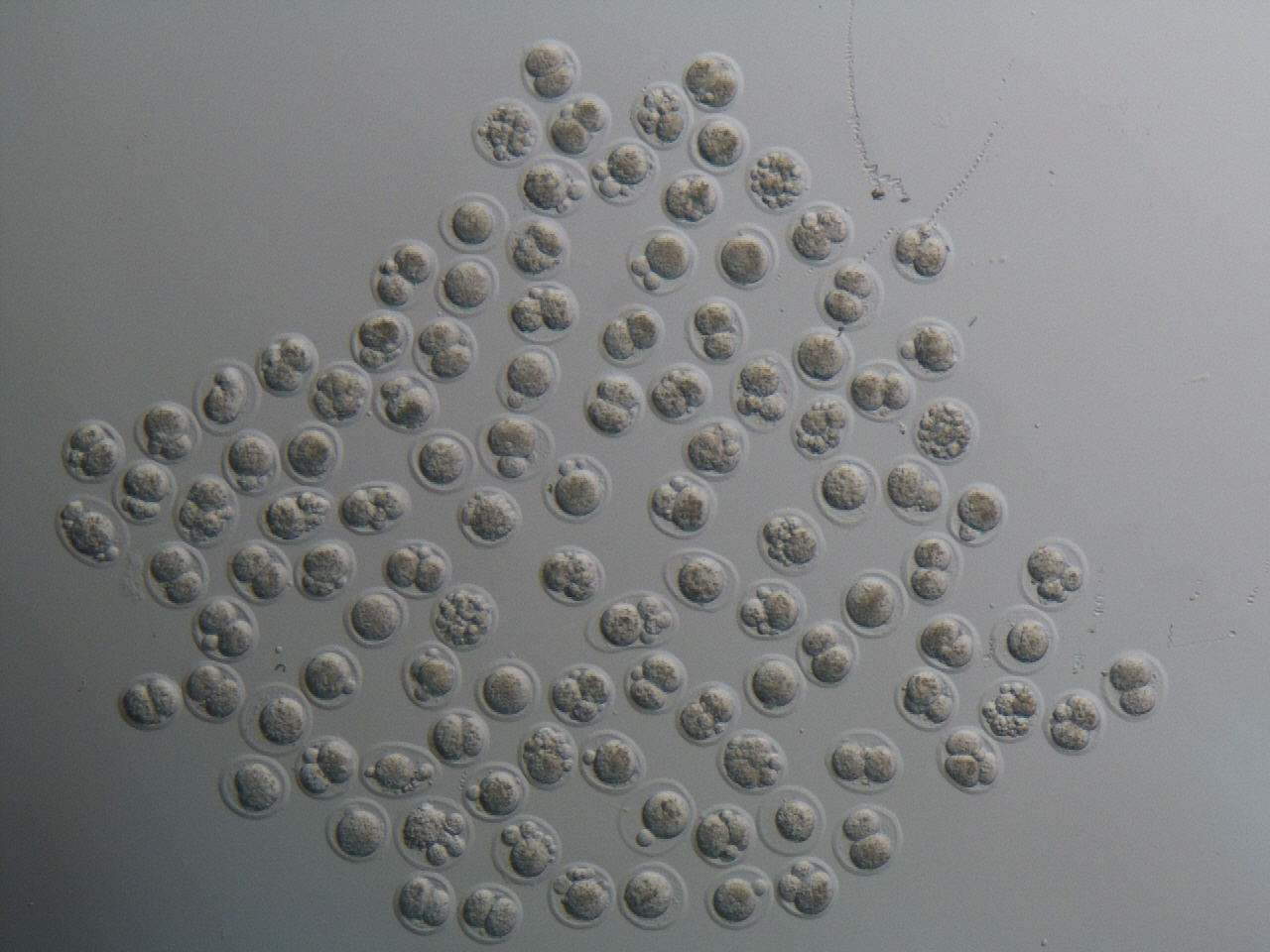

Supplement: Supplementary file 8 — Source Data for Figure 3 [file EMMM-13-e14887-s005.zip › EMM-2021-14887_SDataFig3/Fig. 3E Images/2-cell-ERK cKO-5.jpg]

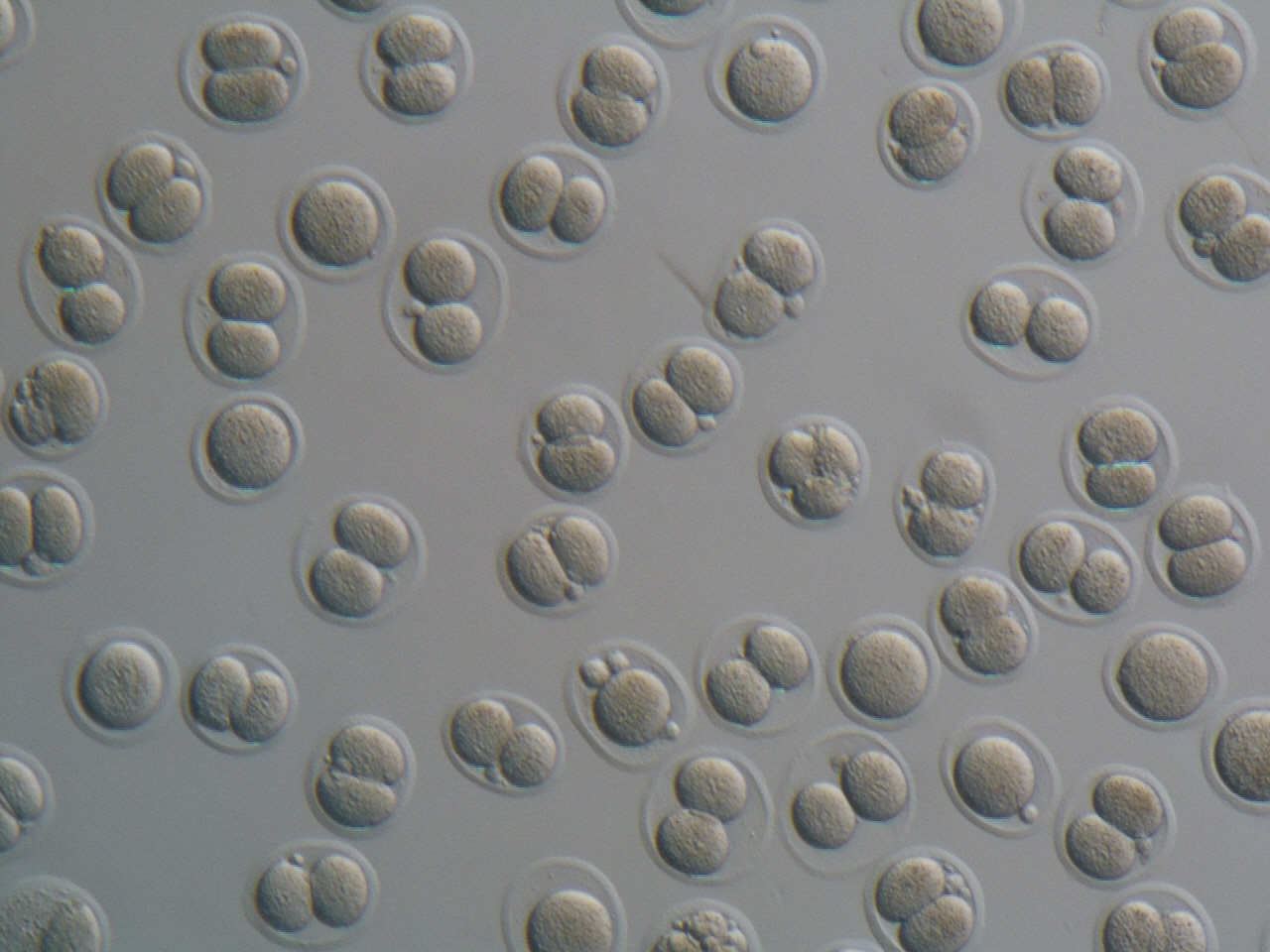

Supplement: Supplementary file 8 — Source Data for Figure 3 [file EMMM-13-e14887-s005.zip › EMM-2021-14887_SDataFig3/Fig. 3E Images/2cell-WT-3.jpg]

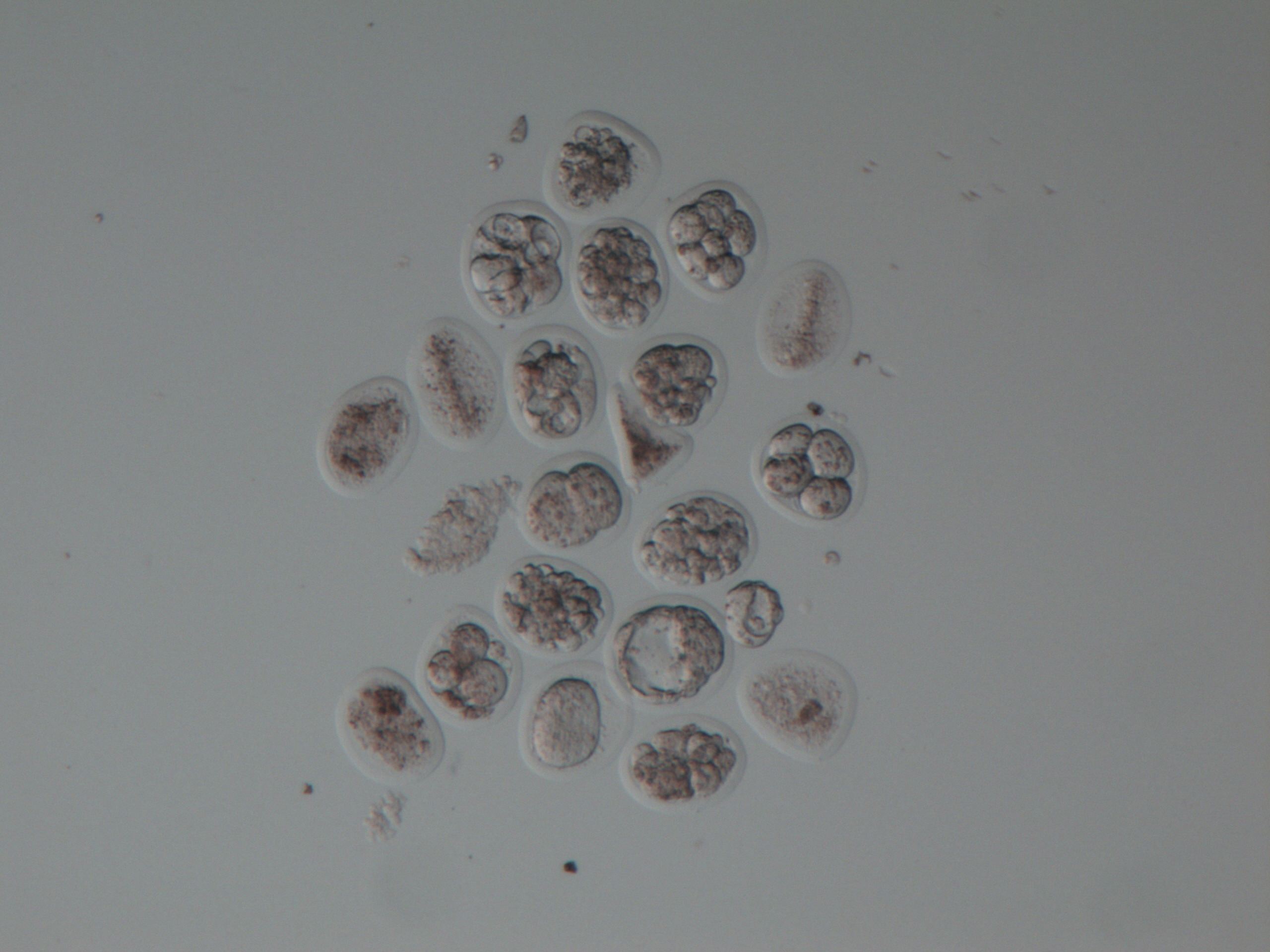

Supplement: Supplementary file 8 — Source Data for Figure 3 [file EMMM-13-e14887-s005.zip › EMM-2021-14887_SDataFig3/Fig. 3E Images/Blastomere_Erk cKO_2.jpg]

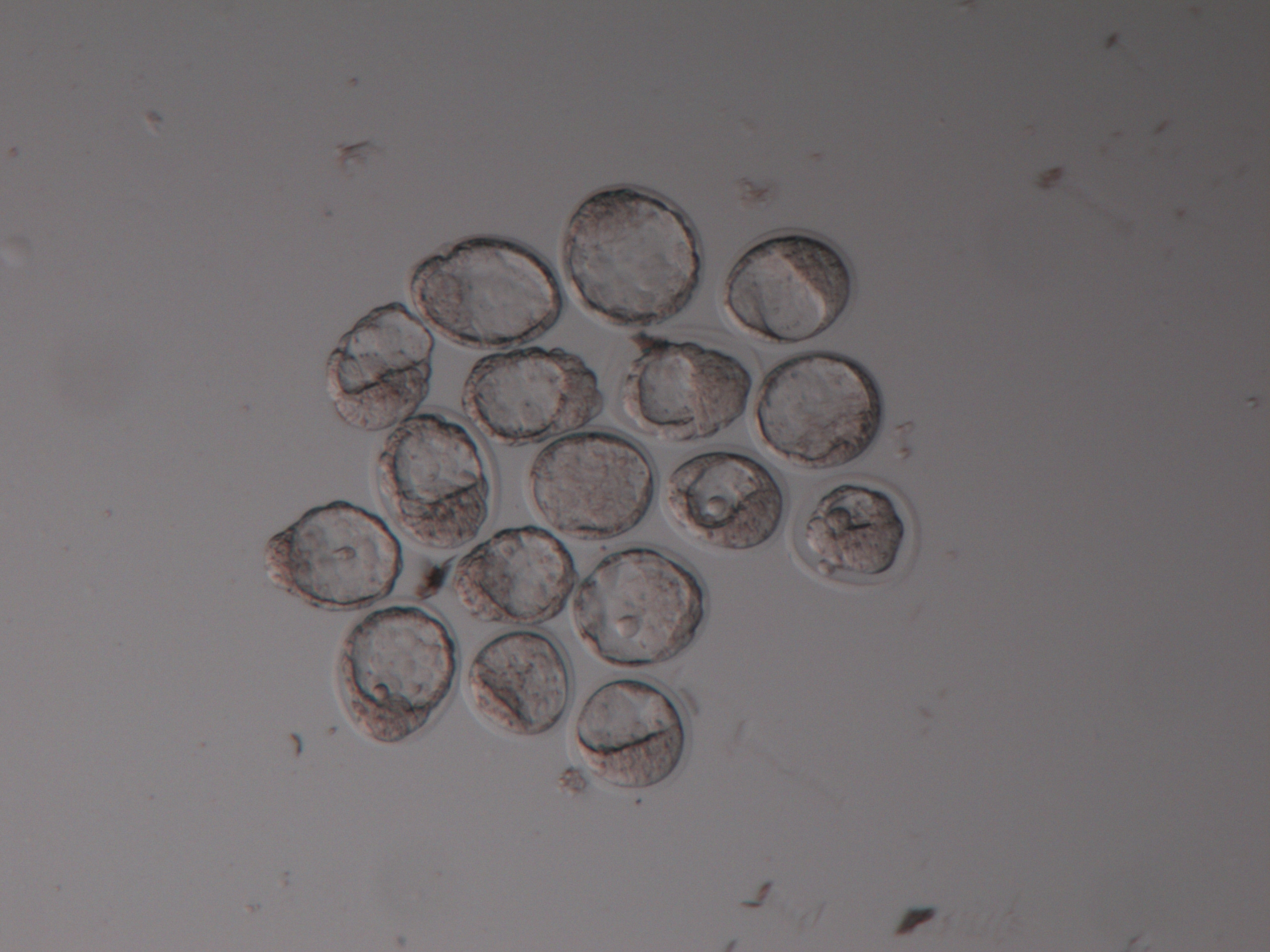

Supplement: Supplementary file 8 — Source Data for Figure 3 [file EMMM-13-e14887-s005.zip › EMM-2021-14887_SDataFig3/Fig. 3E Images/Blastomere_WT_1.jpg]

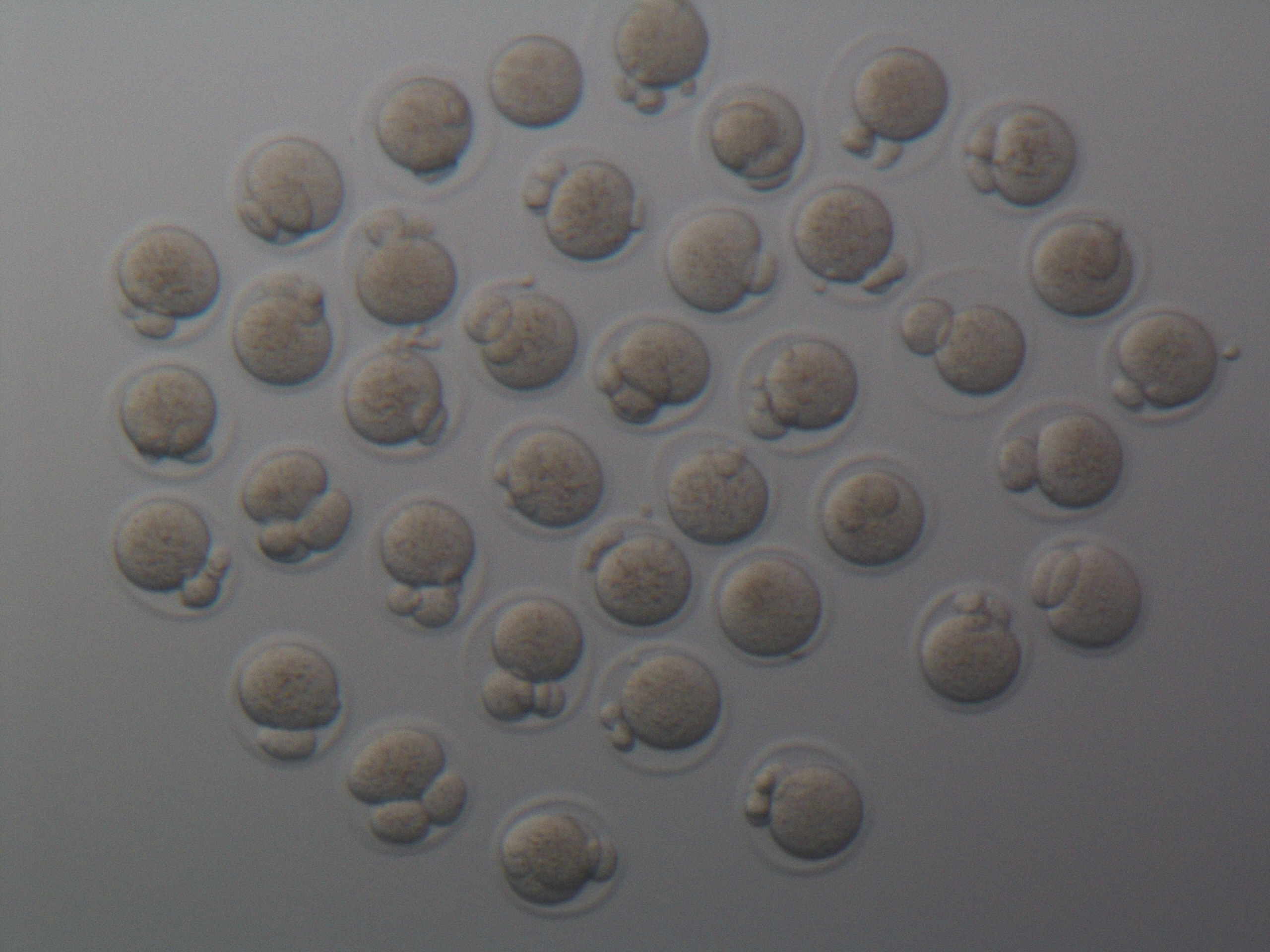

Supplement: Supplementary file 8 — Source Data for Figure 3 [file EMMM-13-e14887-s005.zip › EMM-2021-14887_SDataFig3/Fig. 3E Images/MII-ERK cKO-1.jpg]

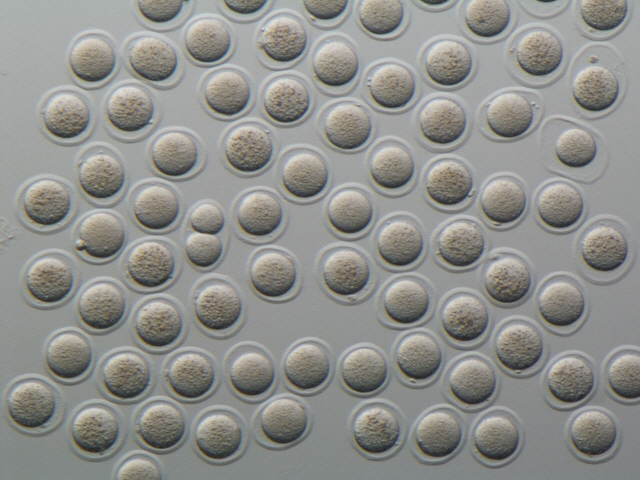

Supplement: Supplementary file 8 — Source Data for Figure 3 [file EMMM-13-e14887-s005.zip › EMM-2021-14887_SDataFig3/Fig. 3E Images/MII-WT.jpg]

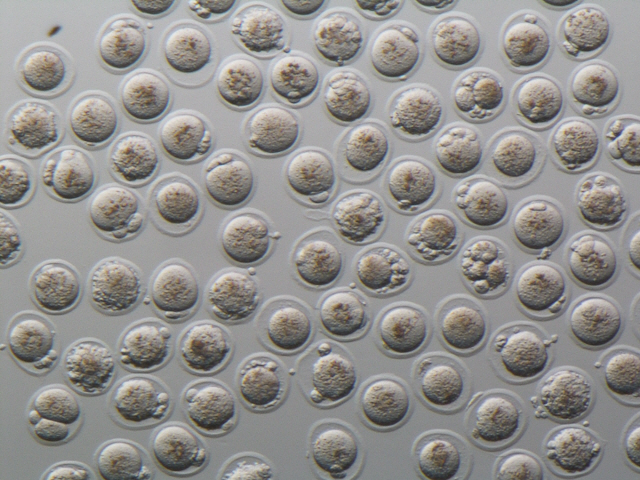

Supplement: Supplementary file 8 — Source Data for Figure 3 [file EMMM-13-e14887-s005.zip › EMM-2021-14887_SDataFig3/Fig. 3E Images/Zygote-Erk Cko-2.jpg]

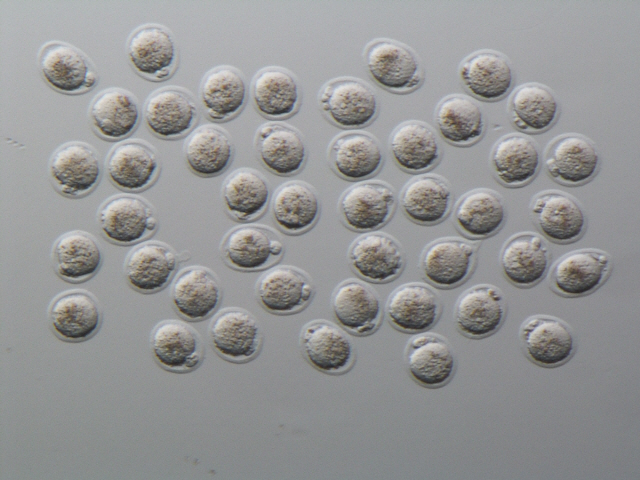

Supplement: Supplementary file 8 — Source Data for Figure 3 [file EMMM-13-e14887-s005.zip › EMM-2021-14887_SDataFig3/Fig. 3E Images/Zygote-WT-PN-2.jpg]

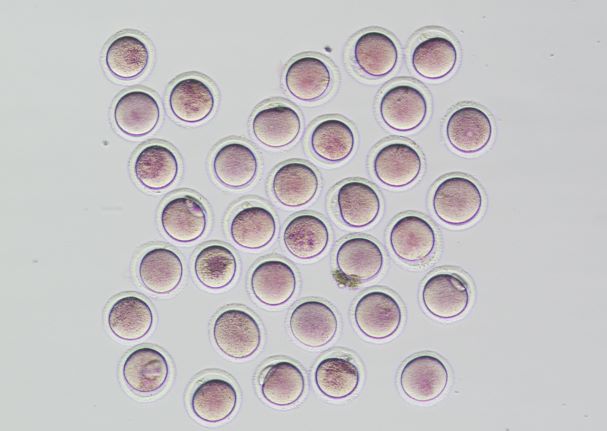

Supplement: Supplementary file 11 — Source Data for Figure 6 [file EMMM-13-e14887-s012.zip › EMM-2021-14887_SDataFig6/Fig. 6C/NT-B-10X-1.jpg]

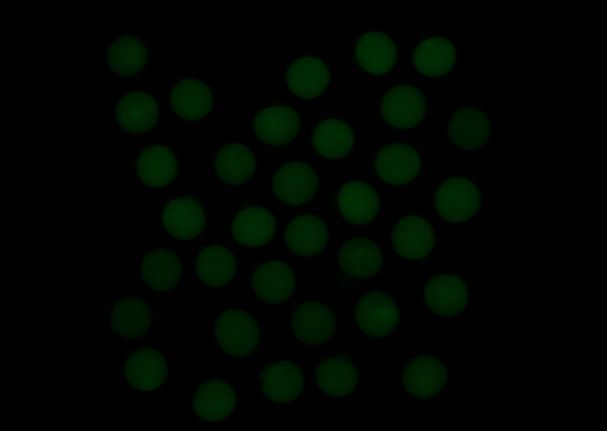

Supplement: Supplementary file 11 — Source Data for Figure 6 [file EMMM-13-e14887-s012.zip › EMM-2021-14887_SDataFig6/Fig. 6C/NT-JC-1-GREEN-10X-1.jpg]

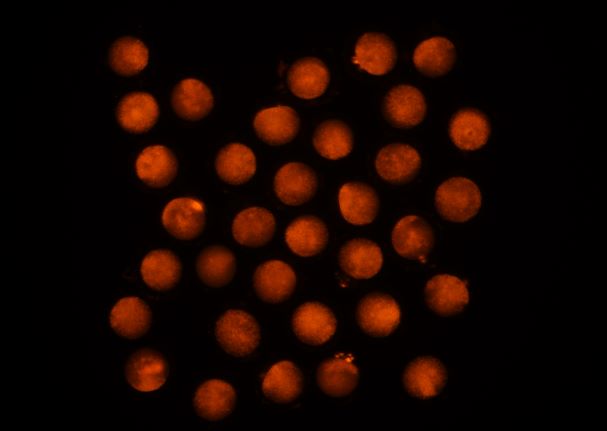

Supplement: Supplementary file 11 — Source Data for Figure 6 [file EMMM-13-e14887-s012.zip › EMM-2021-14887_SDataFig6/Fig. 6C/NT-JC-1-RED-10X-1.jpg]

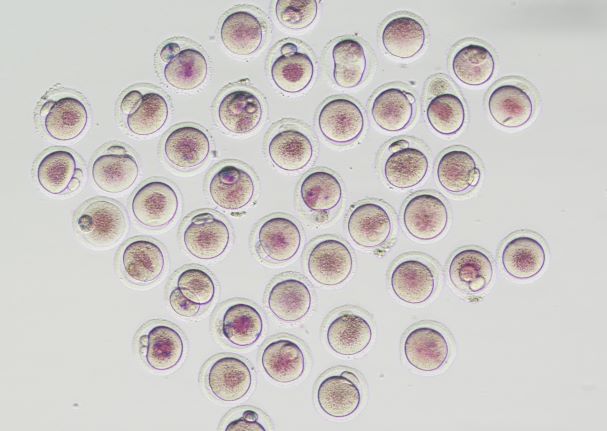

Supplement: Supplementary file 11 — Source Data for Figure 6 [file EMMM-13-e14887-s012.zip › EMM-2021-14887_SDataFig6/Fig. 6C/U0126-B-10X-1.jpg]
