# Supplementary figures and images for: Biallelic mutations in MOS cause female infertility characterized by human early embryonic arrest and fragmentation (part 2 of 2)
Source: EMBO Mol Med. 2021 Nov 15;13(12):e14887. doi: 10.15252/emmm.202114887 (PMC8649871; doi:10.15252/emmm.202114887)

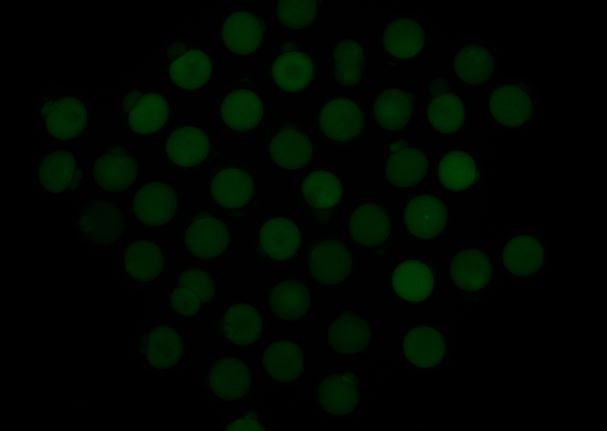

Supplement: Supplementary file 11 — Source Data for Figure 6 [file EMMM-13-e14887-s012.zip › EMM-2021-14887_SDataFig6/Fig. 6C/U0126-JC-1-GREEN-10X-1.jpg]

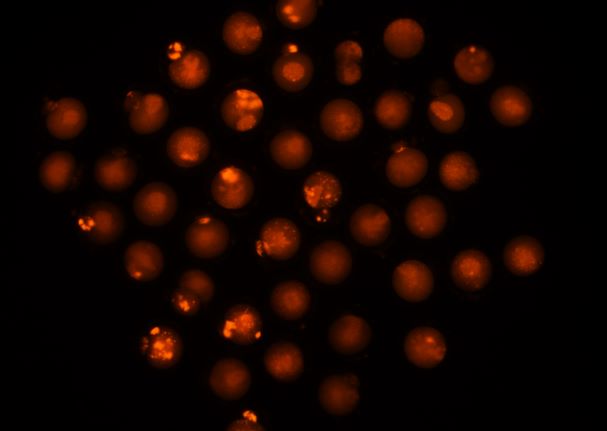

Supplement: Supplementary file 11 — Source Data for Figure 6 [file EMMM-13-e14887-s012.zip › EMM-2021-14887_SDataFig6/Fig. 6C/U0126-JC-1-RED-10X-1.jpg]

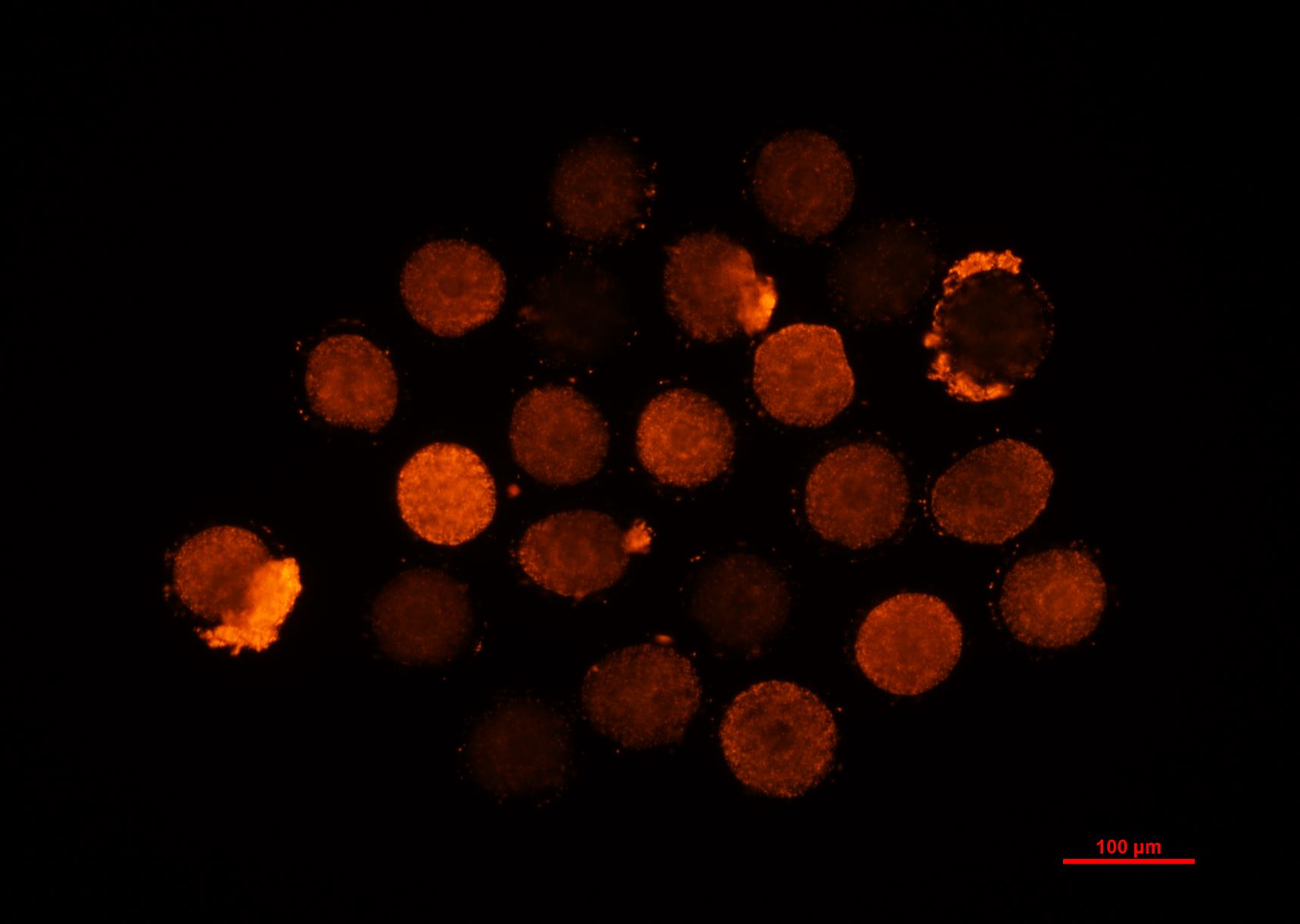

Supplement: Supplementary file 11 — Source Data for Figure 6 [file EMMM-13-e14887-s012.zip › EMM-2021-14887_SDataFig6/Fig. 6E/Erk cko-GV-jc-1-Aggregates-10X-1.jpg]

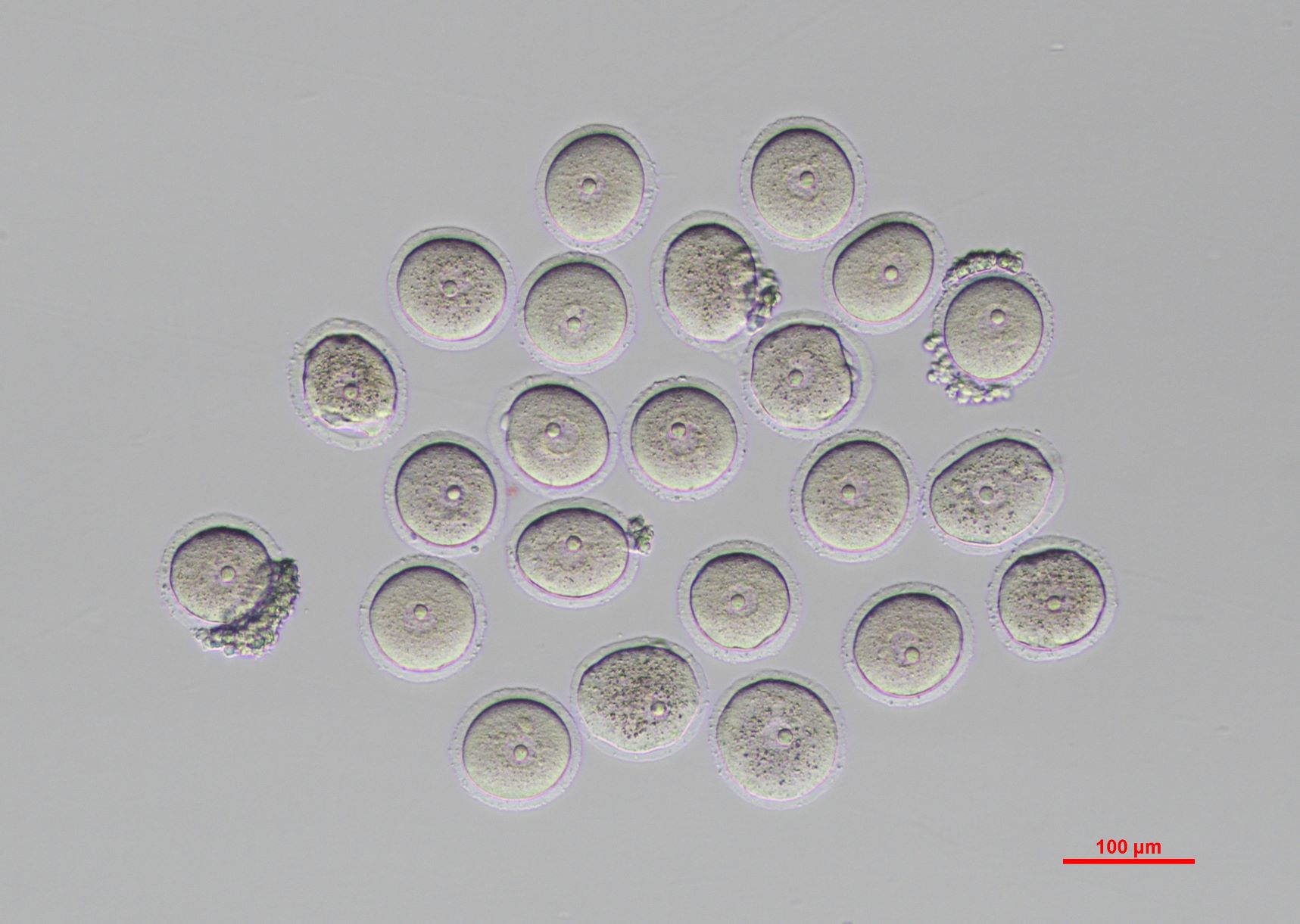

Supplement: Supplementary file 11 — Source Data for Figure 6 [file EMMM-13-e14887-s012.zip › EMM-2021-14887_SDataFig6/Fig. 6E/Erk cko-GV-jc-1-Bright-10X-1.jpg]

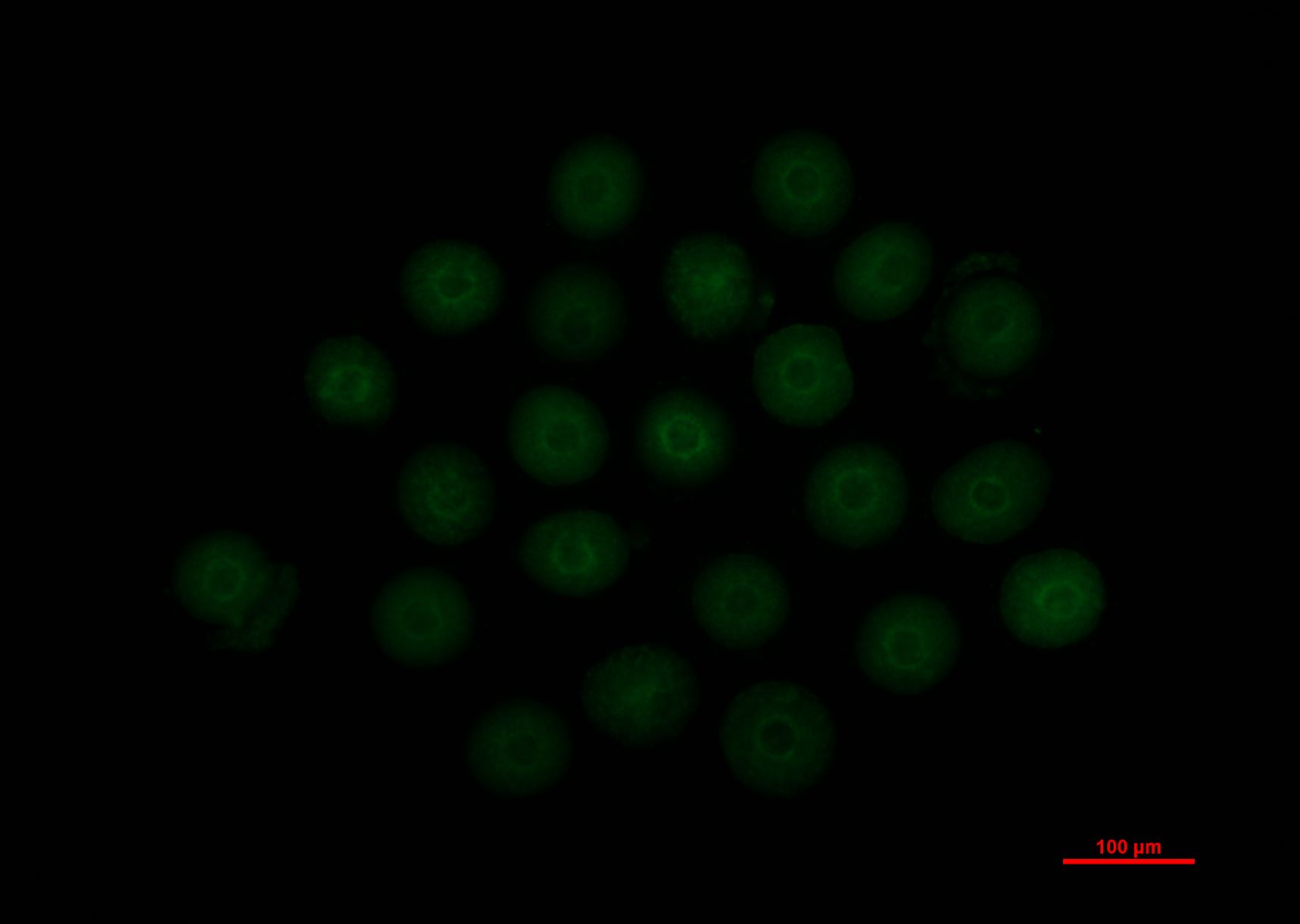

Supplement: Supplementary file 11 — Source Data for Figure 6 [file EMMM-13-e14887-s012.zip › EMM-2021-14887_SDataFig6/Fig. 6E/Erk cko-GV-jc-1-Monomers-10X-1.jpg]

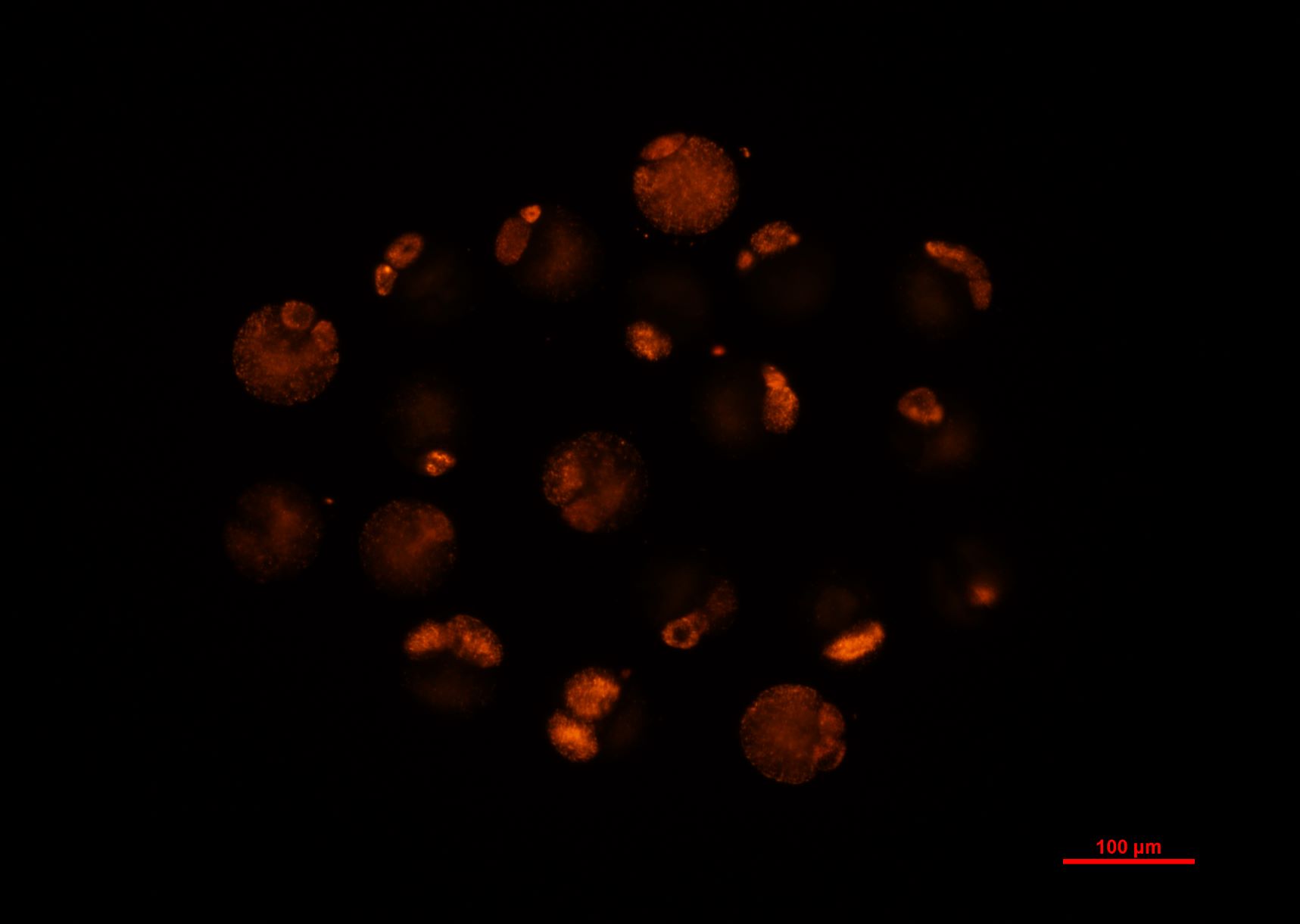

Supplement: Supplementary file 11 — Source Data for Figure 6 [file EMMM-13-e14887-s012.zip › EMM-2021-14887_SDataFig6/Fig. 6E/Erk cko-MII-jc-1-Aggregates-10X-1.jpg]

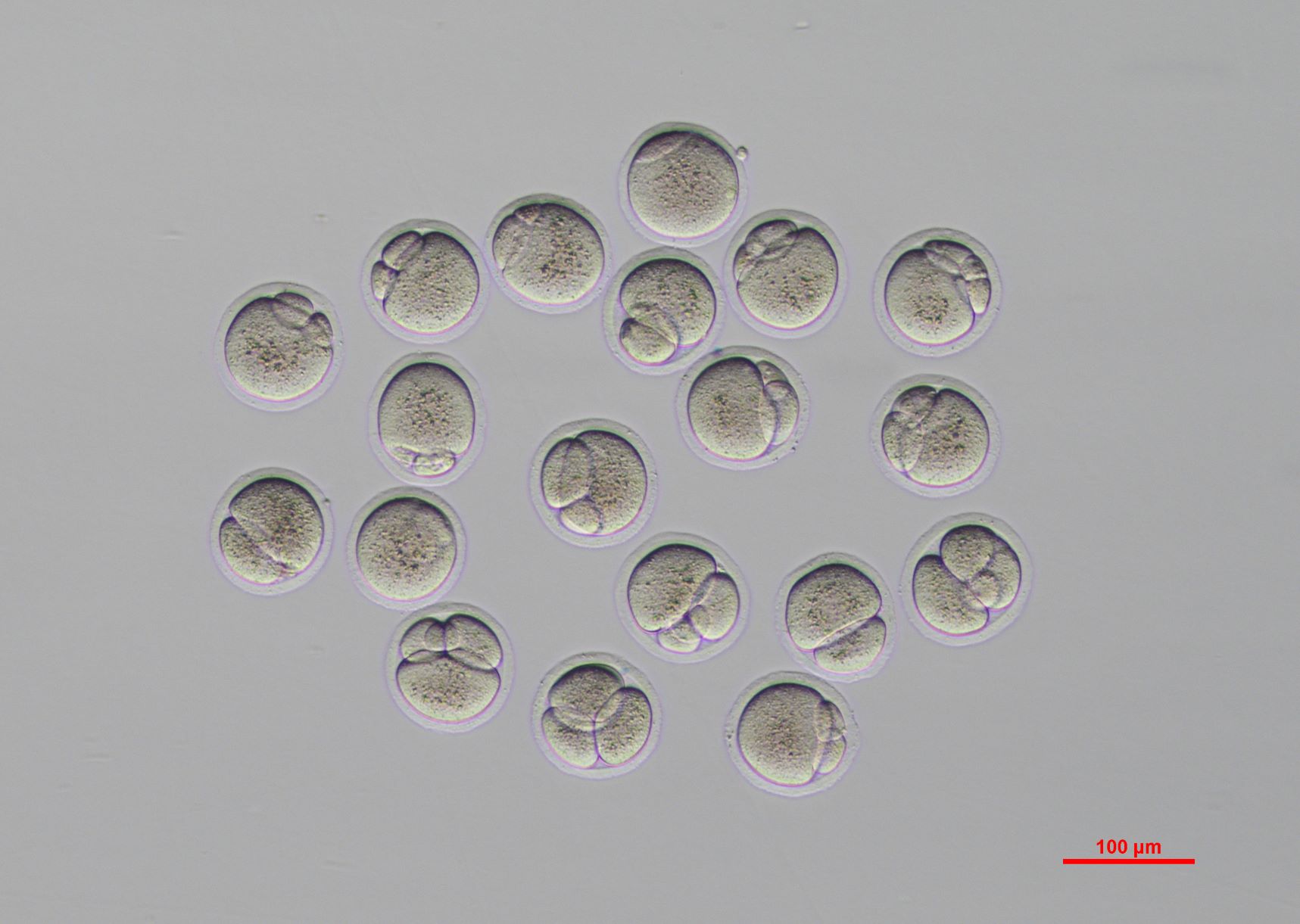

Supplement: Supplementary file 11 — Source Data for Figure 6 [file EMMM-13-e14887-s012.zip › EMM-2021-14887_SDataFig6/Fig. 6E/Erk cko-MII-jc-1-Bright-10X-2.jpg]

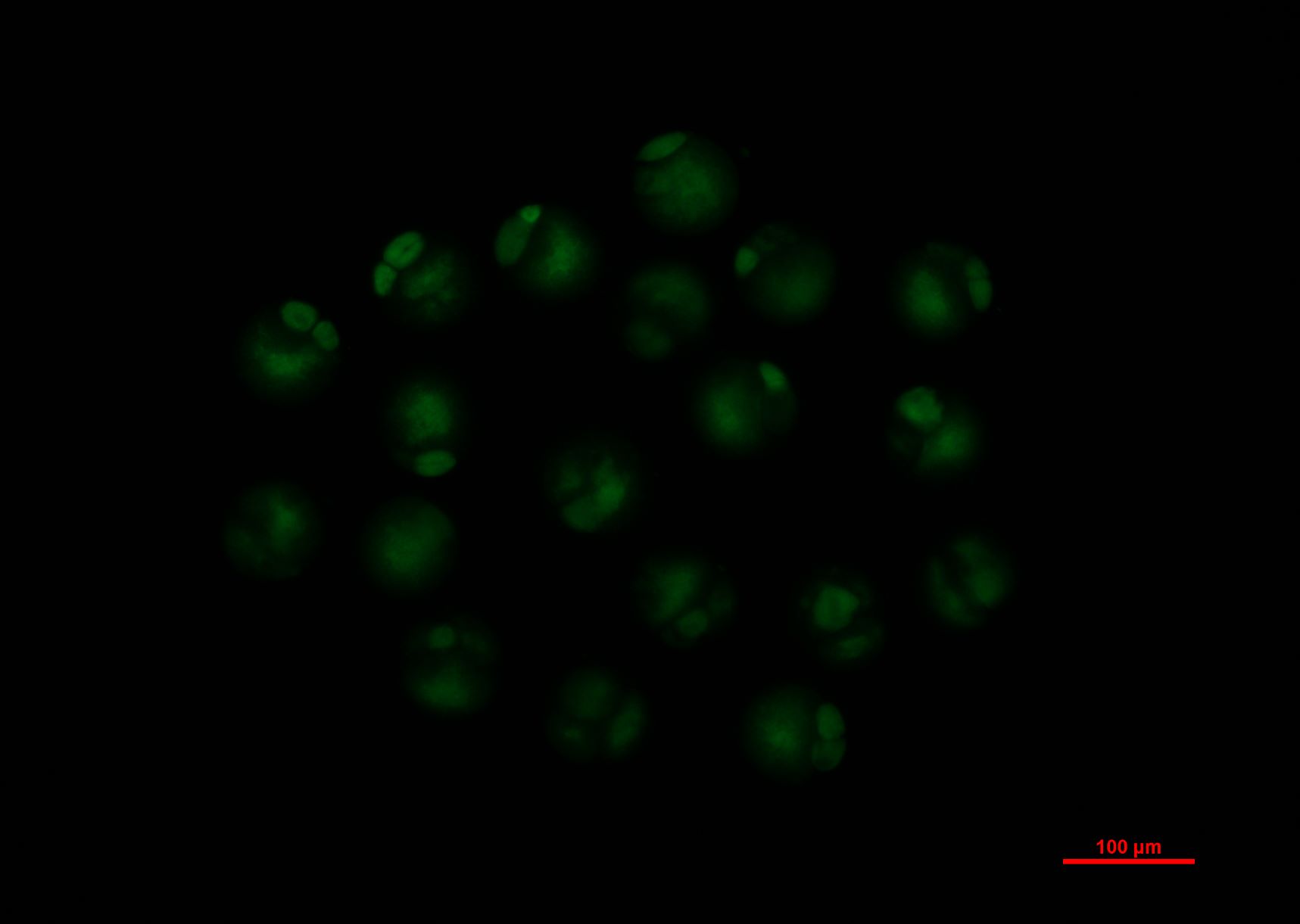

Supplement: Supplementary file 11 — Source Data for Figure 6 [file EMMM-13-e14887-s012.zip › EMM-2021-14887_SDataFig6/Fig. 6E/Erk cko-MII-jc-1-Monomers-10X-1.jpg]

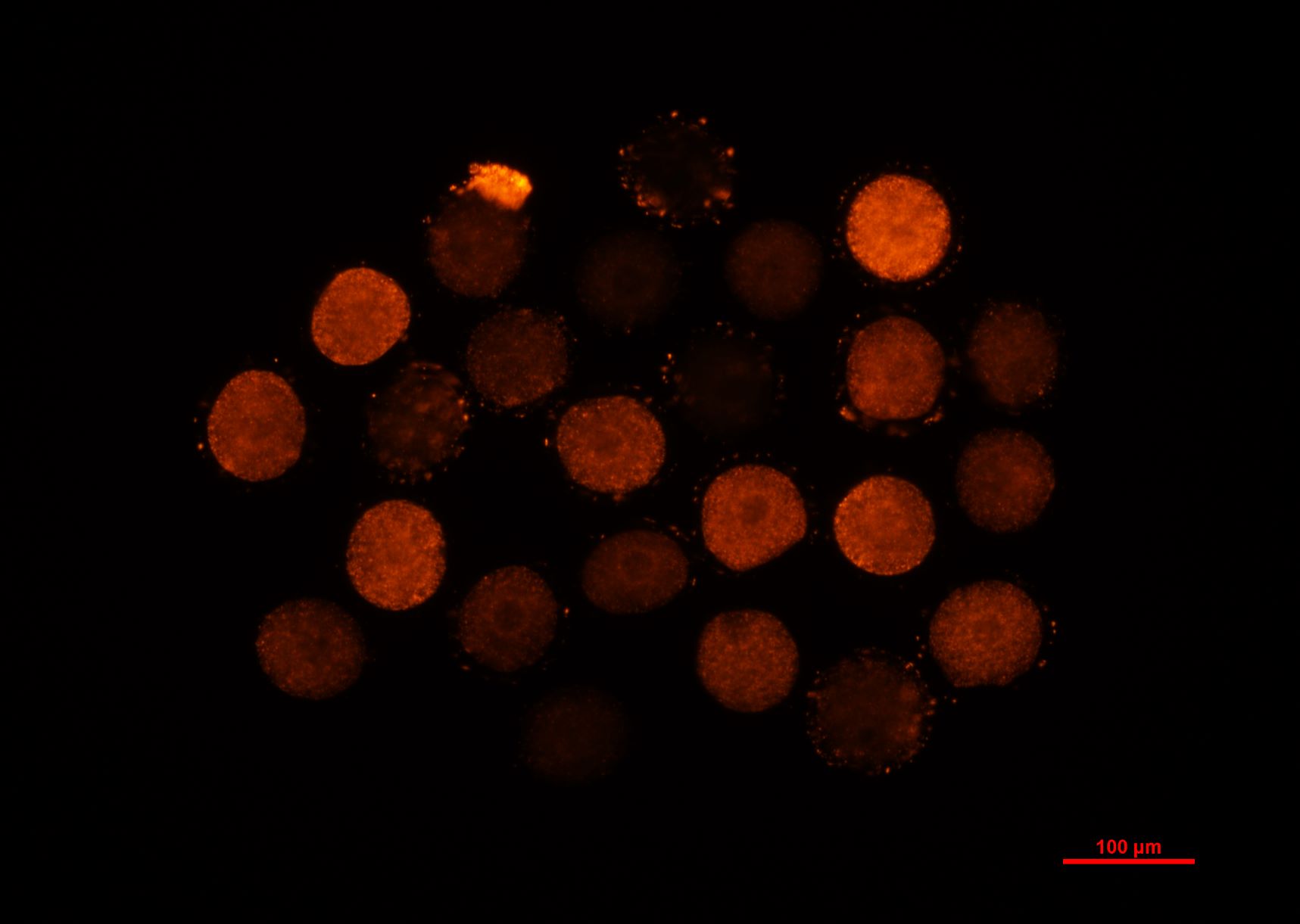

Supplement: Supplementary file 11 — Source Data for Figure 6 [file EMMM-13-e14887-s012.zip › EMM-2021-14887_SDataFig6/Fig. 6E/WT-GV-jc-1-Aggregates-10X-1.jpg]

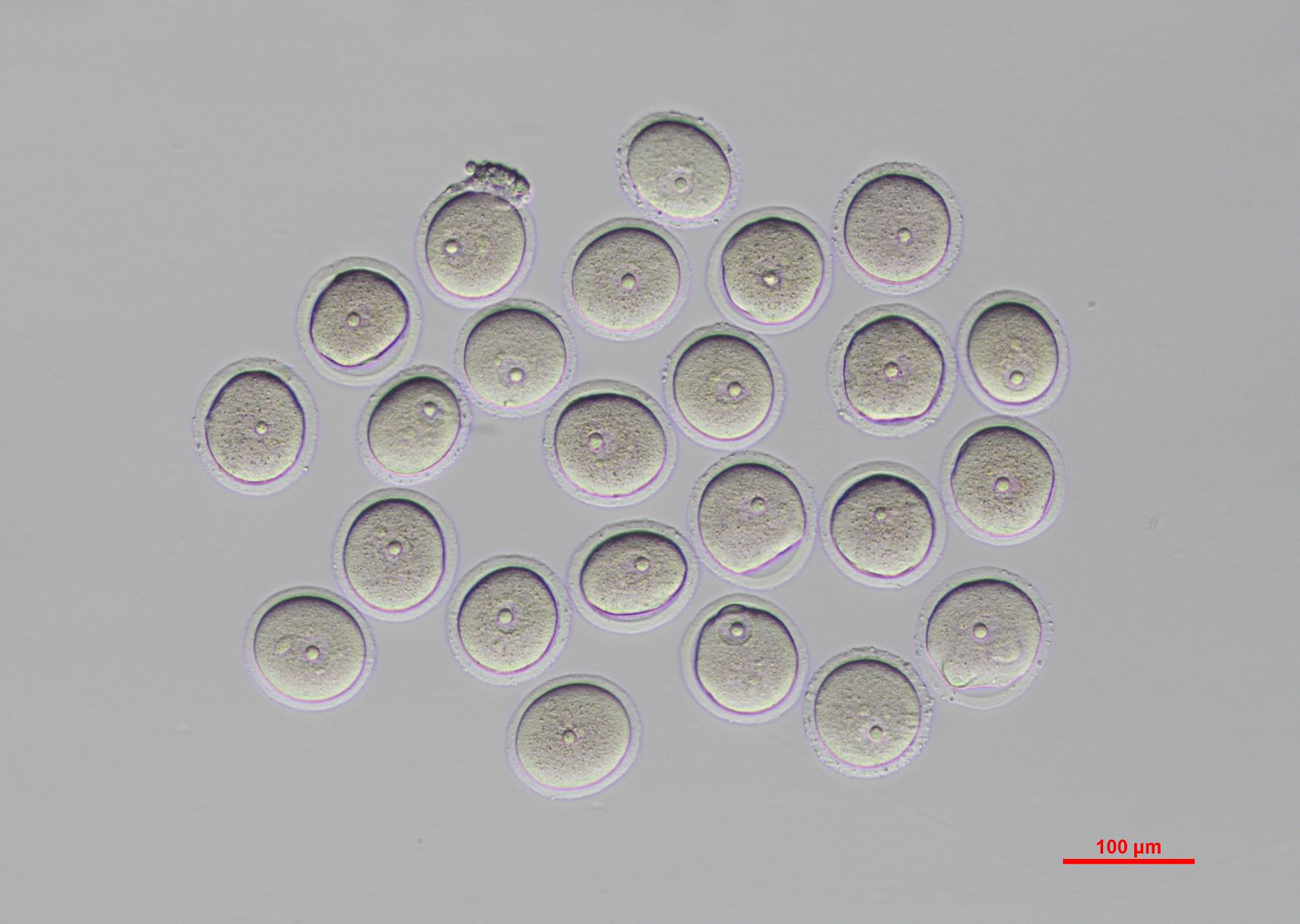

Supplement: Supplementary file 11 — Source Data for Figure 6 [file EMMM-13-e14887-s012.zip › EMM-2021-14887_SDataFig6/Fig. 6E/WT-GV-jc-1-Bright-10X-1.jpg]

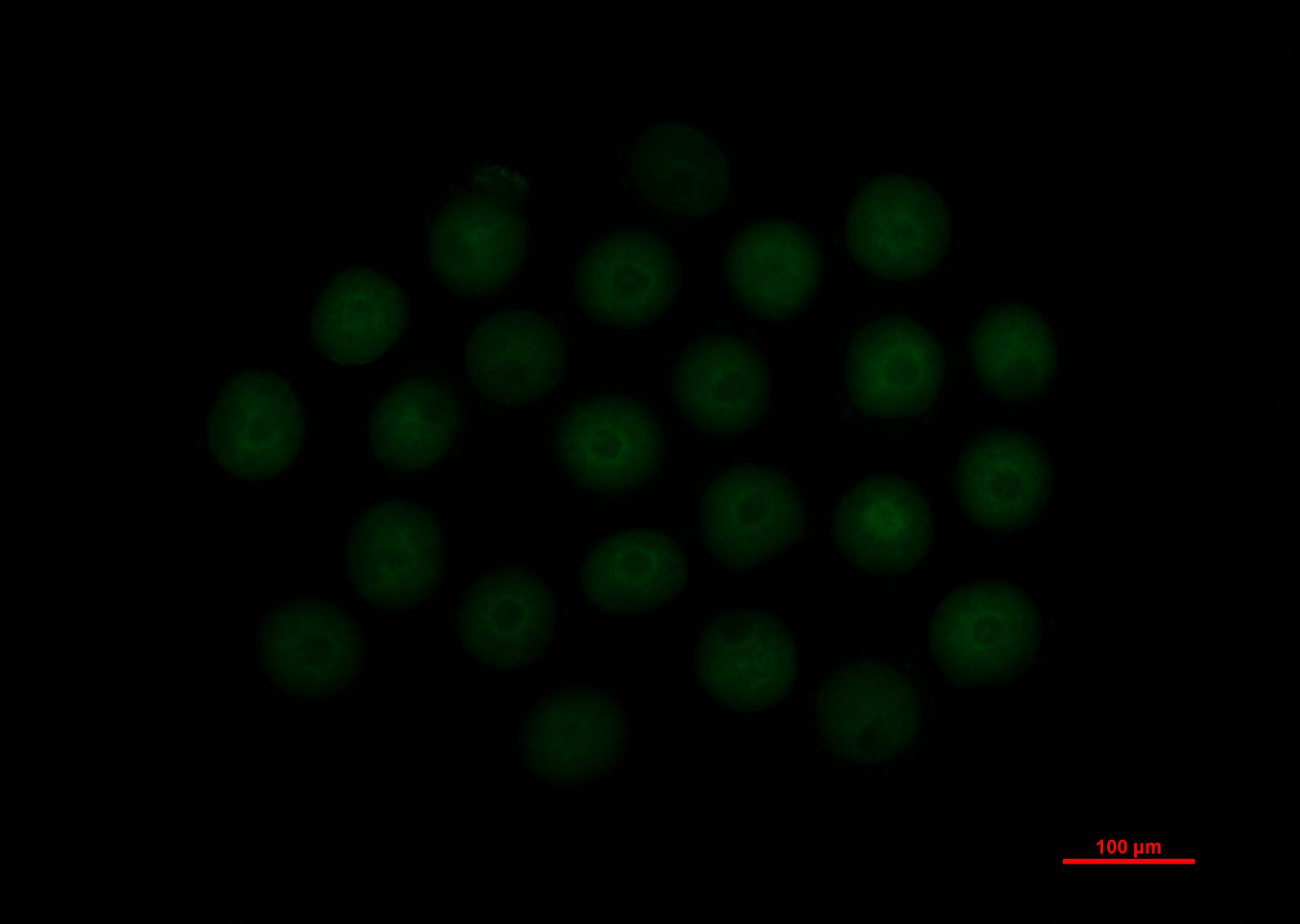

Supplement: Supplementary file 11 — Source Data for Figure 6 [file EMMM-13-e14887-s012.zip › EMM-2021-14887_SDataFig6/Fig. 6E/WT-GV-jc-1-Monomers-10X-1.jpg]

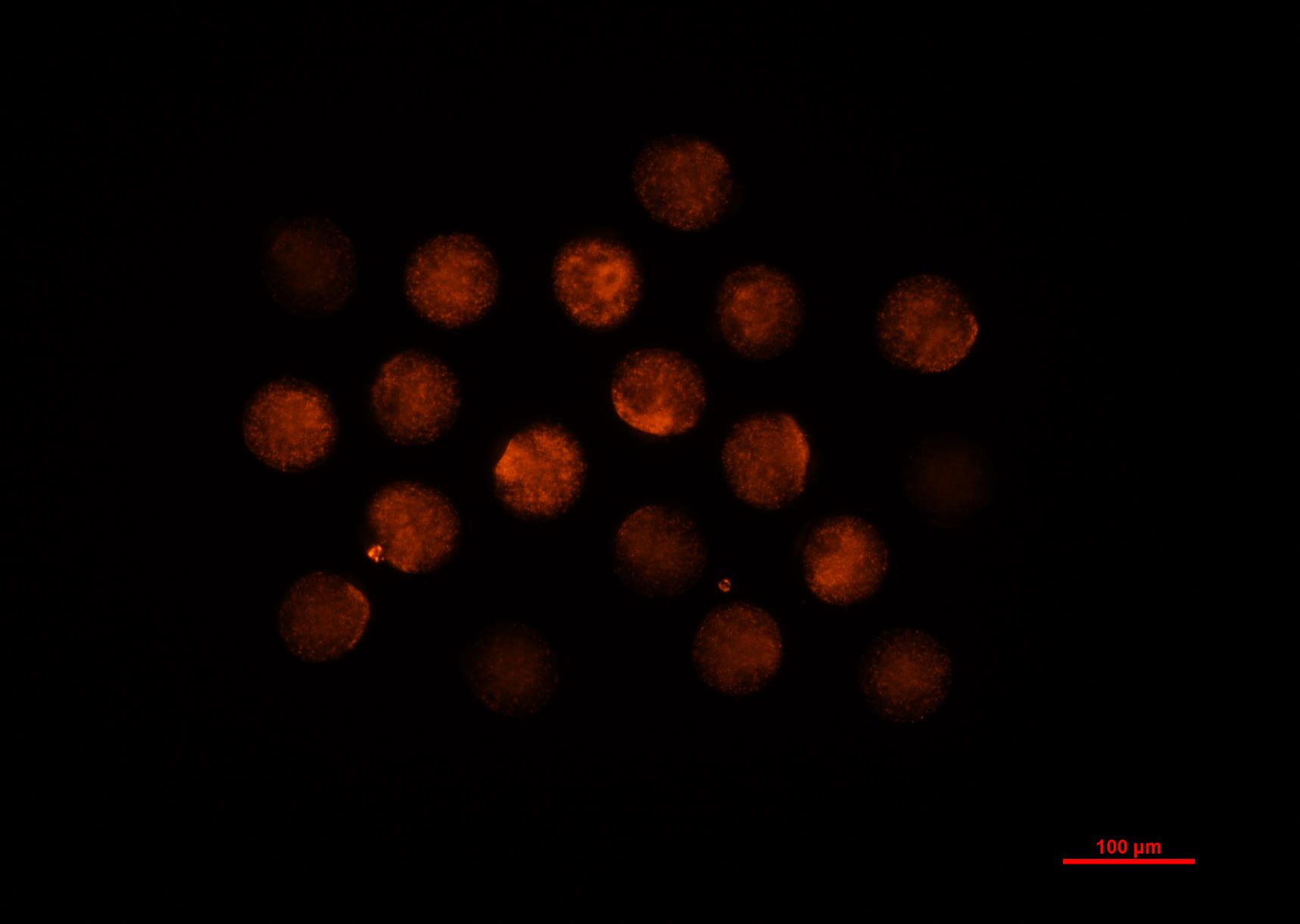

Supplement: Supplementary file 11 — Source Data for Figure 6 [file EMMM-13-e14887-s012.zip › EMM-2021-14887_SDataFig6/Fig. 6E/WT-MII-jc-1-Aggregates-10X-1.jpg]

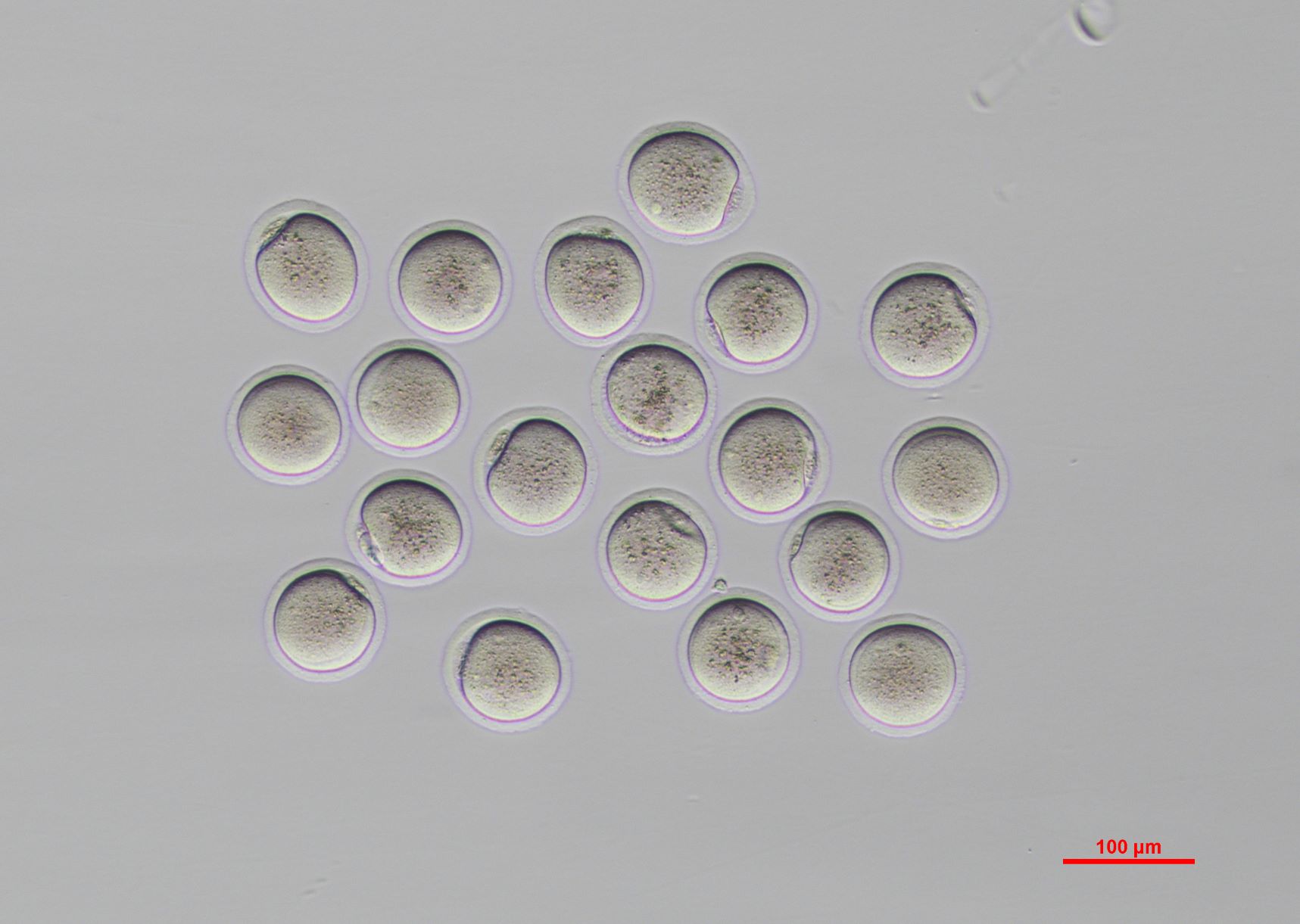

Supplement: Supplementary file 11 — Source Data for Figure 6 [file EMMM-13-e14887-s012.zip › EMM-2021-14887_SDataFig6/Fig. 6E/WT-MII-jc-1-Bright-10X-1.jpg]

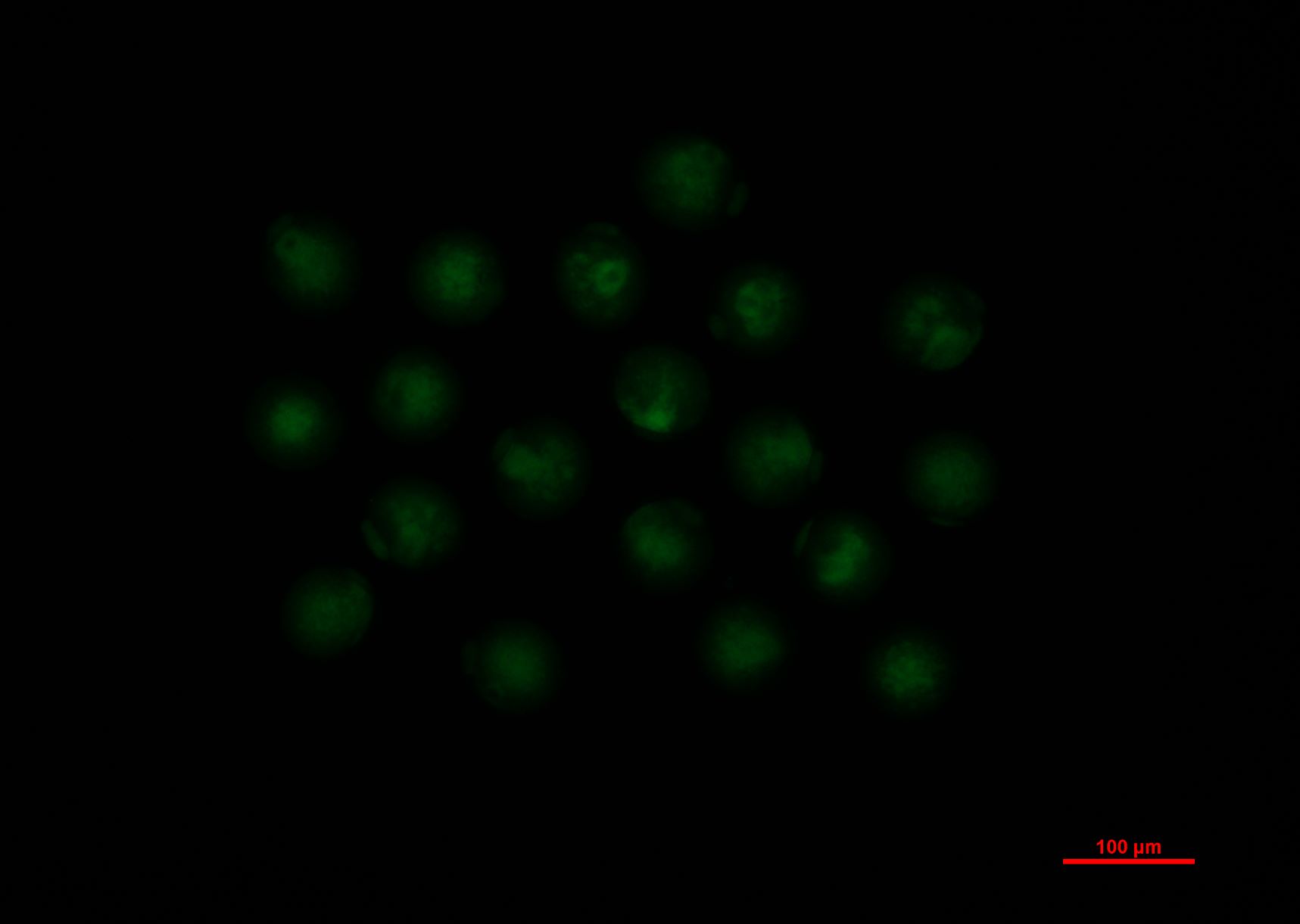

Supplement: Supplementary file 11 — Source Data for Figure 6 [file EMMM-13-e14887-s012.zip › EMM-2021-14887_SDataFig6/Fig. 6E/WT-MII-jc-1-Monomers-10X-1.jpg]

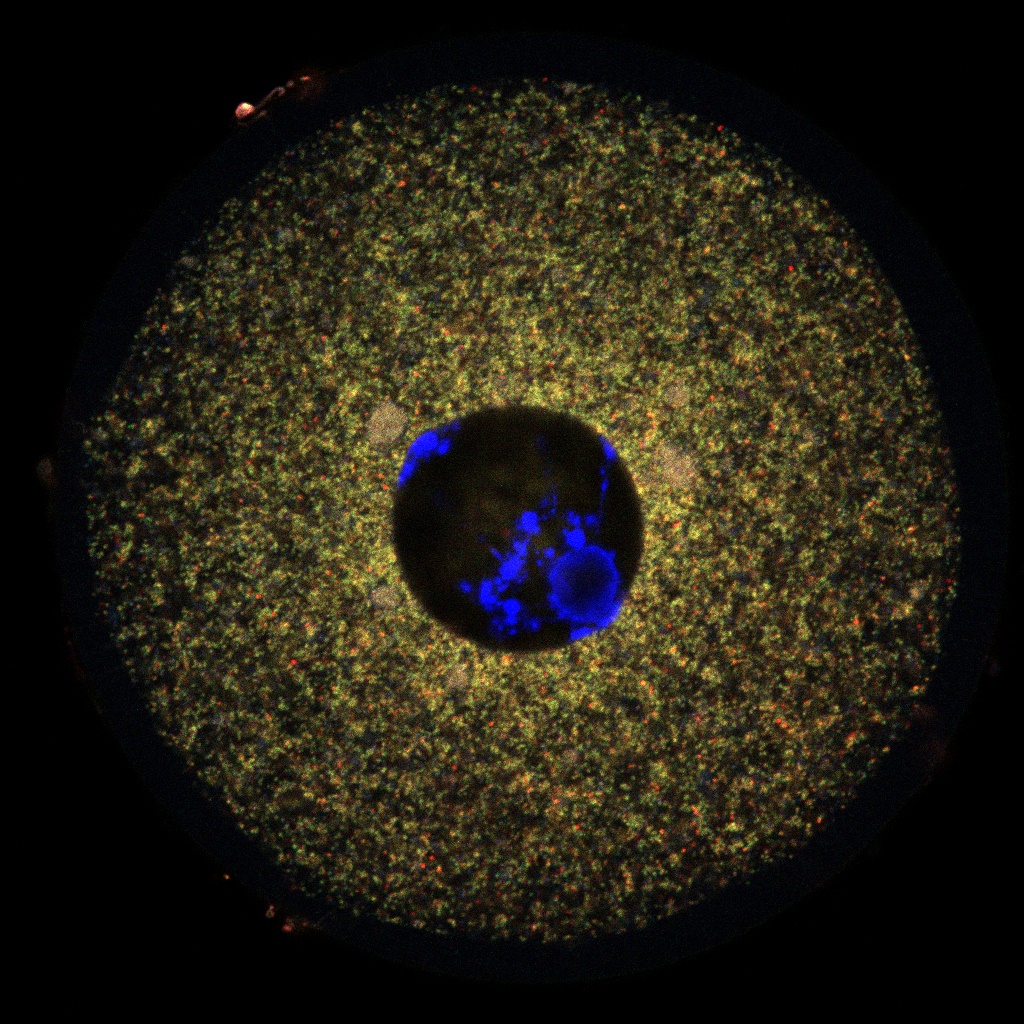

Supplement: Supplementary file 11 — Source Data for Figure 6 [file EMMM-13-e14887-s012.zip › EMM-2021-14887_SDataFig6/Fig. 6F/Erk cko-GV-jc-1-40x-2-Image Export-03/erkcko-GV-jc-1-40x-2-Image Export-03_c1-3.jpg]

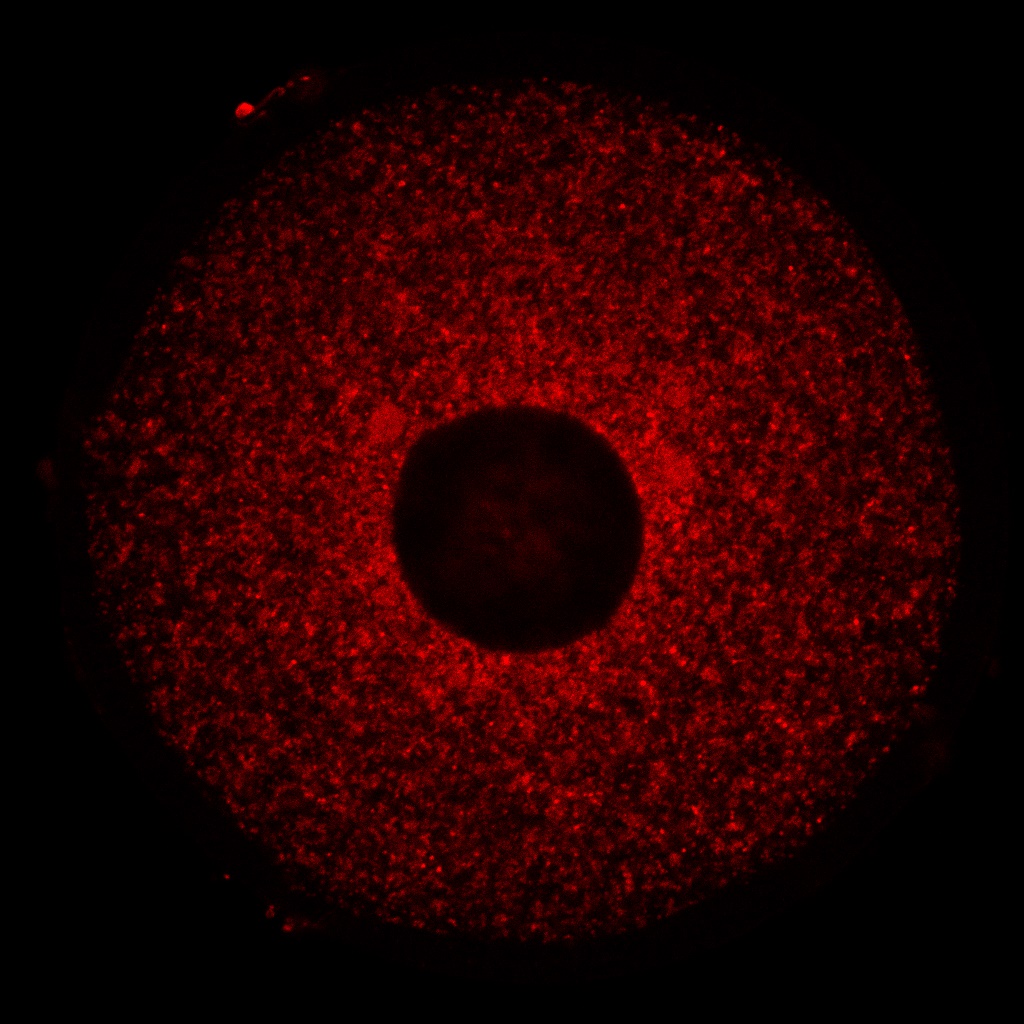

Supplement: Supplementary file 11 — Source Data for Figure 6 [file EMMM-13-e14887-s012.zip › EMM-2021-14887_SDataFig6/Fig. 6F/Erk cko-GV-jc-1-40x-2-Image Export-03/erkcko-GV-jc-1-40x-2-Image Export-03_c1.jpg]

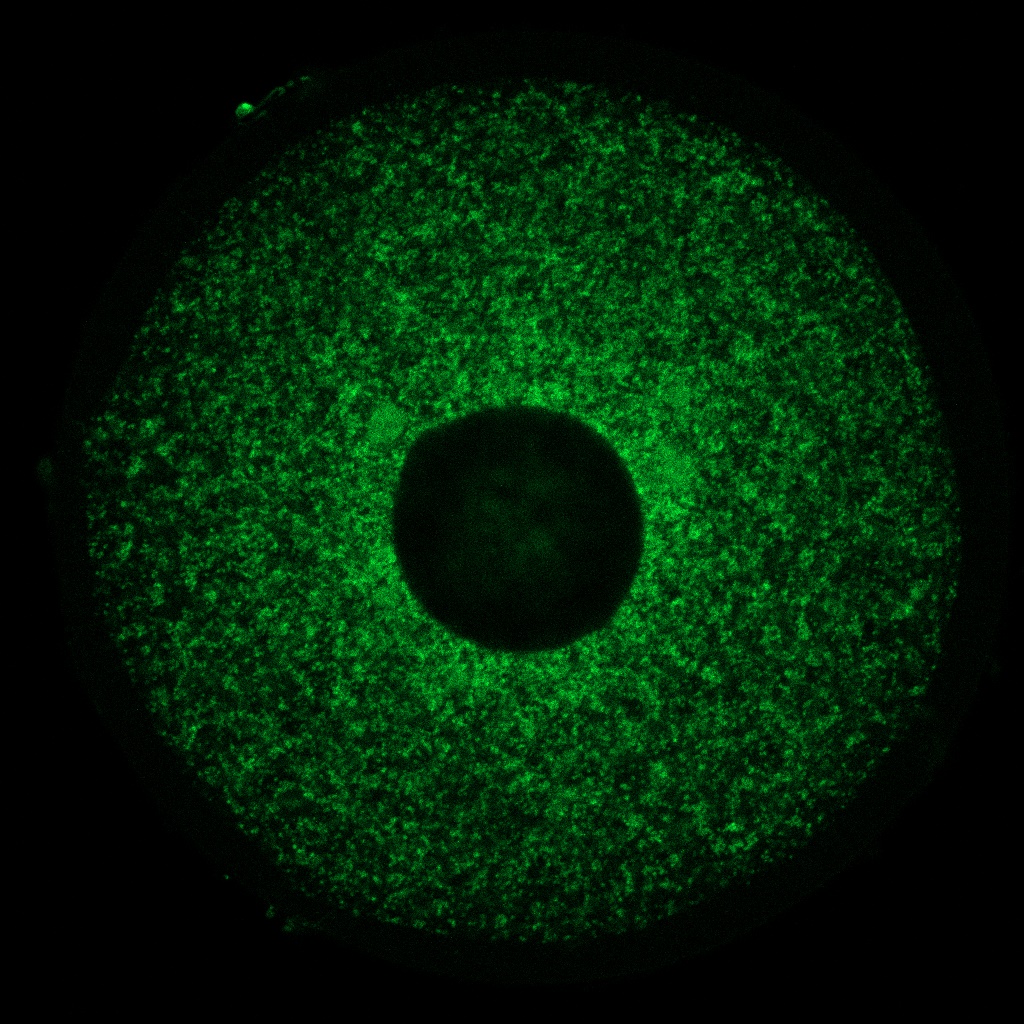

Supplement: Supplementary file 11 — Source Data for Figure 6 [file EMMM-13-e14887-s012.zip › EMM-2021-14887_SDataFig6/Fig. 6F/Erk cko-GV-jc-1-40x-2-Image Export-03/erkcko-GV-jc-1-40x-2-Image Export-03_c2.jpg]

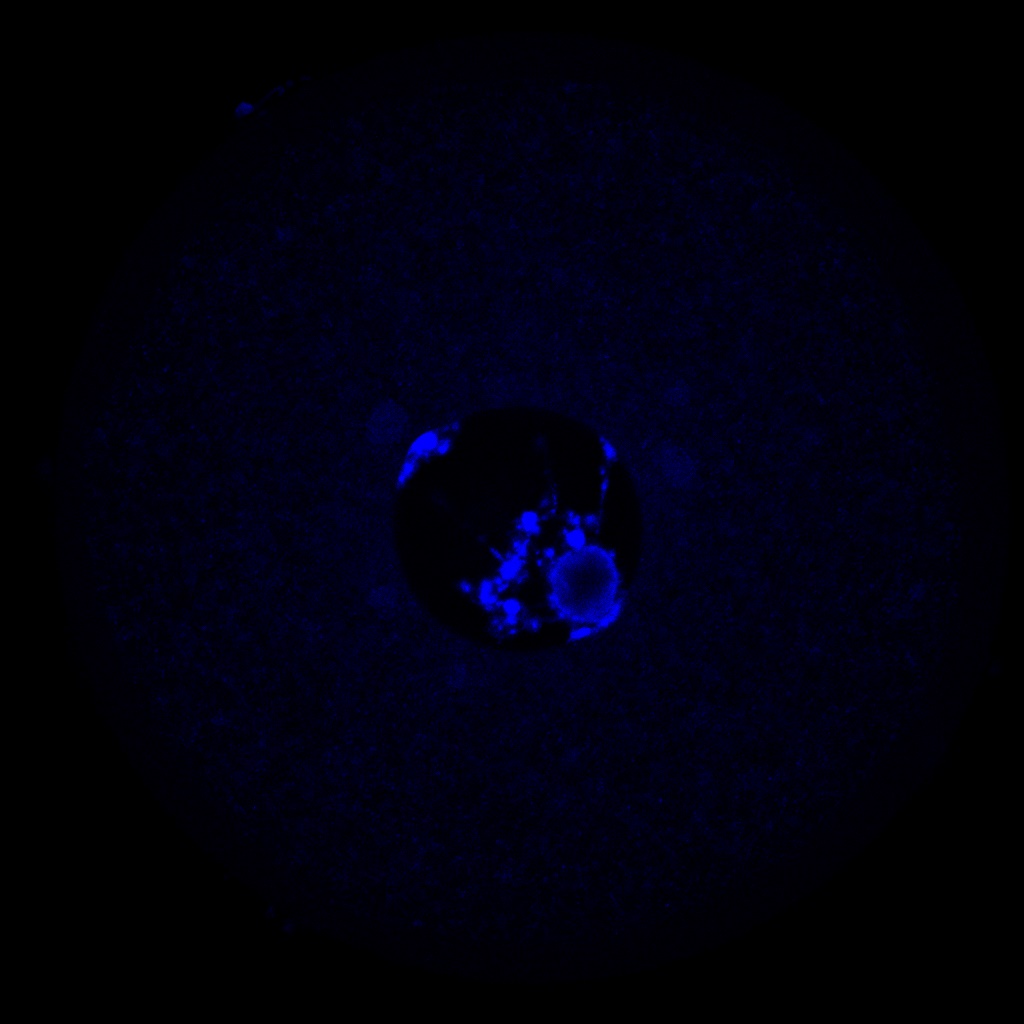

Supplement: Supplementary file 11 — Source Data for Figure 6 [file EMMM-13-e14887-s012.zip › EMM-2021-14887_SDataFig6/Fig. 6F/Erk cko-GV-jc-1-40x-2-Image Export-03/erkcko-GV-jc-1-40x-2-Image Export-03_c3.jpg]

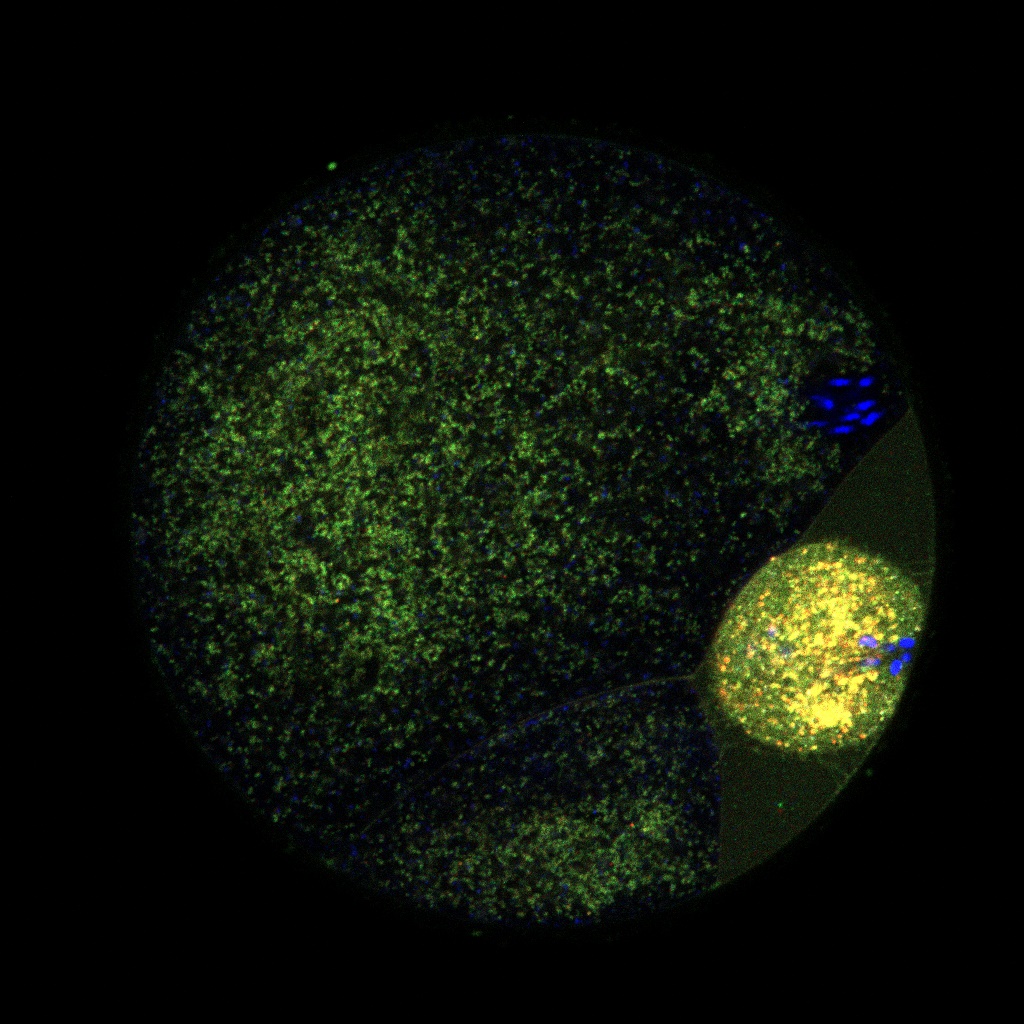

Supplement: Supplementary file 11 — Source Data for Figure 6 [file EMMM-13-e14887-s012.zip › EMM-2021-14887_SDataFig6/Fig. 6F/Erk cko-MII-jc-1-40x-2-Image Export-07/erkcko-mii-jc-1-40x-2-Image Export-07_c1-3.jpg]

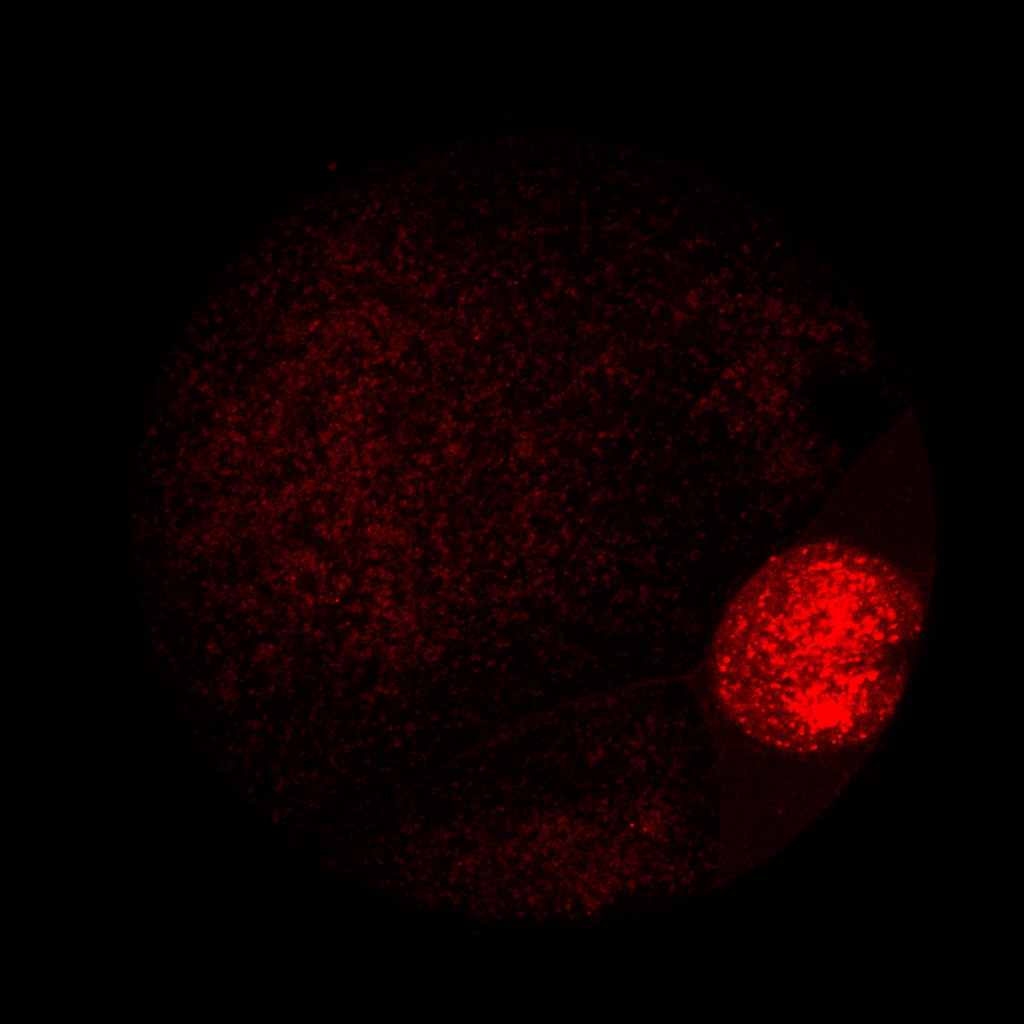

Supplement: Supplementary file 11 — Source Data for Figure 6 [file EMMM-13-e14887-s012.zip › EMM-2021-14887_SDataFig6/Fig. 6F/Erk cko-MII-jc-1-40x-2-Image Export-07/erkcko-mii-jc-1-40x-2-Image Export-07_c1.jpg]

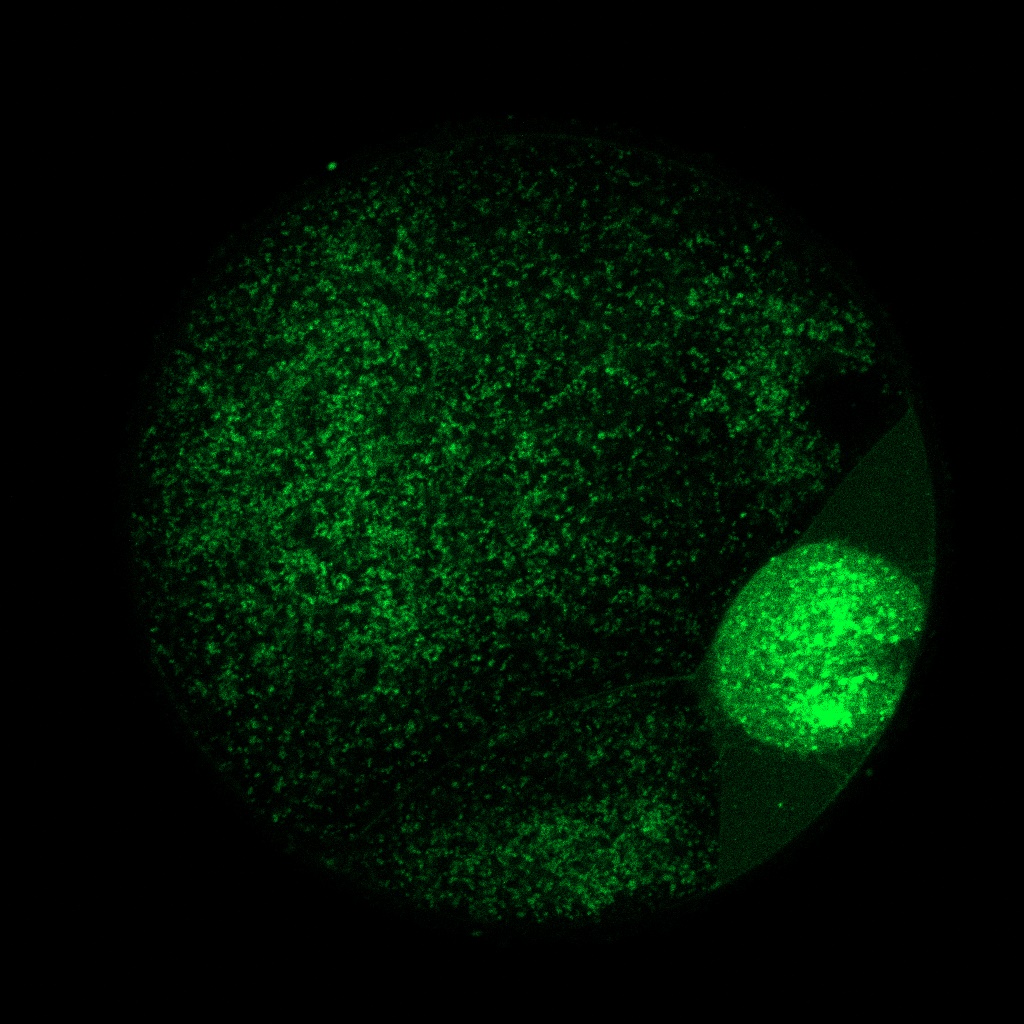

Supplement: Supplementary file 11 — Source Data for Figure 6 [file EMMM-13-e14887-s012.zip › EMM-2021-14887_SDataFig6/Fig. 6F/Erk cko-MII-jc-1-40x-2-Image Export-07/erkcko-mii-jc-1-40x-2-Image Export-07_c2.jpg]

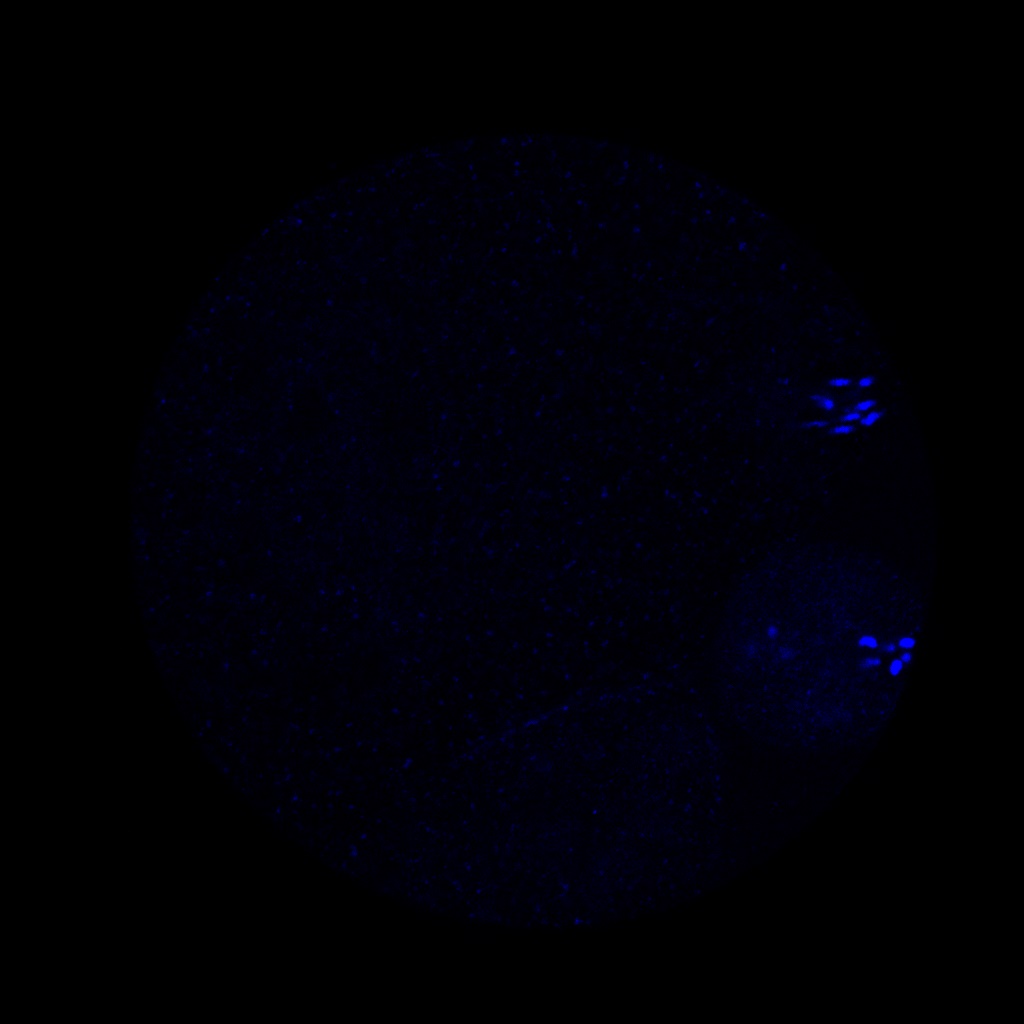

Supplement: Supplementary file 11 — Source Data for Figure 6 [file EMMM-13-e14887-s012.zip › EMM-2021-14887_SDataFig6/Fig. 6F/Erk cko-MII-jc-1-40x-2-Image Export-07/erkcko-mii-jc-1-40x-2-Image Export-07_c3.jpg]

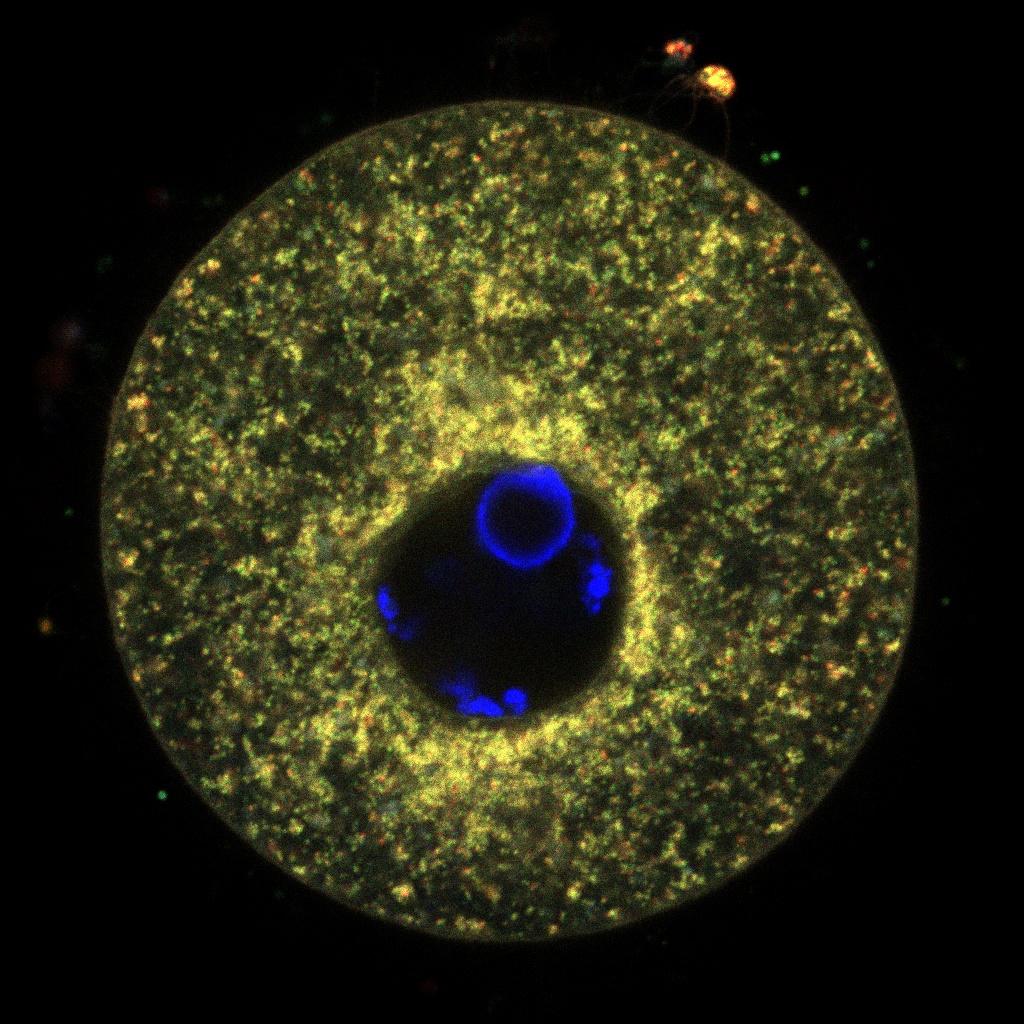

Supplement: Supplementary file 11 — Source Data for Figure 6 [file EMMM-13-e14887-s012.zip › EMM-2021-14887_SDataFig6/Fig. 6F/WT-GV-jc-1-40x-1-Image Export-14/WT-GV-jc-1-40x-1-Image Export-14_c1-3.jpg]

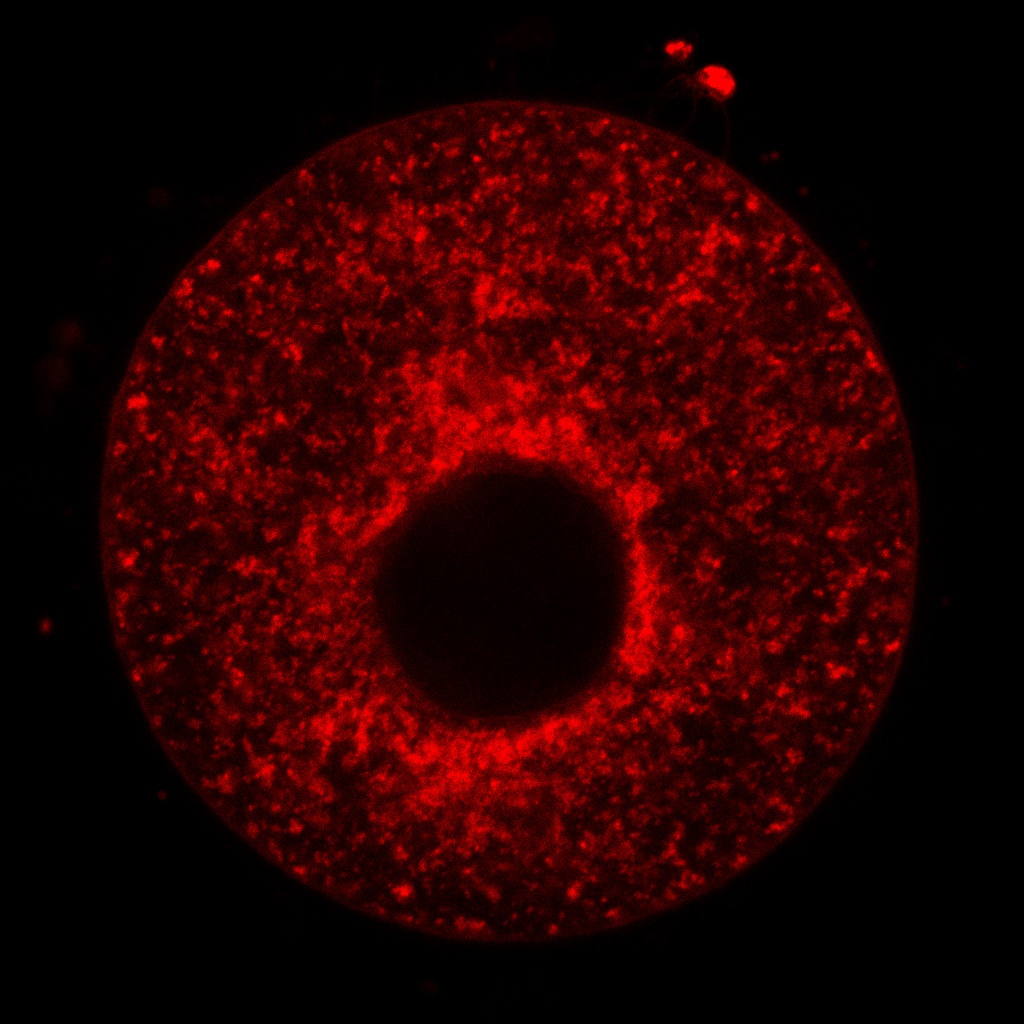

Supplement: Supplementary file 11 — Source Data for Figure 6 [file EMMM-13-e14887-s012.zip › EMM-2021-14887_SDataFig6/Fig. 6F/WT-GV-jc-1-40x-1-Image Export-14/WT-GV-jc-1-40x-1-Image Export-14_c1.jpg]

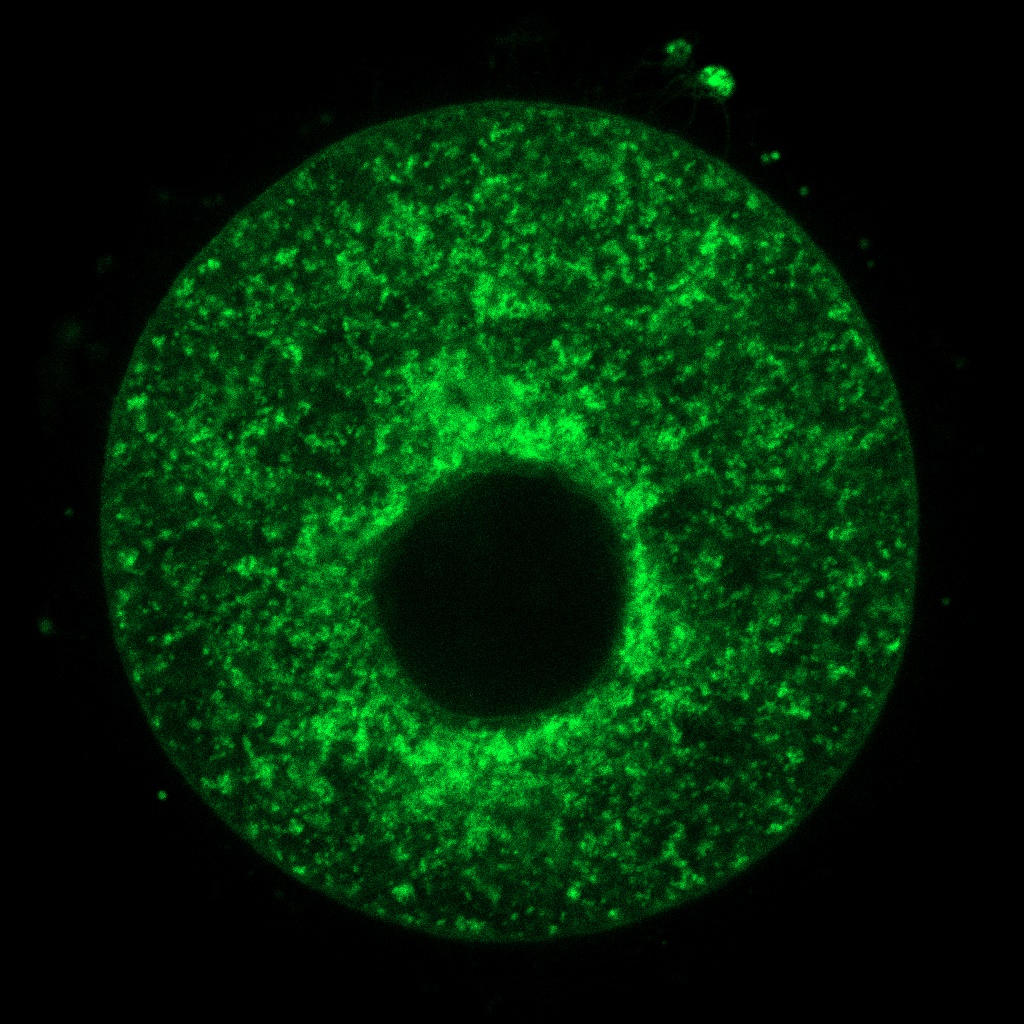

Supplement: Supplementary file 11 — Source Data for Figure 6 [file EMMM-13-e14887-s012.zip › EMM-2021-14887_SDataFig6/Fig. 6F/WT-GV-jc-1-40x-1-Image Export-14/WT-GV-jc-1-40x-1-Image Export-14_c2.jpg]

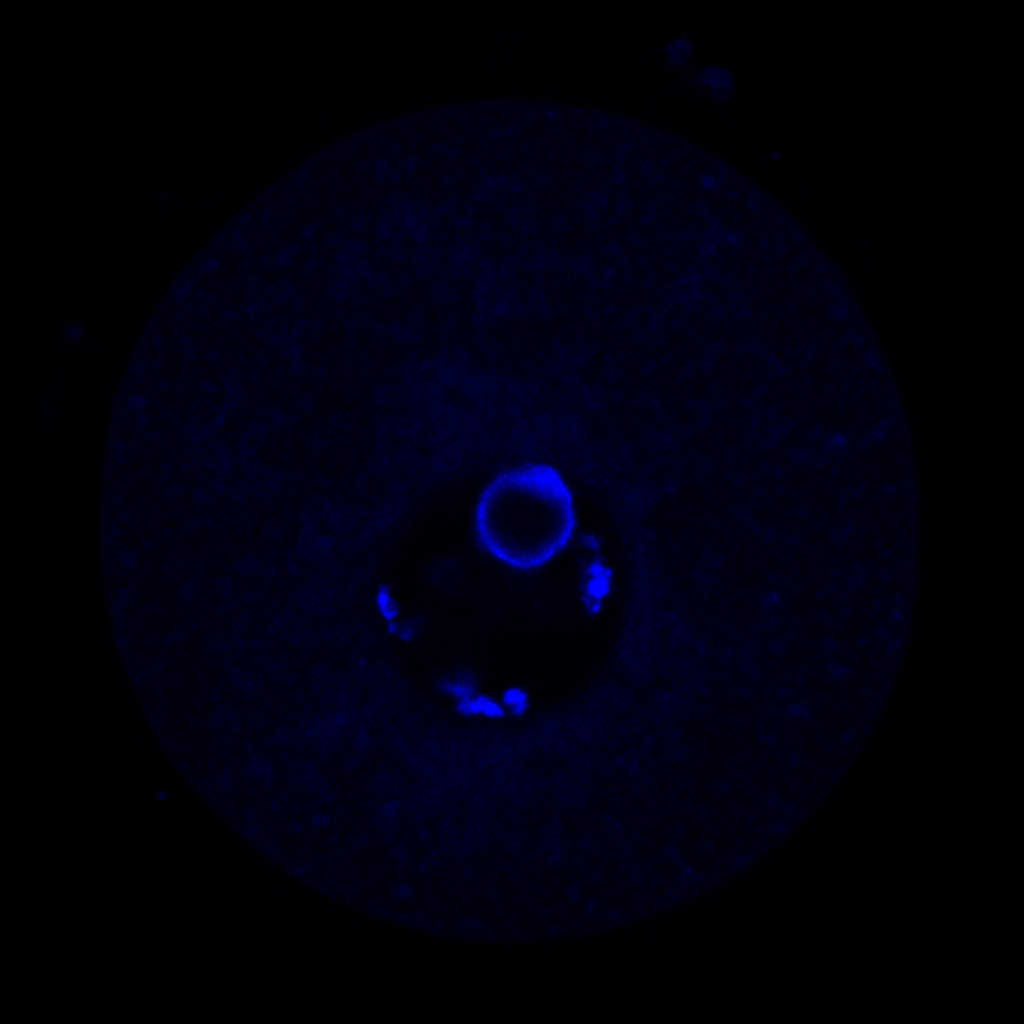

Supplement: Supplementary file 11 — Source Data for Figure 6 [file EMMM-13-e14887-s012.zip › EMM-2021-14887_SDataFig6/Fig. 6F/WT-GV-jc-1-40x-1-Image Export-14/WT-GV-jc-1-40x-1-Image Export-14_c3.jpg]

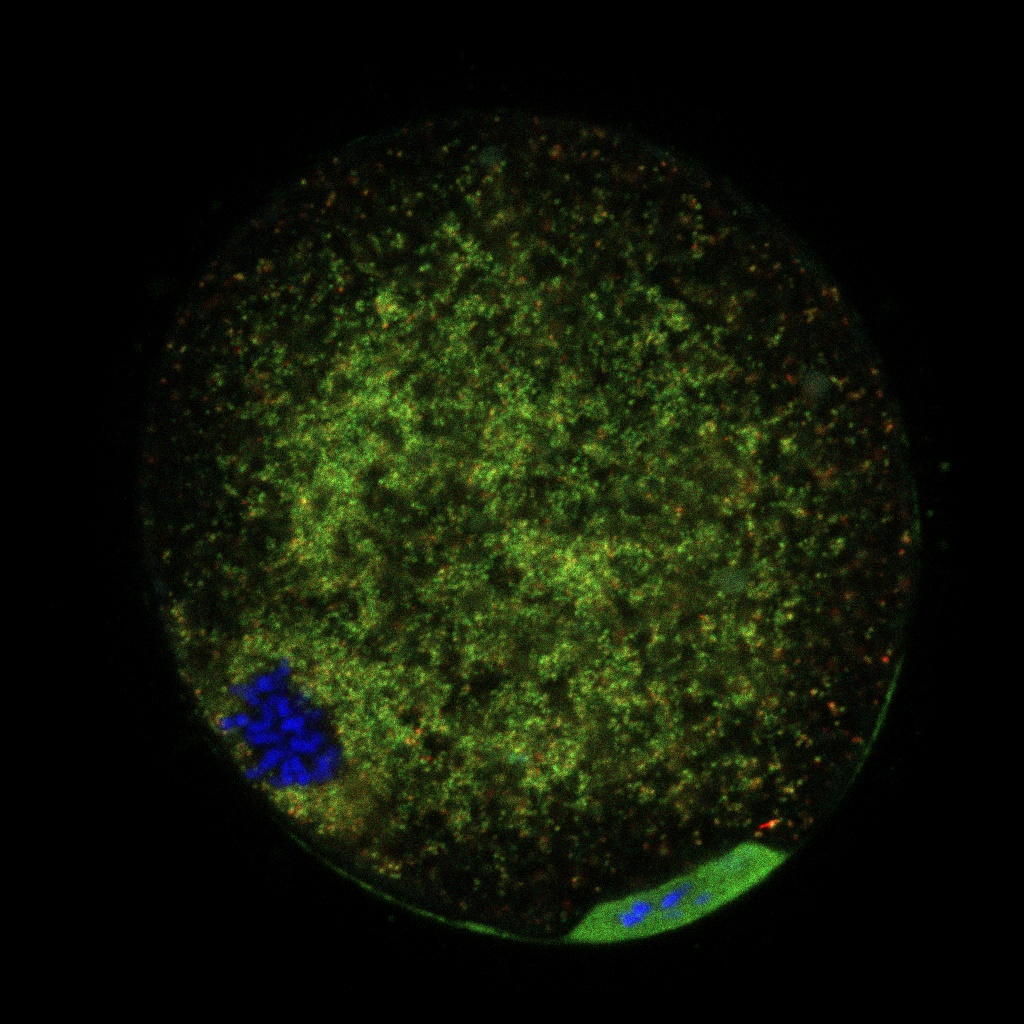

Supplement: Supplementary file 11 — Source Data for Figure 6 [file EMMM-13-e14887-s012.zip › EMM-2021-14887_SDataFig6/Fig. 6F/WT-MII-jc-1-40x-1-Image Export-17/WT-MII-jc-1-40x-1-Image Export-17_c1-3.jpg]

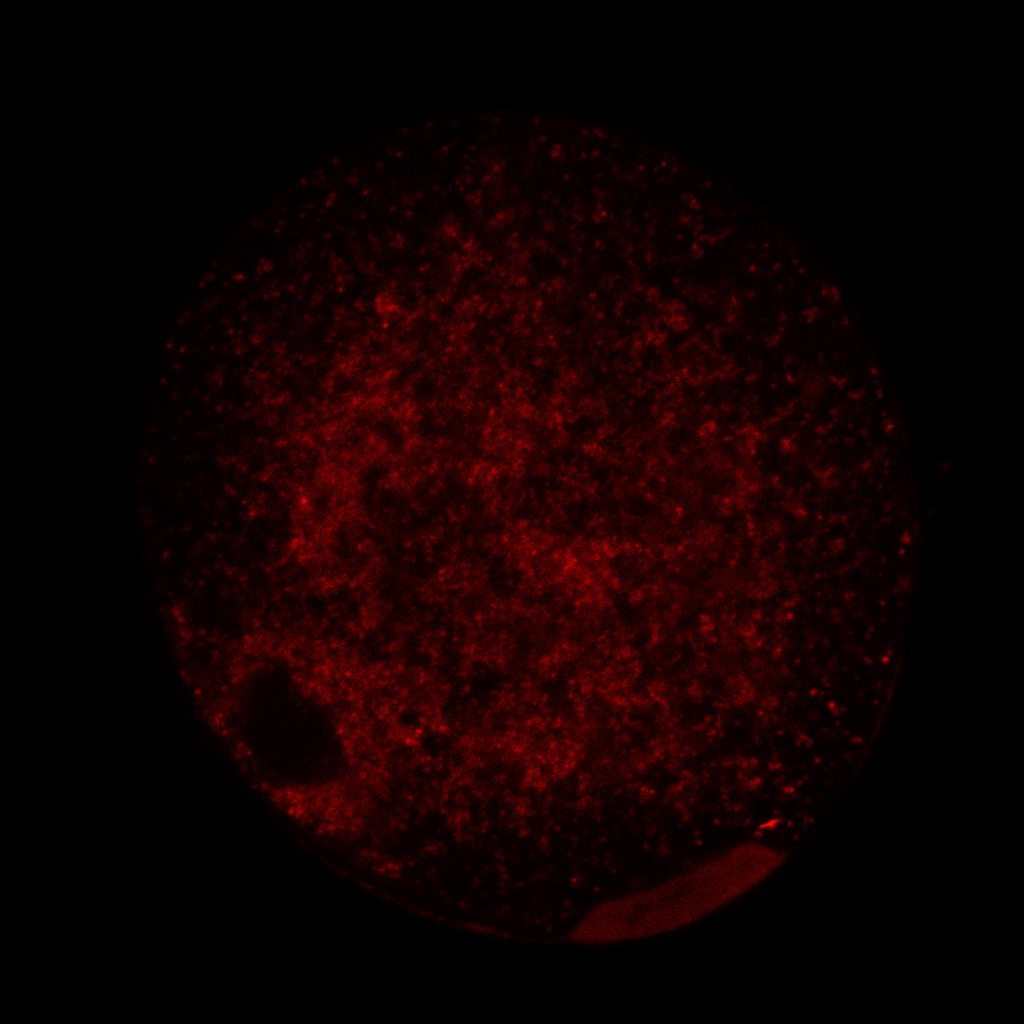

Supplement: Supplementary file 11 — Source Data for Figure 6 [file EMMM-13-e14887-s012.zip › EMM-2021-14887_SDataFig6/Fig. 6F/WT-MII-jc-1-40x-1-Image Export-17/WT-MII-jc-1-40x-1-Image Export-17_c1.jpg]

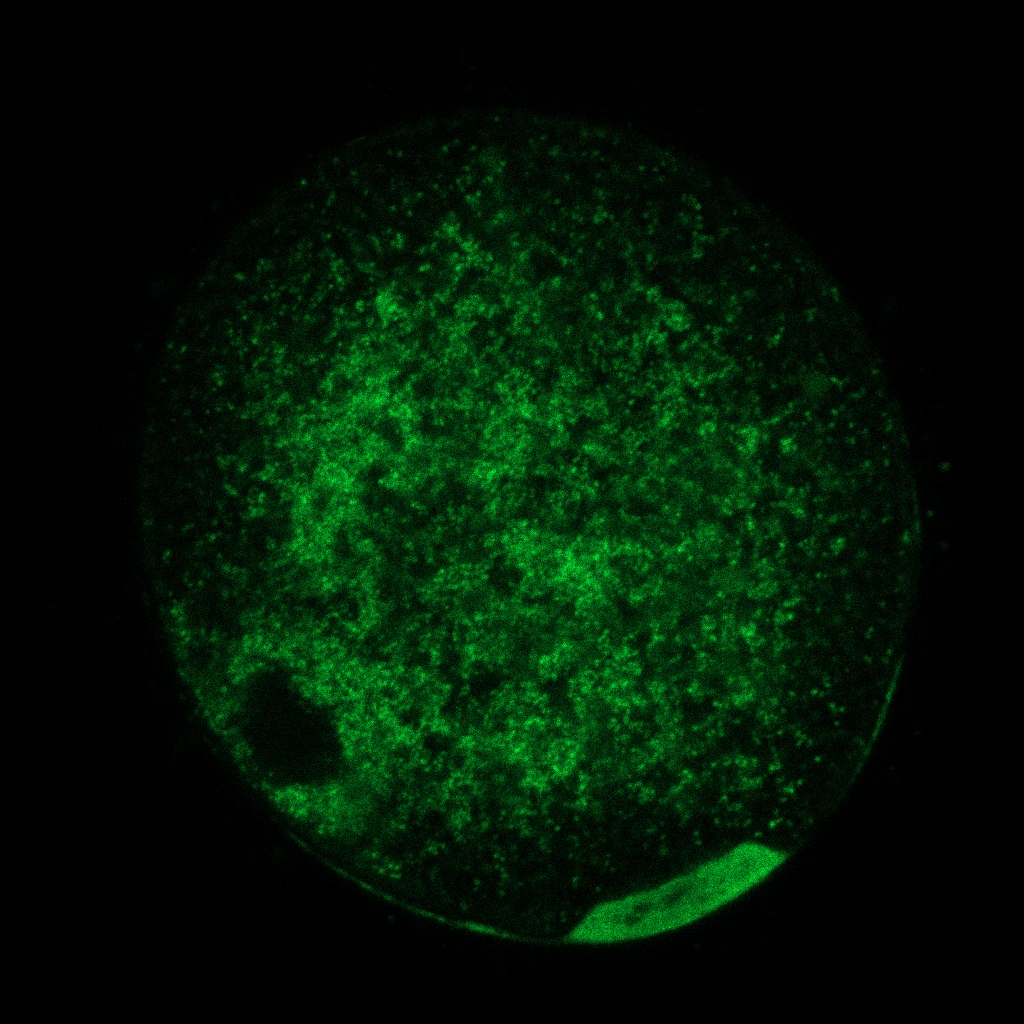

Supplement: Supplementary file 11 — Source Data for Figure 6 [file EMMM-13-e14887-s012.zip › EMM-2021-14887_SDataFig6/Fig. 6F/WT-MII-jc-1-40x-1-Image Export-17/WT-MII-jc-1-40x-1-Image Export-17_c2.jpg]

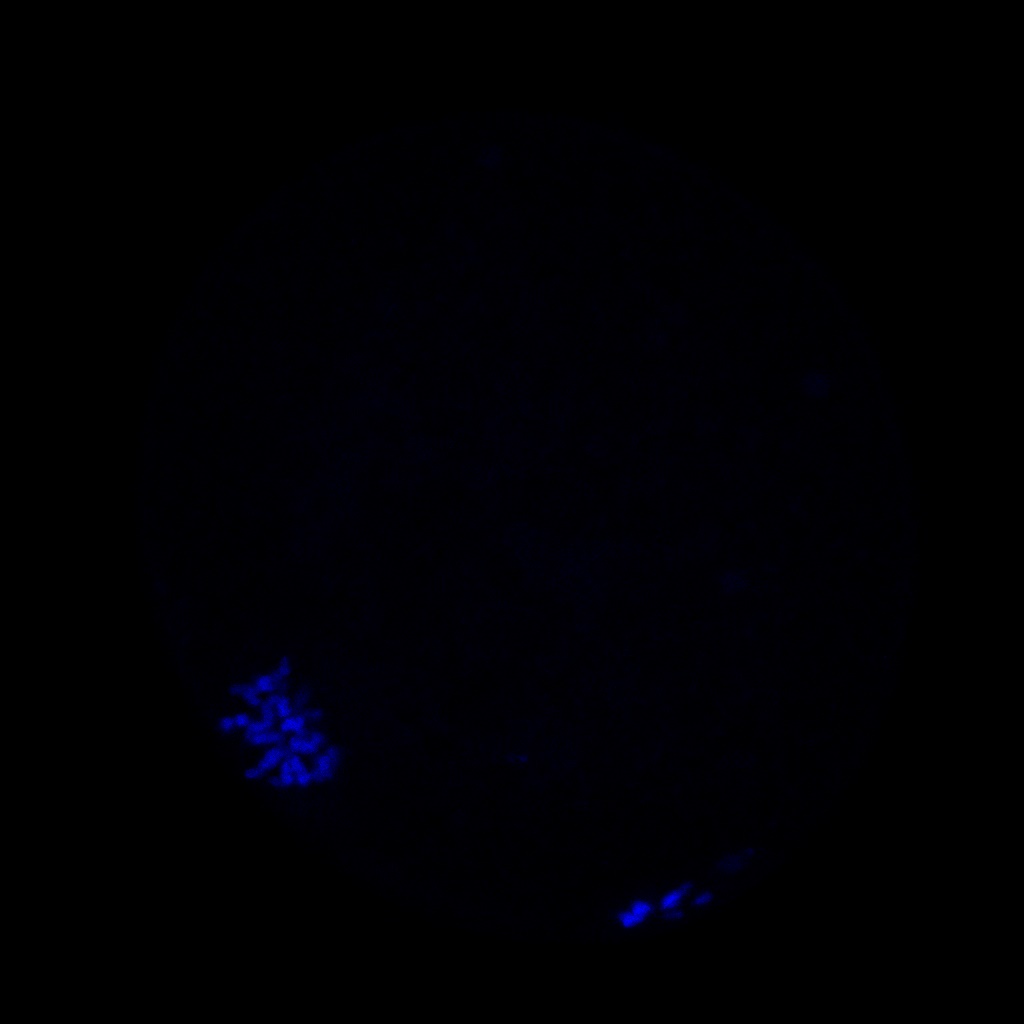

Supplement: Supplementary file 11 — Source Data for Figure 6 [file EMMM-13-e14887-s012.zip › EMM-2021-14887_SDataFig6/Fig. 6F/WT-MII-jc-1-40x-1-Image Export-17/WT-MII-jc-1-40x-1-Image Export-17_c3.jpg]
