# Supplementary material for: Biallelic mutations in MOS cause female infertility characterized by human early embryonic arrest and fragmentation
Source: EMBO Mol Med. 2021 Nov 15;13(12):e14887. doi: 10.15252/emmm.202114887 (PMC8649871; doi:10.15252/emmm.202114887)
Supplement: Supplementary file 12 [file EMMM-13-e14887-s011.pdf]

EMBO Molecular Medicine

Biallelic mutations in MOS cause female infertility characterized by human early embryonic arrest and fragmentation

Songying Zhang, Yinli Zhang, Wei Zheng, Peipei Ren, Huiling Hu, Xiaomei Tong, Shuoping Zhang, Xiang Li, Haichao Wang, Jun-Chao Jiang, Jiamin Jin, Weijie Yang, Lanrui Cao, Yuanlin He, Yerong Ma, Yingyi Zhang, Yifan Gu, Liang Hu, Keli Luo, Fei Gong, Guangxiu Lu, Ge Lin, and Heng-Yu Fan
DOI: 10.15252/emmm.202114887

Corresponding authors: Songying Zhang (zhangsongying@zju.edu.cn) , Heng-Yu Fan (hyfan@zju.edu.cn), Ge Lin (linggf@hotmail.com)

Review Timeline:

Submission Date:	25th Jul 21
Editorial Decision:	12th Aug 21
Revision Received:	15th Sep 21
Editorial Decision:	5th Oct 21
Revision Received:	19th Oct 21
Accepted:	22nd Oct 21

Editor: Zeljko Durdevic

Transaction Report:

(Note: With the exception of the correction of typographical or spelling errors that could be a source of ambiguity, letters and reports are not edited. Depending on transfer agreements, referee reports obtained elsewhere may or may not be included in this compilation. Referee reports are anonymous unless the Referee chooses to sign their reports.)

Dear Dr. Zhang,

Thank you for the submission of your manuscript to EMBO Molecular Medicine. We have now received feedback from the three reviewers who agreed to evaluate your manuscript. As you will see from the reports below, the referees acknowledge the interest of the study but also raise important critique that should be addressed in a major revision.

Further consideration of a revision that addresses reviewers' concerns in full will entail a second round of review. EMBO Molecular Medicine encourages a single round of revision only and therefore, acceptance or rejection of the manuscript will depend on the completeness of your responses included in the next, final version of the manuscript. For this reason, and to save you from any frustrations in the end, I would strongly advise against returning an incomplete revision.

We would welcome the submission of a revised version within three months for further consideration. However, we realize that the current situation is exceptional on the account of the COVID-19/SARS-CoV-2 pandemic. Please let us know if you require longer to complete the revision.

I look forward to receiving your revised manuscript.
Use this link to login to the manuscript system and submit your revision: https://embomolmed.msubmit.net/cgi-bin/main.plex

Yours sincerely,

Zeljko Durdevic

Zeljko Durdevic
Editor
EMBO Molecular Medicine

***** Reviewer's comments *****

Referee #1 (Remarks for Author):

In this work Yin-Li Zhang and colleagues study the mechanism of early embryonic arrest and fragmentation (EEAF) through genetic, cellular and molecular studies. They studied three patients with EEAF by WES and found pathogenic variants in of the same gene MOS a serine-threonine kinase that activates the ERK signaling cascade during oocyte maturation in vertebrates. Using cellular system i.e. HEK cells, human oocytes with overexpression of the MOS control or mutated mRNAs and a rodent model (ERK1/2 knock out mice generated by this group), they could demonstrate that the variants alter MOS expression or ERK1/2 activation, and the MOS-ERK pathway is required in oocytes for cytoskeleton assembly and prevents embryo fragmentation. RNA sequencing in single oocytes of one patient has shown that MOS regulates the degradation of maternal mRNAs during oocyte maturation and that the variants detected in patients mainly prevent the decrease in transcripts from human GV to MII oocytes, similarly to the inhibition of ERK1/2. Finally they studied mitochondrial functions in human and murine MII oocytes after U0126 treatment and in mature oocytes in vivo from WT and ERK1/ -/- mice that this group has generated. They conclude that the human MOS/ ERK cascade is required to maintain mitochondrial function through accelerating mitochondrial mRNA clearance during oocyte maturation.
This work is original, well done, with a large spectrum of combined approaches, in vitro or in vivo, in humans or in rodents, to be able to understand the mechanism of EEAF and associated female infertility, which was not explained by the previously reported genetic causes. It provides important information on the signaling pathway that drives human oocyte maturation to prevent embryo arrest and fragmentation. These results might have a therapeutic impact in the future.
Specific comments:
Patients: are they siblings in the families with potential fertility problems? This is not shown in the pedigrees presented. Are the patients from the same country? No information on their origin is given.
It is striking to observe in the various experiments a minor inhibition of ERK1/2 or MEK activation with the combined p.Met139Thr/p. Arg 246His variants (Fig 2C, EV1D). This is not discussed in the work presented. Are there differences in the EEAF phenotype between the different patients that might be correlated with these differences in ERK1/2 inhibition?
It appears that there is only about 25% alteration of resumption of meiosis with the combined variants, while this alteration is much greater for the other Asn95Lys and Cys320Ter variants. Precise quantification must be performed.
The question is raised as to whether they might be a threshold for ERK activation to allow correct oocyte maturation. In this respect a range of transfected RNAs (control or mutated) could provide the answer. A single dose 500 ng is used in all experiments.

RNA sequencing could also be performed in the oocytes of patient 2 harboring the combined mutations. Fewer RNAs are expected to be deregulated. This could lead to the identification of the functionally most important RNAs deregulated with this milder variant.
Since there is transient expression of MOS, overexpression of WT MOS could be used to compensate for the molecular defects. Do the authors try such experimental approaches in vitro as proof of concept or in mice in vivo and observe the consequences on blastocyst formation and embryonic development?

Referee #2 (Remarks for Author):

This is a solid and comprehensive paper reporting that deleterious alleles of the MOS gene can cause infertility in human females. The authors find 3 pedigrees in which IVF attempts failed due to early arrest of embryos. Exome sequence revealed biallelic mutations of Mos in probands that were inherited from parents. What sets this paper apart from other papers that stop at this gene:phenotype correlative stage is that the authors go on to conduct a number of studies proving that these alleles encode functionally defective proteins that compromise oocyte development and embryo progression. They even make some interesting fundamental observations using genomic transcriptome studies that resulted in the discovery of mitochondrial defects when the MOS s defective.

I found that the paper was well-prepared, especially the figures, although some light English editing would help refine it. I only

have a few minor suggestions for this otherwise welcome report:

Line 116: "....confirming the possible genetic contribution of MOS to human EFAF." The word "confirming" is not appropriate. "Suggesting" would be a more accurate word at this point in the paper.

Line 131/Table 2: It would be useful to provide the actual SIFT and PolyPhen scores in the Table. Also, many computational people like to see CADD scores, for what they're worth.

In the Introduction, it would be helpful to further explain how ERK1/2 phosphorylation maintains MII arrest.

Line 156: It is not obvious or rigorously demonstrated that ".... MOS and MOS variants led to decreased MOS protein levels (Figure 2C and D)."

On line 161, explain the purpose of milrinone.

Referee #3 (Comments on Novelty/Model System for Author):

Early embryonic arrest and fragmentation (EEAF) is a common phenomenon in human embryos. However, the role of MOS in human oocyte maturation and early embryo development is not fully uncovered. In this study, the authors validated the pathogenic roles of four rare MOS variants identified in three individuals exhibiting EEAF. The pathogenic effects of MOS variants were investigated in HEK293 cells and mouse oocytes, including the Erk1/2 conditional knockout mice. Combined with in vivo and in vitro RNA-seq results from control, patient oocytes and U0126-treated oocytes, they concluded that MOS variants caused ERK1/2 signal cascade inactivation, resulted in cortical F-actin assembly defect and impaired maternal mRNA decay, especially on genes relative to mitochondrial function.
The study reveals the important role of maternal MOS in human embryo development and stability. The conclusion is interesting in identifying the association of MOS pathogenic variants and female infertility and establishing MOS-ERK1/2 signal pathway as the critical factors for human oocyte cytoplasmic maturation by maternal mRNA clearance. The conclusion is appropriate and evidenced by the results. Besides, the manuscript is well written. I support for the publication of this manuscript, with an expectation that some aspects of current manuscript could be improved as mentioned in remarks to the author.

Referee #3 (Remarks for Author):

Major points:
1. How many patients were selected for whole exon sequencing in this study? What are the inclusion criteria for the patient with EEAF? Please indicate them in the method or the results. Why do the authors focus on these three patients?
2. Please descript the characteristics of 100-control individuals you needed in this study.
3. Please provide more detail clinical information for the patients, such as hormone and chromosome status.
4. Was the functional role of MOS the same between human and mouse? I noticed that the corresponding amino acids of two identified variants were not conserved between human and mouse.
5. Except RNA-seq data, RT-qPCR was needed to further validate the affected mitochondrial mRNA clearance.
6. Apart from U0126 treatment, it is better to detect mitochondrial dysfunction using Erk1/2 deficient mice.

Minor points:
1. How to explain the decreased pMEK1/2 and pERK1/2 level with unchanged protein levels in patient 2?
2. In Fig. 1D, "Blastocst" should be "Blastocyst".
3. In the discussion, the relationship between MOS-ERK1/2 pathway and mitochondrial function should be added.

Dear editor,

Thanks for editor and reviewers' constructive comments to improve the manuscript.

As suggested, we have re-submitted a .docx formatted version of revised manuscript with “tracked-changes mode”. We have checked each figure according to the guidance of “Figure Guide PDF” of EMBO Molecular Medicine journal. We included the information of ORCID IDs of corresponding authors, Data Availability and Author Contributions in the manuscript, and checked data quality as required. The checklist and source data of each figures have been submitted to the editorial system. In the light of reviewers’ comments, we have made point-by-point response and performed some experiments and added these new results in Appendix Figure S1, S3 and S4. These Supplementary information is uploaded as pdf (without highlighted changes).

Please find the point-by-point response to reviewers' comments below:

***** Reviewer's comments *****

Referee #1 (Remarks for Author):

In this work Yin-Li Zhang and colleagues study the mechanism of early embryonic arrest and fragmentation (EEAF) through genetic, cellular and molecular studies. They studied three patients with EEAF by WES and found pathogenic variants in of the same gene MOS a serine-threonine kinase that activates the ERK signaling cascade during oocyte maturation in vertebrates. Using cellular system i.e. HEK cells, human oocytes with overexpression of the MOS control or mutated mRNAs and a rodent model (ERK1/2 knock out mice generated by this group), they could demonstrate that the variants alter MOS expression or ERK1/2 activation, and the MOS-ERK pathway is required in oocytes for cytoskeleton assembly and prevents embryo fragmentation. RNA sequencing in single oocytes of one patient has shown that MOS regulates the degradation of maternal mRNAs during oocyte maturation and that the variants detected in patients mainly prevent the decrease in transcripts from human GV to MII oocytes, similarly to the inhibition of ERK1/2. Finally they studied mitochondrial functions in human and murine MII oocytes after U0126 treatment and in mature oocytes in vivo from WT and ERK1/ -/- mice that this group has generated. They conclude that the human MOS/ ERK cascade is required to maintain mitochondrial function through accelerating mitochondrial mRNA clearance during oocyte maturation.

This work is original, well done, with a large spectrum of combined approaches, in vitro or in vivo, in humans or in rodents, to be able to understand the mechanism of EEAF and associated female infertility, which was not explained by the previously reported genetic causes. It provides important information on the signaling pathway that drives human oocyte maturation to prevent embryo arrest and fragmentation. These results might have a therapeutic impact in the future.

Response: Thank you for your positive assessment.

Specific comments:

Patients: are they siblings in the families with potential fertility problems? This is not shown in the pedigrees presented. Are the patients from the same country? No information on their origin is given.

Response: The three patients are not from the families with potential fertility problems. They all

are from China and belong to Chinese Han population. We have included this information into Line 104-105 in the revised manuscript.

It is striking to observe in the various experiments a minor inhibition of ERK1/2 or MEK activation with the combined p.Met139Thr/p. Arg 246His variants (Fig 2C, EV1D). This is not discussed in the work presented. Are there differences in the EEAF phenotype between the different patients that might be correlated with these differences in ERK1/2 inhibition?

Response: Thanks for pointing it out. Based on our new added results in Appendix Figure S1, the MOS p.Met139Thr variant caused ERK1/2 inactivation, similar to the other two homozygous variants p.Asn95Lys and p.Cys320Ter, yet the MOS p.Arg246His variant decreased the ability of binding with MEK1 and thereby just decreased ERK1/2 activation to some extent. We microinjected the combined variants with same dose (250 ng/ul mRNAs for each variant, reaching a total dose of 500 ng/ul) in mouse oocyte to mimic the compound heterozygous variants of patient 2. However, we neglect there exists allelic gene expression imbalance in human. If possible, we might validate this by comparing the expression level of the two alleles in future through performing the RNA-seq using oocyte from patient 2. We have added this into Discussion part (Line 381-390).

We approve that the differences in the EEAF phenotype between the different patients are correlated with these differences in ERK1/2 inhibition. The patient 3 carries with a homozygous nonsense variant in MOS gene, causing premature termination at 320th amino acid. The p.C320Ter variant displayed the weakest interaction with MEK1 (Appendix Figure S1B-C) and resulted in lowest ERK1/2 activation (Figure 2C and Figure EV1D). Consistently, the patient 3 exhibited the earliest embryonic arrest (2-3 cell stage) than those of other two patients (2-7 cell stage). We have added this into Discussion part (Line 372-378).

It appears that there is only about 25% alteration of resumption of meiosis with the combined variants, while this alteration is much greater for the other Asn95Lys and Cys320Ter variants. Precise quantification must be performed.

Response: We are apologized for your confusion due to inaccurate description. We have re-checked the mRNA concentration used for microinjection and we make sure that a total 500 ng/ul mRNAs (each variant with 250 ng/ul in the combined p.Met139Thr/p.Arg246His variants group) were used for microinjection in three independent experiments. We have added this into Materials and Method (Line 566-568). We also found the ability of resumption of meiosis is associate with the level of MOS-mediated ERK1/2 signal cascade activation. The source data of this result is provided.

The question is raised as to whether they might be a threshold for ERK activation to allow correct oocyte maturation. In this respect a range of transfected RNAs (control or mutated) could provide the answer. A single dose 500 ng is used in all experiments.

Response: Thank you for this important and helpful idea. Because the 500 ng/ul is commonly used concentration in other published papers and this microinjection dose is sufficient, we selected this single dose 500 ng/ul to confirm the pathogenic roles of four *MOS* variants. As you suggested, we microinjected different concentrations of *MOS* mRNAs (0, 50, 125, 250 and 500 ng/ul) into mouse GV oocytes to determine the threshold of *MOS* mRNAs on ERK1/2 activation to allow

correct oocyte maturation. We found 50 ng/ul and 125ng/ul *MOS* mRNAs are sufficient to promote 35% and 60% oocytes to resume meiotic maturation, respectively. With the increase of *MOS* mRNAs from concentrations of 125ng/ul to 500 ng/ul, oocyte maturation percentage maintained around 50%-60%. Thus, we can conclude that there exists a threshold of *MOS* mRNAs for ERK1/2 activation. We have added this into Discussion part (Line 390-396).
Based on this threshold theory, we could answer your last question "It appears that there is only about 25% alteration of resumption of meiosis with the combined variants…". Except to the imbalanced allelic gene expression, we thought that the 500 ng/ul of mRNAs of the combined MOS p.Met139Thr/p.Arg246His variants are probably overdosed, resulting in relative high maturation rate. We microinjected a total dose of 125 ng/ul wild-type *MOS* mRNAs or the combined p.Met139Thr/p.Arg246His variants mRNAs (each with 62.5 ng/ul) into immature mouse oocytes. We found that the wild-type MOS promotes 53% oocyte maturation, whereas only 12% oocytes resumed meiotic maturation in the combined p.Met139Thr/p.Arg246His variants group, which verified our hypothesis. These results were included in newly Appendix Figure S4 and described in Discussion section (Line 396-402) in the revised manuscript.

RNA sequencing could also be performed in the oocytes of patient 2 harboring the combined mutations. Fewer RNAs are expected to be deregulated. This could lead to the identification of the functionally most important RNAs deregulated with this milder variant.
Response: Thanks for your suggestion. As patient 2 did not agree to donate oocyte to perform experiment, we didn't perform RNA-seq. Unfortunately, we also didn't obtain the oocytes in patient 3. Indeed, we can identify the functionally most important RNAs regulated by MOS through comparing the RNA-seq data from oocytes with different variants. Thanks again for your helpful suggestion.

Since there is transient expression of MOS, overexpression of WT MOS could be used to compensate for the molecular defects. Do the authors try such experimental approaches in vitro as proof of concept or in mice in vivo and observe the consequences on blastocyst formation and embryonic development?
Response: Thank you for your professional and helpful suggestion. In restriction of *Mos* deficiency mice is in the process of generation and is available at least half a year, we didn't perform this experiment. In the our next study, we will focus on the rescuing effect of *MOS* mRNAs on *Mos*$^{-/-}$ mice and patients with *MOS* deleterious variants. At that time, your question can be answered. I believe an appropriate dose of *MOS* mRNA or MOS protein could compensate for the molecular defects of oocyte and embryos from *Mos*$^{-/-}$ mice and patients. Furthermore, the rescuing effect and the potential pathogenic effect of overexpression of *MOS* should carefully evaluated. This question indeed requires many experiments to investigate.

Referee #2 (Remarks for Author):

This is a solid and comprehensive paper reporting that deleterious alleles of the MOS gene can cause infertility in human females. The authors find 3 pedigrees in which IVF attempts failed due

to early arrest of embryos. Exome sequence revealed biallelic mutations of Mos in probands that were inherited from parents. What sets this paper apart from other papers that stop at this gene:phenotype correlative stage is that the authors go on to conduct a number of studies proving that these alleles encode functionally defective proteins that compromise oocyte development and embryo progression. They even make some interesting fundamental observations using genomic transcriptome studies that resulted in the discovery of mitochondrial defects when the MOS s defective.

Response: Thank you for your positive evaluation of this study.

I found that the paper was well-prepared, especially the figures, although some light English editing would help refine it. I only have a few minor suggestions for this otherwise welcome report:

Line 116: "....confirming the possible genetic contribution of MOS to human EFAF." The word "confirming" is not appropriate. "Suggesting" would be a more accurate word at this point in the paper.

Response: Thanks for your advice for improving our manuscript. We have corrected it in Line 132 in the new version of manuscript.

Line 131/Table 2: It would be useful to provide the actual SIFT and PolyPhen scores in the Table. Also, many computational people like to see CADD scores, for what they're worth.

Response: This is a very professional comment. We have added these scores in Table 2 and described it in Line 147-149.

In the Introduction, it would be helpful to further explain how ERK1/2 phosphorylation maintains MII arrest.

Response: Thanks for your helpful suggestion to improve the readability of the manuscript. We have added the description of mechanism about ERK1/2 phosphorylation maintaining MII arrest in Line 79-80. Also, we explained the mechanism of MOS-ERK signal cascade in promotion of meiotic maturation in Line 84-87 in the revised manuscript.

Line 156: It is not obvious or rigorously demonstrated that ".... MOS<Asn95Lys> and MOS<Cys320Ter> variants led to decreased MOS protein levels (Figure 2C and D)."

Response: Yes, this description is not rigorous. We have modified this to "MOS^{Asn95Lys} and MOS^{Cys320Ter} variants encoded MOS protein with slight decrease" in Line175-176 in the revised manuscript.

On line 161, explain the purpose of milrinone.

Response: The milrinone is a PDE3 inhibitor, which is used to prevent oocyte from meiotic resumption by maintaining elevated cAMP levels in oocytes. We have added this information into Line 190-191.

Referee #3 (Comments on Novelty/Model System for Author):

Early embryonic arrest and fragmentation (EEAF) is a common phenomenon in human embryos. However, the role of MOS in human oocyte maturation and early embryo development is not fully uncovered. In this study, the authors validated the pathogenic roles of four rare MOS variants identified in three individuals exhibiting EEAF. The pathogenic effects of MOS variants were investigated in HEK293 cells and mouse oocytes, including the Erk1/2 conditional knockout mice. Combined with in vivo and in vitro RNA-seq results from control, patient oocytes and U0126-treated oocytes, they concluded that MOS variants caused ERK1/2 signal cascade inactivation, resulted in cortical F-actin assembly defect and impaired maternal mRNA decay, especially on genes relative to mitochondrial function.

The study reveals the important role of maternal MOS in human embryo development and stability. The conclusion is interesting in identifying the association of MOS pathogenic variants and female infertility and establishing MOS-ERK1/2 signal pathway as the critical factors for human oocyte cytoplasmic maturation by maternal mRNA clearance. The conclusion is appropriate and evidenced by the results. Besides, the manuscript is well written. I support for the publication of this manuscript, with an expectation that some aspects of current manuscript could be improved as mentioned in remarks to the author.

Response: Thank you for your appreciation on this work.

Referee #3 (Remarks for Author):

Major points:

1. How many patients were selected for whole exon sequencing in this study? What are the inclusion criteria for the patient with EEAF? Please indicate them in the method or the results. Why do the authors focus on these three patients?

Response: We apologize for the unclear description of this part. The EEAF patients were screened according to the following criteria: primary infertility; normal karyotype in both couples; male factors excluded; experienced recurrent failure of IVF/ICSI attempts suffering embryonic arrest and more than 50% of the cleaved embryo represented severer embryo fragmentation occurred in at least one attempt. We have selected 120 patients that experienced EEAF to WES, and just three patients herein carried biallelic mutations in *MOS*. We have added the relevant description to the Materials and Methods section (Line 479-485).

2. Please descript the characteristics of 100-control individuals you needed in this study.

Response: The 100-controls were women who had conceived naturally, without medical intervention. This has been clearly described in the results of the revised manuscript (Line 132).

3. Please provide more detail clinical information for the patients, such as hormone and chromosome status.

Response: These three females possessed the normal endocrine level and karyotype, we have added related information in Appendix Table S1 and described it in Line 106 of revised manuscript.

4. Was the functional role of MOS the same between human and mouse? I noticed that the corresponding amino acids of two identified variants were not conserved between human and mouse.

Response: These are good and very interesting questions. I think your first question is focus on the function of MOS protein, whereas the second question is focus on the different amino acids on MOS's function. *MOS* gene is firstly discovered in *Xenopus* for its role in maintaining MII arrest. Then, many studies demonstrated that MOS is required in mouse oocyte maturation and MII arrest. From gene evolutionary perspective, the amino acids of MOS in *Xenopus* are very different from those of human MOS, but the main function of MOS is conserved. In our study, we also found the functional roles of MOS on activation of ERK1/2 cascade signal and requirement of cytoplasmic maturation are conserved between human and mouse. Several studies demonstrated human being is speeding the evolution of mouse (Mouse Genome Sequencing *et al*, 2002; Vrbanec *et al*, 2021). Thus, we suggest that the un-conserved amino acids in MOS (p.Asn95 and p.Arg246) may be hotspot sites and play key roles in determining MOS's activity. Unfortunately, the MOS p.Asn95Lys and MOS p.Arg246His variants are deleterious. I guess the MOS p.Asn95Asp and MOS p.Arg246Gln may facilitate the function of MOS. The difference of the functional role of MOS between human and mouse requires further investigation. For example, the kinase activity of human and mouse MOS protein may differ.

Reference:

1. Mouse Genome Sequencing C, Waterston RH, Lindblad-Toh K, Birney E, Rogers J, Abril JF, Agarwal P, Agarwala R, Ainscough R, Alexandersson M *et al* (2002) Initial sequencing and comparative analysis of the mouse genome. *Nature* 420: 520-562

2. Vrbanec L, Matijevic V, Guenther A (2021) Enhanced problem-solving ability as an adaptation to urban environments in house mice. *Proc Biol Sci* 288: 20202504

5. Except RNA-seq data, RT-qPCR was needed to further validate the affected mitochondrial mRNA clearance.

Response: Thanks for your professional suggestion. We have performed RT-qPCR of human oocyte with or without U0126 treatment and included these results into Appendix Figure S4.

6. Apart from U0126 treatment, it is better to detect mitochondrial dysfunction using Erk1/2 deficient mice.

Response: Thanks for your professional suggestion. We used *Erk1/2* deficient oocytes to determine the mitochondrial function. Consistent results were obtained with those after U0126 treatment. We found a very interesting phenomenon that many mitochondria is extruded into polar bodies by staining JC-1. These results have been presented in Figure 6 E-I and corresponding descriptions have been added in results (Line 323-332) in the manuscript.

Minor points:

1. How to explain the decreased pMEK1/2 and pERK1/2 level with unchanged protein levels in patient 2?

Response: MOS is a serine/threonine kinase that directly interacts and phosphorylates MEK1/2 and thereby activates ERK1/2 phosphorylation. We performed co-immunoprecipitation of MEK1 and MOS variants, we found MOS p.Arg246His interacted weaker than wild-type MOS with MEK1 (Appendix Figure S1B-C), which may lead to decreased pMEK1/2 and pERK1/2 level with unchanged protein levels.

2. In Fig. 1D, "Blastocst" should be "Blastocyst".

Response: Thanks for your careful revision. We have corrected it in Figure 1D.

3. In the discussion, the relationship between MOS-ERK1/2 pathway and mitochondrial function should be added.

Response: As suggested, we have added the relationship between MOS-ERK1/2 pathway and mitochondrial function in Discussion part (Line 453-468) in the revised manuscript.

Dear Prof. Zhang,

Thank you for the submission of your revised manuscript to EMBO Molecular Medicine. I am pleased to inform you that we will be able to accept your manuscript pending the following final amendments:

1) In the main manuscript file, please do the following:
- Correct/answer the track changes suggested by our data editors by working from the attached document.
- Reduce keywords to max. 5.
- In M&M, a statistical paragraph that should reflect all information that you have filled in the Authors Checklist, especially regarding randomization, blinding, replication.
- In M&M, include a statement that informed consent was obtained from all human subjects and that the experiments conformed to the principles set out in the WMA Declaration of Helsinki and the Department of Health and Human Services Belmont Report.
- Use initials for author contributions.
2) Datasets: Please add legend and title in the separate tab of both files.
3) Source data: Please upload one file per figure for the main Figures (zipp were appropriate) and zipp source data for all EV Figures as one file.
4) The Paper Explained: Please shorten the text in "Problem" and "Results" and revise it for grammar and syntax (i.e. by an English native speaker). Please refer to any of our published primary research articles for an example.
5) Synopsis:
- Synopsis text: Please provide a short stand first (maximum of 300 characters, including space) as well as 2-5 one sentence bullet points that summarise the paper as a .doc file. Please write the bullet points to summarise the key NEW findings. They should be designed to be complementary to the abstract - i.e. not repeat the same text. We encourage inclusion of key acronyms and quantitative information (maximum of 30 words / bullet point). Please use the passive voice.
- Please check your synopsis text and image, revise them if necessary and submit their final versions with your revised manuscript. Please be aware that in the proof stage minor corrections only are allowed (e.g., typos).
6) As part of the EMBO Publications transparent editorial process initiative (see our Editorial at http://embomolmed.embopress.org/content/2/9/329), EMBO Molecular Medicine will publish online a Review Process File (RPF) to accompany accepted manuscripts. This file will be published in conjunction with your paper and will include the anonymous referee reports, your point-by-point response and all pertinent correspondence relating to the manuscript. Let us know whether you agree with the publication of the RPF and as here, if you want to remove or not any figures from it prior to publication. Please note that the Authors checklist will be published at the end of the RPF.
7) Please provide a point-by-point letter INCLUDING my comments as well as the reviewer's reports and your detailed responses (as Word file).

I look forward to reading a new revised version of your manuscript as soon as possible.

Yours sincerely,

Zeljko Durdevic

***** Reviewer's comments *****

Referee #1 (Remarks for Author):

The authors correctly responded to the reviewer's comment and performed the requested additional experiments. The article is now ready for publication in the journal.

Referee #3 (Comments on Novelty/Model System for Author):

The mechanism was well clarified.

Referee #3 (Remarks for Author):

The present version is suitable for publication.

The authors performed the requested editorial changes.

2nd Revision - Editorial Decision **22nd Oct 2021**

We are pleased to inform you that your manuscript is accepted for publication and is now being sent to our publisher to be included in the next available issue of EMBO Molecular Medicine.

EMBO PRESS

YOU MUST COMPLETE ALL CELLS WITH A PINK BACKGROUND ➔

PLEASE NOTE THAT THIS CHECKLIST WILL BE PUBLISHED ALONGSIDE YOUR PAPER

Corresponding Author Name: Songying Zhang, Heng-Yu Fan, Ge Lin

Journal Submitted to: EMBO Molecular Medicine
Manuscript Number: EMM-2021-14887-V2

USEFUL LINKS FOR COMPLETING THIS FORM

http://www.antibodypedia.com
http://1degreebio.org
http://www.equator-network.org/reporting-guidelines/improving-bioscience-research-repor
http://grants.nih.gov/grants/olaw/olaw.htm
http://www.mrc.ac.uk/Ourresearch/Ethicsresearchguidance/Useofanimals/index.htm
http://ClinicalTrials.gov
http://www.consort-statement.org
http://www.consort-statement.org/checklists/view/32-consort/66-title
http://www.equator-network.org/reporting-guidelines/reporting-recommendations-for-tumo
http://datadryad.org
http://figshare.com
http://www.ncbi.nlm.nih.gov/gap
http://www.ebi.ac.uk/ega
http://biomodels.net/
http://biomodels.net/miriam/
http://jjj.biochem.sun.ac.za
https://osp.od.nih.gov/biosafety-biosecurity-and-emerging-biotechnology/
http://www.selectagents.gov/

Reporting Checklist For Life Sciences Articles (Rev. June 2017)

This checklist is used to ensure good reporting standards and to improve the reproducibility of published results. These guidelines are consistent with the Principles and Guidelines for Reporting Preclinical Research issued by the NIH in 2014. Please follow the journal's authorship guidelines in preparing your manuscript.

A- Figures

1. Data

The data shown in figures should satisfy the following conditions:

- ➔ the data were obtained and processed according to the field's best practice and are presented to reflect the results of the experiments in an accurate and unbiased manner.
- ➔ figure panels include only data points, measurements or observations that can be compared to each other in a scientifically meaningful way.
- ➔ graphs include clearly labeled error bars for independent experiments and sample sizes. Unless justified, error bars should not be shown for technical replicates.
- ➔ if n< 5, the individual data points from each experiment should be plotted and any statistical test employed should be justified
- ➔ Source Data should be included to report the data underlying graphs. Please follow the guidelines set out in the author ship guidelines on Data Presentation.

2. Captions

Each figure caption should contain the following information, for each panel where they are relevant:

- ➔ a specification of the experimental system investigated (eg cell line, species name).
- ➔ the assay(s) and method(s) used to carry out the reported observations and measurements
- ➔ an explicit mention of the biological and chemical entity(ies) that are being measured.
- ➔ an explicit mention of the biological and chemical entity(ies) that are altered/varied/perturbed in a controlled manner.

- ➔ the exact sample size (n) for each experimental group/condition, given as a number, not a range;
- ➔ a description of the sample collection allowing the reader to understand whether the samples represent technical or biological replicates (including how many animals, litters, cultures, etc.).
- ➔ a statement of how many times the experiment shown was independently replicated in the laboratory.
- ➔ definitions of statistical methods and measures:
  - common tests, such as t-test (please specify whether paired vs. unpaired), simple χ^2 tests, Wilcoxon and Mann-Whitney tests, can be unambiguously identified by name only, but more complex techniques should be described in the methods section;
  - are tests one-sided or two-sided?
  - are there adjustments for multiple comparisons?
  - exact statistical test results, e.g., P values = x but not P values < x;
  - definition of 'center values' as median or average;
  - definition of error bars as s.d. or s.e.m.

Any descriptions too long for the figure legend should be included in the methods section and/or with the source data.

In the pink boxes below, please ensure that the answers to the following questions are reported in the manuscript itself. Every question should be answered. If the question is not relevant to your research, please write NA (non applicable). We encourage you to include a specific subsection in the methods section for statistics, reagents, animal models and human subjects.

B- Statistics and general methods

Please fill out these boxes ➔ (Do not worry if you cannot see all your text once you press return)

1.a. How was the sample size chosen to ensure adequate power to detect a pre-specified effect size?	The sample size is similar to previous reports in the same field. We screened 120 infertile patients exhibiting early embryonic arrest and fragmentation and only biallelic variants of MOS (OMIM ID: 190060; NM_005372.1) were found in three affected individuals, yet absent in our 100-controls undergoing natural conception database. This suggested the possible genetic contribution of MOS to human female infertility.
1.b. For animal studies, include a statement about sample size estimate even if no statistical methods were used.	We used at least 3 Erk1/2 conditional knockout mice to collect oocytes or embryos for phenotype analysis, which at least 60 oocytes or embryos were obtained. In each independent microinjection experiment, GV oocytes were collected from 25-30 wild-type female mice and randomly divided into 5-6 groups with 150-200 oocytes in each group.
2. Describe inclusion/exclusion criteria if samples or animals were excluded from the analysis. Were the criteria pre-established?	NA
3. Were any steps taken to minimize the effects of subjective bias when allocating animals/samples to treatment (e.g. randomization procedure)? If yes, please describe.	All collected oocytes from wild-type mice were randomized into experimental groups for subsequent microinjection. Then, the survived oocytes in each group were used for experiments and analysis.
For animal studies, include a statement about randomization even if no randomization was used.	NA
4.a. Were any steps taken to minimize the effects of subjective bias during group allocation or/and when assessing results (e.g. blinding of the investigator)? If yes please describe.	In order to minimize the effects of subjective bias, cell transfection, western blot and immunofluorescence staining were performed by two independent investigators. The confocal images were captured with the same parameters in each group.
4.b. For animal studies, include a statement about blinding even if no blinding was done	For the oocyte or embryo phenotype analysis from wild-type and Erk1-/-;Erk2f/f;Gdf9-Cre mice, age-matched animals were grouped based on their genotype, which was not blinded.
5. For every figure, are statistical tests justified as appropriate?	Yes.Differences between two groups were compared using the unpaired two-tailed Student's t-test. In the case three or more groups, one-way ANOVA was used, followed by post hoc Tukey's test for multiple comparisons. P < 0.05 was considered statistically significant. The statistical methods are indicated in the figure legends.
Do the data meet the assumptions of the tests (e.g., normal distribution)? Describe any methods used to assess it.	Yes. Data are presented as mean ± standard deviation (SD) of minimal independent samples. Studies with sufficient N's, we used the box plot to confirm data variability and the degree of normality.

Is there an estimate of variation within each group of data?	Yes. Standard deviation (SD) were generated to show the variation around the mean.
Is the variance similar between the groups that are being statistically compared?	No. We did not assume equal variance between groups.

C- Reagents

6. To show that antibodies were profiled for use in the system under study (assay and species), provide a citation, catalog number and/or clone number, supplementary information or reference to an antibody validation profile. e.g., Antibodypedia (see link list at top right), 1DegreeBio (see link list at top right).	Yes. The information (cat. number, company) about the antibodies and reagents used in this study was included in the materials and methods section in the manuscript.
7. Identify the source of cell lines and report if they were recently authenticated (e.g., by STR profiling) and tested for mycoplasma contamination.	NA

* for all hyperlinks, please see the table at the top right of the document

D- Animal Models

8. Report species, strain, gender, age of animals and genetic modification status where applicable. Please detail housing and husbandry conditions and the source of animals.	Yes. The information regarding strain, gender, age, genetic modification, source of mice and housing conditions was included in the materials and methods section in the manuscript.
9. For experiments involving live vertebrates, include a statement of compliance with ethical regulations and identify the committee(s) approving the experiments.	All animal experiments were performed in accordance with the guidelines of the Animal Committee of Zhejiang University.
10. We recommend consulting the ARRIVE guidelines (see link list at top right) (PLoS Biol. 8(6), e1000412, 2010) to ensure that other relevant aspects of animal studies are adequately reported. See author guidelines, under 'Reporting Guidelines'. See also: NIH (see link list at top right) and MRC (see link list at top right) recommendations. Please confirm compliance.	Yes.

E- Human Subjects

11. Identify the committee(s) approving the study protocol.	Yes, we set this tudy with the approval of ethical committee.
12. Include a statement confirming that informed consent was obtained from all subjects and that the experiments conformed to the principles set out in the WMA Declaration of Helsinki and the Department of Health and Human Services Belmont Report.	Yes, we obtained the informed consent from all subjects for sample collection and further experiments.
13. For publication of patient photos, include a statement confirming that consent to publish was obtained.	Yes, we obtained the approval for photo publication in the informed consent from all the three patients.
14. Report any restrictions on the availability (and/or on the use) of human data or samples.	NA
15. Report the clinical trial registration number (at ClinicalTrials.gov or equivalent), where applicable.	NA
16. For phase II and III randomized controlled trials, please refer to the CONSORT flow diagram (see link list at top right) and submit the CONSORT checklist (see link list at top right) with your submission. See author guidelines, under 'Reporting Guidelines'. Please confirm you have submitted this list.	NA
17. For tumor marker prognostic studies, we recommend that you follow the REMARK reporting guidelines (see link list at top right). See author guidelines, under 'Reporting Guidelines'. Please confirm you have followed these guidelines.	NA

F- Data Accessibility

18: Provide a "Data Availability" section at the end of the Materials & Methods, listing the accession codes for data generated in this study and deposited in a public database (e.g. RNA-Seq data: Gene Expression Omnibus GSE39462, Proteomics data: PRIDE PXD000208 etc.) Please refer to our author guidelines for 'Data Deposition'.<br><br>Data deposition in a public repository is mandatory for:<br>a. Protein, DNA and RNA sequences<br>b. Macromolecular structures<br>c. Crystallographic data for small molecules<br>d. Functional genomics data<br>e. Proteomics and molecular interactions	Yes, we have provided the accession codes for data generated in this study in the section of "Data Availability" in the manuscript.
19. Deposition is strongly recommended for any datasets that are central and integral to the study; please consider the journal's data policy. If no structured public repository exists for a given data type, we encourage the provision of datasets in the manuscript as a Supplementary Document (see author guidelines under 'Expanded View' or in unstructured repositories such as Dryad (see link list at top right) or Figshare (see link list at top right).	The raw data of RNA-seq was deposited in GSA-Human. Two Expanded View Datasets and one Supplementart Document were included in the manuscript.
20. Access to human clinical and genomic datasets should be provided with as few restrictions as possible while respecting ethical obligations to the patients and relevant medical and legal issues. If practically possible and compatible with the individual consent agreement used in the study, such data should be deposited in one of the major public access-controlled repositories such as dbGAP (see link list at top right) or EGA (see link list at top right).	With informed consent's agreement, we deposited raw data generated in this study in a public access-controlled repository, GSA-Human (https://bigd.big.ac.cn/gsa-human/browse/HRA001269).
21. Computational models that are central and integral to a study should be shared without restrictions and provided in a machine-readable form. The relevant accession numbers or links should be provided. When possible, standardized format (SBML, CellML) should be used instead of scripts (e.g. MATLAB). Authors are strongly encouraged to follow the MIRIAM guidelines (see link list at top right) and deposit their model in a public database such as Biomodels (see link list at top right) or JWS Online (see link list at top right). If computer source code is provided with the paper, it should be deposited in a public repository or included in supplementary information.	NA

G- Dual use research of concern

22. Could your study fall under dual use research restrictions? Please check biosecurity documents (see link list at top right) and list of select agents and toxins (APHIS/CDC) (see link list at top right). According to our biosecurity guidelines, provide a statement only if it could.	NA
